# Systems pharmacology-based dissection of mechanisms of Chinese medicinal formula Bufei Yishen as an effective treatment for chronic obstructive pulmonary disease

**DOI:** 10.1038/srep15290

**Published:** 2015-10-15

**Authors:** Jiansheng Li, Peng Zhao, Ya Li, Yange Tian, Yonghua Wang

**Affiliations:** 1 Henan University of Traditional Chinese Medicine, Zhengzhou 450046, China; 2 Collaborative Innovation Center for Respiratory Disease Diagnosis and Treatment & Chinese Medicine Development of Henan Province, Zhengzhou 450046, China; 3 Center of Bioinformatics, Northwest A & F University, Yangling, Shaanxi 712100, China

## Abstract

The present work adopted a systems pharmacology-based approach to provide new insights into the active compounds and therapeutic targets of Bufei Yishen formula (BYF) for the treatment of chronic obstructive pulmonary disease (COPD). In addition, we established a rat model of cigarette smoke- and bacterial infection-induced COPD to validate the mechanisms of BYF action that were predicted in systems pharmacology study. The systems pharmacology model derived 216 active compounds from BYF and 195 potential targets related to various diseases. The compound-target network showed that each herbal drug in the BYF formula acted on similar targets, suggesting potential synergistic effects among these herbal drugs. The ClueGo assay, a Cytoscape plugin, revealed that most targets were related to activation of MAP kinase and matrix metalloproteinases. By using target-diseases network analysis, we found that BYF had great potential to treatment of multiple diseases, such as respiratory tract diseases, immune system, and cardiovascular diseases. Furthermore, we found that BYF had the ability to prevent COPD and its comorbidities, such as ventricular hypertrophy, *in vivo*. Moreover, BYF inhibited the inflammatory cytokine, and hypertrophic factors expression, protease-antiprotease imbalance and the collagen deposition, which may be the underlying mechanisms of action of BYF.

Chronic obstructive pulmonary disease (COPD) is defined as a slowly progressive condition characterized by poorly reversible airflow limitation and systemic inflammation, which represents a substantial economic and social burden throughout the world^1^,^2^. Drug therapies that include bronchodilators, steroids, and phosphodiesterase inhibitor become the main methods in treating. However, despite great progress in COPD treatment, therapies have not changed significantly in the past 20 years because of the underlying pulmonary inflammation or airway and lung tissue remodeling COPD^3^,^4^. Novel therapeutic drugs and strategies accordingly are urgently needed to improve the efficacy in treating this deadly disease.

In recent years, a great deal of attention has been paid to develop Chinese herbal medicine for treatment of COPD. Bufei Yishen formula (BYF), a traditional Chinese medicine, is composed of twelve medicinal herbs and has been shown to possess extensive pharmacological effects on COPD, including alleviating the clinical symptoms of stable COPD patients, reducing the exacerbation frequency, delaying acute exacerbation, and improving pulmonary function and exercise capacity^5^. However, the pharmacological mechanisms of action and active substances of BYF remain poorly understood and warrant further investigation.

Generally, most traditional Chinese medicines (TCM) exert therapeutic effect by targeting multiple molecules of the human body. However, it is difficult to identify the multiple targets of the Chinese herb medicines. Similarly, tens of thousands ingredients involved in the most formulas make it hard to identify active compound. Therefore, a novel strategy that can clarify the active compounds and therapeutic targets of herbal drug, is urgently needed to be developed^6^.

Systems pharmacology is an emerging field that combines oral bioavailability prediction, multiple drug targets prediction and network analysis to understand the active compounds and therapeutic targets of TCM^7^,^8^,^9^. In this study, we proposed a systems pharmacology approach to investigate the pharmacological mechanisms of action and active substances of BYF by systematically incorporating active compounds prediction, therapeutic targets prediction, and drug-target-disease network analysis. Furthermore, we administered BYF to COPD model rats and investigated the effect of BYF on cigarette smoke- and bacterial infection-induced pulmonary inflammatory responses, protease-antiprotease imbalance, collagen deposition and respiratory dysfunction. These results further supplied the *in vivo* experimental evidence to validate the mechanisms of action of BYF that were predicted in the systems pharmacology. The flowchart of systems pharmacology approach was shown in Fig. 1.

## Results and Discussion

### Candidate compounds screening

Much effort has been made in clarifying the therapeutic mechanism of action of TCM, but complex chemical composition of TCM makes it hard to understand its mechanism from a molecular level. However, effective methods which are specifically developed for the identification of the active compounds contained in medicinal herbs are not available up to now. Therefore, a combination of Oral bioavailability (OB) screening and drug-likeness property evaluation was applied to explore the active substance of TCM, such as BYF^10^,^11^. Then, 181 potential compounds with OB ≥ 30% and drug-likeness index ≥0.18 were collected from the herbal constituents of BYF. Additionally, 35 compounds with poor OB (≤30%) or drug-likeness indices (≤0.18) possessed extensive pharmacological activities, which were the typical component of herbal drugs. Thus, these compounds were also collected as the candidate compounds for further analysis. We found that all compounds of Pheretima (a constituent of BYF) were excluded because its compounds showed poor OB or drug-likeness indices. Finally, 216 compounds of the 11 herbs were considered as “active compounds,” including the 181 readily absorbed compounds and the 35 pharmacologically active compounds (Supplementary Table S1). The numbers of active compounds in Ginseng Radix et Rhizoma (GRR), Astragali Radix (AR), Corni Fructus (CF), Lycii Fructus (LF), Schisandrae Chinensis Fructus (SCF), Epimedii Herba (EH), Fritillariae Thunbergii Bulbus (FTB), Paeoniae Rubra Radix (PRR), Perillae Fructus (PF), Ardisiae Japonicae Herba (AJH), and Citri Reticulatae Pericarpium (CRP) was 22, 20, 35, 45, 10, 27, 9, 36, 16, 20 and 8, respectively. Among these, paeonioflorin, icariin, and nobiletin, which are the typical compounds of PRR, EH, and CRP, have been found to have extensive pharmacological activities, such as anti-inflammatory, immunomodulatory and analgesic^11^,^12^,^13^. Similarly, luteolin and quercetin, which occur widely in AR, LF AJH, and EH, have been demonstrated to possess extensive biological and pharmacological properties in the current studies^14^,^15^.

### Target prediction

Generally, TCM formula can effectively prevent the complex diseases through the synergistic effects of multiple compounds and targets^9^. Therefore, besides clarifying the active compounds of BYF, the therapeutic targets exploration is also required. In this study, we used the pharmacophore modeling approach to predict the potential targets based on the candidate compounds. The results indicated that 195 potential targets were predicted for the 182 candidate compounds, and 34 candidate compounds had no corresponding targets based on this method (Supplementary Table S2, 3). Finally, 182 candidate compounds had interactions with 195 potential targets, and the connections between them reached 2268.

The numbers of potential targets connected by GRR, AR, CF, LF, SCF, EH, FTB, PRR, PF, AJH and CRP were 101, 134, 99, 132, 26, 140, 58, 91, 108, 99, and 61, respectively. Although the numbers of each herb-related target were different, there was a significant target overlap between the 11 herbs. These results suggested that different herbal drugs contained in the BYF could regulate these similar targets to exert synergistic effect. For example, many ingredients including paeoniflorin, quercetin, peimisine and bergenin are involved in mediating the activation of inducible nitric oxide synthase, which may provide synergistic therapeutic effects to benefit patients.

### Compound-target network

**Figure 2:**
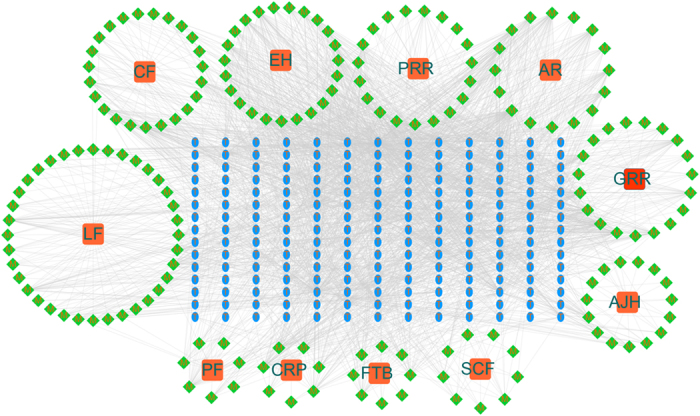
Compound-target network. The compound-target network was constructed by linking the herbs, candidate compounds and all their potential targets. The nodes represent herbs (orange squares), candidate compounds (green rhombus) and targets (blue ellipse). (GRR, Ginseng Radix et Rhizoma; AR, Astragali Radix; CF, Corni Fructus; LF, Lycii Fructus; SCF, Schisandrae Chinensis Fructus; EH, Epimedii Herba; FTB, Fritillariae Thunbergii Bulbus; PRR, Paeoniae Rubra Radix; PF, Perillae Fructus; AJH, Ardisiae Japonicae Herba; CRP, Citri Reticulatae Pericarpium).

TCM formula exerts extensive biological and pharmacological effects through multiple compounds and targets. To understand the complex interaction of compounds and their corresponding targets at a systems level, we constructed the compound-target network based on the candidate compounds of BYF and the potential targets. As shown in Fig. 2, the compound-target network embodies 388 nodes (11 herbs, 182 candidate compounds and 195 potential targets) and 2268 compound-target interactions. The mean degree value (the number of target associated with it) of candidate compounds was 12.5, and 59 compounds possess degree larger than 13, which indicated that most compounds regulated multiple targets to exert various therapeutic effects. Specially, three compounds, such as quercetin, kaempferol and beta-sitosterol, which acted on 87, 55 and 51 targets, respectively, become the crucial active compounds for the BYF due to their important positions in this network. Taking kaempferol as an example, this compound exhibits extensive pharmacological activity, such as anti-inflammatory, anti-hypertensive and anti-oxidative properties, by mediating the activation of inducible nitric oxide synthase, prostaglandin G/H synthase 1 and peroxisome proliferator activated receptor gamma^16^. Additionally, the results indicated that many targets were hit by multiple compounds in the compound-target network. Estrogen receptor and prostaglandin G/H synthase 2, peroxisome proliferator activated receptor gamma are targeted by 122, 83 and 60 compounds. These targets play important pathological roles including inflammation and immune system activation^17^,^18^.

**Figure 3:**
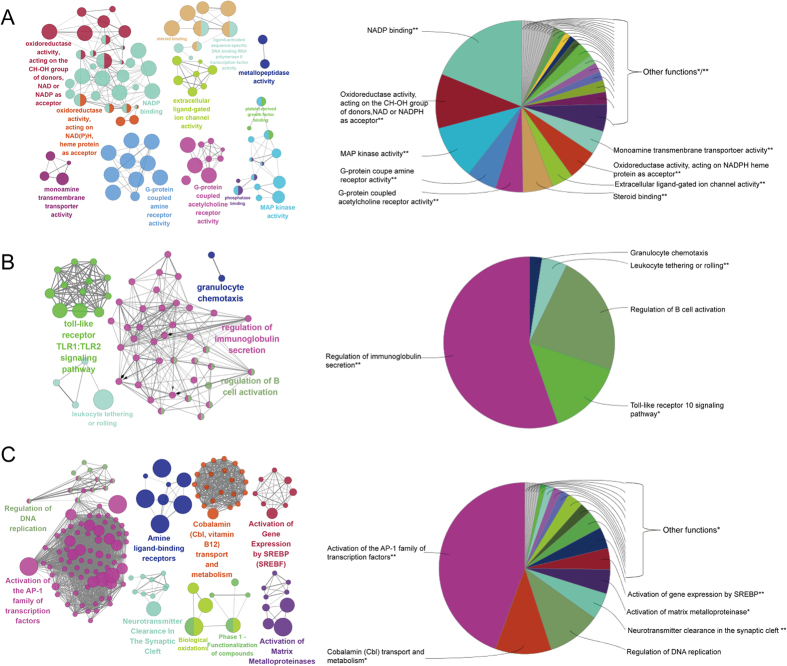
ClueGO analysis of the predicted targets. Functionally grouped network of enriched categories was generated for the target genes. GO terms are represented as nodes, and the node size represents the term enrichment significance. Functionally related groups partially overlap. The node pie charts represent the molecular function, immunesystem processes, reactome analysis of targets. Only the most significant term in the group was labeled. (**A**) Representative molecular function interactions among targets. (**B**) Representative immune system processes interactions among targets. (**C**) Representative reactome analysis interactions among predicted targets.

To decipher the action mechanism of 195 potential targets, ClueGO, a widely used Cytoscape plugin, was applied to identify biological interpretation and interrelations of functional groups in biological networks^19^. As shown in Fig. 3, the results were divided into three stratums: molecular functions, the immunesystem processes, and the reactome analysis. Specially, the molecular functions were mainly consisted of four groups: NADP binding, oxidoreductase activity, MAP kinase activity, and G-protein coupled amine receptor activity, which indicated that most potential targets were related to oxidoreductase activity and MAP kinase activity (Fig. 3A). The immunesystem processes of the targets were mainly contained four groups, including regulation of immunoglobulin secretion, toll-like receptor 10 signaling pathway, regulation of B cell activation, and leukocyte tethering or rolling (Fig. 3B). The reactome of the targets were mainly related to activation of the activator protein (AP)-1 family of transcription factors, cobalamin transport and metabolism, regulation of DNA replication, and activation of matrix metalloproteinases (Fig. 3C). Finally, we found that most of the targets were related to the activation of MAP kinase, AP-1 family of transcription factors and matrix metalloproteinases and regulation of immunoglobulin secretion. These biological functions have been linked to the pathogenesis of various diseases including COPD, asthma and other respiratory diseases^20^,^21^.

### Target-disease network

**Figure 4:**
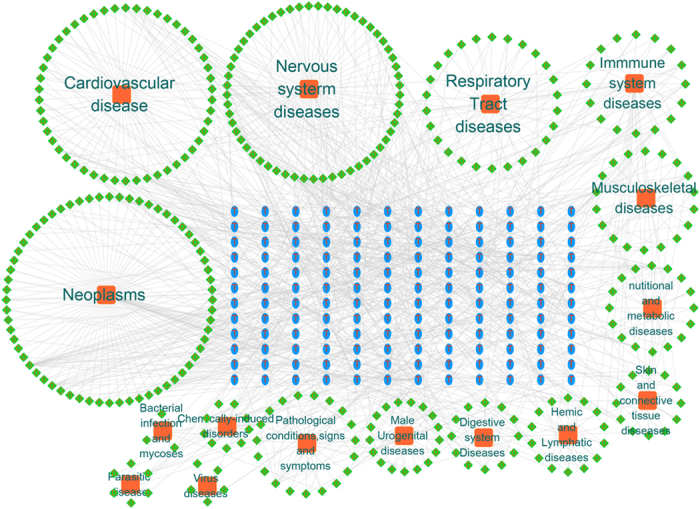
Target-disease network. The target-disease network was built by linking the potential targets and all their corresponding diseases. 145 target proteins (blue ellipse) were connected to 358 diseases (green rhombus) which were divided into 16 groups (orange square) based on Medical Subject Headings.

In order to get a better understanding of the diseases related to BYF, the potential targets were projected to DrugBank, TTD and PharmGkb databases to collect their corresponding diseases. Then, 389 diseases were classified into 16 groups according to the MeSH Browser (2014 MeSH). Finally, Target-disease network was constructed based on potential targets and their corresponding diseases. As shown in Fig. 4, lots of collected diseases belong to neoplasms (84/350), nervous system diseases (67/350), cardiovascular diseases (50/350), and immmune system diseases (35/350) apart from respiratory tract diseases (26/350), which implied that BYF may have effectiveness not only on respiratory diseases but also on these diseases. These results supplied the systematic evidence for the TCM theory that a TCM formula has extensive pharmacological activities and is successful applied into the treatment of various diseases. For example, we found that prostaglandin G/H synthase 2, an important target of BYF, connected with immunity and inflammation-associated diseases, cancer and respiratory diseases, such as inflammatory bowel disease, colorectal cancer, asthma, COPD and other airway diseases, in the target-disease network. Many studies also suggested that prostaglandin G/H synthase 2, a kind of anti-inflammatory drug target, has been link to the pathogenesis of these diseases, and tractable as a new therapeutic target^22^,^23^.

Taken together, we supplied systems pharmacology methods to predict the mechanism of action of BYF by dissecting the effective substances of BYF, their potential targets, and compound-target-diseases network. Based on these findings, BYF contained numerous effective substances with different pharmacologic properties that act on multiple targets with potential synergistic effects. Moreover, to verify further the systems pharmacology predictions, experimental studies were conducted.

### Effect of BYF on COPD and its comorbidity

Previous study showed that BYF had been successful applied into the treatment of COPD patients^5^. In this study, we predicted that BYF was effective on treating various diseases, including respiratory disease, nervous system diseases, cardiovascular disease and cancer. In order to confirm this prediction, we investigated the effect of BYF on COPD rats and its comorbidity, ventricular hypertrophy.

**Figure 5:**
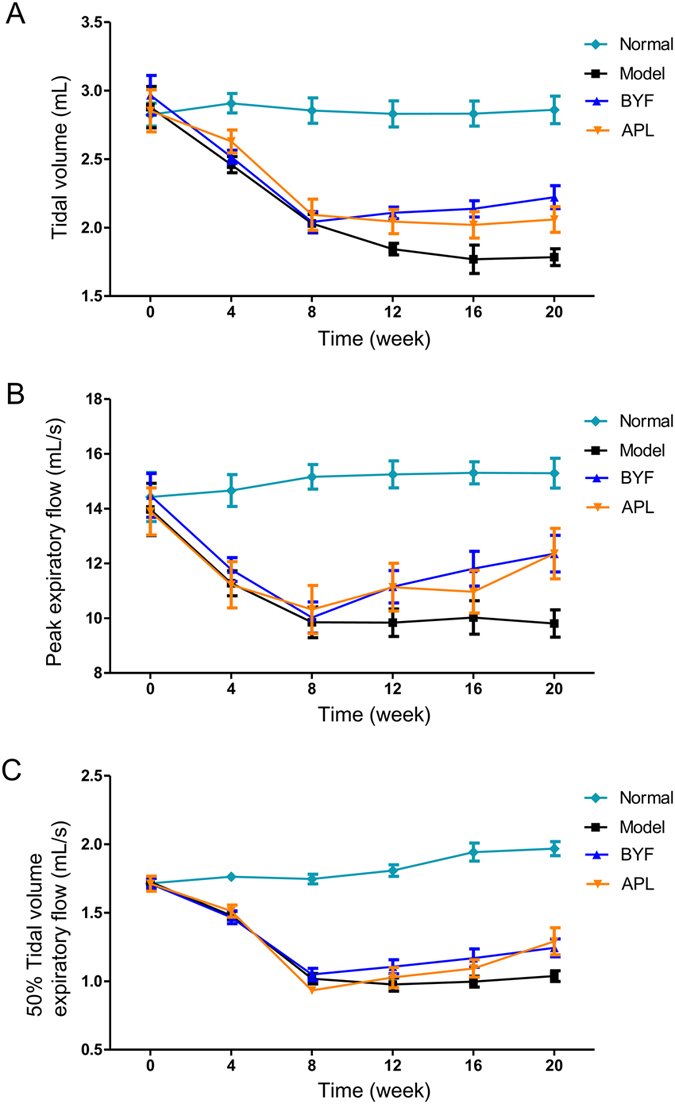
*In vivo* therapeutic efficacies of Bufei Yishen formula (BYF) on the pulmonary function of chronic obstructive pulmonary disease (COPD) rats. The COPD rats were intragastricly treated with 4.44 g/kg of BYF once daily. Control was treated with normal saline, and positive control was treated with aminophylline (APL, 2.3 mg/kg) once daily. Tidal volume (TV) (**A**), peak expiratory flow (PEF) (**B**), and 50% tidal volume expiratory flow (EF50) (**C**) were detected every fourth week from weeks 0 to 20. Results were given as means ± SEM, n = 10.

**Figure 6:**
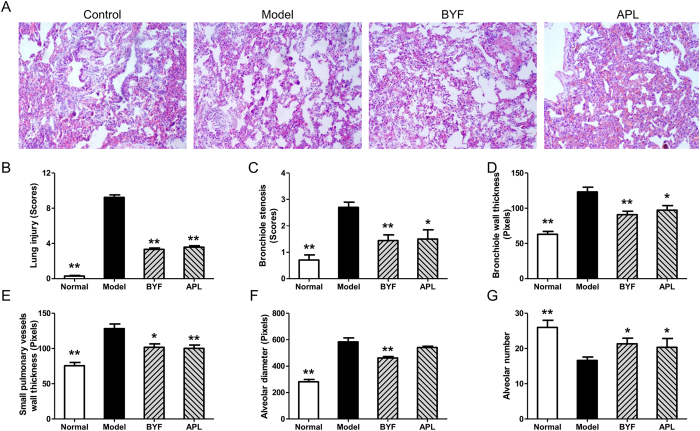
Effect of Bufei Yishen formula (BYF) on the histological changes in lung tissues of chronic obstructive pulmonary disease (COPD) rats. COPD rats were intragastricly treated with BYF (4.44 g/kg) and aminophylline (APL, 2.3 mg/kg) once daily. Histopathologic changes of the lung tissues were analyzed on week 20 (HE staining, magnification, ×100) (**A**). The lung injury scores of all groups were detected (**B**). bronchiole stenosis (**C**), bronchial wall thickness (**D**), Small pulmonary vessels wall thickness (**E**), alveolar number (**F**) and alveolar diameter (**G**) were evaluated. Values represent means ± SEM, n = 10. *P < 0.05, **P < 0.01 vs. model.

To evaluate the effect of BYF on COPD rats, a rat model of cigarette smoke- and bacterial infection-induced COPD was therefore established, and pulmonary function and histopathology were assessed. As shown in Fig. 5, compared with the model rats, BYF increased the TV and PEF in the COPD rats at week 20. Aminophylline (APL), a classical bronchodilator, also increased the TV and PEF in COPD rats (Fig. 5A,B). In addition, BYF and APL could increase the EF50 at week 20 (Fig. 5C). Furthermore, lung injury scores, bronchiole wall thickness, small pulmonary vessels wall thickness, bronchiole stenosis, and alveolar diameter increased in the model rat, and this increase was significantly suppressed by BYF (Fig. 6A–F). In addition, BYF also markedly increased the alveolar number in COPD rats (Fig. 6G). These results demonstrated that BYF is effective on treating COPD.

**Figure 7:**
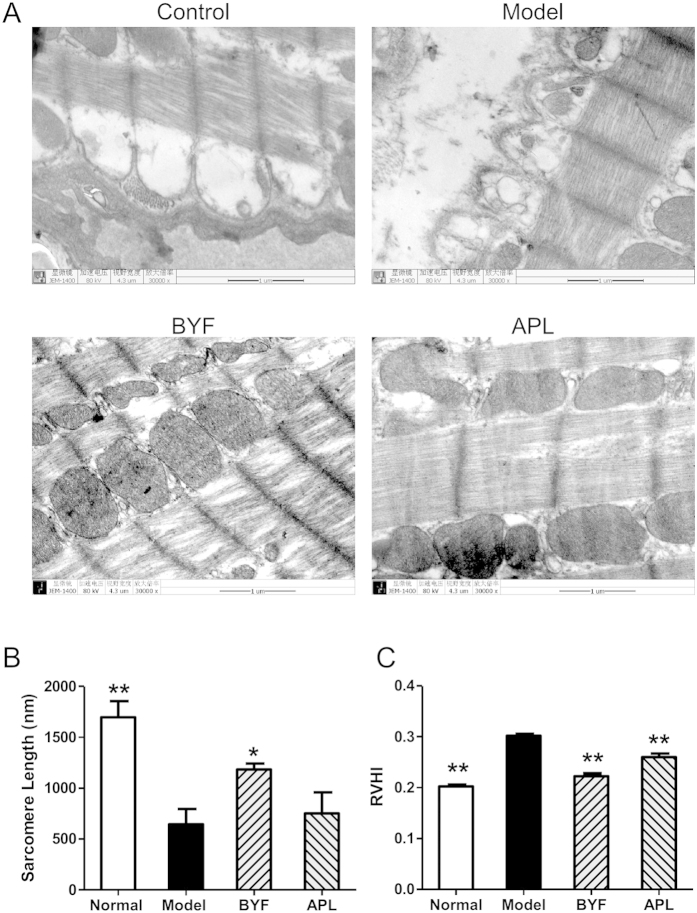
Effect of Bufei Yishen formula (BYF) on the myocardial ultrastructure and the right ventricular hypertrophy index (RVHI) changes in chronic obstructive pulmonary disease (COPD) rats. COPD rats were intragastricly treated with BYF (4.44 g/kg) and aminophylline (APL, 2.3 mg/kg) once daily. The myocardial ultrastructural changes were analyzed by transmission electron microscope analysis on week 20. The muscular fibers (**A**) (magnification, ×30,000) of myocardial ultrastructure images were taken. The sarcomere length (**B**), and RVHI (**C**) were also evaluated. Values represent means ± SEM, n = 8. *P < 0.05, **P < 0.01 vs. model.

**Figure 8:**
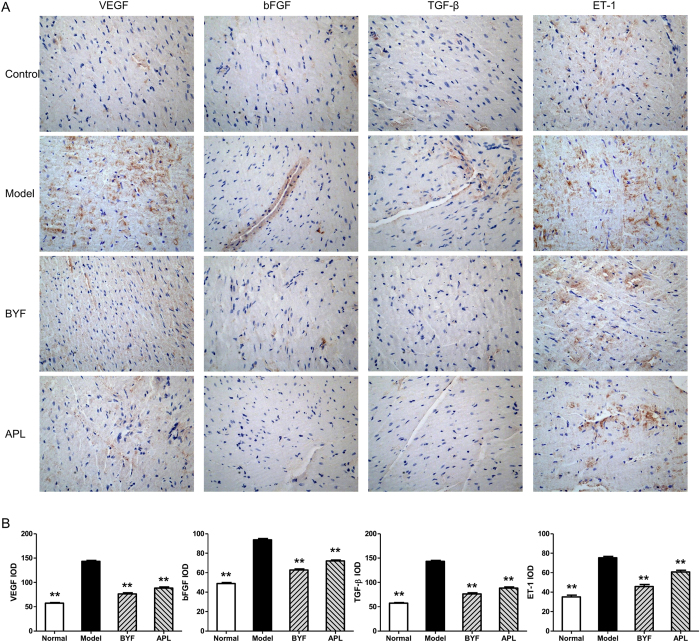
Effect of Bufei Yishen formula (BYF) on the expression of VEGF, bFGF, TGF-β, and ET-1 in the right ventricle obtained from chronic obstructive pulmonary disease (COPD) rats. COPD rats were intragastricly treated with BYF (4.44 g/kg) and aminophylline (APL, 2.3 mg/kg) once daily. VEGF, bFGF, TGF-β and ET-1 in right ventricle of COPD rats expression were analyzed by immunohistochemistry (magnification, ×100) (**A**). The expression of VEGF, bFGF, TGF-β and ET-1 was quantitatively analyzed (**B**). Values represent means ± SEM, n = 8. **P < 0.01 vs. model. VEGF, vascular endothelial growth factor; bFGF, basic fibroblast growth factor; TGF-β, transforming growth factor-β; and ET-1, endothelin-1.

Pharmacologic treatment of chronic disease, such as COPD is complex, especially considering that single drug is usually developed for single diseases. However, TCM formula designed for one complex disease may also favorably affect other diseases, including its comorbidities^9^. In this study, we also found that rats with COPD are at an increased risk of ventricular hypertrophy. We thus investigated the effect of BYF on ventricular hypertrophy. As shown in Fig. 7, compared with the model rats, BYF markedly increased the myocardial sarcomere length of cardiocytes and reversed the RVHI at week 20. In addition, we evaluated the effect of BYF on the expression of hypertrophic factors, such as VEGF, bFGF, ET-1, and TGF-β. The result showed that BYF and APL significantly suppressed the expression of VEGF, bFGF, TGF-β, and ET-1 (Fig. 8).

These results demonstrated that BYF treatment had significant effects on COPD and ventricular hypertrophy, and these findings supplied the direct experimental evidences for the systems pharmacology prediction.

### Effect of BYF on inflammatory responses in COPD rats

**Figure 9:**
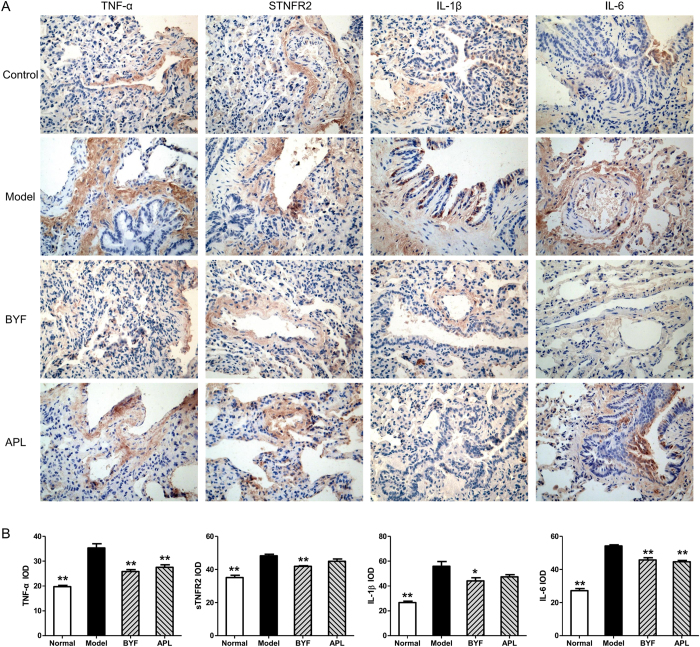
Effect of Bufei Yishen formula (BYF) on the expression of TNF-α, sTNFR2, IL-1β and IL-6 in the lung of chronic obstructive pulmonary disease (COPD) rats. COPD rats were intragastricly treated with BYF (4.44 g/kg) and aminophylline (APL, 2.3 mg/kg) once daily. The expression of tumor necrosis factor (TNF)-α, soluble TNF-α receptor (sTNFR) 2, interleukin (IL)- 1β, and IL-6 in lung tissues were characterized by immunohistochemistry (magnification, ×100) (**A**). Quantitative analysis for IL-6, IL-1β, TNF-α and sTNFR2 expression (**B**). Values represent means ± SEM, n = 10. *P < 0.05, **P < 0.01 vs. model.

In this study, the systems pharmacology prediction showed that lots of potential targets of BYF are related to activation of the AP-1 family of transcription factors and MAPK targets/nuclear events mediated by MAP. In addition, inflammatory response of the lungs has been linked to the COPD processes. Specially, the pro-inflammatory mediators, such as IL-1β, IL-6 and TNF-α, play important roles in the pathogenesis of COPD. In addition, these inflammatory mediators, expressed in the lung tissues of COPD patients, are regulated by MAPK/AP-1^1^,^24^,^25^. Therefore, we evaluated the effect of BYF on the expression of inflammatory mediators in the lung tissues of COPD rats. As shown in Fig. 9, compare the model rats, BYF significantly inhibited the expression of IL-1β, IL-6, TNF-α, and sTNFR2 induced by cigarette smoke and bacterial infection exposures. These findings demonstrated that BYF treatment could effectively inhibit the inflammatory response of the lungs, and the results are consistent with those obtained by target protein function analysis.

### Effect of BYF on collagen degradation and protease-antiprotease imbalance

The systems pharmacology results showed that many potential targets were related to activation of matrix metalloproteinases (MMPs). These zinc-dependent endopeptidases degrade the protein components of the extracellular matrix, and the imbalance of MMPs/anti-MMPs may induce destruction of the lung parenchyma and development of emphysema^26^,^27^,^28^. The collagen degradation makes great contribution to excessive remodeling, and accumulation of structural proteins, which causes the persistent tissue injury and airflow obstruction in COPD patients^29^.

**Figure 10:**
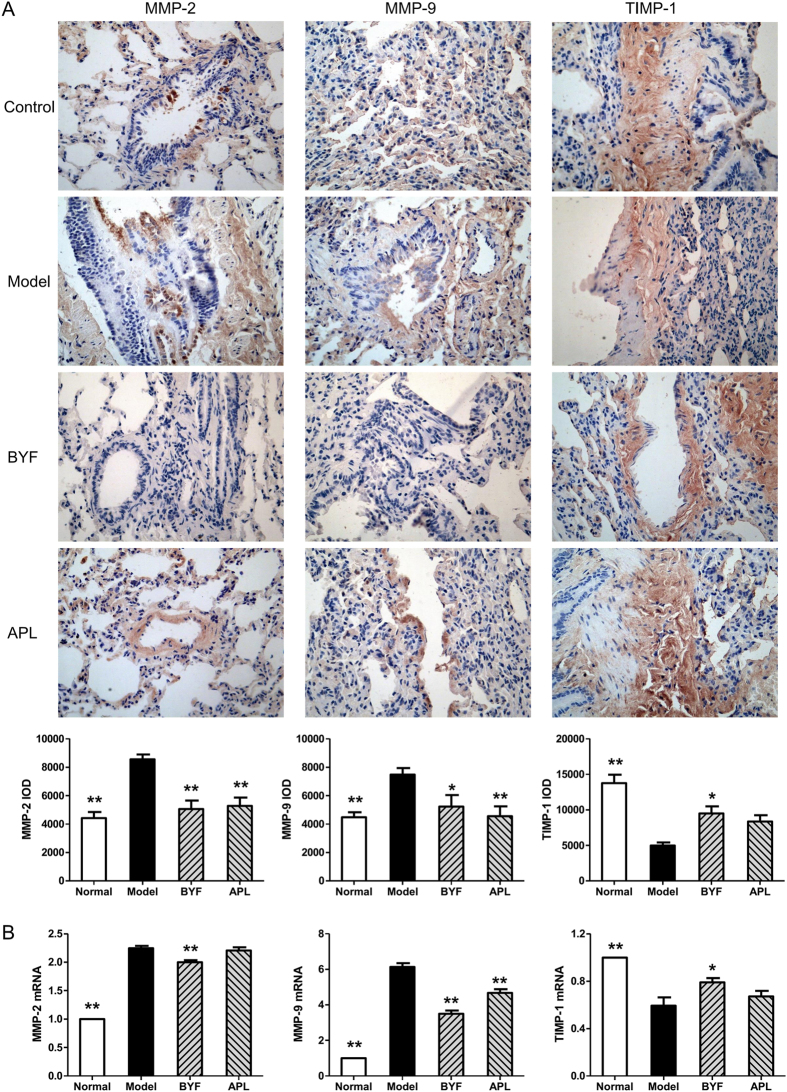
Effect of Bufei Yishen formula (BYF) on the expression of MMP-2, MMP-9 and TIMP-1 in the lung of chronic obstructive pulmonary disease (COPD) rats. COPD rats were intragastricly treated with BYF (4.44 g/kg) and aminophylline (APL, 2.3 mg/kg) once daily. Immunohistochemical and quantitative analysis for the expression of matrix metalloproteinase (MMP)-2, MMP-9, and tissue inhibitor of MMP (TIMP)-1 in the lung of COPD rats were performed on week 20 (magnification, ×100) (**A**). *MMP-2*, *MMP-9* and *TIMP-1* mRNA levels were analyzed by reverse transcriptase-polymerase chain reaction (RT-PCR) (**B**). Values represent means ± SEM, n = 10. *P < 0.05, **P < 0.01 vs. model.

**Figure 11:**
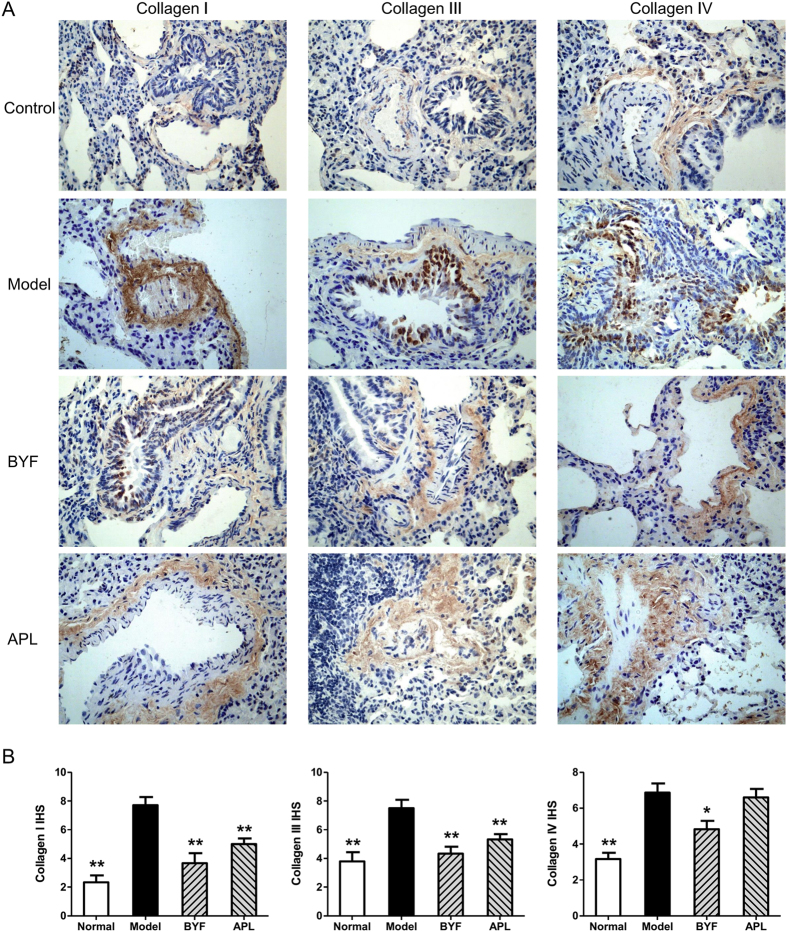
Effect of Bufei Yishen formula (BYF) on the expression of collagens I, III and IV in the lung tissues of chronic obstructive pulmonary disease (COPD) rats. COPD rats were intragastricly treated with BYF (4.44 g/kg) and aminophylline (APL, 2.3 mg/kg) once daily. Immunohistochemical staining on lung sections: collagen I, III and IV (magnification, ×100) (**A**). Collagens I, III and IV expression were quantitatively analyzed (**B**). Values represent means ± SEM, n = 10. *P < 0.05, **P < 0.01 vs. model.

To test this prediction of systems pharmacology, we thus investigated the effect of BYF on the expression of MMP-2, MMP-9, TIMP-1, and collagens I, III, and IV in lung tissues of COPD rats. As shown in Fig. 10A, BYF significantly suppressed the levels of MMP-2 and MMP-9 and increased the level of TIMP-1. This alteration also was observed at the mRNA level (Fig. 10B). Similarly, BYF treatment significantly suppressed the expression of collagens I, III, and IV induced by the cigarette smoke and bacterial infection exposures (Fig. 11). These findings indicated that BYF suppressed the cigarette smoke- and bacterial infection-induced collagen deposition and protease-antiprotease imbalance by modulating the expression of collagens I, III, and IV, MMP-2/9, and TIMP-1.

Taken together, we demonstrated that BYF provided protective and therapeutic benefits against cigarette smoke- and bacterial infection-induced pulmonary inflammation, collagen deposition, protease-antiprotease imbalance and hypertrophic factors production, suggesting that this formula could be effective for the treatment of COPD and its comorbidity, ventricular hypertrophy. These findings provided the powerful experimental evidences for the systems pharmacology predictions.

## Conclusion

In this study, we proposed an integrative systems pharmacology approach to dissect the active compounds and therapeutic targets of BYF by incorporating candidate compounds prediction, multiple drug target prediction and compound-target-disease network analysis. To create the annotations network, ClueGO provided functional analysis of potential targets, which is a useful strategy to predict the mechanism of action of BYF. Moreover, we experimentally validated that BYF was effective for the treatment of COPD and ventricular hypertrophy due to its inhibitory effect on the inflammatory cytokine, and hypertrophic factors expression, protease-antiprotease imbalance and collagen deposition.

In summary, the systems pharmacology approach constructed in this work have potential implications toward understanding the pharmacological mechanism of action and active substances of TCM, which may propel the new ways toward exploring new drug therapies for complex diseases.

## Materials and Methods

### Chemicals and animals

The tobacco (Hongqi Canal® Filter tip cigarette; tobacco type, tar: 10 mg; nicotine content: 1.0 mg; carbon monoxide: 12 mg) was purchased from Henan Tobacco Industry (Zhengzhou, China). *Klebsiella pneumoniae* (strain ID: 46114) was obtained from the National Center for Medical Culture Collection (CMCC, Beijing, China). Aminophylline was obtained from Shandong Xinhua Pharmaceutical Co., LTD. (Shandong, China). Mayer’s hematoxylin and 1% eosin alcohol solution were purchased from MUTO Pure Chemicals (Tokyo, Japan). Antibodies against interleukin (IL)-6, IL-1β, tumor necrosis factor (TNF)-α, soluble TNF-α receptor (sTNFR) 2, collagen I, collagen III, collagen IV, endothelin (ET)-1, transforming growth factor (TGF)-β, vascular endothelial growth factor (VEGF), basic fibroblast growth factor (bFGF), matrix metalloproteinase (MMP)-2, MMP-9, and TIMP metallopeptidase inhibitor (TIMP)-1 were purchased from Santa Cruz Biotechnology (Santa Cruz, CA, USA). Forty-two Sprague-Dawley rats (21 male and 21 female), weighing 180–220 g, were purchased from the Experimental Animal Center of Henan Province (Zhengzhou, China). The rats were raised under controlled temperature (26–28 °C), humidity (50 ± 10%) and daily light intensity (12 h light/12 h dark cycle), and were fed with standard laboratory food and water adlibitum. All animals were handled with humane care throughout the experiment. The animal experiments were conducted with the approval of the Experimental Animal Care and Ethics Committee of the First Affiliated Hospital, Henan University of Traditional Chinese Medicine. The methods were carried out in accordance with the approved guidelines of the Experimental Animal Care and Ethics Committee of the First Affiliated Hospital, Henan University of Traditional Chinese Medicine.

### Dataset construction

All ingredients of BYF were extracted from the Chinese Academy of Sciences Chemistry Database (http://www.organchem.csdb.cn), Traditional Chinese Medicine System Pharmacology Database (http://tcmspnw.com) and literatures, and then added into the ingredient database of BYF. Generally, glycosides can be metabolized rapidly to their de-glycosylation products by enteric bacteria after oral administration^30^. Therefore, based on the rule of glycosidase hydrolysis reaction, glycosides contained in the BYF were further deglycosylated, and the corresponding products were added into ingredient database. Taken together, a total of 1456 chemicals were included: 190 in Ginseng Radix et Rhizoma (GRR), 87 in Astragali Radix (AR), 226 in Corni Fructus (CF), 188 in Lycii Fructus (LF), 130 in Schisandrae Chinensis Fructus (SCF), 130 in Epimedii Herba (EH), 17 in Fritillariae Thunbergii Bulbus (FTB), 75 in Paeoniae Rubra Radix (PRR), 28 in Pheretima (Ph) 128 in Perillae Fructus (PF), 193 in Ardisiae Japonicae Herba (AJH), 64 in Citri Reticulatae Pericarpium (CRP). All information of the 1456 ingredients is displayed in Supplementary Table S4.

### Oral bioavailability and drug-likeness screening

Oral bioavailability (OB) prescreening, which indicates the fraction of the oral dose of drug that reaches the bloodstream, is one of the important stages of drug discovery and development. In this work, the OB values were calculated by a robust in silico model OBioavail 1.1^31^,^32^. In addition, drug-likeness is a qualitative concept used in drug screening for evaluating the structural similarity between the compounds and the drugs in the DrugBank database, which is estimated at the early stage of drug discovery^11^. The drug-likeness prediction method was shown as follows:


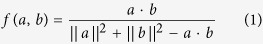


where *a* represents the herbal ingredients, and *b* represents the average molecular drug-likeness index of all drugs in the DrugBank database. Finally, OB ≥ 30% and drug-likeness index ≥0.18 were set as the threshold to select candidate compounds. Additionally, several compounds, such as icariside I, bergenin, tangeretin, naringin, hesperidin, paeonioflorin, and paeonolide initially were omitted according to these screening rules; however, these compounds were supported by experimental evidence and, therefore, also were obtained as candidate compounds for further analysis^13^,^33^,^34^,^35^,^36^,^37^,^38^.

### Target and diseases analysis

Target identification is the critical step in the drug development process. The systematic drug targeting approach is designed to identify potential targets for drugs, natural products, which successfully integrate the chemical, genomic, and pharmacological information for target prediction. In this work, the targets were identified by using the systematic drug targeting approach^39^. Random Forest score ≥0.8 and Support Vector Machine score (SVM) ≥ 0.7 were set as the threshold to select potential targets. Then, the diseases related to the potential targets were extracted from the Therapeutic Target (TTD), DrugBank, and PharmGkb databases, and the diseases were divided further into different groups based on the MeSH Browser (2014 MeSH).

### Network construction

To make a deep understanding of the complex relationships among the compounds, targets, and diseases, the networks were constructed. The herbs, candidate compounds, potential targets, and diseases were applied to construct the compound-target and target-disease networks. The networks were generated and analyzed by using Cytoscape 3.2.1^40^. The degree of a node defines as the number of edges connected to it, implying the importance of the node in a network.

### COPD model and drug administration

COPD rat model was prepared as described in the previous study^41^. Briefly, rats were placed into a chamber connected to a cigarette smoke -producing apparatus (volume 300 L) and exposed to the tobacco smoke and *Klebsiella pneumonia* infection. At the end of week 8, two COPD rats were sacrificed to collect the lung tissues to validate that this rat model was successful.

On week 9, thirty COPD rats were randomly divided into three groups. Then COPD rats were intragastrically treated with normal saline (2 mL), BYF (4.44 g/kg, 0.5 g/ml), and aminophylline (2.3 mg/kg) every day for 12 weeks. The control rats also were administrated intragastrically normal saline (2 mL) for the same amount of time. Finally, all rats were sacrificed, and the heart and lung tissues were collected at week 20. The components of BYF were as follows: GRR 9 g, AR 15 g, CF 12 g, LF 12 g, SCF 9 g, EH 9 g, FTB 9 g, PRR 9 g, Ph 12 g, PF 9 g, AJH 15 g, CRP 9 g. The herbal drugs were identified and prepared in fluid extract. The experiments were conducted in accordance with guidelines of the Committee on the Care and Use of Laboratory Animals of the First Affiliated Hospital, Henan University of Traditional Chinese Medicine, China.

### Respiratory function analyses

Measurement of respiratory function was performed with unrestrained pulmonary function testing plethysmography conducted every fourth week from weeks 0 to 20. Rats were placed inside a closed unrestrained Whole Body Plethysmograph (UWBP, Buxco Electronics, Troy, NY, U.S.A.) connected to a transducer and computer. The respiratory curve was analyzed by the software, and finally tidal volume (VT), peak expiratory flow (PEF) and 50% tidal volume expiratory flow (EF50) were calculated.

### Histological analyses

Paraformaldehyde-fixed heart and lung tissue samples were paraffin-embedded, cut into 4-μm sections and stained with Mayer’s hematoxylin and then with 1% eosin alcohol solution (H&E staining). The morphological changes were examined by using light microscopic.

Alveolar number, alveolar diameter, small pulmonary vessels, and bronchial wall thickness were examined by using Image-Pro Plus® (IPP) 6.0 software (Media Cybernetics, MD, USA). Bronchia, lung injury, and bronchiole stenosis were observed under an optical microscope. The morphometric analysis at the light microscopic level was conducted by an investigator blinded to the study protocol.

For immunohistochemical analysis, the right ventricle and lung tissue paraffin sections (4 μm) were heat fixed, deparaffinized and rehydrated through graded alcohols to distilled water. Then, the sections were subjected to antigen retrieval and treated with 3% hydrogen peroxide to block endogenous peroxidase activity, followed by were incubation with a protein-blocking agent, and then incubated with the primary antibodies (Santa Cruz Biotechnology) against ET-1, TGF-β, VEGF, bFGF, MMP-2, MMP-9, TIMP-1, IL-6, IL-1β, TNF-α, sTNFR2, and collagens I, III, and IV in the presence of 2.5% bovine serum albumin overnight at 4 °C. After that, the sections were incubated with the secondary antibody for 2 hours at room temperature. Immune complexes were visualized using the Catalyzed Signal Amplification System (Dako, Glostrup, Denmark). Samples were inspected and measured using Image-Pro Plus® (IPP) 6.0 software (Media Cybernetics, MD, USA).

### Right Ventricular Hypertrophy Index (RVHI)

After removing the arterial and adipose tissue on the epicardium, the right ventricle (RV), left ventricle (LV), and interventricular septum (S) were separated and weighed. The RVHI was calculated using the equation^42^:


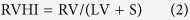


### Myocardial ultrastructure

The right ventricle was fixed with 3% glutaraldehyde in 0.1 mol/L sodium cacodylate buffer (pH 7.4) for 4 h, and then was post-fixed in 1% osmium tetroxide, dehydrated through graded concentrations of ethanol and propylene oxide. The samples were embedded in Epon812 and then sectioned using an ultramicrotome. The sections were stained with uranyl acetate. Finally, myocardial ultrastructural alterations were detected by transmission electron microscopy (TEM).

### Real-time reverse transcriptase polymerase chain reaction (RT-PCR) analysis

Total RNA from lung tissues was extracted using TRIzol solution (Invitrogen; Carlsbad, CA) according to the manufacturer’s protocol. A total of 5 μg RNA was converted to cDNA using a first-strand cDNA synthesis kit (Invitrogen, Carlsbad, CA, USA). TaqMan Master Mix (Biosystems, Foster City, CA, USA) was used to amplify 2 μg cDNA. The expression of *MMP-2 MMP-9* and *TIMP-1* was normalized to *β-actin* levels. The data was analyzed by using the comparative threshold cycle (2^−ΔCT^) method.

### Statistical analysis

Differences between groups were determined by one-way analysis of variance (ANOVA) with the SPSS 19.0 software package (SPSS, Chicago, IL, USA). Values are expressed as means ± SEM. For all tests, a two-sided *P* value less than 0.05 was considered significant.

## Additional Information

**How to cite this article**: Li, J. *et al.* Systems pharmacology-based dissection of mechanisms of Chinese medicinal formula Bufei Yishen as an effective treatment for chronic obstructive pulmonary disease. *Sci. Rep.* **5**, 15290; doi: 10.1038/srep15290 (2015).

## Supplementary Material

Supplementary Table S1, 2, 3, 5

Supplementary Table S4

## Figures and Tables

**Figure 1:**
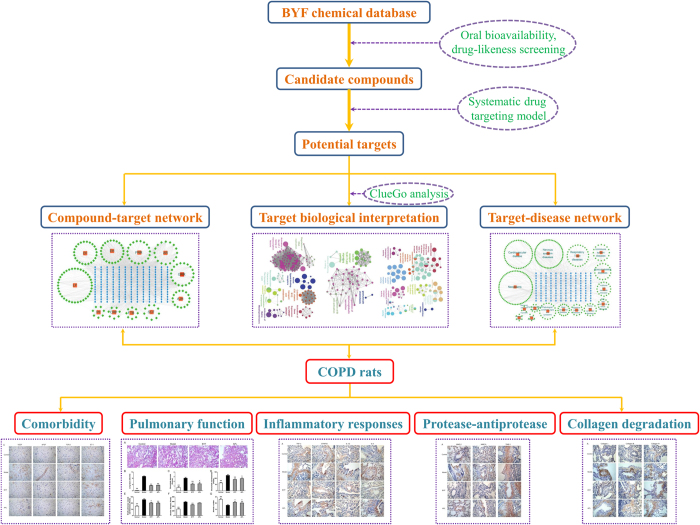
The flowchart of systems pharmacology approach. A systems pharmacology approach was developed to identify the active compounds and therapeutic mechanisms of Bufei Yishen formula (BYF) for the treatment of chronic obstructive pulmonary disease (COPD).
