# Supplementary material for: Systems pharmacology-based dissection of mechanisms of Chinese medicinal formula Bufei Yishen as an effective treatment for chronic obstructive pulmonary disease
Source: Sci Rep. 2015 Oct 15;5:15290. doi: 10.1038/srep15290 (PMC4606809; doi:10.1038/srep15290)
Supplement: Supplementary Table S1, 2, 3, 5 [file srep15290-s1.pdf]

Systems pharmacology-based dissection of mechanisms of Chinese medicinal formula Bufei Yishen as an effective treatment for chronic obstructive pulmonary disease

Jiansheng Li[1, 2, a, *], Peng Zhao[1, 2, a], Ya Li[1, 2], Yange Tian[1, 2], Yonghua Wang[2, 3]

[1] Henan University of Traditional Chinese Medicine, Zhengzhou 450046, China

[2] Collaborative Innovation Center for Respiratory Disease Diagnosis and Treatment & Chinese Medicine Development of Henan Province, Zhengzhou 450046, China

[3] Center of Bioinformatics, Northwest A & F University, Yangling, Shaanxi 712100, China

[a] These authors equally contribute to this paper.

*Corresponding author

E-mail: li_js8@163.com (LJS); Tel.: +86-371-65676568

Table S1 Chemical information of 216 candidate compounds

Herb	MOL_ID	molecule_name	OB	mw	drug-likeness	inchikey	PubChem_Cid
GRR.1	MOL000358	beta-sitosterol	36.91390583	414.790	0.75123	KZJWDPNRJALLNS-VJSFXXLFSA-N	222284
GRR.2	MOL000422	kaempferol	41.88224954	286.250	0.24066	IYRMWMYZSQPJKC-UHFFFAOYSA-N	5280863
GRR.3	MOL000449	Stigmasterol	43.82985158	412.770	0.75665	HCXVJBMSMIARIN-PHZDYDNGSA-N	5280794
GRR.4	MOL000787	Fumarine	59.26250458	353.400	0.82694	GPTFURBXHJWNHR-UHFFFAOYSA-N	Not Available
GRR.5	MOL002879	Diop	43.59332547	390.620	0.39247	IJFPVINAQGWBRJ-UHFFFAOYSA-N	33934
GRR.6	MOL003648	Inermin	65.83093145	284.280	0.53754	HUKSJTUUSUGIDC-BDJLRTHQSA-N	Not Available
GRR.7	MOL004492	Chrysanthemaxanthin	38.72398115	584.960	0.58352	JRHJXXLCNATYLS-HYHFCDFPSA-N	Not Available
GRR.8	MOL005308	Aposiopolamine	66.64690713	271.340	0.21999	UPWMWFSEBOFTNA-SBAKFKMJSA-N	Not Available
GRR.9	MOL005314	Celabenzine	101.8825954	379.550	0.48772	LSYKFBZWBDMZLQ-OAQYLSRUSA-N	Not Available
GRR.10	MOL005317	Deoxyharringtonine	39.27443988	515.660	0.8116	WRCBXHDQHPUVHW-CDRKEARJSA-N	Not Available
GRR.11	MOL005318	Dianthramine	40.44641187	289.260	0.19676	SVZLTRRSGWXPBL-UHFFFAOYSA-N	441562
GRR.12	MOL005320	arachidonate	45.57324991	304.520	0.20491	YZXBAPSDXZZRGB-CGRWFSSPSA-N	5312542
GRR.13	MOL005321	Frutinone A	65.9037307	264.240	0.34184	RFWULRHBGYKEEZ-UHFFFAOYSA-N	441965
GRR.14	MOL005344	ginsenoside rh2	36.31951162	622.980	0.55868	CKUVNOCSB	119307

GRR.15	MOL005348	Ginsenoside-Rh 4_qt	31.11214828	458.800	0.77829	YYHIS-IRFFN ABBSA-N JKPOYAJYRY OGBN-ARWCP IPWSA-N	Not Available
GRR.16	MOL005356	Girinimbin	61.21530251	263.360	0.31484	GAEQWKVG MHUUKO-UH FFFAOYSA-N	96943
GRR.17	MOL005357	Gomisin B	31.99042428	514.620	0.82858	XDVOVYYAP HHHBE-YGIM CSHQSA-N	Not Available
GRR.18	MOL005360	malkangunin	57.71384384	432.560	0.62642	DTMIMKTZET WDJV-DUUKB JRLSA-N	Not Available
GRR.19	MOL005376	Panaxadiol	33.0879606	460.820	0.79404	PVLHOJXLNB FHDX-BMTJS WHCSA-N	Not Available
GRR.20	MOL005384	suchilactone	57.51882425	368.410	0.55573	GVNUFBXIXQ NOCF-CRNMQ VKPSA-N	Not Available
GRR.21	MOL005399	alexandrin_qt	36.91390583	414.790	0.75268	KZJWDPNRJA LLNS-HEEOY UHISA-N	Not Available
GRR.22	MOL005401	ginsenoside Rg5_qt	39.56307142	442.800	0.78506	FCMJTYFZRU KOPU-FFFKM GBISA-N	Not Available
PR.1	MOL000033	(3S,8S,9S,10R,13R,14S,17R)-10,13-dimethyl-17-[(2R,5S)-5-propan-2-yloctan-2-yl]-2,3,4,7,8,9,11,12,14,15,16,17-dodecahydro-1H-cyclopenta[a]phenanthren-3-ol	36.22847056	428.820	0.78288	KLEXDBGYS OIREE-UIFQY PGESA-N	15976101
PR.2	MOL000098	quercetin	46.43334812	302.250	0.27525	REFJWTPEDV JJIY-UHFFFAO YSA-N	5280343
PR.3	MOL000211	Mairin	55.37707338	456.780	0.7761	QGJZLNKBHJ ESQX-FZFNOL FKSA-N	64971

PR.4	MOL000239	Jaranol	50.82881677	314.310	0.29148	BJBUTJQYZDYRMJ-UHFFFAOYSA-N	5318869
PR.5	MOL000296	hederagenin	36.91390583	414.790	0.75072	KZJWDPNRJALLNS-CQXWNKEUSA-N	Not Available
PR.6	MOL000354	isorhamnetin	49.60437705	316.280	0.306	IZQSVPBOUDKVDZ-UHFFFAOYSA-N	5281654
PR.7	MOL000371	3,9-di-O-methylnissolin	53.74152673	314.360	0.47573	RFFNFQZKHNKOPO-BBRMVZONSA-N	15689655
PR.8	MOL000374	5'-hydroxyiso-muronulatol-2',5'-di-O-glucoside	41.71766574	642.670	0.69251	SRVGYVIWVOOXQO-FQRJZKGRSA-N	Not Available
PR.9	MOL000378	7-O-methylisomucronulatol	74.68613752	316.380	0.29792	BLHQCBJSTMDZQA-LBPRGKRZSA-N	15689652
PR.10	MOL000379	9,10-dimethoxypterocarpan-3-O-β-D-glucoside	36.73668801	462.490	0.9243	PCIXSTFFMHVOMF-PBGSHFJYSA-N	Not Available
PR.11	MOL000380	(6aR,11aR)-9,10-dimethoxy-6a,11a-dihydro-6H-benzofurano[3,2-c]chromen-3-ol	64.25545452	300.330	0.42486	UOVGCLXUTLXAEC-WFASDCNBSA-N	14077830
PR.12	MOL000387	Bifendate	31.09782391	418.380	0.66553	JMZOMFYRADAWOG-UHFFFAOYSA-N	108213
PR.13	MOL000392	formononetin	69.67388061	268.280	0.21202	HKQYGTCOTHHOMP-UHFFFAOYSA-N	5280378
PR.14	MOL000398	isoflavanone	109.9866565	316.330	0.29572	JNSVNRWHSLLCBG-LLVKDONJSA-N	Not Available
PR.15	MOL000417	Calycosin	47.75182783	284.280	0.24278	ZZAJQOPSWWVMBI-UHFFFAOYSA-N	5280448
PR.16	MOL000422	kaempferol	41.88224954	286.250	0.24066	IYRMWMYZSQPJKC-UHFFFAOYSA-N	5280863
PR.17	MOL000433	FA	68.96043622	441.450	0.7057	OVBPIULPVIDEAO-LBPRGK	6037

						RZSA-N	
PR.18	MOL000438	(3R)-3-(2-hydroxy-3,4-dimethoxyphenyl)chroman-7-ol	67.66747949	302.350	0.26479	NQRBAPDEZYMKFL-NSHDSACASA-N	10380176
PR.19	MOL000439	isomucronulatol-7,2'-di-O-glucosiole	49.28105539	626.670	0.62065	NHOPAJCVMDIGBN-MEPKZADGSA-N	15689653
PR.20	MOL000442	1,7-Dihydroxy-3,9-dimethoxy pterocarpene	39.04541112	314.310	0.47943	RVGZSUMTFIEORY-UHFFFAOYSA-N	5316760
LF.1	MOL000098	quercetin	46.43334812	302.250	0.27525	REFJWTPEDVJJIY-UHFFFAOYSA-N	5280343
LF.2	MOL000358	beta-sitosterol	36.91390583	414.790	0.75123	KZJWDPNRJALLNS-VJSFXXLFSA-N	222284
LF.3	MOL000449	Stigmasterol	43.82985158	412.770	0.75665	HCXVJBMSMIARIN-PHZDYDNGSA-N	5280794
LF.4	MOL000953	CLR	37.87389754	386.730	0.67677	HVYWMOMLDIMFJA-DPAQBDIFSA-N	5997
LF.5	MOL001323	Sitosterol alpha1	43.28127042	426.800	0.78354	LPZCCMIISIBREI-JXMPMKKESA-N	9548595
LF.6	MOL001494	Mandenol	41.99620045	308.560	0.19321	FMMOOAYVCKXGMF-MURFETPASA-N	5282184
LF.7	MOL001495	Ethyl linolenate	46.10096327	306.540	0.19716	JYYFMIOPGOFNPK-XSHSMGBESA-N	6371716
LF.8	MOL001979	LAN	42.11918897	426.800	0.74787	CAHGCLMLTWQZNJ-BQNIITSRSA-N	246983
LF.9	MOL003578	Cycloartenol	38.68565906	426.800	0.78093	ONQRKEUAIJMULO-YBXTVTTCSA-N	Not Available
LF.10	MOL005406	atropine	45.97058178	289.410	0.19328	RKUNBYITZUJHSG-QKPAOTATSA-N	154417
LF.11	MOL005438	campesterol	37.57681789	400.760	0.71488	SGNBVLSWZMBQTH-KAAS	Not Available

						KNFBSA-N	
LF.12	MOL006209	cyanin	47.42092269	411.660	0.75918	ZNWVDIROEWRDJT-DARPEHSRSA-N	Not Available
LF.13	MOL007449	24-methylidenelophenol	44.19264545	412.770	0.7533	RSMKYRDCCSNYFM-AAGDOFLISA-N	5283640
LF.14	MOL008173	daucosterol_qt	36.91390583	414.790	0.75316	KZJWDPNRJALLNS-ZBNQVYKFSA-N	Not Available
LF.15	MOL008400	glycitein	50.47891366	284.280	0.23826	DXYUAIFZCFRPTH-UHFFFAOYSA-N	5317750
LF.16	MOL009604	14b-pregnane	34.77923299	288.570	0.33723	JWMFYGXQPXQEEM-MTUNSFAGSA-N	Not Available
LF.17	MOL009612	(24R)-4alpha-Methyl-24-ethylcholesta-7,25-dien-3beta-ylacetate	46.35749925	482.870	0.8398	YXSNMSCMEXMDCO-ZDOZIUNCSA-N	Not Available
LF.18	MOL009615	24-Methylenecycloartan-3beta,21-diol	37.31728162	456.830	0.79751	FJXNINQGUTYPNE-DLJSKDEVSA-N	Not Available
LF.19	MOL009617	24-ethylcholest-22-enol	37.09454086	414.790	0.7511	CSVWWLUMXNHWSU-YASJBQHGSA-N	Not Available
LF.20	MOL009618	24-ethylcholesta-5,22-dienol	43.82985158	412.770	0.75636	HCXVJBMSMIARIN-KRUSZOTOSA-N	Not Available
LF.21	MOL009620	24-methyl-31-norlanost-9(11)-enol	37.9996853	428.820	0.75092	NWPXYRKVJLBSQU-NTJPQSOTSA-N	Not Available
LF.22	MOL009621	24-methylenelanost-8-enol	42.36819868	440.830	0.76769	XJLZCPIILZRCPS-MCOFMKOSSA-N	Not Available
LF.23	MOL009622	Fucosterol	43.77639556	412.770	0.75668	OSELKOCHBMDKEJ-JUGJNGJRSA-N	5281328
LF.24	MOL009631	31-Norcyclolaudenol	38.68209614	440.830	0.81391	OSKBBWPZDJRQMS-TVCYAWOFSA-N	Not Available
LF.25	MOL009633	31-norlanost-9(11)-enol	38.35394137	414.790	0.7249	SZCKXGWHINUNKB-SNKUZ	Not Available

						YNWSA-N	
LF.26	MOL009634	31-norlanosterol	42.20462055	412.770	0.73012	KLZWTHGLLDRKHD-MGAQDEEHSA-N	Not Available
LF.27	MOL009635	4,24-methyllophenol	37.83467433	414.790	0.74999	AOQRDALGACAKHI-AHSCMWHKSA-N	Not Available
LF.28	MOL009639	Lophenol	38.12940252	400.760	0.714	LMYZQUNLYGJIHI-SPONXPENSA-N	160482
LF.29	MOL009640	4alpha,14alpha,24-trimethylcholesta-8,24-dienol	38.90988973	426.800	0.75772	GCZFZGBLBXBCJP-VXRRTDEQSA-N	Not Available
LF.30	MOL009641	4alpha,24-dimethylcholesta-7,24-dienol	42.65304098	412.770	0.75297	KPIRFXVTLHBVFL-AAGDOFLISA-N	Not Available
LF.31	MOL009642	4alpha-methyl-24-ethylcholesta-7,24-dienol	42.29509453	426.800	0.78304	HIIFUGOBGVCVLO-MTFRKTCUSA-N	Not Available
LF.32	MOL009644	6-Fluoroindole-7-Dehydrocholesterol	43.72602513	402.700	0.72224	HMEVHMIHKDZWLI-WXBCRLQESA-N	Not Available
LF.33	MOL009646	7-O-Methylluteolin-6-C-beta-glucoside_qt	40.77368843	318.300	0.30497	PHGRMBKBKPAXKQ-LLVKDONJSA-N	Not Available
LF.34	MOL009650	Atropine	42.15897078	289.410	0.19299	RKUNBYITZUJHSG-JJXSEGSLSA-N	637577
LF.35	MOL009651	Cryptoxanthin monoepoxide	46.95371937	568.960	0.56103	CMOLUFWHADIFGS-WZIUPQIASA-N	Not Available
LF.36	MOL009653	Cycloeucalenol	39.72647216	426.800	0.79446	HUNLTIZKNQDZEI-PGFZVWMDSA-N	Not Available
LF.37	MOL009656	(E,E)-1-ethyl octadeca-3,13-dienoate	41.99620045	308.560	0.19364	WDXJYOKJLIZASF-RSIFQWQVSA-N	Not Available
LF.38	MOL009660	methyl (1R,4aS,7R,7aS)-4a,7-dihydroxy-7-methyl-1-[(2S,3R,4S,5S,6R)-3,4,5-trihydroxy	39.42847682	406.430	0.46558	RWMXKBUPLSNIJL-VALIWVFLSA-N	11968619

		-6-(hydroxymethyl)oxan-2-yl]oxy-1,5,6,7a-tetrahydrocyclopenta[d]pyran-4-carboxylate					
LF.39	MOL009662	Lantadene A	38.67942417	552.870	0.57405	KCLIRHUTOPOHKJ-DMKBDCOSSA-N	Not Available
LF.40	MOL009664	Physalin A	91.70647491	526.580	0.27207	VELDODQHYQSJOF-RLGRCWQRSA-N	Not Available
LF.41	MOL009665	Physcion-8-O-beta-D-gentiobioside	43.90358656	608.600	0.62426	LHWONDXFTUKXDH-BFTLVBKUSA-N	5320543
LF.42	MOL009677	lanost-8-en-3beta-ol	34.22630373	428.820	0.74036	MBZYKEVPFYHDOH-BQNIITSRSA-N	440560
LF.43	MOL009678	lanost-8-enol	34.22630373	428.820	0.74167	MBZYKEVPFYHDOH-ZSLNGYDASA-N	Not Available
LF.44	MOL009681	Obtusifoliol	42.55200222	426.800	0.7565	MMNYKQIDRZNIKT-VSADUBDNSA-N	65252
LF.45	MOL010234	delta-Carotene	31.80094312	536.960	0.54639	WGIYGODPCLMGQH-GOXCNPTKSA-N	5281230
SCF.1	MOL004624	Longikaurin A	47.72214984	348.480	0.53015	PSVHVXLCVSKJGM-MHRDNBEJSA-N	Not Available
SCF.2	MOL005317	Deoxyharringtonine	39.27443988	515.660	0.8116	WRCBXHDQHPUVHW-CDRKEARJSA-N	Not Available
SCF.3	MOL008956	Angeloylgomisin O	31.96538945	498.620	0.84763	PLKFSXFJGNZAER-VDJLVHAZSA-N	Not Available
SCF.4	MOL008957	Schizandrer B	30.70577053	514.620	0.82854	BKGUPIVDQHHVMV-TWJXSMCESA-N	Not Available
SCF.5	MOL008968	Gomisin-A	30.69375343	416.510	0.77723	ZWRRJEICIPUPHZ-MYODQAERSA-N	Not Available
SCF.6	MOL008974	Gomisin G	32.67834097	508.610	0.82713	AZMQTBXLM	Not Available

						HEDNG-JCNF ZPKBSA-N	
SCF.7	MOL008978	Gomisin R	34.84255546	400.460	0.85805	HOPDFAWBF XSPSA-FHGN ATFXSA-N	Not Available
SCF.8	MOL003546	Aristolone	45.30748966	218.37	0.13009	UGVIZCBJCS XBCJ-JWFUO XDNSA-N	165536
SCF.9	MOL003541	()-alpha-Longipi nene	57.47116489	204.39	0.12474	HICYDYJTCD BHMZ-UKTAR XLSSA-N	Not Available
SCF.10	MOL008992	Wuweizisu C	46.26685721	384.460	0.8436	HTBWBWWA DZJXID-TXEJJ XNPSA-N	Not Available
EH.1	MOL000006	luteolin	36.16262934	286.250	0.24552	IQPNAANSBP BGFQ-UHFFF AOYSA-N	5280445
EH.2	MOL000098	quercetin	46.43334812	302.250	0.27525	REFJWTPEDV JJIY-UHFFFAO YSA-N	5280343
EH.3	MOL000359	sitosterol	36.91390583	414.790	0.7512	KZJWDPNRJA LLNS-ZFVHJZ ABSA-N	12303645
EH.4	MOL000422	kaempferol	41.88224954	286.250	0.24066	IYRMWMYZS QPJKC-UHFFF AOYSA-N	5280863
EH.5	MOL000622	Magnograndioli de	63.70888436	266.370	0.18833	VHFXPBHLQO PQHJ-ABBQY LIMSA-N	5319198
EH.6	MOL001510	24-epicampester ol	37.57681789	400.760	0.71413	SGNBVLSWZ MBQTH-ZRUU VFCLSA-N	5283637
EH.7	MOL001645	Linoleyl acetate	42.10076623	308.560	0.19845	KFXARGMQY WECBV-ZDVG BALWSA-N	5319042
EH.8	MOL001771	poriferast-5-en-3beta-ol	36.91390583	414.790	0.75034	KZJWDPNRJA LLNS-FBZNIE FRSA-N	457801
EH.9	MOL001792	DFV	32.76272375	256.270	0.18316	FURUXTVZLH CCNA-AWEZN QCLSA-N	114829
EH.10	MOL003044	Chryseriol	35.85089483	300.280	0.27415	SCZVLDHREV KTSH-UHFFF	5280666

						AOYSA-N	
EH.11	MOL003542	8-Isopentenyl-kaempferol	38.04433524	354.380	0.3948	NADCVNHITZNGJU-UHFFFAOYSA-N	5318624
EH.12	MOL004367	olivil	62.22859563	376.440	0.40642	BVHIKUCXNBQDEM-XMCHAPAWSA-N	Not Available
EH.13	MOL004373	Anhydroicaritin	45.41193421	368.410	0.43786	TUUXBSASAQJECY-UHFFFAOYSA-N	5318980
EH.14	MOL004380	C-Homoerythrinan, 1,6-didehydro-3,15,16-trimethoxy-, (3.beta.)-	39.13992598	329.480	0.49461	VFNBFPRWBICVGZ-JXFKEZNVSA-N	Not Available
EH.15	MOL004382	Yinyanghuo A	56.9573795	420.490	0.76747	ZAUWPDSVLSOCDG-SFHVURJKSA-N	Not Available
EH.16	MOL004384	Yinyanghuo C	45.67199685	336.360	0.50155	GPXYBBZISZKRAH-UHFFFAOYSA-N	5315395
EH.17	MOL004386	Yinyanghuo E	51.63212506	352.360	0.5474	FIKLOAGOJKGOFT-UHFFFAOYSA-N	5315397
EH.18	MOL004388	6-hydroxy-11,12-dimethoxy-2,2-dimethyl-1,8-dioxo-2,3,4,8-tetrahydro-1H-isochromeno[3,4-h]isoquinolin-2-ium	60.64150904	370.410	0.65693	FUBYUUKASUJMSZ-UHFFFAOYSA-N	12115137
EH.19	MOL004391	8-(3-methylbut-2-enyl)-2-phenyl-chromone	48.54449639	290.380	0.25066	FMPOBQILEGSRSJ-UHFFFAOYSA-N	17861868
EH.20	MOL004394	Anhydroicaritin-3-O-alpha-L-rhamnoside	41.5834004	676.730	0.60981	TZJALUIVHRYQQB-YPRONELTSA-N	Not Available
EH.21	MOL004396	1,2-bis(4-hydroxy-3-methoxyphenyl)propan-1,3-diol	52.31424958	320.370	0.22066	DFUOJBWSSSODTR-SJCJKPOMSA-N	12468616
EH.22	MOL004425	Icariin	41.5834004	676.730	0.61051	TZJALUIVHRYQQB-XLRX	5318997

EH.23	MOL004385	Yinyanghuo D	13.9852424578	338.380	0.37995	WWTNSA-NPFQMUQWFRINBBG-UHFFFAOYSA-N	5315396
EH.24	MOL004393	anhydroicaritin	28.2735277666	368.410	0.59309	PPCHTBBOSVKORE-UHFFFAOYSA-N	14583584
EH.25	MOL004422	Hexandraside E	13.5562634422	678.700	0.596	SLUGZPRLJCECEX-YVBMKHBZSA-N	Not Available
EH.26	MOL004430	icariside I	21.8791275439	530.570	0.84822	IYCPMVXIUPYNHI-RBOMYRCTSA-N	Not Available
EH.27	MOL004427	Icariside A7	31.90509191	462.490	0.85568	HNMHZSRVQJZGPQ-PUIBNRJISA-N	5318401
FTB.1	MOL000358	beta-sitosterol	36.91390583	414.790	0.75123	KZJWDPNRJALLNS-VJSFXXLFSA-N	222284
FTB.2	MOL001004	pelargonidin	37.98831233	271.260	0.21204	SAURRTSFHXYOSN-UHFFFAOYSA-N	Not Available
FTB.3	MOL004440	Peimisine	57.4023933	427.690	0.8055	KYELXPJVGNZIGC-GKFGJCLESA-N	161294
FTB.4	MOL004443	Zhebeiresinol	58.72053449	280.300	0.19384	AUXYOVQIZNPKSO-KKFJDGPESA-N	Not Available
FTB.5	MOL004444	Ziebeimine	64.24657792	413.710	0.70486	OEJGVNMSFPGDPP-BDFZTFKFSA-N	Not Available
FTB.6	MOL004446	6-Methoxyl-2-acetyl-3-methyl-1,4-naphthoquinone-8-O-beta-D-glucopyranoside	33.30734381	422.420	0.57257	GVMOOQOCLYWVKF-BNCZOOBYSA-N	5319462
FTB.7	MOL004447	6-Methoxyl-2-acetyl-3-methyl-1,4-naphthoquinone-8-O-beta-D-glucopyranoside_qt	19.87055512	260.26	0.15427	GBEJSKTVOVIDMP-UHFFFAOYSA-N	67877406
FTB.8	MOL004453	Peiminoside_qt	11.75276358	431.73	0.66821	IUKLSMSEHK	Not Available

						DIIP-FWSIRKJPSA-N	
FTB.9	MOL004450	Chaksine	65.63417036	450.660	0.66463	CGGAHJGHSHWGLE-WJQMWINMSA-N	120699
PF.1	MOL000006	luteolin	36.16262934	286.250	0.24552	IQPNAANSBPBGFQ-UHFFFAOYSA-N	5280445
PF.2	MOL000358	beta-sitosterol	36.91390583	414.790	0.75123	KZJWDPNRJALLNS-VJSFXXLFSA-N	222284
PF.3	MOL000449	Stigmasterol	43.82985158	412.770	0.75665	HCXVJBMSMIARIN-PHZDYDNGSA-N	5280794
PF.4	MOL000953	CLR	37.87389754	386.730	0.67677	HVYWMOMLDIMFJA-DPAQBDIFSA-N	5997
PF.5	MOL001439	arachidonic acid	45.57324991	304.520	0.20409	YZXBAPSDXZZRGB-DOFZRALJSA-N	444899
PF.6	MOL002773	beta-carotene	37.18433337	536.960	0.58358	OENHQHLEOONYIE-JLTXGRSLSA-N	5280489
PF.7	MOL004355	Spinasterol	42.97936552	412.770	0.75534	JZVFJDZBLUFKCA-FXIAWGAOSA-N	5281331
PF.8	MOL005030	gondoic acid	30.70294255	310.580	0.19743	BITHHVVYSMSWAG-KTKRTIGZSA-N	5282768
PF.9	MOL005043	campest-5-en-3beta-ol	37.57681789	400.760	0.71481	SGNBVLSWZMBQTH-PODYLUTMSA-N	173183
PF.10	MOL005481	2,6,10,14,18-pentamethylicosa-2,6,10,14,18-pentaene	33.4041173	342.670	0.24028	IMXDCJPVYKXJPD-FMOJUEAUSA-N	5366013
PF.11	MOL007449	24-methylidenelophenol	44.19264545	412.770	0.7533	RSMKYRDCCSNYFM-AAGDOFLISA-N	5283640
PF.12	MOL009653	Cycloeucalenol	39.72647216	426.800	0.79446	HUNLTIZKNQDZEI-PGFZVWMDSA-N	Not Available
PF.13	MOL009681	Obtusifoliol	42.55200222	426.800	0.7565	MMNYKQIDR	65252

						ZNIKT-VSADUBDNSA-N	
PF.14	MOL012888	citrostadienol	43.28127042	426.800	0.78568	LPZCCMIISIBREI-ZXBKQEFASA-N	Not Available
PF.15	MOL012891	(2E,4E,6E)-icosa-2,4,6-trienoic acid	41.64184852	306.540	0.20213	BBWMTEYXFFWPIF-CJBMEHDJSA-N	6506063
PF.16	MOL012893	(E)-(4-methylbenzylidene)-(4-phenyltriazol-1-yl)amine	57.87220103	262.340	0.18812	HGKZMFCXZIIEEU-GZTJUZNOSA-N	9602469
AJH.1	MOL000098	quercetin	46.43334812	302.250	0.27525	REFJWTPEDVJJIY-UHFFFAOYSA-N	5280343
AJH.2	MOL000422	kaempferol	41.88224954	286.250	0.24066	IYRMWMYZSQPJKC-UHFFFAOYSA-N	5280863
AJH.3	MOL001663	(4aS,6aR,6aS,6bR,8aR,10R,12aR,14bS)-10-hydroxy-2,2,6a,6b,9,9,12a-heptamethyl-1,3,4,5,6,6a,7,8,8a,10,11,12,13,14b-tetradecahydropicene-4a-carboxylic acid	32.02801329	456.780	0.75713	MIJYXULNPSFWEK-KDQGZELNSA-N	11869658
AJH.4	MOL002879	Diop	43.59332547	390.620	0.39247	IJFPVINAQGWBRJ-UHFFFAOYSA-N	33934
AJH.5	MOL009278	Laricitrin	35.38099156	332.280	0.34199	CFYMYCCYMJIYAB-UHFFFAOYSA-N	5282154
AJH.6	MOL010934	ardisianoside K_qt	31.97790441	472.780	0.62575	DLWHFHBRYSITBI-WGIDJJHTSA-N	Not Available
AJH.7	MOL010953	triterpenoid glycoside 1_qt	34.11143581	472.780	0.63118	UBWMMEPLQFWYCH-GZAMYCOFSA-N	Not Available
AJH.8	MOL010964	MAESANIN	42.77499821	362.560	0.35103	WVHQJXPRVZBEFP-SREVYHEPSA-N	5384838

AJH.9	MOL010973	Rapanone	34.1530898	322.490	0.23812	AMKNOBHCKRZHIO-UHFFFAOYSA-N	100659
AJH.10	MOL010974	tri-O-methylnorbergenin	33.17034587	356.360	0.40979	RGHGUQJYNLPWPT-GVMTXOEMSA-N	44584185
AJH.11	MOL010976	triterpene glycoside 4_qt	41.39996748	472.780	0.63122	UBWMMEPLQFWYCH-RMXBMUOASA-N	Not Available
AJH.12	MOL010981	triterpenoid glycoside 3_qt	44.03723066	488.780	0.6009	WTFUHAGPAIREPL-RYZHOHRSSA-N	Not Available
AJH.13	MOL010982	2,5-dihydroxy-3-[(10Z)-pentadec-10-en-1-yl][1,4]benzoquinone	34.73921496	460.770	0.60316	OZOJZESTKHHHQP-WAYWQWQTSA-N	Not Available
AJH.14	MOL010983	2,5-Dihydroxy-3-[(10Z)-pentadec-10-en-1-yl]cyclohexa-2,5-diene-1,4-dione	37.3018181	348.530	0.32034	YRIWERDENGDRIR-WAYWQWQTSA-N	Not Available
AJH.15	MOL010985	2-hydroxy-5-methoxy-3-pentadecaenylbenzoquinone	41.61117697	362.560	0.3151	GSFLASJBASRJKZ-HYPNTESJSA-N	Not Available
AJH.16	MOL011002	5-ethoxy-2-hydroxy-3-[(10Z)-pentadec-10-en-1-yl][1,4]benzoquinone	42.77105866	376.590	0.38323	YQDOWDWMMZKLQR-FPLPWBNLSA-N	Not Available
AJH.17	MOL011003	5-ethoxy-2-hydroxy-3-[(8Z)-tridec-8-en-1-yl][1,4]benzoquinone	43.22748811	348.530	0.2961	XELCDMYKAGKICS-FPLPWBNLSA-N	Not Available
AJH.18	MOL011019	ardisianone A	44.22012847	346.560	0.24889	WNZXOLJKBYUFDZ-ZFYPLVIYSA-N	Not Available
AJH.19	MOL002816	Bergenin	14.1130213978	328.300	0.34195	YWJXCIXBAKGUKZ-HJJNZUOJSA-N	66065
AJH.20	MOL011020	ardisianone B	60.90178195	334.500	0.19783	TYEYBRGUMMKRGI-ZHANPKHBSA-N	Not Available

CRP.1	MOL000001	anthocyanidin	45.59739165	251.220	0.18968	YLJQHJNWIHUOKW-UHFFFAOYSA-N	Not Available
CRP.2	MOL000359	sitosterol	36.91390583	414.790	0.7512	KZJWDPNRJALLNS-ZFVHJZABSA-N	12303645
CRP.3	MOL004328	naringenin	59.29389773	272.270	0.21128	FTVWIRXFELQLPI-ZDUSSCGKSA-N	439246
CRP.4	MOL005100	5,7-dihydroxy-2-(3-hydroxy-4-methoxyphenyl)chroman-4-one	47.73643694	302.300	0.27226	AIONOLUJZLIMTK-CQSZACIVSA-N	676152
CRP.5	MOL005815	Citromitin	86.90404672	404.450	0.51439	LTRBUBSPQISFFL-CQSZACIVSA-N	Not Available
CRP.6	MOL007930	hesperidin	13.33460023	610.62	0.66749	QUQPHWDTPGMPEX-QJBIFVCTSA-N	10621
CRP.7	MOL005814	tangeretin	21.3751908	372.4	0.42958	ULSUXBXHSYSGDT-UHFFFAOYSA-N	68077
CRP.8	MOL005828	nobiletin	61.66943932	402.430	0.51652	MRIAQLRQZPPODS-UHFFFAOYSA-N	72344
CF.1	MOL000358	beta-sitosterol	36.91390583	414.790	0.75123	KZJWDPNRJALLNS-VJSFXXLFSA-N	222284
CF.2	MOL000359	sitosterol	36.91390583	414.790	0.7512	KZJWDPNRJALLNS-ZFVHJZABSA-N	12303645
CF.3	MOL000449	Stigmasterol	43.82985158	412.770	0.75665	HCXVJBMSMIARIN-PHZDYDNGSA-N	5280794
CF.4	MOL000554	gallic acid-3-O-(6'-O-galloyl)-glucoside	30.25032187	484.400	0.6746	NRQUZRZEYPSZEY-IDXPAVDQSA-N	Not Available
CF.5	MOL001494	Mandenol	41.99620045	308.560	0.19321	FMMOOAYVCKXGMF-MURFETPASA-N	5282184
CF.6	MOL001495	Ethyl linolenate	46.10096327	306.540	0.19716	JYYFMIOPGOFNPK-XSHSM	6371716

						GBESA-N	
CF.7	MOL001771	poriferast-5-en-3beta-ol	36.91390583	414.790	0.75034	KZJWDPNRJALLNS-FBZNIEFRSA-N	457801
CF.8	MOL002879	Diop	43.59332547	390.620	0.39247	IJFPVINAQGWBRJ-UHFFFAOYSA-N	33934
CF.9	MOL002883	Ethyl oleate (NF)	32.39738821	310.580	0.19061	LVGKNOAMLMIIKO-QXMHVHEDSA-N	5363269
CF.10	MOL003137	Leucanthoside	32.11589283	462.440	0.78146	DLVLXOYLQKCAME-DGHBBABESA-N	442659
CF.11	MOL005360	malkangunin	57.71384384	432.560	0.62642	DTMIMKTZETWDJV-DUUKBJRLSA-N	Not Available
CF.12	MOL005481	2,6,10,14,18-pentamethylicosa-2,6,10,14,18-pentaene	33.4041173	342.670	0.24028	IMXDCJPVYKXJPD-FMOJUEAUSA-N	5366013
CF.13	MOL005486	3,4-Dehydrolycopen-16-al	46.64445252	548.920	0.4906	FPLASDSFNINBIY-QETLVAEPSA-N	5316458
CF.14	MOL005489	3,6-Digalloylglucose	31.41521237	484.400	0.66343	LRSHPKZSGRNHIX-MPWSSXMOSA-N	Not Available
CF.15	MOL005503	Cornudentanone	39.6634055	378.560	0.327	JIUGZSYPFREDLG-HXUWFJFHSA-N	46191017
CF.16	MOL005530	Hydroxygenkwanin	36.46699689	300.280	0.27206	RRRSSAVLTCVNIQ-UHFFFAOYSA-N	5318214
CF.17	MOL005531	Telocinobufagin	69.99386894	402.580	0.79297	PBSOJKPTQWWJJD-XECVOWJVSA-N	Not Available
CF.18	MOL005552	gemin D	68.8303535	634.490	0.56075	XKVYZLLWKHGKMT-UPMOLNEASA-N	Not Available
CF.19	MOL005557	lanosta-8,24-dien-3-ol,3-acetate	44.29553995	468.840	0.82425	BQPPJGMMIYJVBR-MJGQBHONSA-N	Not Available
CF.20	MOL005544	Cornin	12.68527831	388.410	0.43901	HLXRWTJXGMHOFN-XJSN	73467

						KYLASA-N	
CF.21	MOL005545	cornin_qt	25.1044089	226.250	0.097726	ICLHTGIHDLYEDX-PPZZJSARSA-N	12444745
CF.22	MOL005546	cornuside	2.610111139	542.540	0.70539	SMTKSCGLXONVGL-MUCSSEFLSA-N	Not Available
CF.23	MOL005547	cornuside_qt	2.372602589	380.380	0.39489	KDGCSTFYBWGBNX-PJYBLOJUSA-N	Not Available
CF.24	MOL005467	Epicatechin gallate	17.88586712	442.400	0.74627	LSHVYAFMTMFKBA-FPOVZHCZSA-N	65056
CF.25	MOL003166	Swertiamarin	21.90382697	374.380	0.42213	HEYZWPRKKUGDCR-QBXMEVCASA-N	442435
CF.26	MOL002343	tetrandrine	26.63974111	622.820	0.10166	WVTKBKWTSCPRNU-KYJUHHDHSA-N	Not Available
CF.27	MOL001996	Betulonic acid	16.83371616	454.760	0.77936	SLJTWDNVZKIDAU-SVAFSPIFSA-N	122844
CF.28	MOL001680	Loganin	5.901127525	390.430	0.43563	AMBQHHVBBHTQBF-UOUCRYGSSA-N	87691
CF.29	MOL000890	(+)-alpha-Curcumene	26.56378766	202.370	0.055919	VMYXUZSZMNBRCN-AWEZNQCLSA-N	3083834
CF.30	MOL000431	coumarin	29.16755329	146.150	0.043014	ZYGHJZDHTFUPRJ-UHFFFAOYSA-N	323
CF.31	MOL000027	alpha-Curcumene	4.677265254	202.370	0.055937	VMYXUZSZMNBRCN-CQSZACIVSA-N	442360
CF.32	MOL000105	protocatechuic acid	25.36646796	154.130	0.035092	YQUVCSBJEUQKSH-UHFFFAOYSA-N	72
CF.33	MOL000223	caffeic acid	25.76439804	180.170	0.050089	QAIPRVGONGVQAS-DUXPYHPUSA-N	689043
CF.34	MOL000263	oleanolic acid	29.02084142	456.780	0.75599	MIJYXULNPSFWEK-GTOFXWBISA-N	10494

CF.35	MOL008457	Tetrahydroalstonine	32.41977527	352.470	0.81311	GRTOGORTSDXSFK-DLLGKBFGSA-N	Not Available
PRR.1	MOL000358	beta-sitosterol	36.91390583	414.790	0.75123	KZJWDPNRJALLNS-VJSFXXLFSA-N	222284
PRR.2	MOL000359	sitosterol	36.91390583	414.790	0.7512	KZJWDPNRJALLNS-ZFVHJZABSA-N	12303645
PRR.3	MOL000449	Stigmasterol	43.82985158	412.770	0.75665	HCXVJBMSMIARIN-PHZDYDNGSA-N	5280794
PRR.4	MOL000492	(+)-catechin	54.82643405	290.290	0.24164	PFTAWBLQPZVEMU-DZGCQCFKSA-N	9064
PRR.5	MOL001002	ellagic acid	43.06455858	302.200	0.43417	AFSDNFLWKVMVRB-UHFFFAOYSA-N	Not Available
PRR.6	MOL001918	paeoniflorgenone	87.59312084	318.350	0.36678	BANPEMKDTXIFRE-GHVWTTSJSA-N	Not Available
PRR.7	MOL001921	Lactiflorin	49.12131675	462.490	0.79711	KEMSOUIGHYEWRY-UAJYNZJHSA-N	Not Available
PRR.8	MOL001924	paeoniflorin	53.87037516	480.510	0.78709	YKRGDOXKVOZESV-WRJNSLSBSA-N	Not Available
PRR.9	MOL001925	paeoniflorin_qt	68.17576188	318.350	0.39507	GWQHMWOOQLVRLG-JGAAPJFWSA-N	Not Available
PRR.10	MOL002714	baicalein	33.51891869	270.250	0.20888	FXNFHKRTJBSTCS-UHFFFAOYSA-N	5281605
PRR.11	MOL002776	Baicalin	40.12360996	446.390	0.75264	IKIIZLYTISPENI-ZFORQUDYSA-N	64982
PRR.12	MOL004355	Spinasterol	42.97936552	412.770	0.75534	JZVFJDZBLUFKCA-FXIAWGAOSA-N	5281331
PRR.13	MOL006990	(1S,2S,4R)-trans-2-hydroxy-1,8-cineole-B-D-glucopyranoside	30.25241156	332.440	0.27464	NWZYTZHMCGWGOF-KOQJZNESSA-N	Not Available

PRR.14	MOL006992	(2R,3R)-4-methoxyl-distylin	59.98325098	318.300	0.29949	ITWRRUUKFUXICF-CVEARBPZSA-N	Not Available
PRR.15	MOL006994	1-o-beta-d-glucopyranosyl-8-o-benzoylpaeonisuffrone_qt	36.01305796	302.350	0.29897	RZPMQFMUQXXSKB-VDNDLQMASA-N	Not Available
PRR.16	MOL006996	1-o-beta-d-glucopyranosylpaeonisuffrone_qt	65.08186655	332.380	0.35391	GHLQXVJMYVGPCU-WYGQYTNYSA-N	Not Available
PRR.17	MOL006999	stigmast-7-en-3-ol	37.42312067	414.790	0.75088	YSKVBPGQYRAUQO-NPAMSQCVSA-N	Not Available
PRR.18	MOL007003	benzoyl paeoniflorin	31.13866577	584.620	0.54227	LATYEZNGPQKAIK-RZRIYOMWSA-N	Not Available
PRR.19	MOL007004	Albiflorin	30.24614292	480.510	0.77038	QQUHMASGPODSIW-BNAVNNOTSA-N	Not Available
PRR.20	MOL007005	Albiflorin_qt	48.70011649	318.350	0.32628	WSVOZDIRZKFUCH-GVDRCSPZSA-N	Not Available
PRR.21	MOL007008	4-ethyl-paeoniflorin_qt	56.86957727	332.380	0.44483	UPDCGAQEQJAPLA-NLHADHJGSA-N	Not Available
PRR.22	MOL007012	4-o-methyl-paeoniflorin_qt	56.70351745	332.380	0.42562	LKWXNVNXHRSLRT-HZYJHYJVSA-N	Not Available
PRR.23	MOL007014	8-debenzoylpaeonidanin	31.74314844	390.430	0.45389	FICAFDZLGACQCA-GTDRRMEFSA-N	Not Available
PRR.24	MOL007016	Paeoniflorigenone	65.3341131	318.350	0.36711	BANPEMKDTXIFRE-LQPBRMSDSA-N	Not Available
PRR.25	MOL007018	9-ethyl-neo-paeoniaflorin A_qt	64.41989313	334.400	0.29598	LNQMUAGNQVZCET-BODMPHMZSA-N	Not Available
PRR.26	MOL007022	evofolinB	64.73661695	318.350	0.22232	QMYGRGKKZBRZKH-LBPRGKRZSA-N	Not Available
PRR.27	MOL007025	isobenzoylpaeoniflorin	31.13866577	584.620	0.54234	LATYEZNGPQKAIK-SYYPVXOBSA-N	Not Available

PRR.28	MOL007026	paeonin,a	20.34604497	372.410	0.36898	RNAOSOMTAGOVSH-LBOLTGTOSA-N	Not Available
PRR.29	MOL007028	paeonin,b	17.28582054	358.380	0.33542	JJUACHJWALUCMP-JLMIEGKISA-N	Not Available
PRR.30	MOL007030	paeonin,c	10.25770016	372.410	0.36127	OMNBOKMNIVQDNN-IWEBEACQSA-N	Not Available
PRR.31	MOL007002	paeonioflorin	10.22068492	480.510	0.7872	YKRGDOXKVOZESV-QVMPQSNJSA-N	Not Available
PRR.32	MOL007006	oxypaeoniflorin	12.9810553	496.510	0.782	FCHVXNVDFYXLIL-QVMPQSNJSA-N	Not Available
PRR.33	MOL001932	galloylpaeoniflorin	3.028419263	632.620	0.42335	KLFIUQCKSSAFFU-JRWJHFJZSA-N	Not Available
PRR.34	MOL003867	Paeonolide	6.298773621	460.480	0.6449	IDZZECHGWAZTIB-NYBIBFQCSA-N	442923
PRR.35	MOL005090	oxypaeoniflorin_qt	19.39679608	334.350	0.44099	FICRKDWVMQNZMX-JGAAPJFWSA-N	Not Available
PRR.36	MOL000874	paeonol	28.78723811	166.190	0.039185	UILPJVPSNHJFIK-UHFFFAOYSA-N	11092

GRR, Ginseng Radix et Rhizoma; AR, Astragali Radix; CF, Corni Fructus; LF, Lycii Fructus; SCF, Schisandrae Chinensis Fructus; EH, Epimedii Herba;

FTB, Fritillariae Thunbergii Bulbus; PRR, Paeoniae Rubra Radix; PF, Perillae Fructus; AJH, Ardisiae Japonicae Herba; CRP, Citri Reticulatae Pericarpium.

OB, oral bioavailability; DL, drug-likeness

_qt: the compound with glycosyl groups is deglycosylated by the rule of glycosidase hydrolysis reaction.

Table S2 The Candidate Compounds and their Potential Targets

MOL_ID	Degree	molecule_name	TAR_ID	Degree	target_name
MOL000098	87	quercetin	TAR00048	122	Androgen receptor
MOL000422	55	kaempferol	TAR00046	121	Estrogen receptor
MOL000358	51	beta-sitosterol	TAR00094	83	Prostaglandin G/H synthase 2
MOL000378	47	7-O-methylisomucronulatol	TAR00332	77	Dipeptidyl peptidase IV
MOL000449	44	Stigmasterol	TAR00017	68	Thrombin
MOL000787	44	Fumarine	TAR03276	63	Nuclear receptor coactivator 2
MOL004380	44	C-Homoerythrinan, 1,6-didehydro-3,15,16-trimethoxy-, (3.beta.)-	TAR00078	60	Peroxisome proliferator activated receptor gamma
MOL000006	41	luteolin	TAR00117	58	Carbonic anhydrase II
MOL004373	39	Anhydroicaritin	TAR02928	55	Trypsin-1
MOL000354	36	isorhamnetin	TAR00482	53	Cell division protein kinase 2
MOL000392	36	formononetin	TAR00006	50	Prostaglandin G/H synthase 1
MOL000371	35	3,9-di-O-methylnissolin	TAR00307	50	Estrogen receptor beta
MOL008457	35	Tetrahydroalstonine	TAR02966	50	Proto-oncogene serine/threonine-protein kinase Pim-1
MOL000296	34	hederagenin	TAR00422	49	Glycogen synthase kinase-3 beta
MOL000380	34	(6aR,11aR)-9,10-dimethoxy-6a,11a-dihydro-6H-benzofurano[3,2-c]chromen-3-ol	TAR00444	49	Heat shock protein HSP 90
MOL003542	34	8-Isopentenyl-kaempferol	TAR00229	48	mRNA of Protein-tyrosine phosphatase, non-receptor type 1
MOL002714	33	baicalein	TAR03025	45	Cyclin-A2
MOL004328	33	naringenin	TAR00165	43	Acetylcholinesterase
MOL005814	32	tangeretin	TAR00402	42	Mitogen-activated protein kinase 14
MOL001439	31	arachidonic acid	TAR00209	39	Progesterone receptor
MOL003648	31	Inermin	TAR00287	34	DNA topoisomerase

					II
MOL005384	31	suchilactone	TAR03907	34	Calmodulin
MOL004391	30	8-(3-methylbut-2-enyl)-2-phenyl-chromone	TAR00647	33	Serine/threonine-protein kinase Chk1
MOL005828	30	nobiletin	TAR00308	32	Glucocorticoid receptor
MOL004447	29	6-Methoxyl-2-acetyl-3-methyl-1,4-naphthoquinone-8-O-beta-D-glucopyranoside_qt	TAR00070	30	Sodium channel protein type 5 subunit alpha
MOL009650	29	Atropine	TAR00095	30	Nitric-oxide synthase, endothelial
MOL000223	28	caffeic acid	TAR00261	29	Beta-2 adrenergic receptor
MOL004422	27	Hexandraside E	TAR00309	27	Gamma-aminobutyric acid receptor subunit alpha-1
MOL004430	27	icariside I	TAR00499	27	Beta-lactamase
MOL005321	27	Frutinone A	TAR00699	26	mRNA of PKA Catalytic Subunit C-alpha
MOL000874	26	paeonol	TAR00158	24	Retinoic acid receptor RXR-alpha
MOL005100	26	5,7-dihydroxy-2-(3-hydroxy-4-methoxyphenyl)chroman-4-one	TAR00079	23	Coagulation factor Xa
MOL005406	26	atropine	TAR00038	21	Muscarinic acetylcholine receptor M1
MOL000417	25	Calycosin	TAR00252	21	Mineralocorticoid receptor
MOL001004	25	pelargonidin	TAR00016	18	Muscarinic acetylcholine receptor M3
MOL004393	25	anhydroicaritin	TAR00216	18	Alpha-1B adrenergic receptor
MOL008400	25	glycitein	TAR00172	16	CGMP-inhibited 3',5'-cyclic phosphodiesterase A
MOL001792	24	DFV	TAR00491	15	Phosphatidylinositol-4,5-bisphosphate

					3-kinase catalytic subunit, gamma isoform
MOL003044	24	Chryseriol	TAR00037	14	Potassium voltage-gated channel subfamily H member 2
MOL000492	23	(+)-catechin	TAR00123	13	Coagulation factor VII
MOL005356	23	Girinimbin	TAR00239	13	Sodium-dependent dopamine transporter
MOL005530	23	Hydroxygenkwanin	TAR00290	13	Sodium-dependent serotonin transporter
MOL005815	23	Citromitin	TAR00210	12	Muscarinic acetylcholine receptor M2
MOL009278	22	Laricitrin	TAR00565	12	Amine oxidase [flavin-containing] B
MOL000239	21	Jaranol	TAR03279	12	Nuclear receptor coactivator 1
MOL004386	21	Yinyanghuo E	TAR00186	11	Sodium-dependent noradrenaline transporter
MOL009646	21	7-O-Methylluteolin-6-C-beta-glucoside_qt	TAR00191	11	Alpha-1A adrenergic receptor
MOL004396	20	1,2-bis(4-hydroxy-3-methoxyphenyl)propan-1,3-diol	TAR00272	11	Alpha-1D adrenergic receptor
MOL004384	19	Yinyanghuo C	TAR03727	10	Calcium-activated potassium channel subunit alpha 1
MOL004385	19	Yinyanghuo D	TAR00063	9	Beta-1 adrenergic receptor
MOL000431	18	coumarin	TAR00086	8	Apoptosis regulator Bcl-2
MOL004367	17	olivil	TAR00154	8	Muscarinic acetylcholine receptor M4
MOL000442	16	1,7-Dihydroxy-3,9-dimethoxy pterocarpene	TAR00175	8	5-hydroxytryptamine 2A receptor
MOL004388	16	6-hydroxy-11,12-dim	TAR00581	8	Neuronal

		ethoxy-2,2-dimethyl-1,8-dioxo-2,3,4,8-tetrahydro-1H-isochromeno[3,4-h]isoquinolin-2-ium			acetylcholine receptor protein, alpha-7 chain
MOL004443	16	Zhebeiresinol	TAR01143	8	Cytochrome P450-cam
MOL000105	15	protocatechuic acid	TAR00007	7	Dopamine D1 receptor
MOL006992	15	(2R,3R)-4-methoxyl-distylin	TAR00087	7	Muscarinic acetylcholine receptor M5
MOL000387	14	Bifendate	TAR00126	7	Alpha-2C adrenergic receptor
MOL002773	13	beta-carotene	TAR00139	7	Vascular endothelial growth factor receptor 2
MOL001002	12	ellagic acid	TAR00141	7	Gamma-aminobutyric-acid receptor alpha-2 subunit
MOL001323	12	Sitosterol alpha1	TAR00265	7	Tumor necrosis factor
MOL003546	12	Aristolone	TAR00299	7	Mu-type opioid receptor
MOL011020	11	ardisianone B	TAR00414	7	Transcription factor AP-1
MOL002816	10	Bergenin	TAR00105	6	Alpha-2A adrenergic receptor
MOL004382	10	Yinyanghuo A	TAR00163	6	Delta-type opioid receptor
MOL004446	10	6-Methoxyl-2-acetyl-3-methyl-1,4-naphthoquinone-8-O-beta-D-glucopyranoside	TAR00200	6	Gamma-aminobutyric-acid receptor alpha-3 subunit
MOL005320	10	arachidonate	TAR00733	6	Glutathione S-transferase P
MOL008978	10	Gomisin R	TAR02132	6	Heme oxygenase 1
MOL010974	10	tri-O-methylnorbergenin	TAR00088	5	Arachidonate 5-lipoxygenase
MOL012893	10	(E)-(4-methylbenzylidene)-(4-phenyltriazol-1-yl)amine	TAR00113	5	5-hydroxytryptamine receptor 3A
MOL000953	9	CLR	TAR00354	5	Mitogen-activated protein kinase 1

MOL003541	9	()-alpha-Longipinene	TAR00568	5	Xanthine dehydrogenase/oxidase
MOL000890	8	(+)-alpha-Curcumene	TAR00646	5	Cellular tumor antigen p53
MOL001494	8	Mandenol	TAR00727	5	Alcohol dehydrogenase 1C
MOL001645	8	Linoleyl acetate	TAR00740	5	Vascular endothelial growth factor A
MOL002879	8	Diop	TAR01201	5	Ig gamma-1 chain C region
MOL005308	8	Aposiopolamine	TAR03216	5	Glutamate receptor 2
MOL001495	7	Ethyl linolenate	TAR00214	4	Alpha-2B adrenergic receptor
MOL005503	7	Cornudentanone	TAR00238	4	72 kDa type IV collagenase
MOL005545	7	cornin_qt	TAR00288	4	Aldose reductase
MOL006209	7	cyanin	TAR00353	4	Interstitial collagenase
MOL009604	7	14b-pregnane	TAR00365	4	Interferon gamma
MOL011019	7	ardisianone A	TAR00566	4	Amine oxidase [flavin-containing] A
MOL000027	6	alpha-Curcumene	TAR00573	4	Cell division protein kinase 4
MOL000263	6	oleanolic acid	TAR00621	4	Cytochrome P450 3A4
MOL000359	6	sitosterol	TAR03284	4	Gamma-aminobutyric-acid receptor subunit alpha-6
MOL000379	6	9,10-dimethoxypterocarpan-3-O-β-D-glucoside	TAR03978	4	Interleukin-2
MOL001979	6	LAN	TAR00011	3	Insulin receptor
MOL002776	6	Baicalin	TAR00106	3	5-hydroxytryptamine 1A receptor
MOL004355	6	Spinasterol	TAR00173	3	Histamine H1 receptor
MOL004624	6	Longikaurin A	TAR00181	3	Gamma-aminobutyric-acid receptor alpha-5 subunit
MOL005030	6	gondoic acid	TAR00203	3	5-hydroxytryptamine 2C receptor
MOL005344	6	ginsenoside rh2	TAR00284	3	Neuronal

					acetylcholine receptor subunit alpha-2
MOL007449	6	24-methylidenelophenol	TAR00292	3	D(2) dopamine receptor
MOL009622	6	Fucosterol	TAR00310	3	5-hydroxytryptamine 1B receptor
MOL009641	6	4alpha,24-dimethylcholesta-7,24-dienol	TAR00346	3	Urokinase-type plasminogen activator
MOL009681	6	Obtusifoliol	TAR00351	3	Interleukin-6
MOL010964	6	MAESANIN	TAR00431	3	Cell division control protein 2 homolog
MOL000622	5	Magnograndiolide	TAR00440	3	Vascular cell adhesion protein 1
MOL001680	5	Loganin	TAR00724	3	Cytochrome P450 1A2
MOL001771	5	poriferast-5-en-3beta-ol	TAR01172	3	Lysozyme
MOL002343	5	tetrandrine	TAR01696	3	Chymotrypsinogen B
MOL002883	5	Ethyl oleate (NF)	TAR02952	3	Glutathione S-transferase Mu 1
MOL003137	5	Leucanthoside	TAR03204	3	Aryl hydrocarbon receptor
MOL003867	5	Paeonolide	TAR03688	3	Glutathione S-transferase Mu 2
MOL004427	5	Icariside A7	TAR00056	2	D(1B) dopamine receptor
MOL005318	5	Dianthramine	TAR00246	2	Cytosolic phospholipase A2
MOL005348	5	Ginsenoside-Rh4_qt	TAR00267	2	Retinoic acid receptor RXR-gamma
MOL007026	5	paeonin,a	TAR00298	2	Epidermal growth factor receptor
MOL009618	5	24-ethylcholesta-5,22-dienol	TAR00318	2	Maltase-glucoamylase, intestinal
MOL009634	5	31-norlanosterol	TAR00349	2	Hepatocyte growth factor receptor
MOL009644	5	6-Fluoroindole-7-Dehydrocholesterol	TAR00350	2	Glycogen phosphorylase, muscle form
MOL009656	5	(E,E)-1-ethyl octadeca-3,13-dienoate	TAR00357	2	Serum paraoxonase/arylesterase 1

MOL009677	5	lanost-8-en-3beta-ol	TAR00418	2	Interleukin-1 beta
MOL009678	5	lanost-8-enol	TAR00427	2	E-selectin
MOL011003	5	5-ethoxy-2-hydroxy-3-[(8Z)-tridec-8-en-1-yl][1,4]benzoquinone	TAR00428	2	Myeloperoxidase
MOL000211	4	Mairin	TAR00436	2	Gap junction alpha-1 protein
MOL001510	4	24-epicampesterol	TAR00462	2	P-selectin
MOL004440	4	Peimisine	TAR00466	2	Tissue factor
MOL005531	4	Telocinobufagin	TAR00470	2	NAD(P)H dehydrogenase [quinone] 1
MOL008173	4	daucosterol_qt	TAR00597	2	Superoxide dismutase [Cu-Zn]
MOL008956	4	Angeloylgomisin O	TAR00648	2	Amyloid beta A4 protein
MOL008992	4	Wuweizisu C	TAR00704	2	Mitogen-activated protein kinase 8
MOL009621	4	24-methylenelanost-8-enol	TAR00726	2	Alcohol dehydrogenase 1B
MOL000033	3	(3S,8S,9S,10R,13R,14S,17R)-10,13-dimethyl-17-[(2R,5S)-5-propan-2-yloctan-2-yl]-2,3,4,7,8,9,11,12,14,15,16,17-dodecahydro-1H-cyclopenta[a]phenanthren-3-ol	TAR00734	2	Pro-epidermal growth factor
MOL000433	3	FA	TAR00735	2	Catalase
MOL001663	3	(4aS,6aR,6aS,6bR,8aR,10R,12aR,14bS)-10-hydroxy-2,2,6a,6b,9,9,12a-heptamethyl-1,3,4,5,6,6a,7,8,8a,10,11,12,13,14b-tetradecahydropicene-4a-carboxylic acid	TAR00789	2	Retinoic acid receptor RXR-beta
MOL001918	3	paeoniflorgenone	TAR01293	2	DNA topoisomerase 1
MOL003578	3	Cycloartenol	TAR02262	2	Nicotinate-nucleotide--dimethylbenzimidazole phosphoribosyltransfe

					rase
MOL004453	3	Peiminoside_qt	TAR02915	2	Retinoblastoma-associated protein
MOL005043	3	campest-5-en-3beta-ol	TAR02963	2	78 kDa glucose-regulated protein
MOL005399	3	alexandrin_qt	TAR03236	2	Aldo-keto reductase family 1 member C1
MOL005438	3	campesterol	TAR03575	2	Caspase-7
MOL005544	3	Cornin	TAR03586	2	cAMP-dependent protein kinase inhibitor alpha
MOL005557	3	lanosta-8,24-dien-3-ol,3-acetate	TAR03674	2	Glutathione S-transferase A2
MOL006999	3	stigmast-7-en-3-ol	TAR00049	1	Aldehyde dehydrogenase, mitochondrial
MOL007028	3	paeonin,b	TAR00092	1	4-aminobutyrate aminotransferase, mitochondrial
MOL008957	3	Schizandrer B	TAR00153	1	Ornithine decarboxylase
MOL008968	3	Gomisin-A	TAR00190	1	Low-density lipoprotein receptor
MOL009617	3	24-ethylcholest-22-enol	TAR00202	1	Aspartate aminotransferase, cytoplasmic
MOL009620	3	24-methyl-31-norlanost-9(11)-enol	TAR00222	1	Aldo-keto reductase family 1 member C3
MOL009633	3	31-norlanost-9(11)-enol	TAR00231	1	Acetyl-CoA carboxylase 1
MOL009635	3	4,24-methyllophenol	TAR00240	1	Glutathione reductase, mitochondrial
MOL009639	3	Lophenol	TAR00306	1	Multidrug resistance-associated protein 1
MOL009640	3	4alpha,14alpha,24-trimethylcholesta-8,24-dienol	TAR00363	1	Cathepsin D
MOL009642	3	4alpha-methyl-24-ethylcholesta-7,24-dienol	TAR00368	1	Tumor necrosis factor receptor superfamily member 1B

MOL012888	3	citrostadienol	TAR00374	1	Fatty acid synthase
MOL012891	3	(2E,4E,6E)-icosa-2,4,6-trienoic acid	TAR00377	1	Collagenase 3
MOL001924	2	paeoniflorin	TAR00396	1	Peroxisome proliferator activated receptor delta
MOL001996	2	Betulonic acid	TAR00404	1	Transient receptor potential cation channel subfamily V member 1
MOL004444	2	Ziebeimine	TAR00417	1	C-C motif chemokine 2
MOL005317	2	Deoxyharringtonine	TAR00421	1	Mitogen-activated protein kinase 3
MOL005376	2	Panaxadiol	TAR00434	1	Tissue-type plasminogen activator
MOL005467	2	Epicatechin gallate	TAR00441	1	Stromelysin-1
MOL005481	2	2,6,10,14,18-pentamethylicosa-2,6,10,14,18-pentaene	TAR00457	1	Thrombomodulin
MOL007018	2	9-ethyl-neo-paeoniaflorin A_qt	TAR00468	1	Neutrophil collagenase
MOL007030	2	paeonin,c	TAR00488	1	Tyrosine-protein kinase BTK
MOL007930	2	hesperidin	TAR00489	1	Type IV phosphodiesterase
MOL008974	2	Gomisin G	TAR00521	1	Leukotriene A-4 hydrolase
MOL009615	2	24-Methylenecycloartan-3beta,21-diol	TAR00593	1	Prostaglandin E2 receptor EP3 subtype
MOL009631	2	31-Norcyclolaudenol	TAR00622	1	Pancreatic alpha-amylase
MOL009653	2	Cycloeucalenol	TAR00642	1	Cytochrome P450 2A6
MOL010934	2	ardisianoside K_qt	TAR00725	1	Stromelysin-2
MOL010953	2	triterpenoid glycoside 1_qt	TAR00731	1	Collagen alpha-1(I) chain
MOL011002	2	5-ethoxy-2-hydroxy-3-[(10Z)-pentadec-10-en-1-yl][1,4]benzoquinone	TAR00744	1	NADPH--cytochrome P450 reductase
MOL000439	1	isomucronulatol-7,2'-	TAR00751	1	ATP-binding cassette

		di-O-glucosiole			sub-family G member 1
MOL004394	1	Anhydroicaritin-3-O-alpha-L-rhamnoside	TAR00766	1	Glutathione S-transferase A1
MOL004425	1	Icariin	TAR00781	1	Estrogen sulfotransferase
MOL004450	1	Chaksine	TAR00783	1	Aldehyde dehydrogenase, dimeric NADP-preferring
MOL004492	1	Chrysanthemaxanthin	TAR00787	1	Tumor necrosis factor receptor superfamily member 1A
MOL005360	1	malkangunin	TAR01173	1	Beta-galactosidase
MOL006994	1	1-o-beta-d-glucopyranosyl-8-o-benzoylpaeonisuffrone_qt	TAR01245	1	NADH-ubiquinone oxidoreductase chain 6
MOL006996	1	1-o-beta-d-glucopyranosylpaeonisuffrone_qt	TAR01306	1	Ferrichrome-iron receptor
MOL007005	1	Albiflorin_qt	TAR01366	1	DNA gyrase subunit B
MOL007016	1	Paeoniflorigenone	TAR01604	1	Glucose-6-phosphate 1-dehydrogenase
MOL009612	1	(24R)-4alpha-Methyl-24-ethylcholesta-7,25-dien-3beta-ylacetate	TAR01644	1	3-hydroxy-3-methylglutaryl-coenzyme A reductase
MOL009662	1	Lantadene A	TAR02875	1	Complement C1r subcomponent
MOL009665	1	Physcion-8-O-beta-D-gentiobioside	TAR02885	1	Collagen alpha-1(III) chain
MOL010976	1	triterpene glycoside 4_qt	TAR02916	1	ATP synthase subunit beta, mitochondrial
MOL010981	1	triterpenoid glycoside 3_qt	TAR02936	1	Collagen alpha-2(I) chain
MOL010983	1	2,5-Dihydroxy-3-[(10Z)-pentadec-10-en-1-yl]cyclohexa-2,5-diene-1,4-dione	TAR02998	1	Prostatic acid phosphatase
MOL010985	1	2-hydroxy-5-methoxy-3-pentadecaenylbenzoquinone	TAR03015	1	Pituitary adenylate cyclase-activating polypeptide

TAR03202	1	Sterol O-acyltransferase 1
TAR03256	1	Voltage-dependent L-type calcium channel subunit alpha-1S
TAR03412	1	Gamma-aminobutyric acid receptor subunit gamma-3
TAR03433	1	Egl nine homolog 1
TAR03611	1	Cytochrome c
TAR03781	1	Glutamate--cysteine ligase catalytic subunit
TAR03828	1	Ras-related C3 botulinum toxin substrate 1
TAR03952	1	Trypsin-3
TAR03967	1	Gamma-aminobutyric acid receptor subunit epsilon
TAR03971	1	Serine/threonine-protein phosphatase 2B catalytic subunit alpha isoform
TAR03986	1	Potassium channel subfamily K member 2
TAR03987	1	Microtubule-associated protein 2

Table S3 34 candidate compounds without targets and their corresponding herbs

MOL_ID	molecule_name	MW	inchikey	Herb_name
MOL010973	Rapanone	322.49	AMKNOBHCKRZHIO-UHFFFAOYSA-N	Ardisiae Japonicae Herba
MOL010982	2,5-dihydroxy-3-[(10Z)-pentadec-10-en-1-yl][1,4]benzoquinone	460.77	OZOJZESTKHHHQP-WAYWQWQTSA-N	Ardisiae Japonicae Herba
MOL000001	anthocyanidin	251.22	YLJQHJNWIHUOKW-UHFFFAOYSA-N	Citri Reticulatae Pericarpium
MOL001921	Lactiflorin	462.49	KEMSOUIGHYEWRY-UAJYNZJHSA-N	Paeoniae Rubra Radix
MOL001925	paeoniflorin_qt	318.35	GWQHMWOOQLVRLG-JGAAPJFWSA-N	Paeoniae Rubra Radix
MOL001932	galloylpaeoniflorin	632.62	KLFIUQCKSSAFFU-JRWJHFJZSA-N	Paeoniae Rubra Radix
MOL005090	oxypaeoniflorin_qt	334.35	FICRKDWVMQNZMX-JGAAPJFWSA-N	Paeoniae Rubra Radix
MOL006990	(1S,2S,4R)-trans-2-hydroxy-1,8-cineole-B-D-glucopyranoside	332.44	NWZYTZHMCGWGOF-KOQJZNESSA-N	Paeoniae Rubra Radix
MOL007002	paeonioflorin	480.51	YKRGDOXKVOZESV-QVMPQSNJSA-N	Paeoniae Rubra Radix
MOL007003	benzoyl paeoniflorin	584.62	LATYEZNGPQKAIK-RZRIYOMWSA-N	Paeoniae Rubra Radix
MOL007004	Albiflorin	480.51	QQUHMASGPODSIW-BNAVNNOTSA-N	Paeoniae Rubra Radix
MOL007006	oxypaeoniflorin	496.51	FCHVXNVDFYXLIL-QVMPQSNJSA-N	Paeoniae Rubra Radix
MOL007008	4-ethyl-paeoniflorin_qt	332.38	UPDCGAQEQJAPLA-NLHADHJGSA-N	Paeoniae Rubra Radix
MOL007012	4-o-methyl-paeoniflorin_qt	332.38	LKWXNVNXHRSLRT-HZYJHYJVS	Paeoniae Rubra Radix

			A-N	
MOL007014	8-debenzoylpaeonidanin	390.43	FICAFDZLGACQCA-GTDRRMEFSA-N	Paeoniae Rubra Radix
MOL007022	evofolinB	318.35	QMYGRGKKZBRZKH-LBPRGKRZSA-N	Paeoniae Rubra Radix
MOL007025	isobenzoylpaeoniflorin	584.62	LATYEZNGPQKAIK-SYYPVXOBSA-N	Paeoniae Rubra Radix
MOL009651	Cryptoxanthin monoepoxide	568.96	CMOLUFWHADIFGS-WZIUPQIASA-N	Lycii Fructus
MOL009660	methyl (1R,4aS,7R,7aS)-4a,7-dihydroxy-7-methyl-1-[(2S,3R,4S,5S,6R)-3,4,5-trihydroxy-6-(hydroxymethyl)oxan-2-yl]oxy-1,5,6,7a-tetrahydrocyclopenta[d]pyran-4-carboxylate	406.43	RWMXKBUPLSNIJL-VALIWVFLSA-N	Lycii Fructus
MOL009664	Physalin A	526.58	VELDODQHYQSJOF-RLGRCWQRSA-N	Lycii Fructus
MOL010234	delta-Carotene	536.96	WGIYGODPCLMGQH-GOXCNPTKSA-N	Lycii Fructus
MOL000374	5'-hydroxyiso-muronulatol-2',5'-di-O-glucoside	642.67	SRVGYVIWVOOXQO-FQRJZKGRSA-N	Astragali Radix
MOL000398	isoflavanone	316.33	JNSVNRWHSLLCBG-LLVKDONJSA-N	Astragali Radix
MOL000438	(3R)-3-(2-hydroxy-3,4-dimethoxyphenyl)chroman-7-ol	302.35	NQRBAPDEZYMKFL-NSHDSACASA-N	Astragali Radix
MOL005314	Celabenzine	379.55	LSYKFBZWBDMZLQ-OAQYLSRUSA-N	Ginseng Radix et Rhizoma
MOL005357	Gomisin B	514.62	XDVOVYYAPHHHBE-YGIMCSHQS	Ginseng Radix et Rhizoma

			A-N	
MOL005401	ginsenoside Rg5_qt	442.80	FCMJTYFZRUKOPU-FFFKMGBISA-N	Ginseng Radix et Rhizoma
MOL000554	gallic acid-3-O-(6'-O-galloyl)-glucoside	484.40	NRQUZRZEYPSZEY-IDXPAVDQSA-N	Corni Fructus
MOL003166	Swertiamarin	374.38	HEYZWPRKKUGDCR-QBXMEVCASA-N	Corni Fructus
MOL005486	3,4-Dehydrolycopen-16-al	548.92	FPLASDSFNINBIY-QETLVAEPSA-N	Corni Fructus
MOL005489	3,6-Digalloylglucose	484.40	LRSHPKZSGRNHIX-MPWSSXMOSA-N	Corni Fructus
MOL005546	cornuside	542.54	SMTKSCGLXONVGL-MUCSSEFLSA-N	Corni Fructus
MOL005547	cornuside_qt	380.38	KDGCSTFYBWGBNX-PJYBLOJUSA-N	Corni Fructus
MOL005552	gemin D	634.49	XKVYZLLWKHGKMT-UPMOLNEASA-N	Corni Fructus

Table S5 The Potential Targets and their corresponding diseases

TAR_ID	Degree	Target_name	DIS_ID	Degree	Disease_name
TAR00094	32	Prostaglandin G/H synthase 2	DIS00130	21	Cancer, unspecific
TAR00088	26	Arachidonate 5-lipoxygenase	DIS00051	11	Alzheimer's Disease
TAR00078	21	Peroxisome proliferator activated receptor gamma	DIS00055	9	Analgesics
TAR00292	18	D(2) dopamine receptor	DIS00072	9	Anxiety disorder, unspecified
TAR00724	16	Cytochrome P450 1A2	DIS00077	9	Asthma
TAR00173	15	Histamine H1 receptor	DIS00117	9	Breast cancer
TAR00238	13	72 kDa type IV collagenase	DIS00612	9	Pancreatic Cancer
TAR00261	13	Beta-2 adrenergic receptor	DIS00659	9	Prostate cancer
TAR00265	13	Tumor necrosis factor	DIS00711	9	Schizophrenia
TAR00298	13	Epidermal growth factor receptor	DIS00144	8	Cardiovascular disease, unspecified
TAR00402	13	Mitogen-activated protein kinase 14	DIS00416	8	Inflammation
TAR00482	13	Cell division protein kinase 2	DIS00586	8	Osteoarthritis
TAR00733	13	Glutathione S-transferase P	DIS00079	7	Atherosclerosis
TAR00444	12	Heat shock protein HSP 90	DIS00471	7	Lung Cancer
TAR00175	10	5-hydroxytryptamine 2A receptor	DIS00567	7	Noninsulin-dependent diabetes mellitus
TAR00353	10	Interstitial collagenase	DIS00569	7	Non-small Cell Lung Cancer
TAR00095	9	Nitric-oxide synthase, endothelial	DIS00572	7	Obesity
TAR00210	9	Muscarinic acetylcholine receptor M2	DIS00621	7	Parkinson's disease
TAR00377	9	Collagenase 3	DIS00703	7	Rheumatoid arthritis, unspecified
TAR00441	9	Stromelysin-1	DIS00228	6	Depression
TAR00017	8	Thrombin	DIS00489	6	Malaria
TAR00046	8	Estrogen receptor	DIS00744	6	Solid tumors

TAR00063	8	Beta-1 adrenergic receptor	DIS00193	5	Cocaine dependence
TAR00106	8	5-hydroxytryptamine 1A receptor	DIS00201	5	Colorectal Neoplasms
TAR00287	8	DNA topoisomerase II	DIS00234	5	Diabetes mellitus
TAR00349	8	Hepatocyte growth factor receptor	DIS00353	5	Heart Failure
TAR00374	8	Fatty acid synthase	DIS00397	5	Hypertension
TAR00396	8	Peroxisome proliferator activated receptor delta	DIS00550	5	Neurodegenerative diseases
TAR00079	7	Coagulation factor Xa	DIS00611	5	Pain, unspecified
TAR00351	7	Interleukin-6	DIS00174	4	Chronic inflammatory diseases
TAR00414	7	Transcription factor AP-1	DIS00196	4	Cognitive deficits
TAR00417	7	C-C motif chemokine 2	DIS00213	4	Crohns's Disease, unspecified
TAR00466	7	Tissue factor	DIS00527	4	Multiple Myeloma
TAR00070	6	Sodium channel protein type 5 subunit alpha	DIS00529	4	Multiple Sclerosis
TAR00163	6	Delta-type opioid receptor	DIS00533	4	Myocardial Infarction
TAR00246	6	Cytosolic phospholipase A2	DIS00689	4	Renal Cell Carcinoma
TAR00288	6	Aldose reductase	DIS00772	4	Thrombosis
TAR00299	6	Mu-type opioid receptor	DIS00041	3	Alcoholism
TAR00404	6	Transient receptor potential cation channel subfamily V member 1	DIS00082	3	Atopic Dermatitis
TAR00422	6	Glycogen synthase kinase-3 beta	DIS00093	3	Bacterial Infections
TAR00427	6	E-selectin	DIS00103	3	Bladder cancer
TAR00581	6	Neuronal acetylcholine receptor protein, alpha-7 chain	DIS00111	3	Brain Cancer
TAR00704	6	Mitogen-activated protein kinase 8	DIS00113	3	Brain injury

TAR00153	5	Ornithine decarboxylase	DIS00137	3	Cardiac arrhythmias
TAR00154	5	Muscarinic acetylcholine receptor M4	DIS00183	3	Chronic obstructive pulmonary disease, unspecified
TAR00165	5	Acetylcholinesterase	DIS00192	3	Coagulative disorders
TAR00181	5	Gamma-aminobutyric-acid receptor alpha-5 subunit	DIS00330	3	Glaucoma
TAR00307	5	Estrogen receptor beta	DIS00364	3	Hepatocellular carcinoma
TAR00332	5	Dipeptidyl peptidase IV	DIS00380	3	Hormone-refractory Prostate cancer
TAR00418	5	Interleukin-1 beta	DIS00430	3	Insulin resistance
TAR00468	5	Neutrophil collagenase	DIS00441	3	Ischemia
TAR00491	5	Phosphatidylinositol-4, 5-bisphosphate 3-kinase catalytic subunit, gamma isoform	DIS00443	3	Ischemic heart disease
TAR00521	5	Leukotriene A-4 hydrolase	DIS00446	3	Kaposi's Sarcoma
TAR00646	5	Cellular tumor antigen p53	DIS00505	3	Melanoma
TAR00731	5	Collagen alpha-1(I) chain	DIS00534	3	Myocardial infarction (MI)
TAR00787	5	Tumor necrosis factor receptor superfamily member 1A	DIS00558	3	Neurological diseases
TAR00038	4	Muscarinic acetylcholine receptor M1	DIS00595	3	Osteoporosis, unspecified
TAR00092	4	4-aminobutyrate aminotransferase, mitochondrial	DIS00597	3	Ovarian cancer
TAR00113	4	5-hydroxytryptamine receptor 3A	DIS00598	3	Ovarian Neoplasms
TAR00203	4	5-hydroxytryptamine 2C receptor	DIS00668	3	Psoriasis
TAR00310	4	5-hydroxytryptamine 1B receptor	DIS00669	3	Psoriasis and Psoriatic Disorders

TAR00428	4	Myeloperoxidase	DIS00749	3	Squamous cell carcinoma
TAR00086	3	Apoptosis regulator Bcl-2	DIS00773	3	Thrombotic disease
TAR00105	3	Alpha-2A adrenergic receptor	DIS00797	3	Tumors
TAR00117	3	Carbonic anhydrase II	DIS00008	2	Acute coronary syndromes
TAR00172	3	CGMP-inhibited 3',5'-cyclic phosphodiesterase A	DIS00022	2	Acute ureteric colic
TAR00214	3	Alpha-2B adrenergic receptor	DIS00028	2	Adult respiratory distress syndrome
TAR00229	3	mRNA of Protein-tyrosine phosphatase, non-receptor type 1	DIS00044	2	Allergic diseases
TAR00308	3	Glucocorticoid receptor	DIS00047	2	Allergic rhinitis, unspecified
TAR00421	3	Mitogen-activated protein kinase 3	DIS00075	2	Arthritis
TAR00565	3	Amine oxidase [flavin-containing] B	DIS00088	2	Autoimmune Diseases
TAR00566	3	Amine oxidase [flavin-containing] A	DIS00097	2	Behcet's disease
TAR00573	3	Cell division protein kinase 4	DIS00121	2	Breast Neoplasms
TAR00648	3	Amyloid beta A4 protein	DIS00179	2	Chronic myeloid leukemia
TAR00006	2	Prostaglandin G/H synthase 1	DIS00200	2	Colorectal cancer
TAR00016	2	Muscarinic acetylcholine receptor M3	DIS00203	2	Congestive Heart Failure
TAR00048	2	Androgen receptor	DIS00206	2	Coronary atherosclerosis
TAR00087	2	Muscarinic acetylcholine receptor M5	DIS00210	2	Cough
TAR00126	2	Alpha-2C adrenergic receptor	DIS00226	2	Dementia
TAR00139	2	Vascular endothelial	DIS00243	2	Diabetic neuropathy

		growth factor receptor 2			
TAR00141	2	Gamma-aminobutyric-acid receptor alpha-2 subunit	DIS00246	2	Diarrhea
TAR00191	2	Alpha-1A adrenergic receptor	DIS00255	2	Disorders of initiating and maintaining sleep [insomnias]
TAR00200	2	Gamma-aminobutyric-acid receptor alpha-3 subunit	DIS00258	2	Drug dependence
TAR00222	2	Aldo-keto reductase family 1 member C3	DIS00259	2	Drug Toxicity
TAR00231	2	Acetyl-CoA carboxylase 1	DIS00265	2	Dyspnea
TAR00240	2	Glutathione reductase, mitochondrial	DIS00279	2	Epilepsy
TAR00252	2	Mineralocorticoid receptor	DIS00332	2	Glioblastoma multiforme
TAR00284	2	Neuronal acetylcholine receptor subunit alpha-2	DIS00334	2	Gliomas
TAR00306	2	Multidrug resistance-associated protein 1	DIS00408	2	Hypothalamic-pituit ary ACTH function
TAR00309	2	Gamma-aminobutyric acid receptor subunit alpha-1	DIS00417	2	Inflammatory Bowel Disease
TAR00346	2	Urokinase-type plasminogen activator	DIS00418	2	Inflammatory diseases
TAR00350	2	Glycogen phosphorylase, muscle form	DIS00420	2	Inflammatory Disorders, Unspecified
TAR00354	2	Mitogen-activated protein kinase 1	DIS00425	2	Inflammatory skin disorder
TAR00363	2	Cathepsin D	DIS00488	2	Major Depressive Disorder
TAR00365	2	Interferon gamma	DIS00517	2	Migraine
TAR00431	2	Cell division control protein 2 homolog	DIS00522	2	Mood [affective] disorders
TAR00457	2	Thrombomodulin	DIS00538	2	Nausea and vomiting

TAR00462	2	P-selectin	DIS00542	2	Neoplasms
TAR00470	2	NAD(P)H dehydrogenase [quinone] 1	DIS00562	2	Neuropsychiatric disorders
TAR00488	2	Tyrosine-protein kinase BTK	DIS00607	2	Pain
TAR00597	2	Superoxide dismutase [Cu-Zn]	DIS00651	2	Precursor Cell Lymphoblastic Leukemia-Lymphom a
TAR00622	2	Pancreatic alpha-amylase	DIS00657	2	Proliferative diseases
TAR00734	2	Pro-epidermal growth factor	DIS00680	2	Rectal Neoplasms
TAR00735	2	Catalase	DIS00698	2	Respiratory diseases
TAR00740	2	Vascular endothelial growth factor A	DIS00720	2	Sepsis
TAR00751	2	ATP-binding cassette sub-family G member 1	DIS00737	2	Skin diseases
TAR00766	2	Glutathione S-transferase A1	DIS00743	2	Solid Tumor
TAR00783	2	Aldehyde dehydrogenase, dimeric NADP-preferring	DIS00754	2	Stroke
TAR00789	2	Retinoic acid receptor RXR-beta	DIS00765	2	Tardive dyskinesia
TAR00003	1	Nitric oxide synthase, inducible	DIS00770	2	Thromboembolic disorders
TAR00007	1	Dopamine D1 receptor	DIS00800	2	Ulcerative colitis
TAR00011	1	Insulin receptor	DIS00804	2	Urinary incontinence
TAR00037	1	Potassium voltage-gated channel subfamily H member 2	DIS00810	2	Vascular disease
TAR00049	1	Aldehyde dehydrogenase, mitochondrial	DIS00001	1	Abdominal aortic aneurysm
TAR00056	1	D(1B) dopamine receptor	DIS00002	1	Abortion, Spontaneous
TAR00123	1	Coagulation factor VII	DIS00005	1	Acne
TAR00158	1	Retinoic acid receptor	DIS00010	1	Acute lymphoblastic

		RXR-alpha			leukaemia
TAR00186	1	Sodium-dependent noradrenaline transporter	DIS00011	1	Acute lymphoblastic leukaemia (therapy-refractory)
TAR00190	1	Low-density lipoprotein receptor	DIS00012	1	Acute lymphoblastic leukemia (ALL)
TAR00202	1	Aspartate aminotransferase, cytoplasmic	DIS00013	1	Acute migraine
TAR00209	1	Progesterone receptor	DIS00015	1	Acute myeloid leukemia (AML)
TAR00216	1	Alpha-1B adrenergic receptor	DIS00019	1	Acute promyelocytic leukemia
TAR00239	1	Sodium-dependent dopamine transporter	DIS00024	1	Adenomatous polyposis
TAR00267	1	Retinoic acid receptor RXR-gamma	DIS00026	1	Adrenocorticotrophic hormone-secreting pituitary tumors
TAR00272	1	Alpha-1D adrenergic receptor	DIS00032	1	Advanced solid tumors
TAR00290	1	Sodium-dependent serotonin transporter	DIS00034	1	African trypanosomiasis
TAR00318	1	Maltase-glucoamylase, intestinal	DIS00039	1	Airway hyperreactivity
TAR00357	1	Serum paraoxonase/arylesterase 1	DIS00043	1	Allergic airway inflammation
TAR00368	1	Tumor necrosis factor receptor superfamily member 1B	DIS00058	1	Anemia
TAR00434	1	Tissue-type plasminogen activator	DIS00060	1	Anesthesia
TAR00436	1	Gap junction alpha-1 protein	DIS00062	1	Angina
TAR00440	1	Vascular cell adhesion protein 1	DIS00064	1	Angioedema
TAR00489	1	Type IV phosphodiesterase	DIS00065	1	Angiogenesis
TAR00499	1	Beta-lactamase	DIS00070	1	Antigen-induced decrease in coronary flow and cardiac anaphylaxis

TAR00568	1	Xanthine dehydrogenase/oxidase	DIS00073	1	Anxiety Disorders
TAR00593	1	Prostaglandin E2 receptor EP3 subtype	DIS00074	1	Arterial embolism and thrombosis
TAR00621	1	Cytochrome P450 3A4	DIS00076	1	Arthritis, Rheumatoid
TAR00642	1	Cytochrome P450 2A6	DIS00083	1	Atrial fibrillation and flutter
TAR00647	1	Serine/threonine-protein kinase Chk1	DIS00084	1	Attention-deficit hyperactivity disorder
TAR00699	1	mRNA of PKA Catalytic Subunit C-alpha	DIS00086	1	Autoimmune and sudden sensorineural hearing loss
TAR00725	1	Stromelysin-2	DIS00087	1	Autoimmune cardiomyopathy
TAR00726	1	Alcohol dehydrogenase 1B	DIS00090	1	B cell immunodeficiency
TAR00727	1	Alcohol dehydrogenase 1C	DIS00096	1	B-cell malignancies
TAR00744	1	NADPH--cytochrome P450 reductase	DIS00098	1	Benign prostate hyperplasia
TAR00781	1	Estrogen sulfotransferase	DIS00100	1	Bipolar Affective Disorder
			DIS00107	1	Bone Diseases, Metabolic
			DIS00114	1	Brain ischemia
			DIS00122	1	Bronchial asthma
			DIS00123	1	Bronchiolar carcinoma
			DIS00124	1	Bronchoconstriction (cold air-induced)
			DIS00125	1	Bronchospasm (histamine induced)
			DIS00127	1	Cachexia
			DIS00129	1	Cancer (multidrug resistant)
			DIS00133	1	Cancers
			DIS00134	1	Carcinoma in situ, unspecified
			DIS00135	1	Carcinoma, Non-Small-Cell

		Lung
DIS00139	1	Cardiac dysrhythmias
DIS00147	1	Carpal tunnel syndrome
DIS00150	1	Central nervous system diseases
DIS00157	1	Cerebral vasospasm
DIS00158	1	Cervical cancer
DIS00160	1	Chemotherapy-induced nausea and vomiting
DIS00164	1	Chondrosarcoma
DIS00172	1	Chronic hepatitis C
DIS00176	1	Chronic lymphocytic leukemia
DIS00177	1	Chronic lymphocytic leukemia (CLL)
DIS00178	1	Chronic Myelogenous Leukemia (CML)
DIS00182	1	Chronic Obstructive Pulmonary Disease (COPD)
DIS00185	1	Chronic pathological pain
DIS00188	1	Chronic rhinitis
DIS00190	1	Chronic urticaria
DIS00199	1	Colon cancer
DIS00204	1	Convulsions
DIS00205	1	Coronary Artery Disease
DIS00207	1	Coronary heart disease
DIS00208	1	Coronary syndromes
DIS00211	1	Crescentic glomerulonephritis
DIS00212	1	Crohns's Disease
DIS00219	1	Cystic Fibrosis
DIS00220	1	Cystitis
DIS00221	1	Cytokine-mediated diseases

DIS00224	1	Delirium
DIS00225	1	Delusional disorder
DIS00231	1	Dermatological disorders
DIS00232	1	Detrusor hyperreflexia
DIS00236	1	Diabetes Mellitus Type 2
DIS00239	1	Diabetic complications
DIS00242	1	Diabetic nephropathy
DIS00244	1	Diabetic retinopathy
DIS00250	1	Dilated cardiomyopathy
DIS00251	1	Disabling peak-dose dyskinesias
DIS00256	1	Disseminated intravascular coagulation
DIS00264	1	Dysmenorrhea, unspecified
DIS00269	1	Emphysema
DIS00270	1	Endocrine independent cancer
DIS00271	1	Endometrial carcinoma
DIS00272	1	Endometrial Neoplasms
DIS00273	1	Endometriosis
DIS00274	1	Endotoxemia
DIS00275	1	Endotoxin-induced myocardial neutrophil accumulation and contractile dysfunction
DIS00278	1	Epidermal hyperplasia
DIS00280	1	Epileptic seizures
DIS00282	1	ER beta-positive prostate tumors

DIS00283	1	Erectile dysfunction
DIS00286	1	Essential (primary) hypertension
DIS00295	1	Fatigue
DIS00302	1	Fractures, Bone
DIS00304	1	Fungal diseases
DIS00308	1	Gastric Cancer
DIS00310	1	Gastric emptying disorders
DIS00316	1	Gastrointestinal Cancers
DIS00319	1	Gastrointestinal Neoplasms
DIS00321	1	Gastrointestinal Stromal Tumors (GIST)
DIS00325	1	Genitourinary tumors
DIS00327	1	Gestational hypertension
DIS00329	1	Gilles de la Tourette's disorder
DIS00335	1	Glomerulonephritis
DIS00336	1	Gout
DIS00345	1	HCV infection
DIS00346	1	Head and Neck Cancer
DIS00347	1	Head and Neck Neoplasms
DIS00349	1	Head and neck tumors
DIS00350	1	Hearing Loss
DIS00352	1	Heart disease, unspecified
DIS00356	1	Helminth infection
DIS00357	1	Hematological Malignancies
DIS00359	1	Heparin-induced thrombocytopenia type II
DIS00365	1	Hepatocellular Carcinoma (HCC)

DIS00367	1	HER2-positive Metastatic Breast Cancer
DIS00370	1	Hereditary Polyposis Syndromes
DIS00372	1	Herpes virus infection
DIS00384	1	Huntington's disease
DIS00392	1	Hyperimmunoglobulinemia D
DIS00393	1	Hyperinflammatory provoked organ injury
DIS00395	1	Hyperlipidemia
DIS00398	1	Hypertension, Angina
DIS00402	1	Hypertrophic vascular disease
DIS00403	1	Hypoglycemia
DIS00407	1	Hypotension
DIS00409	1	Hypothermia
DIS00410	1	Hypoxic-ischemic encephalopathy
DIS00413	1	Immunodeficiency
DIS00419	1	Inflammatory diseases associated with hypoxia
DIS00421	1	Inflammatory lung disease
DIS00429	1	Insomnia
DIS00432	1	Insulin-dependent diabetes mellitus
DIS00434	1	Intimal hyperplasia
DIS00438	1	Irritable bowel syndrome
DIS00442	1	Ischemia reperfusion injuries
DIS00444	1	Ischemic injury of the liver
DIS00445	1	Ischemic Stroke
DIS00447	1	Kidney Cancer
DIS00449	1	Kidney

		Transplantation
DIS00454	1	Leishmania Infections
DIS00458	1	Leukemia, Myeloid
DIS00460	1	Leukemia, Unspecified
DIS00461	1	Lipid metabolic disorders
DIS00474	1	Lymphangiomatosis
DIS00485	1	Macular Degeneration
DIS00487	1	Magnesium deficiency dermatitis
DIS00491	1	Malignancies
DIS00496	1	Malignant mesothelioma
DIS00499	1	Manic disorder
DIS00508	1	Meningioma
DIS00509	1	Mesothelioma
DIS00510	1	Metabolic Disease
DIS00512	1	Metabolic syndrome X
DIS00514	1	Metastatic osteosarcoma in the lung
DIS00523	1	Motion sickness
DIS00524	1	Motor disorder
DIS00525	1	Motor neurone disease
DIS00528	1	Multiple organ failure
DIS00537	1	Nasopharyngeal Cancer (NPC)
DIS00546	1	Nephrosis
DIS00548	1	Neural Tube Defects
DIS00552	1	Neurogenic bladder
DIS00555	1	Neuroleptic malignant syndrome
DIS00556	1	Neurologic and psychiatric diseases
DIS00561	1	Neuropathic pain
DIS00563	1	Neurotoxicity

		Syndromes
DIS00564	1	Neutropenia
DIS00565	1	Non-Hodgkin's Lymphoma
DIS00571	1	Not Available
DIS00573	1	Obsessive-compulsive disorder
DIS00574	1	Obstructive airway disease
DIS00577	1	Oesophageal cancer
DIS00578	1	Opioid dependence
DIS00579	1	Opioid-induced bowel dysfunction
DIS00585	1	Oropharyngeal squamous cell carcinoma
DIS00587	1	Osteoarthritis pain
DIS00590	1	Osteonecrosis
DIS00592	1	Osteoporosis
DIS00600	1	Over-expression of TF
DIS00609	1	Pain, Acute or Chronic
DIS00614	1	Pancreatic disease
DIS00618	1	Parasitic diseases
DIS00619	1	Parkinson Disease
DIS00620	1	Parkinsonian symptoms
DIS00622	1	Pathological angiogenesis
DIS00624	1	Pediatric
DIS00626	1	Periodic fever syndrome
DIS00629	1	Peripheral Nervous System Diseases
DIS00632	1	Peripheral Vascular Disease
DIS00633	1	Peutz-Jeghers syndrome
DIS00634	1	Peyronie's disease
DIS00643	1	Polyarthritis, unspecified

DIS00647	1	Postmenopausal symptoms
DIS00649	1	Postoperative nausea and vomiting
DIS00650	1	Postoperative residual curarisation
DIS00655	1	Prion diseases
DIS00661	1	Prostate cancer (hormone refractory)
DIS00662	1	Prostate cancer (metastatic)
DIS00670	1	Psychiatric illness
DIS00672	1	Pulmonary Disease, Chronic Obstructive
DIS00674	1	Pulmonary fibrosis
DIS00675	1	Pulmonary hypertension
DIS00676	1	Pyresis
DIS00677	1	Radiation enteropathy
DIS00679	1	Raynaud's syndrome
DIS00683	1	Refractory Hematological Malignancies
DIS00684	1	Refractory partial epilepsy
DIS00692	1	Renal failure
DIS00694	1	Renal ischemia-reperfusion injury
DIS00699	1	Respiratory distress syndrome
DIS00701	1	Rheumatic diseases
DIS00702	1	Rheumatoid arthritis
DIS00713	1	Seasonal allergic rhinitis
DIS00726	1	Shy-Drager syndrome
DIS00730	1	Sjogren-Larsson syndrome
DIS00733	1	Skeletal muscle associated spasticity

DIS00734	1	Skeletal muscle wasting
DIS00735	1	Skeletal muscle weakness
DIS00739	1	Sleeping Sickness
DIS00740	1	Small cell lung cancer
DIS00741	1	Smooth muscle hyperplasia
DIS00742	1	Social phobias
DIS00747	1	Spinal and bulbar muscular atrophy
DIS00752	1	Streptococcus pneumoniae infections
DIS00757	1	Sustained ventricular tachycardia
DIS00759	1	Systemic arterial vasodilation
DIS00762	1	Systemic-onset juvenile idiopathic arthritis
DIS00767	1	Testicular cancer
DIS00771	1	Thromboembolism
DIS00774	1	Thyroid follicular carcinoma
DIS00779	1	Tobacco Use Disorder
DIS00786	1	Traumatic brain injury
DIS00789	1	Tremor, unspecified
DIS00790	1	Trichomoniasis
DIS00802	1	Urge incontinence
DIS00803	1	Urinary Bladder Neoplasms
DIS00808	1	Urological cancers
DIS00813	1	Vascular injury response
DIS00814	1	Vascular lesion regression
DIS00816	1	Vasospasm
DIS00823	1	Viral infection,

			unspecified
	DIS00829	1	Vitamin B6 deficiency
	DIS00831	1	Vomiting
	DIS00833	1	Waldenstrom's macroglobulinemia
