# Supplementary material for: Systems pharmacology-based dissection of mechanisms of Chinese medicinal formula Bufei Yishen as an effective treatment for chronic obstructive pulmonary disease
Source: Sci Rep. 2015 Oct 15;5:15290. doi: 10.1038/srep15290 (PMC4606809; doi:10.1038/srep15290)
Supplement: Supplementary Table S4 [file srep15290-s2.doc]

**Systems pharmacology-based dissection of mechanisms of Chinese medicinal formula Bufei Yishen as an effective treatment for chronic obstructive pulmonary disease**

Jiansheng Li^1, 2, a^, *, Peng Zhao^1, 2, a^, Ya Li^1, 2^, Yange Tian^1, 2^, Yonghua Wang^2, 3^

^1^ Henan University of Traditional Chinese Medicine, Zhengzhou 450046, China

^2^ Collaborative Innovation Center for Respiratory Disease Diagnosis and Treatment & Chinese Medicine Development of Henan Province, Zhengzhou 450046, China

^3^ Center of Bioinformatics, Northwest A & F University, Yangling, Shaanxi 712100, China

^a^ These authors equally contribute to this paper.

*Corresponding author

E-mail: li_js8@163.com (LJS); Tel.: +86-371-65676568

| Table S4 Pharmacokinetic property predictions for all compounds | | | | | | | |
| --- | --- | --- | --- | --- | --- | --- | --- |
| **Herb** | **MOL_ID** | **molecule_name** | **OB** | **mw** | **drug-**  **likeness** | **inchikey** | **PubChem_Cid** |
| GRR.1 | MOL000029 | beta-Humulene | 26.86643239 | 204.390 | 0.061239 | HAVYZKHVTLAPDZ-PPGMXFKZSA-N | 5318102 |
| GRR.2 | MOL000035 | beta-Selinene | 24.38821416 | 204.390 | 0.081081 | YOVSPTNQHMDJAG-QLFBSQMISA-N | 442393 |
| GRR.3 | MOL000036 | beta-caryophyllene | 29.70229451 | 204.390 | 0.089893 | NPNUFJAVOOONJE-GFUGXAQUSA-N | 5281515 |
| GRR.4 | MOL000066 | alloaromadedrene | 53.46135969 | 204.390 | 0.10414 | ITYNGVSTWVVPIC-PDWCTOEPSA-N | 44584667 |
| GRR.5 | MOL000069 | palmitic acid | 19.2965647 | 256.480 | 0.098573 | IPCSVZSSVZVIGE-UHFFFAOYSA-N | 985 |
| GRR.6 | MOL000269 | Elemicin | 21.94067579 | 208.280 | 0.060865 | BPLQKQKXWHCZSS-UHFFFAOYSA-N | 10248 |
| GRR.7 | MOL000358 | beta-sitosterol | 36.91390583 | 414.790 | 0.75123 | KZJWDPNRJALLNS-VJSFXXLFSA-N | 222284 |
| GRR.8 | MOL000422 | kaempferol | 41.88224954 | 286.250 | 0.24066 | IYRMWMYZSQPJKC-UHFFFAOYSA-N | 5280863 |
| GRR.9 | MOL000449 | Stigmasterol | 43.82985158 | 412.770 | 0.75665 | HCXVJBMSMIARIN-PHZDYDNGSA-N | 5280794 |
| GRR.10 | MOL000628 | darutoside | 21.31817387 | 574.930 | 0.63003 | VWDLOXMZIGUBKM-CQJGLIEWSA-N | Not Available |
| GRR.11 | MOL000676 | DBP | 64.5416405 | 278.380 | 0.13409 | DOIRQSBPFJWKBE-UHFFFAOYSA-N | 3026 |
| GRR.12 | MOL000749 | Linoleic | 41.90443602 | 280.500 | 0.14468 | OYHQOLUKZRVURQ-AVQMFFATSA-N | 5282457 |
| GRR.13 | MOL000787 | Fumarine | 59.26250458 | 353.400 | 0.82694 | GPTFURBXHJWNHR-UHFFFAOYSA-N | Not Available |
| GRR.14 | MOL000864 | MYS | 13.9810568 | 212.470 | 0.04922 | YCOZIPAWZNQLMR-UHFFFAOYSA-N | 12391 |
| GRR.15 | MOL000874 | paeonol | 28.78723811 | 166.190 | 0.039185 | UILPJVPSNHJFIK-UHFFFAOYSA-N | 11092 |
| GRR.16 | MOL000879 | methyl palmitate | 18.08756063 | 270.510 | 0.11594 | FLIACVVOZYBSBS-UHFFFAOYSA-N | 8181 |
| GRR.17 | MOL000886 | tetradecane | 15.94226412 | 198.440 | 0.039319 | BGHCVCJVXZWKCC-UHFFFAOYSA-N | 12389 |
| GRR.18 | MOL000908 | beta-elemene | 25.63362343 | 204.390 | 0.060519 | OPFTUNCRGUEPRZ-QLFBSQMISA-N | 6918391 |
| GRR.19 | MOL000935 | Hepanal | 53.83317567 | 204.390 | 0.10397 | SPCXZDDGSGTVAW-XIDUGBJDSA-N | 15560276 |
| GRR.20 | MOL000942 | (1R,4S,4aR,8aR)-4-isopropyl-1,6-dimethyl-3,4,4a,7,8,8a-hexahydro-2H-naphthalen-1-ol | 31.67305829 | 222.410 | 0.093126 | LHYHMMRYTDARSZ-BYNSBNAKSA-N | 10398656 |
| GRR.21 | MOL000968 | beta-Bisabolene | 29.58966171 | 204.390 | 0.055235 | XZRVRYFILCSYSP-HNNXBMFYSA-N | 68128 |
| GRR.22 | MOL001212 | Loxanol V | 14.18992173 | 214.440 | 0.051747 | HLZKNKRTKFSKGZ-UHFFFAOYSA-N | 8209 |
| GRR.23 | MOL001218 | Pisol | 18.49651888 | 186.380 | 0.032811 | LQZZUXJYWNFBMV-UHFFFAOYSA-N | 8193 |
| GRR.24 | MOL001312 | 9-HEXADECENOIC ACID | 35.77585321 | 254.460 | 0.1 | SECPZKHBENQXJG-BQYQJAHWSA-N | 5282745 |
| GRR.25 | MOL001392 | Methyl myristate | 19.68203501 | 242.450 | 0.079634 | ZAZKJZBWRNNLDS-UHFFFAOYSA-N | 31284 |
| GRR.26 | MOL001396 | PENTADECYLIC ACID | 20.18459315 | 242.450 | 0.081479 | WQEPLUUGTLDZJY-UHFFFAOYSA-N | 13849 |
| GRR.27 | MOL001641 | METHYL LINOLEATE | 41.93435814 | 294.530 | 0.16711 | WTTJVINHCBCLGX-NQLNTKRDSA-N | 5284421 |
| GRR.28 | MOL001706 | 2,6-dimethyl-3,7-octadiene-2,6-diol | 52.40225788 | 170.280 | 0.031108 | QEOHJVNDENHRCH-VQCYPWCPSA-N | Not Available |
| GRR.29 | MOL001738 | MLI | 21.68952295 | 104.070 | 0.0092112 | OFOBLEOULBTSOW-UHFFFAOYSA-N | 23511544 |
| GRR.30 | MOL001817 | Methyl stearate | 16.80312275 | 298.570 | 0.16341 | HPEUJPJOZXNMSJ-UHFFFAOYSA-N | 8201 |
| GRR.31 | MOL001818 | Methyl palmitelaidate | 34.6122634 | 268.490 | 0.11751 | IZFGRAGOVZCUFB-CMDGGOBGSA-N | 638303 |
| GRR.32 | MOL001819 | METHYL PENTADECANOATE | 18.81525029 | 256.480 | 0.096511 | XIUXKAZJZFLLDQ-UHFFFAOYSA-N | 23518 |
| GRR.33 | MOL001949 | panaxynol | 42.43849753 | 244.410 | 0.097799 | UGJAEDFOKNAMQD-QXPKXGMISA-N | 5281149 |
| GRR.34 | MOL001965 | Dauricine (8CI) | 23.65048221 | 624.840 | 0.36975 | AQASRZOCERRGBL-ROJLCIKYSA-N | 73400 |
| GRR.35 | MOL002121 | (1S,4E,8E,10R)-4,8,11,11-tetramethylbicyclo[8.1.0]undeca-4,8-diene | 21.69102126 | 204.390 | 0.083616 | VPDZRSSKICPUEY-AGJXFOQBSA-N | 11820258 |
| GRR.36 | MOL002136 | neocnidilide | 83.83010305 | 194.300 | 0.065252 | UPJFTVFLSIQQAV-ONGXEEELSA-N | 12315453 |
| GRR.37 | MOL002137 | OCT | 29.71597607 | 114.260 | 0.0072628 | TVMXDCGIABBOFY-UHFFFAOYSA-N | 356 |
| GRR.38 | MOL002307 | 20-Hexadecanoylingenol | 28.20395415 | 586.940 | 0.68297 | DSTCZBGJCUOFLM-SXNKARFESA-N | Not Available |
| GRR.39 | MOL002312 | [(3S,4R,5R)-5-[[(2R,3S,4S,5R,6S)-6-(2-acetyl-5-methoxyphenoxy)-3,4,5-trihydroxyoxan-2-yl]methoxy]-3,4-dihydroxyoxolan-3-yl]methyl 3,4,5-trihydroxybenzoate | 5.614162772 | 612.590 | 0.63433 | QNVPMKAFCVXMFH-NDDHQHNUSA-N | 9986231 |
| GRR.40 | MOL002323 | L-Adenosine | 18.06175721 | 267.280 | 0.17879 | OIRDTQYFTABQOQ-DEGSGYPDSA-N | 448374 |
| GRR.41 | MOL002377 | Kaempferol-3-arabofuranoside | 2.728217561 | 418.380 | 0.65082 | POQICXMTUPVZMX-BQCJVYABSA-N | Not Available |
| GRR.42 | MOL002526 | 3691-11-0 | 23.65592856 | 204.390 | 0.072163 | YHAJBLWYOIUHHM-GUTXKFCHSA-N | 94275 |
| GRR.43 | MOL002669 | Campesteryl ferulate | 22.09784892 | 576.940 | 0.59241 | SWIWTAJTJOYCTB-WNUOVGGOSA-N | Not Available |
| GRR.44 | MOL002879 | Diop | 43.59332547 | 390.620 | 0.39247 | IJFPVINAQGWBRJ-UHFFFAOYSA-N | 33934 |
| GRR.45 | MOL003346 | Psuedohypericin | 16.943378 | 520.460 | 0.073558 | YXBUQQDFTYOHQI-UHFFFAOYSA-N | Not Available |
| GRR.46 | MOL003648 | Inermin | 65.83093145 | 284.280 | 0.53754 | HUKSJTUUSUGIDC-BDJLRTHQSA-N | Not Available |
| GRR.47 | MOL003845 | Folinic acid | 23.60043457 | 473.500 | 0.74014 | WGEMODLYPVOIIW-QWHCGFSZSA-N | Not Available |
| GRR.48 | MOL003902 | methyl (Z)-icos-11-enoate | 29.49442949 | 324.610 | 0.22588 | RBKMRGOHCLRTLZ-KHPPLWFESA-N | 5463047 |
| GRR.49 | MOL004100 | N-Salicylidene-salicylamine | 95.45748766 | 227.280 | 0.10926 | UASXFKHZBZLKLY-DHDCSXOGSA-N | Not Available |
| GRR.50 | MOL004174 | epsilon-Cadinene | 16.41043148 | 204.390 | 0.077488 | NOLWRMQDWRAODO-KKUMJFAQSA-N | 12302130 |
| GRR.51 | MOL004237 | δ-elemene | 25.99022265 | 204.390 | 0.060207 | MXDMETWAEGIFOE-HUUCEWRRSA-N | 12309451 |
| GRR.52 | MOL004275 | (1R,4E,7E,11R)-1,5,9,9-tetramethyl-12-oxabicyclo[9.1.0]dodeca-4,7-diene | 23.65865221 | 220.390 | 0.09682 | RKQDKXOBRXTSFS-UOAUIWSESA-N | 14038843 |
| GRR.53 | MOL004492 | Chrysanthemaxanthin | 38.72398115 | 584.960 | 0.58352 | JRHJXXLCNATYLS-HYHFCDFPSA-N | Not Available |
| GRR.54 | MOL004498 | 12-O-Nicotinoylisolineolone | 20.70427369 | 469.630 | 0.83406 | OQFVSKGDZKMZEH-WCYOEAGXSA-N | Not Available |
| GRR.55 | MOL004647 | TDA | 22.31567357 | 214.390 | 0.054133 | SZHOJFHSIKHZHA-UHFFFAOYSA-N | 12530 |
| GRR.56 | MOL005155 | ginsenoside Ro_qt | 17.62097764 | 455.770 | 0.75859 | MIJYXULNPSFWEK-CGXHKNHBSA-N | 7055468 |
| GRR.57 | MOL005269 | (+)-Maalioxide | 55.92680377 | 222.410 | 0.127 | PZKNYJWHOZUWDF-OSRDXIQISA-N | Not Available |
| GRR.58 | MOL005270 | n-Heptadecanol | 12.96641747 | 256.530 | 0.093861 | GOQYKNQRPGWPLP-UHFFFAOYSA-N | 15076 |
| GRR.59 | MOL005271 | 1-HEXADECYNE | 3.943791985 | 222.460 | 0.063045 | UCIDYSLOTJMRAM-UHFFFAOYSA-N | 12396 |
| GRR.60 | MOL005272 | 13-Tetradecenyl acetate | 36.76038067 | 254.460 | 0.10041 | DZXBZPMJYIXTTI-UHFFFAOYSA-N | 521718 |
| GRR.61 | MOL005273 | 16-Oxoseratenediol | 15.10080154 | 456.780 | 0.75175 | VNOKAWVKCFUZGK-PUFFNIGPSA-N | Not Available |
| GRR.62 | MOL005274 | Neohexane | 37.80941867 | 86.200 | 0.006562 | HNRMPXKDFBEGFZ-UHFFFAOYSA-N | 6403 |
| GRR.63 | MOL005275 | 2,3,4-Trimethyldecane | 16.147675 | 184.410 | 0.03038 | YFHGNGNLIWGTTR-CHWSQXEVSA-N | Not Available |
| GRR.64 | MOL005276 | 2,3,8-Trimethyldecane | 5.508787248 | 184.410 | 0.030214 | DXHBSDCNOWJBQW-CHWSQXEVSA-N | Not Available |
| GRR.65 | MOL005277 | 2,6,10,15-tetramethylheptadecane | 13.73377727 | 296.650 | 0.12959 | ZZEQNXPBKOFTBG-ACRUOGEOSA-N | Not Available |
| GRR.66 | MOL005278 | 2-METHYLTRIDECANE | 5.74985642 | 198.440 | 0.039044 | CJBFZKZYIPBBTO-UHFFFAOYSA-N | 15269 |
| GRR.67 | MOL005279 | ginsenoside-Rh1 | 3.858921997 | 638.980 | 0.57239 | RAQNTCRNSXYLAH-UQORERNTSA-N | Not Available |
| GRR.68 | MOL005280 | ginsenoside-Rh1_qt | 20.12704418 | 476.820 | 0.77963 | SHCBCKBYTHZQGZ-AJZBJTNWSA-N | Not Available |
| GRR.69 | MOL005281 | 20(S)-Ginsenoside-Rh1 | 3.942248531 | 638.980 | 0.57259 | RAQNTCRNSXYLAH-SLRRGMQASA-N | Not Available |
| GRR.70 | MOL005282 | 20(S)-Ginsenoside-Rh1_qt | 20.12704418 | 476.820 | 0.77995 | SHCBCKBYTHZQGZ-IQABTIBQSA-N | Not Available |
| GRR.71 | MOL005283 | 20(S)-ginsenoside-Rg2 | 10.09229647 | 785.140 | 0.25819 | AGBCLJAHARWNLA-CBWWKSIWSA-N | Not Available |
| GRR.72 | MOL005284 | (3S,5R,6S,8R,9R,10R,12R,13R,14R,17S)-17-[(2S)-2-hydroxy-6-methylhept-5-en-2-yl]-4,4,8,10,14-pentamethyl-2,3,5,6,7,9,11,12,13,15,16,17-dodecahydro-1H-cyclopenta[a]phenanthrene-3,6,12-triol | 20.12704418 | 476.820 | 0.77961 | SHCBCKBYTHZQGZ-CJPZEJHVSA-N | 11468733 |
| GRR.73 | MOL005285 | 20(s)-protopanaxadiol | 29.69203747 | 460.820 | 0.77148 | PYXFVCFISTUSOO-VUFVRDRTSA-N | 9920281 |
| GRR.74 | MOL005286 | 20(R)-ginsenoside Rg2 | 10.09229647 | 785.140 | 0.25878 | AGBCLJAHARWNLA-LVJGBEOWSA-N | Not Available |
| GRR.75 | MOL005287 | 20-(S)-Ginsenoside-Rg3 | 13.68989131 | 785.140 | 0.21593 | RWXIFXNRCLMQCD-LYTHEZCASA-N | Not Available |
| GRR.76 | MOL005288 | 20-(S)-Ginsenoside-Rg3_qt | 29.69203747 | 460.820 | 0.7707 | PYXFVCFISTUSOO-DCDFIXCCSA-N | Not Available |
| GRR.77 | MOL005289 | 3,4-Dimethylheptane | 46.67453501 | 128.290 | 0.012044 | MAKRYGRRIKSDES-RKDXNWHRSA-N | 59954486 |
| GRR.78 | MOL005290 | 3,5-Dimethyl-p-anisic acid | 61.1054575 | 180.220 | 0.047297 | WXVQURJGDUNJCS-UHFFFAOYSA-N | 88944 |
| GRR.79 | MOL005291 | 3-O-beta-D-Glucuronopyranosyl gypsogenin | 8.680585119 | 646.900 | 0.32446 | NUSHOJSYOLRGAX-IIAUIHIBSA-N | Not Available |
| GRR.80 | MOL005292 | 3-O-beta-D-Glucuronopyranosyl gypsogenin_qt | 23.71646736 | 470.760 | 0.7457 | QMHCWDVPABYZMC-YCAMUBHUSA-N | Not Available |
| GRR.81 | MOL005293 | 3-Ethyl-3-methylheptane | 37.33447082 | 142.320 | 0.017734 | HSOMNBKXPGCNBH-UHFFFAOYSA-N | 140213 |
| GRR.82 | MOL005294 | 3-methylheptane | 37.09624917 | 114.260 | 0.0079803 | LAIUFBWHERIJIH-QMMMGPOBSA-N | 12263095 |
| GRR.83 | MOL005295 | 3-methylundecane | 6.57435271 | 170.380 | 0.023915 | HTZWVZNRDDOFEI-GFCCVEGCSA-N | 14656881 |
| GRR.84 | MOL005296 | 4-Methyldodecane | 6.389487134 | 184.410 | 0.030047 | UZTXSMATBUWDDZ-CYBMUJFWSA-N | Not Available |
| GRR.85 | MOL005297 | 5-Isobutylnonane | 6.080089889 | 184.410 | 0.026968 | RCOXMOQVMZTXOO-UHFFFAOYSA-N | 545936 |
| GRR.86 | MOL005298 | 5-heptadec-12-enylresorcinol | 3.290743257 | 346.610 | 0.3225 | KDUIMXINOLVPCT-AATRIKPKSA-N | 5318012 |
| GRR.87 | MOL005299 | 5-methyl-tetradecane | 16.15325318 | 212.470 | 0.046466 | SQKZZFHVQCSUHZ-OAHLLOKOSA-N | Not Available |
| GRR.88 | MOL005300 | 6'-Malonylginsenoside Rd1 | 6.70401535 | 1195.510 | 0.026846 | NTYAVUNEPXGZQJ-JPOZCNCBSA-N | Not Available |
| GRR.89 | MOL005301 | 6'-Malonylginsenoside Rd1_qt1 | 29.69203747 | 460.820 | 0.77247 | PYXFVCFISTUSOO-BFYBINCJSA-N | Not Available |
| GRR.90 | MOL005302 | 7-(beta-Xylosyl)cephalomannine | 27.32561845 | 962.150 | 0.17471 | ZVXASUNIAXYESJ-CPMIRZSNSA-N | Not Available |
| GRR.91 | MOL005303 | 7-Tetradecyne | 20.06596267 | 194.400 | 0.039931 | AFNWSIIBAYUTTL-UHFFFAOYSA-N | 141979 |
| GRR.92 | MOL005304 | 7alpha-L-Rhamnosyl-6-methoxylutcolin | 15.0296892 | 462.440 | 0.78679 | UXCXDWDJBSJZOU-IOHFVGAQSA-N | Not Available |
| GRR.93 | MOL005305 | Nepetin | 26.75038273 | 316.280 | 0.30835 | FHHSEFRSDKWJKJ-UHFFFAOYSA-N | 5317284 |
| GRR.94 | MOL005306 | Acetal | 26.39611428 | 118.200 | 0.0087235 | DHKHKXVYLBGOIT-UHFFFAOYSA-N | 7765 |
| GRR.95 | MOL005307 | Adenosine triphosphate | 8.229794672 | 491.220 | 0.5781 | VKYOVEAZNCJNGQ-HUEBKHCJSA-N | Not Available |
| GRR.96 | MOL005308 | Aposiopolamine | 66.64690713 | 271.340 | 0.21999 | UPWMWFSEBOFTNA-SBAKFKMJSA-N | Not Available |
| GRR.97 | MOL005309 | Araloside A | 16.96367721 | 927.210 | 0.063469 | KQSFNXMDCOFFGW-OUFZWZSTSA-N | Not Available |
| GRR.98 | MOL005310 | (4aS,6aR,6aS,6bR,8aR,10S,12aR,14bR)-10-hydroxy-2,2,6a,6b,9,9,12a-heptamethyl-1,3,4,5,6,6a,7,8,8a,10,11,12,13,14b-tetradecahydropicene-4a-carboxylic acid | 14.35588597 | 456.780 | 0.7559 | MIJYXULNPSFWEK-JZQYXDLISA-N | 7048528 |
| GRR.99 | MOL005311 | Argininyl-fructosyl-glucose | 0.740573214 | 498.560 | 0.61876 | NLEHZOMZLCBMKF-GOYIYKMHSA-N | Not Available |
| GRR.100 | MOL005312 | Argininyl-fructosyl-glucose_qt | 11.25045275 | 336.400 | 0.21957 | DLFIWFDONDYZKI-BSPPWYPYSA-N | Not Available |
| GRR.101 | MOL005313 | 5-[(3aS,6R,6aR)-2-keto-1,3,3a,4,6,6a-hexahydrothieno[3,4-d]imidazol-6-yl]valeric acid | 75.7530285 | 244.350 | 0.095535 | YBJHBAHKTGYVGT-ZXFLCMHBSA-N | 5315463 |
| GRR.102 | MOL005314 | Celabenzine | 101.8825954 | 379.550 | 0.48772 | LSYKFBZWBDMZLQ-OAQYLSRUSA-N | Not Available |
| GRR.103 | MOL005315 | (R)-()-Citronellal | 50.77851548 | 154.280 | 0.020405 | NEHNMFOYXAPHSD-SNVBAGLBSA-N | 75427 |
| GRR.104 | MOL005316 | MAV | 56.17201459 | 194.160 | 0.055106 | AEMOLEFTQBMNLQ-BYHBOUFCSA-N | 657117 |
| GRR.105 | MOL005317 | Deoxyharringtonine | 39.27443988 | 515.660 | 0.8116 | WRCBXHDQHPUVHW-CDRKEARJSA-N | Not Available |
| GRR.106 | MOL005318 | Dianthramine | 40.44641187 | 289.260 | 0.19676 | SVZLTRRSGWXPBL-UHFFFAOYSA-N | 441562 |
| GRR.107 | MOL005319 | Ditertbutyl phthalate | 43.66867352 | 278.380 | 0.12938 | RYCNBIYTZSGSPI-UHFFFAOYSA-N | 121712 |
| GRR.108 | MOL005320 | arachidonate | 45.57324991 | 304.520 | 0.20491 | YZXBAPSDXZZRGB-CGRWFSSPSA-N | 5312542 |
| GRR.109 | MOL005321 | Frutinone A | 65.9037307 | 264.240 | 0.34184 | RFWULRHBGYKEEZ-UHFFFAOYSA-N | 441965 |
| GRR.110 | MOL005322 | Gamma-Selinene | 22.58083514 | 204.390 | 0.08087 | RMZHSBMIZBMVMN-HUUCEWRRSA-N | Not Available |
| GRR.111 | MOL005323 | ginsenoside La | 17.73740163 | 783.120 | 0.13863 | CYUTWBUOPDPXJL-FHZWBSINSA-N | Not Available |
| GRR.112 | MOL005324 | ginsenoside La_qt | 15.70290488 | 458.800 | 0.77942 | XSFXFJMBHIGYOZ-KMPFTHCMSA-N | Not Available |
| GRR.113 | MOL005325 | ginsenoside Ro | 1.981216791 | 957.240 | 0.054963 | NFZYDZXHKFHPGA-UPULUGQVSA-N | Not Available |
| GRR.114 | MOL005326 | Ginsenoside-Ra0 | 7.297044588 | 1271.620 | 0.014792 | LDIAQNKCRRXZCD-UJQRMWHLSA-N | Not Available |
| GRR.115 | MOL005327 | Gypnoside V_qt | 29.69203747 | 460.820 | 0.77045 | PYXFVCFISTUSOO-XPRAATSESA-N | Not Available |
| GRR.116 | MOL005328 | Ginsenoside-Ra1 | 7.089457445 | 1211.560 | 0.018342 | KVMXBSSOCCPAOR-KHMVMXOBSA-N | Not Available |
| GRR.117 | MOL005329 | Ginsenoside-Ra2 | 7.621579779 | 1211.560 | 0.018521 | UEBIBJSWHIZNCA-MUXWUWCSSA-N | Not Available |
| GRR.118 | MOL005330 | Ginsenoside-Ra3 | 7.186741103 | 1241.590 | 0.017309 | QUNSGRLNZDSQJC-AYBHOFBPSA-N | Not Available |
| GRR.119 | MOL005331 | ginsenoside Rb1 | 6.236328734 | 1109.460 | 0.038125 | GZYPWOGIYAIIPV-MPBJDOIPSA-N | Not Available |
| GRR.120 | MOL005332 | (3R,5R,8R,9R,10R,12R,13R,14R,17S)-17-[(2S)-2-hydroxy-6-methylhept-5-en-2-yl]-4,4,8,10,14-pentamethyl-2,3,5,6,7,9,11,12,13,15,16,17-dodecahydro-1H-cyclopenta[a]phenanthrene-3,12-diol | 29.69203747 | 460.820 | 0.77143 | PYXFVCFISTUSOO-WMQFBVARSA-N | 11048822 |
| GRR.121 | MOL005333 | ginsenoside-Rb2 | 6.020630855 | 1079.430 | 0.043353 | NODILNFGTFIURN-GAAOXUKQSA-N | Not Available |
| GRR.122 | MOL005334 | (3S,5R,8R,9R,10R,12R,13R,14R,17S)-17-[(2S)-2-hydroxy-6-methylhept-5-en-2-yl]-4,4,8,10,14-pentamethyl-2,3,5,6,7,9,11,12,13,15,16,17-dodecahydro-1H-cyclopenta[a]phenanthrene-3,12-diol | 29.69203747 | 460.820 | 0.77048 | PYXFVCFISTUSOO-HKUCOEKDSA-N | 11213350 |
| GRR.123 | MOL005335 | Gypenoside LXIX | 7.725880742 | 1079.430 | 0.043345 | NODILNFGTFIURN-FCUDRQNNSA-N | Not Available |
| GRR.124 | MOL005336 | ginsenoside-Rc | 8.159301851 | 1079.430 | 0.043559 | JDCPEKQWFDWQLI-ZCKGZTPXSA-N | Not Available |
| GRR.125 | MOL005337 | (2S,3R,4S,5S,6R)-2-[(2S)-2-[(3S,5R,8R,9R,10R,12R,13R,14R,17S)-3-[(2R,3R,4S,5S,6R)-4,5-dihydroxy-6-(hydroxymethyl)-3-[(2S,3R,4S,5S,6R)-3,4,5-trihydroxy-6-(hydroxymethyl)oxan-2-yl]oxyoxan-2-yl]oxy-12-hydroxy-4,4,8,10,14-pentamethyl-2,3,5,6,7,9,11,12,13,15,1 | 5.499218806 | 947.300 | 0.094758 | RLDVZILFNVRJTL-IWFVLDDISA-N | 11679800 |
| GRR.126 | MOL005338 | Ginsenoside Re | 4.272725573 | 947.300 | 0.12287 | PWAOOJDMFUQOKB-WCZZMFLVSA-N | 441921 |
| GRR.127 | MOL005340 | (3S,5R,6S,8R,9R,10R,12R,13R,14R,17S)-17-[(2R)-2-hydroxy-6-methylhept-5-en-2-yl]-4,4,8,10,14-pentamethyl-2,3,5,6,7,9,11,12,13,15,16,17-dodecahydro-1H-cyclopenta[a]phenanthrene-3,6,12-triol | 20.12704418 | 476.820 | 0.77985 | SHCBCKBYTHZQGZ-DLHMIPLTSA-N | 9847853 |
| GRR.128 | MOL005341 | Sanchinoside C1 | 10.04245744 | 801.140 | 0.27807 | YURJSTAIMNSZAE-HHNZYBFYSA-N | 441923 |
| GRR.129 | MOL005342 | Ginsenoside-Rg3 | 17.7459805 | 785.140 | 0.21625 | RWXIFXNRCLMQCD-DRJVPZJKSA-N | Not Available |
| GRR.130 | MOL005343 | Ginsenoside-Rg3_qt | 29.69203747 | 460.820 | 0.77244 | PYXFVCFISTUSOO-HHYRSGPWSA-N | Not Available |
| GRR.131 | MOL005344 | ginsenoside rh2 | 36.31951162 | 622.980 | 0.55868 | CKUVNOCSBYYHIS-IRFFNABBSA-N | 119307 |
| GRR.132 | MOL005345 | (2R,3S,4S,5R,6R)-2-(hydroxymethyl)-6-[[(3S,5R,8R,9R,10R,12R,13R,14R,17S)-12-hydroxy-4,4,8,10,14-pentamethyl-17-[(2Z)-6-methylhepta-2,5-dien-2-yl]-2,3,5,6,7,9,11,12,13,15,16,17-dodecahydro-1H-cyclopenta[a]phenanthren-3-yl]oxy]oxane-3,4,5-triol | 12.08620622 | 604.960 | 0.58836 | PHLXREOMFNVWOH-YAGNRYSRSA-N | 20839223 |
| GRR.133 | MOL005346 | Ginsenoside-Rh3_qt | 13.09121634 | 442.800 | 0.76447 | OURRZLJECNEIMI-MQCBTYGISA-N | Not Available |
| GRR.134 | MOL005347 | Ginsenoside-Rh4 | 5.218347545 | 620.960 | 0.59893 | OZTXYFOXQFKYRP-IKDRHPKYSA-N | Not Available |
| GRR.135 | MOL005348 | Ginsenoside-Rh4_qt | 31.11214828 | 458.800 | 0.77829 | JKPOYAJYRYOGBN-ARWCPIPWSA-N | Not Available |
| GRR.136 | MOL005349 | Ginsenoside-Rs1 | 6.266311789 | 1121.470 | 0.03995 | ZCVPBYMQBJPBCT-WZCOPVJESA-N | Not Available |
| GRR.137 | MOL005350 | Ginsenoside-Rs2 | 8.139898697 | 1121.470 | 0.040138 | YLMBQJRKOKVUCP-QVKAQDGOSA-N | Not Available |
| GRR.138 | MOL005351 | Ginsenoyne A | 66.21860082 | 258.390 | 0.13444 | FTXZFRIHQNXZNH-YESZJQIVSA-N | Not Available |
| GRR.139 | MOL005352 | Ginsenoyne B | 39.78780676 | 294.850 | 0.12668 | MORPELUWUARUFU-ULQDDVLXSA-N | Not Available |
| GRR.140 | MOL005353 | Ginsenoyne C | 43.38104405 | 276.410 | 0.13166 | WNVDKDQMWFSCPI-ULQDDVLXSA-N | Not Available |
| GRR.141 | MOL005354 | Ginsenoyne D | 19.32146266 | 262.430 | 0.12903 | WDZQEROINMBCOK-YESZJQIVSA-N | Not Available |
| GRR.142 | MOL005355 | Ginsenoyne E | 36.52750425 | 258.390 | 0.13269 | WIONCQLWGYLTME-DLBZAZTESA-N | 5317636 |
| GRR.143 | MOL005356 | Girinimbin | 61.21530251 | 263.360 | 0.31484 | GAEQWKVGMHUUKO-UHFFFAOYSA-N | 96943 |
| GRR.144 | MOL005357 | Gomisin B | 31.99042428 | 514.620 | 0.82858 | XDVOVYYAPHHHBE-YGIMCSHQSA-N | Not Available |
| GRR.145 | MOL005358 | L-erythro-isocitric acid | 32.94958887 | 192.140 | 0.044297 | ODBLHEXUDAPZAU-VVJJHMBFSA-N | 439238 |
| GRR.146 | MOL005359 | D-erythro-Isocitric acid | 65.42895741 | 192.140 | 0.044308 | ODBLHEXUDAPZAU-OKKQSCSOSA-N | 447805 |
| GRR.147 | MOL005360 | malkangunin | 57.71384384 | 432.560 | 0.62642 | DTMIMKTZETWDJV-DUUKBJRLSA-N | Not Available |
| GRR.148 | MOL005361 | Malonylginsenoside Rc | 7.841298739 | 1165.480 | 0.030597 | UOFHLCPZXZURFL-RJBDEUDKSA-N | Not Available |
| GRR.149 | MOL005362 | Malonylginsenoside Rc_qt1 | 29.69203747 | 460.820 | 0.77223 | PYXFVCFISTUSOO-SPPDXTJESA-N | Not Available |
| GRR.150 | MOL005363 | Malonylginsenoside Rd | 8.836860516 | 1033.350 | 0.065535 | OSXWNRAKZUNVDR-HUOLQKEOSA-N | Not Available |
| GRR.151 | MOL005364 | Malonylginsenoside Rd_qt | 29.69203747 | 460.820 | 0.77066 | PYXFVCFISTUSOO-KQSXEGFZSA-N | Not Available |
| GRR.152 | MOL005365 | MAL | 1.799318313 | 342.340 | 0.24313 | GUBGYTABKSRVRQ-QUYVBRFLSA-N | 6255 |
| GRR.153 | MOL005366 | Malvic acid | 30.98846042 | 280.500 | 0.15423 | HPSSZFFAYWBIPY-UHFFFAOYSA-N | 10416 |
| GRR.154 | MOL005367 | GUP | 43.04274895 | 180.180 | 0.044671 | WQZGKKKJIJFFOK-RWOPYEJCSA-N | 439680 |
| GRR.155 | MOL005368 | Methyl tricosanoate | 14.60931994 | 368.720 | 0.32985 | VORKGRIRMPBCCZ-UHFFFAOYSA-N | 75519 |
| GRR.156 | MOL005369 | Mycosinol | 82.12398216 | 214.230 | 0.094081 | FZRGCIPZQGXDCM-OAIDTJHVSA-N | Not Available |
| GRR.157 | MOL005370 | NN-Dimethyldecanamide | 55.49706298 | 199.380 | 0.041167 | HNXNKTMIVROLTK-UHFFFAOYSA-N | 26690 |
| GRR.158 | MOL005371 | Nonacosanediol-6,8 | 17.79057562 | 188.350 | 0.031056 | IAABKQHJKGRFAU-MNOVXSKESA-N | Not Available |
| GRR.159 | MOL005372 | notoginsenoside R2 | 17.73699322 | 771.110 | 0.27685 | FNIRVWPHRMMRQI-CVTXWOHCSA-N | Not Available |
| GRR.160 | MOL005373 | notoginsenoside R2_qt | 20.12704418 | 476.820 | 0.78292 | SHCBCKBYTHZQGZ-AELBPGHMSA-N | Not Available |
| GRR.161 | MOL005374 | Notoginsenoside R6 | 4.702837797 | 963.300 | 0.11605 | YPUHYSBFIMWSEC-FSMABQBOSA-N | Not Available |
| GRR.162 | MOL005375 | Stearyl acetate | 16.26634578 | 312.600 | 0.19391 | OIZXRZCQJDXPFO-UHFFFAOYSA-N | 69968 |
| GRR.163 | MOL005376 | Panaxadiol | 33.0879606 | 460.820 | 0.79404 | PVLHOJXLNBFHDX-BMTJSWHCSA-N | Not Available |
| GRR.164 | MOL005378 | Panaxytriol | 33.75825582 | 278.430 | 0.12899 | RDIMTXDFGHNINN-IXDOHACOSA-N | Not Available |
| GRR.165 | MOL005379 | Pancratistatin | 13.13439279 | 325.300 | 0.45995 | VREZDOWOLGNDPW-ALTGWBOUSA-N | 441597 |
| GRR.166 | MOL005380 | Pandamine | 16.14730319 | 552.790 | 0.79075 | STKZKAJIJHJDCQ-HPKJDOSXSA-N | Not Available |
| GRR.167 | MOL005381 | 2-Formylpyrrole | 41.58148546 | 95.110 | 0.010115 | ZSKGQVFRTSEPJT-UHFFFAOYSA-N | 13854 |
| GRR.168 | MOL005382 | Ramalic acid | 5.997082178 | 346.360 | 0.29665 | GEZCJRBINSDUSC-UHFFFAOYSA-N | 5320886 |
| GRR.169 | MOL005383 | Methylselenocysteine | 35.73551127 | 182.100 | 0.010179 | XDSSPSLGNGIIHP-VKHMYHEASA-N | 45266674 |
| GRR.170 | MOL005384 | suchilactone | 57.51882425 | 368.410 | 0.55573 | GVNUFBXIXQNOCF-CRNMQVKPSA-N | Not Available |
| GRR.171 | MOL005385 | Suffruticoside A_qt1 | 13.90144674 | 302.260 | 0.19873 | RHHKXYIFKCWYMH-MVWJERBFSA-N | Not Available |
| GRR.172 | MOL005386 | Vulgarin | 29.21124048 | 264.350 | 0.1974 | NGPDZEACIWDCKX-WUDKWMPASA-N | 94253 |
| GRR.173 | MOL005388 | Undecane, 3,6-dimethyl | 12.84635739 | 184.410 | 0.02896 | WLUQEGDKTQZXBV-QWHCGFSZSA-N | Not Available |
| GRR.174 | MOL005389 | PANGAMIC ACID | 10.07720283 | 436.620 | 0.31928 | RVSTWRHIGKXTLG-WCXIOVBPSA-N | 83182 |
| GRR.175 | MOL005390 | 3-[[(2S)-2,4-dihydroxy-3,3-dimethylbutanoyl]amino]propanoic acid | 21.29315424 | 219.270 | 0.060324 | GHOKWGTUZJEAQD-SSDOTTSWSA-N | 5748353 |
| GRR.176 | MOL005391 | (Z,Z)-alpha-farnesene | 8.469192417 | 204.390 | 0.047295 | CXENHBSYCFFKJS-LOQWIJHWSA-N | 5317320 |
| GRR.177 | MOL005392 | alpha-Guttiferin | 4.425802197 | 452.590 | 0.64929 | MJJZVOIXKVUBQD-YISVQYQQSA-N | Not Available |
| GRR.178 | MOL005394 | (Z)-2-methyl-5-[(1S,2R,4R)-2-methyl-3-methylene-2-norbornanyl]pent-2-en-1-ol | 35.28336521 | 220.390 | 0.088125 | OJYKYCDSGQGTRJ-GQYWAMEOSA-N | 6857681 |
| GRR.179 | MOL005396 | cis-Widdrol alpha-epoxide | 69.04305505 | 238.410 | 0.14728 | BSLAWKTUBVGEDH-UNQGMJICSA-N | Not Available |
| GRR.180 | MOL005397 | Dammarane | 19.73422157 | 414.840 | 0.70137 | OORMXZNMRWBSTK-XYCZMWNLSA-N | 3036917 |
| GRR.181 | MOL005398 | alexandrin | 20.63193686 | 576.950 | 0.62713 | NPJICTMALKLTFW-FYZSWKHYSA-N | Not Available |
| GRR.182 | MOL005399 | alexandrin_qt | 36.91390583 | 414.790 | 0.75268 | KZJWDPNRJALLNS-HEEOYUHISA-N | Not Available |
| GRR.183 | MOL005400 | ginsenoside Rg5 | 6.147407463 | 767.120 | 0.22766 | VNVVACHTDGQAHA-DONNJNOYSA-N | Not Available |
| GRR.184 | MOL005401 | ginsenoside Rg5_qt | 39.56307142 | 442.800 | 0.78506 | FCMJTYFZRUKOPU-FFFKMGBISA-N | Not Available |
| GRR.185 | MOL005402 | Methyl margarate | 17.40884666 | 284.540 | 0.13808 | HUEBIMLTDXKIPR-UHFFFAOYSA-N | 15609 |
| GRR.186 | MOL005403 | oleanane | 6.690722227 | 412.820 | 0.75839 | VCNKUCWWHVTTBY-YTMGNKDFSA-N | Not Available |
| GRR.187 | MOL005404 | p-Glucosyloxymandelonitrile | 12.11574154 | 298.370 | 0.18426 | PXWNPQIYWRFPGW-ILQXODQXSA-N | Not Available |
| GRR.188 | MOL006651 | Trifolirhizin | 7.624142413 | 446.440 | 0.78562 | VGSYCWGXBYZLLE-QEEQPWONSA-N | Not Available |
| GRR.189 | MOL007500 | panaxatriol | 15.41984471 | 476.820 | 0.79324 | QFJUYMMIBFBOJY-UXZRXANASA-N | 73599 |
| GRR.190 | MOL011400 | ginsenoside rf | 17.74107778 | 801.140 | 0.24146 | UZIOUZHBUYLDHW-XUBRWZAZSA-N | 441922 |
| PR.1 | MOL000033 | (3S,8S,9S,10R,13R,14S,17R)-10,13-dimethyl-17-[(2R,5S)-5-propan-2-yloctan-2-yl]-2,3,4,7,8,9,11,12,14,15,16,17-dodecahydro-1H-cyclopenta[a]phenanthren-3-ol | 36.22847056 | 428.820 | 0.78288 | KLEXDBGYSOIREE-UIFQYPGESA-N | 15976101 |
| PR.2 | MOL000054 | L- | 47.64200167 | 174.240 | 0.031878 | ODKSFYDXXFIFQN-BYPYZUCNSA-N | 28782 |
| PR.3 | MOL000061 | Prolinum | 77.57468129 | 115.150 | 0.014161 | ONIBWKKTOPOVIA-BYPYZUCNSA-N | 6971047 |
| PR.4 | MOL000069 | palmitic acid | 19.2965647 | 256.480 | 0.098573 | IPCSVZSSVZVIGE-UHFFFAOYSA-N | 985 |
| PR.5 | MOL000098 | quercetin | 46.43334812 | 302.250 | 0.27525 | REFJWTPEDVJJIY-UHFFFAOYSA-N | 5280343 |
| PR.6 | MOL000114 | vanillic acid | 35.47235319 | 168.160 | 0.040917 | WKOLLVMJNQIZCI-UHFFFAOYSA-N | 8468 |
| PR.7 | MOL000131 | EIC | 41.90443602 | 280.500 | 0.14347 | OYHQOLUKZRVURQ-HZJYTTRNSA-N | 5280450 |
| PR.8 | MOL000211 | Mairin | 55.37707338 | 456.780 | 0.7761 | QGJZLNKBHJESQX-FZFNOLFKSA-N | 64971 |
| PR.9 | MOL000239 | Jaranol | 50.82881677 | 314.310 | 0.29148 | BJBUTJQYZDYRMJ-UHFFFAOYSA-N | 5318869 |
| PR.10 | MOL000251 | Rhamnocitrin | 12.89911753 | 300.280 | 0.26607 | MQSZRBPYXNEFHF-UHFFFAOYSA-N | 5320946 |
| PR.11 | MOL000295 | alexandrin | 20.63193686 | 576.950 | 0.62697 | NPJICTMALKLTFW-LJBLLCJFSA-N | Not Available |
| PR.12 | MOL000296 | hederagenin | 36.91390583 | 414.790 | 0.75072 | KZJWDPNRJALLNS-CQXWNKEUSA-N | Not Available |
| PR.13 | MOL000354 | isorhamnetin | 49.60437705 | 316.280 | 0.306 | IZQSVPBOUDKVDZ-UHFFFAOYSA-N | 5281654 |
| PR.14 | MOL000356 | lupeol | 12.12076413 | 426.800 | 0.77716 | MQYXUWHLBZFQQO-QGTGJCAVSA-N | 259846 |
| PR.15 | MOL000371 | 3,9-di-O-methylnissolin | 53.74152673 | 314.360 | 0.47573 | RFFNFQZKHNKOPO-BBRMVZONSA-N | 15689655 |
| PR.16 | MOL000372 | 3-Hydroxy-2-picoline | 62.47266029 | 109.140 | 0.015684 | AQSRRZGQRFFFGS-UHFFFAOYSA-N | 70719 |
| PR.17 | MOL000373 | (2S)-4-methoxy-7-methyl-2-[1-methyl-1-[(2S,3R,4S,5S,6R)-3,4,5-trihydroxy-6-methylol-tetrahydropyran-2-yl]oxy-ethyl]-2,3-dihydrofuro[3,2-g]chromen-5-one | 5.377531958 | 452.500 | 0.80846 | QVGFPTYGKPLXPK-OOBAEQHESA-N | 21670038 |
| PR.18 | MOL000374 | 5'-hydroxyiso-muronulatol-2',5'-di-O-glucoside | 41.71766574 | 642.670 | 0.69251 | SRVGYVIWVOOXQO-FQRJZKGRSA-N | Not Available |
| PR.19 | MOL000375 | 5'-hydroxyiso-muronulatol-2',5'-di-O-glucoside_qt | 3.652901822 | 480.510 | 0.80196 | LBCJEVRJKNFCRZ-MDOOIGBLSA-N | Not Available |
| PR.20 | MOL000376 | 7,2'-dihydroxy-3',4'-dimethoxyisoflavone-7-O-β-D-glucoside | 16.15920535 | 476.470 | 0.85522 | LQEMSOXERJRDFG-JTLUYSSBSA-N | 46899140 |
| PR.21 | MOL000377 | 7-hydroxy-3-(2-hydroxy-3,4-dimethoxy-phenyl)chromone | 5.446255355 | 314.310 | 0.3025 | NBYYPWKEQRNADF-UHFFFAOYSA-N | 5359024 |
| PR.22 | MOL000378 | 7-O-methylisomucronulatol | 74.68613752 | 316.380 | 0.29792 | BLHQCBJSTMDZQA-LBPRGKRZSA-N | 15689652 |
| PR.23 | MOL000379 | 9,10-dimethoxypterocarpan-3-O-β-D-glucoside | 36.73668801 | 462.490 | 0.9243 | PCIXSTFFMHVOMF-PBGSHFJYSA-N | Not Available |
| PR.24 | MOL000380 | (6aR,11aR)-9,10-dimethoxy-6a,11a-dihydro-6H-benzofurano[3,2-c]chromen-3-ol | 64.25545452 | 300.330 | 0.42486 | UOVGCLXUTLXAEC-WFASDCNBSA-N | 14077830 |
| PR.25 | MOL000381 | 13-hydroxy-9,11-octadecadienoic acid | 35.59672144 | 296.500 | 0.16728 | HNICUWMFWZBIFP-FSQPMHOZSA-N | 21159006 |
| PR.26 | MOL000382 | Arabinose,d | 1.87147918 | 150.150 | 0.022118 | PYMYPHUHKUWMLA-WDCZJNDASA-N | 66308 |
| PR.27 | MOL000383 | D-Galacturonic acid, homopolymer | 29.7522627 | 194.160 | 0.044648 | IAJILQKETJEXLJ-RSJOWCBRSA-N | 84740 |
| PR.28 | MOL000384 | DL-Glucuronic acid | 3.351961332 | 194.160 | 0.044666 | IAJILQKETJEXLJ-QTBDOELSSA-N | 65041 |
| PR.29 | MOL000386 | Fucopyranose, L- | 42.51087068 | 164.180 | 0.027276 | PNNNRSAQSRJVSB-KCDKBNATSA-N | 3034656 |
| PR.30 | MOL000387 | Bifendate | 31.09782391 | 418.380 | 0.66553 | JMZOMFYRADAWOG-UHFFFAOYSA-N | 108213 |
| PR.31 | MOL000388 | gamma-aminobutyric acid | 24.08906813 | 103.140 | 0.0070086 | BTCSSZJGUNDROE-UHFFFAOYSA-N | 6992099 |
| PR.32 | MOL000389 | FERULIC ACID (CIS) | 54.96547685 | 194.200 | 0.058005 | KSEBMYQBYZTDHS-HYXAFXHYSA-N | 1548883 |
| PR.33 | MOL000390 | daidzein | 19.44106266 | 254.250 | 0.18694 | ZQSIJRDFPHDXIC-UHFFFAOYSA-N | 5281708 |
| PR.34 | MOL000391 | Ononin | 11.52205649 | 430.440 | 0.7756 | MGJLSBDCWOSMHL-MIUGBVLSSA-N | 442813 |
| PR.35 | MOL000392 | formononetin | 69.67388061 | 268.280 | 0.21202 | HKQYGTCOTHHOMP-UHFFFAOYSA-N | 5280378 |
| PR.36 | MOL000393 | Soyasaponin I | 2.055317878 | 943.260 | 0.051908 | PTDAHAWQAGSZDD-IOVCITQVSA-N | 122097 |
| PR.37 | MOL000394 | choline | 0.474664771 | 104.200 | 0.0087188 | GDPPXFUBIJJIKR-UHFFFAOYSA-N | 305 |
| PR.38 | MOL000395 | GGB | 54.95155671 | 176.210 | 0.033506 | FSBIGDSBMBYOPN-VKHMYHEASA-N | 46224610 |
| PR.39 | MOL000396 | (+)-Syringaresinol | 3.291713283 | 418.480 | 0.72244 | KOWMJRJXZMEZLD-HCIHMXRSSA-N | 443023 |
| PR.40 | MOL000397 | cis-p-Coumarate | 45.98222688 | 164.170 | 0.039073 | NGSWKAQJJWESNS-UTCJRWHESA-N | 1549106 |
| PR.41 | MOL000398 | isoflavanone | 109.9866565 | 316.330 | 0.29572 | JNSVNRWHSLLCBG-LLVKDONJSA-N | Not Available |
| PR.42 | MOL000399 | Docosanoate | 15.68844863 | 340.660 | 0.26054 | UKMSUNONTOPOIO-UHFFFAOYSA-N | 8215 |
| PR.43 | MOL000400 | Flavaxin | 18.18360547 | 376.410 | 0.4997 | AUNGANRZJHBGPY-MBNYWOFBSA-N | 6759 |
| PR.44 | MOL000401 | astragalosideI | 46.79290415 | 869.170 | 0.10777 | KXHCYYSIAXMSPA-RRNPXHQQSA-N | Not Available |
| PR.45 | MOL000402 | astragalosideI_qt | 12.34383193 | 707.010 | 0.20385 | HAUCZONIQZLEEW-SQCLOWIESA-N | Not Available |
| PR.46 | MOL000403 | astragalosideII | 46.05958281 | 827.130 | 0.12588 | AYWNHWGQTMCQIV-IRBWRUHMSA-N | Not Available |
| PR.47 | MOL000404 | astragalosideII_qt | 11.54941535 | 664.970 | 0.24848 | KILZIJKCZIGLFJ-QOICHAAMSA-N | Not Available |
| PR.48 | MOL000405 | astragalosideⅢ | 31.82878056 | 785.090 | 0.095752 | FVFSMBDVZVUETN-ADMPTBELSA-N | Not Available |
| PR.49 | MOL000406 | astragalosideⅢ_qt | 5.351776892 | 622.930 | 0.31537 | VXHVFDQYSSFKAR-ZNDWJJNASA-N | Not Available |
| PR.50 | MOL000407 | astragalosideⅣ | 22.50297823 | 785.090 | 0.15118 | QMNWISYXSJWHRY-IZPZSTDWSA-N | Not Available |
| PR.51 | MOL000408 | astragalosideⅣ_qt | 7.072842708 | 622.930 | 0.31535 | VXHVFDQYSSFKAR-VOVRKYFDSA-N | Not Available |
| PR.52 | MOL000409 | AstragalosideIV | 17.74148629 | 785.090 | 0.15123 | QMNWISYXSJWHRY-DIDCDVCNSA-N | Not Available |
| PR.53 | MOL000410 | AstragalosideIV_qt | 7.072842708 | 622.930 | 0.31558 | VXHVFDQYSSFKAR-SLFWFADNSA-N | Not Available |
| PR.54 | MOL000411 | Astraisoflavanin | 18.37130469 | 464.510 | 0.85623 | ABIQOWLHYABFIJ-IMVNFGOTSA-N | 131420 |
| PR.55 | MOL000412 | Mucronulatol | 4.215732055 | 302.350 | 0.26462 | NUNFZNIXYWTZMW-LLVKDONJSA-N | 442811 |
| PR.56 | MOL000413 | astrachrysoside A | 24.55274198 | 769.090 | 0.10329 | LZRSXNSZMFQNOA-JSDRKFJJSA-N | Not Available |
| PR.57 | MOL000414 | Caffeate | 54.97053959 | 180.170 | 0.050036 | QAIPRVGONGVQAS-RQOWECAXSA-N | 1549111 |
| PR.58 | MOL000415 | rutin | 3.201533128 | 610.570 | 0.68283 | IKGXIBQEEMLURG-NVPNHPEKSA-N | 5280805 |
| PR.59 | MOL000416 | Lariciresinol | 5.526192384 | 360.440 | 0.37941 | MHXCIKYXNYCMHY-AUSJPIAWSA-N | 332427 |
| PR.60 | MOL000417 | Calycosin | 47.75182783 | 284.280 | 0.24278 | ZZAJQOPSWWVMBI-UHFFFAOYSA-N | 5280448 |
| PR.61 | MOL000418 | 3'-Hydroxy-4'-methoxyisoflavone-7-O-beta-D-glucoside | 10.0514797 | 446.440 | 0.81027 | WACBUPFEGWUGPB-MIUGBVLSSA-N | 5318267 |
| PR.62 | MOL000419 | astrasieversianin XV | 11.18922482 | 901.220 | 0.066847 | KQSZHSKJNNXOTD-FWLWVOKOSA-N | Not Available |
| PR.63 | MOL000420 | XLS | 51.07636714 | 150.150 | 0.022107 | PYMYPHUHKUWMLA-VPENINKCSA-N | 644160 |
| PR.64 | MOL000421 | nicotinic acid | 47.64529278 | 123.120 | 0.020168 | PVNIIMVLHYAWGP-UHFFFAOYSA-N | 938 |
| PR.65 | MOL000422 | kaempferol | 41.88224954 | 286.250 | 0.24066 | IYRMWMYZSQPJKC-UHFFFAOYSA-N | 5280863 |
| PR.66 | MOL000423 | rhamnocitrin-3-O-glucoside | 2.868950136 | 462.440 | 0.76403 | ULVBHEFDGPIWAT-LFXZADKFSA-N | 14704550 |
| PR.67 | MOL000424 | RAM | 50.49983975 | 164.180 | 0.036875 | SHZGCJCMOBCMKK-HGVZOGFYSA-N | 439710 |
| PR.68 | MOL000425 | asernestioside A | 11.06572823 | 931.250 | 0.032981 | AFDAWVKSWHBRTN-OZBABBFWSA-N | Not Available |
| PR.69 | MOL000426 | asernestioside A_qt | 24.55274198 | 769.090 | 0.1033 | LZRSXNSZMFQNOA-JMMMEHHASA-N | Not Available |
| PR.70 | MOL000427 | asernestioside B | 12.53747269 | 973.290 | 0.030286 | FKYIDLGPGAGAHN-HDFSLNGOSA-N | Not Available |
| PR.71 | MOL000428 | asernestioside B_qt | 14.02555321 | 811.130 | 0.094284 | FHLAVHKZODZFKA-NBMPFHOKSA-N | Not Available |
| PR.72 | MOL000429 | Crystal VI | 83.95759702 | 132.140 | 0.016451 | DCXYFEDJOCDNAF-REOHCLBHSA-N | 6992089 |
| PR.73 | MOL000430 | betaine | 40.92229672 | 117.170 | 0.012606 | GMTCLSZGPOBNLA-UHFFFAOYSA-N | 248 |
| PR.74 | MOL000431 | coumarin | 29.16755329 | 146.150 | 0.043014 | ZYGHJZDHTFUPRJ-UHFFFAOYSA-N | 323 |
| PR.75 | MOL000432 | linolenic acid | 45.00906591 | 278.480 | 0.14709 | DTOSIQBPPRVQHS-PDBXOOCHSA-N | 5280934 |
| PR.76 | MOL000433 | FA | 68.96043622 | 441.450 | 0.7057 | OVBPIULPVIDEAO-LBPRGKRZSA-N | 6037 |
| PR.77 | MOL000434 | acetylastragaloside I | 43.53815288 | 911.210 | 0.094201 | KWZSMZJAHIHRRT-GHWJIYAISA-N | Not Available |
| PR.78 | MOL000435 | acetylastragaloside I_qt | 30.75317786 | 749.050 | 0.17237 | YEXAKIVRCUYYCW-GJWPHNHISA-N | Not Available |
| PR.79 | MOL000436 | (Z)-1-(2,4-dihydroxyphenyl)-3-(4-hydroxyphenyl)prop-2-en-1-one | 87.50845173 | 256.270 | 0.14769 | DXDRHHKMWQZJHT-BAQGIRSFSA-N | 6603886 |
| PR.80 | MOL000437 | Hirsutrin | 1.858078251 | 464.410 | 0.76904 | OVSQVDMCBVZWGM-QSOFNFLRSA-N | 5280804 |
| PR.81 | MOL000438 | (3R)-3-(2-hydroxy-3,4-dimethoxyphenyl)chroman-7-ol | 67.66747949 | 302.350 | 0.26479 | NQRBAPDEZYMKFL-NSHDSACASA-N | 10380176 |
| PR.82 | MOL000439 | isomucronulatol-7,2'-di-O-glucosiole | 49.28105539 | 626.670 | 0.62065 | NHOPAJCVMDIGBN-MEPKZADGSA-N | 15689653 |
| PR.83 | MOL000440 | isomucronulatol-7,2'-di-O-glucosiole_qt | 23.42394286 | 464.510 | 0.79041 | PTMFWXXHLUSFEC-AWAQQNJZSA-N | Not Available |
| PR.84 | MOL000441 | LUPENONE | 11.65681758 | 424.780 | 0.78096 | GRBHNQFQFHLCHO-BHMAJAPKSA-N | 92158 |
| PR.85 | MOL000442 | 1,7-Dihydroxy-3,9-dimethoxy pterocarpene | 39.04541112 | 314.310 | 0.47943 | RVGZSUMTFIEORY-UHFFFAOYSA-N | 5316760 |
| PR.86 | MOL001955 | Heriguard | 11.93273511 | 354.340 | 0.32642 | CWVRJTMFETXNAD-JUHZACGLSA-N | 1794427 |
| PR.87 | MOL005928 | isoferulic acid | 50.82647607 | 194.200 | 0.058232 | QURCVMIEKCOAJU-HWKANZROSA-N | 736186 |
| LF.1 | MOL000040 | Scopoletol | 27.77346082 | 192.180 | 0.076154 | RODXRVNMMDRFIK-UHFFFAOYSA-N | 5280460 |
| LF.2 | MOL000069 | palmitic acid | 19.2965647 | 256.480 | 0.098573 | IPCSVZSSVZVIGE-UHFFFAOYSA-N | 985 |
| LF.3 | MOL000098 | quercetin | 46.43334812 | 302.250 | 0.27525 | REFJWTPEDVJJIY-UHFFFAOYSA-N | 5280343 |
| LF.4 | MOL000131 | EIC | 41.90443602 | 280.500 | 0.14347 | OYHQOLUKZRVURQ-HZJYTTRNSA-N | 5280450 |
| LF.5 | MOL000199 | Safrol | 45.3367099 | 162.200 | 0.047125 | ZMQAAUBTXCXRIC-UHFFFAOYSA-N | 5144 |
| LF.6 | MOL000216 | Scopolin | 22.90867653 | 354.340 | 0.38712 | SGTCGCCQZOUMJJ-ZIRHEVKLSA-N | Not Available |
| LF.7 | MOL000305 | lauric acid | 23.58793922 | 200.360 | 0.043637 | POULHZVOKOAJMA-UHFFFAOYSA-N | 3893 |
| LF.8 | MOL000356 | lupeol | 12.12076413 | 426.800 | 0.77716 | MQYXUWHLBZFQQO-QGTGJCAVSA-N | 259846 |
| LF.9 | MOL000358 | beta-sitosterol | 36.91390583 | 414.790 | 0.75123 | KZJWDPNRJALLNS-VJSFXXLFSA-N | 222284 |
| LF.10 | MOL000361 | Amyrin | 17.60191826 | 426.800 | 0.7633 | JFSHUTJDVKUMTJ-QHPUVITPSA-N | 73145 |
| LF.11 | MOL000388 | gamma-aminobutyric acid | 24.08906813 | 103.140 | 0.0070086 | BTCSSZJGUNDROE-UHFFFAOYSA-N | 6992099 |
| LF.12 | MOL000397 | cis-p-Coumarate | 45.98222688 | 164.170 | 0.039073 | NGSWKAQJJWESNS-UTCJRWHESA-N | 1549106 |
| LF.13 | MOL000415 | rutin | 3.201533128 | 610.570 | 0.68283 | IKGXIBQEEMLURG-NVPNHPEKSA-N | 5280805 |
| LF.14 | MOL000421 | nicotinic acid | 47.64529278 | 123.120 | 0.020168 | PVNIIMVLHYAWGP-UHFFFAOYSA-N | 938 |
| LF.15 | MOL000429 | Crystal VI | 83.95759702 | 132.140 | 0.016451 | DCXYFEDJOCDNAF-REOHCLBHSA-N | 6992089 |
| LF.16 | MOL000430 | betaine | 40.92229672 | 117.170 | 0.012606 | GMTCLSZGPOBNLA-UHFFFAOYSA-N | 248 |
| LF.17 | MOL000449 | Stigmasterol | 43.82985158 | 412.770 | 0.75665 | HCXVJBMSMIARIN-PHZDYDNGSA-N | 5280794 |
| LF.18 | MOL000476 | Physcion | 22.28640405 | 284.280 | 0.26659 | FFWOKTFYGVYKIR-UHFFFAOYSA-N | 10639 |
| LF.19 | MOL000514 | Nonacosane | 8.124095127 | 408.890 | 0.39372 | IGGUPRCHHJZPBS-UHFFFAOYSA-N | 12409 |
| LF.20 | MOL000530 | Lupeol acetate | 9.103261113 | 468.840 | 0.7633 | ODSSDTBFHAYYMD-YOJQYFTNSA-N | 92157 |
| LF.21 | MOL000615 | delta-amorphene | 17.94609655 | 204.390 | 0.077173 | FUCYIEXQVQJBKY-ZFWWWQNUSA-N | 441005 |
| LF.22 | MOL000628 | darutoside | 21.31817387 | 574.930 | 0.63003 | VWDLOXMZIGUBKM-CQJGLIEWSA-N | Not Available |
| LF.23 | MOL000676 | DBP | 64.5416405 | 278.380 | 0.13409 | DOIRQSBPFJWKBE-UHFFFAOYSA-N | 3026 |
| LF.24 | MOL000720 | Safranal | 39.55760595 | 150.240 | 0.035053 | SGAWOGXMMPSZPB-UHFFFAOYSA-N | 61041 |
| LF.25 | MOL000749 | Linoleic | 41.90443602 | 280.500 | 0.14468 | OYHQOLUKZRVURQ-AVQMFFATSA-N | 5282457 |
| LF.26 | MOL000767 | Dotriacontanol | 10.2451896 | 466.980 | 0.45938 | QOEHNLSDMADWEF-UHFFFAOYSA-N | 96117 |
| LF.27 | MOL000864 | MYS | 13.9810568 | 212.470 | 0.04922 | YCOZIPAWZNQLMR-UHFFFAOYSA-N | 12391 |
| LF.28 | MOL000865 | hexadecane | 12.31600907 | 226.500 | 0.060928 | DCAYPVUWAIABOU-UHFFFAOYSA-N | 11006 |
| LF.29 | MOL000867 | Heptadekan | 8.642472374 | 240.530 | 0.074658 | NDJKXXJCMXVBJW-UHFFFAOYSA-N | 12398 |
| LF.30 | MOL000868 | LFA | 8.4605189 | 282.620 | 0.12999 | CBFCDTFDPHXCNY-UHFFFAOYSA-N | 8222 |
| LF.31 | MOL000869 | Henicosane | 8.412905294 | 296.650 | 0.15364 | FNAZRRHPUDJQCJ-UHFFFAOYSA-N | 12403 |
| LF.32 | MOL000874 | paeonol | 28.78723811 | 166.190 | 0.039185 | UILPJVPSNHJFIK-UHFFFAOYSA-N | 11092 |
| LF.33 | MOL000875 | Cedrol | 16.23454047 | 222.410 | 0.12172 | SVURIXNDRWRAFU-OGMFBOKVSA-N | Not Available |
| LF.34 | MOL000878 | Farnesylacetone | 37.84164437 | 262.480 | 0.10214 | LTUMRKDLVGQMJU-IUBLYSDUSA-N | 1711945 |
| LF.35 | MOL000879 | methyl palmitate | 18.08756063 | 270.510 | 0.11594 | FLIACVVOZYBSBS-UHFFFAOYSA-N | 8181 |
| LF.36 | MOL000880 | Tricosane | 8.3304795 | 324.710 | 0.2088 | FIGVVZUWCLSUEI-UHFFFAOYSA-N | 12534 |
| LF.37 | MOL000886 | tetradecane | 15.94226412 | 198.440 | 0.039319 | BGHCVCJVXZWKCC-UHFFFAOYSA-N | 12389 |
| LF.38 | MOL000953 | CLR | 37.87389754 | 386.730 | 0.67677 | HVYWMOMLDIMFJA-DPAQBDIFSA-N | 5997 |
| LF.39 | MOL000971 | Ethylpalmitate | 18.98672237 | 284.540 | 0.13539 | XIRNKXNNONJFQO-UHFFFAOYSA-N | 12366 |
| LF.40 | MOL000983 | n-Triacontanol | 10.45995943 | 438.920 | 0.45146 | REZQBEBOWJAQKS-UHFFFAOYSA-N | 68972 |
| LF.41 | MOL001323 | Sitosterol alpha1 | 43.28127042 | 426.800 | 0.78354 | LPZCCMIISIBREI-JXMPMKKESA-N | 9548595 |
| LF.42 | MOL001393 | myristic acid | 21.18117264 | 228.420 | 0.066784 | TUNFSRHWOTWDNC-UHFFFAOYSA-N | 11005 |
| LF.43 | MOL001394 | Oktadekan | 9.806386361 | 254.560 | 0.090617 | RZJRJXONCZWCBN-UHFFFAOYSA-N | 11635 |
| LF.44 | MOL001396 | PENTADECYLIC ACID | 20.18459315 | 242.450 | 0.081479 | WQEPLUUGTLDZJY-UHFFFAOYSA-N | 13849 |
| LF.45 | MOL001399 | TWT | 8.365694483 | 310.680 | 0.17997 | HOWGUJZVBDQJKV-UHFFFAOYSA-N | 12405 |
| LF.46 | MOL001402 | Octacosane | 8.146611389 | 394.860 | 0.36732 | ZYURHZPYMFLWSH-UHFFFAOYSA-N | 12408 |
| LF.47 | MOL001410 | Zeaxanthin | 21.17385818 | 568.960 | 0.54186 | JKQXZKUSFCKOGQ-QAYBQHTQSA-N | 5280899 |
| LF.48 | MOL001456 | citric acid | 56.2198911 | 192.140 | 0.047066 | KRKNYBCHXYNGOX-UHFFFAOYSA-N | 19782904 |
| LF.49 | MOL001468 | MLT | 59.6169838 | 134.100 | 0.017766 | BJEPYKJPYRNKOW-REOHCLBHSA-N | 222656 |
| LF.50 | MOL001494 | Mandenol | 41.99620045 | 308.560 | 0.19321 | FMMOOAYVCKXGMF-MURFETPASA-N | 5282184 |
| LF.51 | MOL001495 | Ethyl linolenate | 46.10096327 | 306.540 | 0.19716 | JYYFMIOPGOFNPK-XSHSMGBESA-N | 6371716 |
| LF.52 | MOL001496 | ETHYLMYRISTATE | 20.5787978 | 256.480 | 0.094355 | MMKRHZKQPFCLLS-UHFFFAOYSA-N | 31283 |
| LF.53 | MOL001498 | Ethyl stearate | 17.70435054 | 312.600 | 0.18836 | MVLVMROFTAUDAG-UHFFFAOYSA-N | 8122 |
| LF.54 | MOL001585 | 9,17-OCTADECADIENAL (Z) | 38.25691975 | 264.500 | 0.1196 | RXORHYFDDNAOQS-KTKRTIGZSA-N | 5365667 |
| LF.55 | MOL001600 | copaene | 29.47338384 | 204.390 | 0.12401 | VLXDPFLIRFYIME-QRTUWBSPSA-N | Not Available |
| LF.56 | MOL001619 | UPL | 8.520498415 | 268.590 | 0.10901 | LQERIDTXQFOHKA-UHFFFAOYSA-N | 12401 |
| LF.57 | MOL001691 | vitamin c | 13.34350738 | 176.140 | 0.043187 | CIWBSHSKHKDKBQ-JLAZNSOCSA-N | 54670067 |
| LF.58 | MOL001747 | Tetracosane | 8.284074578 | 338.740 | 0.23973 | POOSGDOYLQNASK-UHFFFAOYSA-N | 12592 |
| LF.59 | MOL001752 | EUG | 38.39193486 | 150.190 | 0.030821 | YOMSJEATGXXYPX-UHFFFAOYSA-N | 332 |
| LF.60 | MOL001815 | 1-Tricosene | 16.25211924 | 322.690 | 0.21311 | SJDSOBWGZRPKSB-UHFFFAOYSA-N | 181154 |
| LF.61 | MOL001863 | METHYL ISOPALMITATE | 9.418332099 | 270.510 | 0.11471 | WAKCWJNDXBPEBP-UHFFFAOYSA-N | 21205 |
| LF.62 | MOL001880 | OXL | 29.67836693 | 90.040 | 0.0071888 | MUBZPKHOEPUJKR-UHFFFAOYSA-N | 18676629 |
| LF.63 | MOL001889 | Methyl linolelaidate | 41.93435814 | 294.530 | 0.16791 | WTTJVINHCBCLGX-ZDVGBALWSA-N | 5362793 |
| LF.64 | MOL001901 | 24-Methylenecycloartanol | 10.39704683 | 440.830 | 0.78773 | BDHQMRXFDYJGII-UEBIAWITSA-N | Not Available |
| LF.65 | MOL001979 | LAN | 42.11918897 | 426.800 | 0.74787 | CAHGCLMLTWQZNJ-BQNIITSRSA-N | 246983 |
| LF.66 | MOL002223 | TAU | 24.37137319 | 125.170 | 0.00981 | XOAAWQZATWQOTB-UHFFFAOYSA-N | 4068592 |
| LF.67 | MOL002225 | Styrone | 38.35217514 | 134.190 | 0.022038 | OOCCDEMITAIZTP-QPJJXVBHSA-N | 5315892 |
| LF.68 | MOL002313 | TR-saponin A | 2.672649453 | 1037.330 | 0.062245 | QCMIZNQBGXTSJB-YNAAZIRKSA-N | Not Available |
| LF.69 | MOL002314 | TR-saponin A_qt | 22.62976271 | 715.030 | 0.3183 | XFFHNUNUTSWQFG-CZMNASQASA-N | Not Available |
| LF.70 | MOL002363 | beta-Ionone | 20.63241193 | 192.330 | 0.054165 | PSQYTAPXSHCGMF-BQYQJAHWSA-N | 638014 |
| LF.71 | MOL002376 | PENTACOSANE | 8.249355127 | 352.770 | 0.27262 | YKNWIILGEFFOPE-UHFFFAOYSA-N | 12406 |
| LF.72 | MOL002442 | Cholesteryl ferulate | 22.42538976 | 562.910 | 0.62907 | CPBQNAKTSMCPNH-DCNQEUNQSA-N | Not Available |
| LF.73 | MOL002669 | Campesteryl ferulate | 22.09784892 | 576.940 | 0.59241 | SWIWTAJTJOYCTB-WNUOVGGOSA-N | Not Available |
| LF.74 | MOL002742 | LUT | 15.74236581 | 568.960 | 0.5393 | KBPHJBAIARWVSC-NSIPBSJQSA-N | 448437 |
| LF.75 | MOL002783 | Cumalic acid | 43.09828612 | 140.100 | 0.028014 | ORGPJDKNYMVLFL-UHFFFAOYSA-N | 68141 |
| LF.76 | MOL003126 | alpha cadinene | 18.7279396 | 204.390 | 0.077252 | QMAYBMKBYCGXDH-QLFBSQMISA-N | 12306049 |
| LF.77 | MOL003304 | Hentriacontan | 8.068057784 | 436.950 | 0.50918 | IUJAMGNYPWYUPM-UHFFFAOYSA-N | 12410 |
| LF.78 | MOL003484 | PEY | 25.69685654 | 178.240 | 0.097919 | YNPNZTXNASCQKK-UHFFFAOYSA-N | 995 |
| LF.79 | MOL003501 | (2R,4aS,6S,8aR)-2,6-dimethyldecalin | 46.93318203 | 166.340 | 0.046821 | XNOHNIPVHGINQP-BKUVIOGVSA-N | 21718045 |
| LF.80 | MOL003535 | 1,1,6-trimethyl-2H-naphthalene | 24.93733273 | 172.290 | 0.061599 | RTUMCNDCAVLXEP-UHFFFAOYSA-N | 121677 |
| LF.81 | MOL003547 | Azaron | 38.38574731 | 208.280 | 0.060667 | RKFAZBXYICVSKP-AATRIKPKSA-N | 636822 |
| LF.82 | MOL003578 | Cycloartenol | 38.68565906 | 426.800 | 0.78093 | ONQRKEUAIJMULO-YBXTVTTCSA-N | Not Available |
| LF.83 | MOL003800 | CIR | 52.96024676 | 175.220 | 0.033089 | RHGKLRLOHDJJDR-BYPYZUCNSA-N | 6992098 |
| LF.84 | MOL004498 | 12-O-Nicotinoylisolineolone | 20.70427369 | 469.630 | 0.83406 | OQFVSKGDZKMZEH-WCYOEAGXSA-N | Not Available |
| LF.85 | MOL004708 | δ-cadinol | 14.02619912 | 222.410 | 0.093113 | LHYHMMRYTDARSZ-KBUPBQIOSA-N | 51394521 |
| LF.86 | MOL004766 | Stearyl chloride | 17.73740163 | 289 | 0.108 | VUQPJRPDRDVQMN-UHFFFAOYSA-N | 18815 |
| LF.87 | MOL005224 | TETRATETRACONTANE | 7.817699989 | 619.340 | 0.24686 | KMXFZRSJMDYPPG-UHFFFAOYSA-N | 23494 |
| LF.88 | MOL005406 | atropine | 45.97058178 | 289.410 | 0.19328 | RKUNBYITZUJHSG-QKPAOTATSA-N | 154417 |
| LF.89 | MOL005438 | campesterol | 37.57681789 | 400.760 | 0.71488 | SGNBVLSWZMBQTH-KAASKNFBSA-N | Not Available |
| LF.90 | MOL005439 | Ostreasterol | 13.61538731 | 398.740 | 0.72038 | INDVLXYUCBVVKW-PXBBAZSNSA-N | 92113 |
| LF.91 | MOL005479 | pentatriacont-17-ene | 13.18621037 | 491.050 | 0.43259 | BLCUZCCTSBVFSV-LAPDZXRHSA-N | 5365022 |
| LF.92 | MOL005521 | phytane | 13.86308864 | 282.620 | 0.10838 | GGYKPYDKXLHNTI-VAMGGRTRSA-N | Not Available |
| LF.93 | MOL005528 | 2,6,10,14-tetramethylpentadecane | 3.60206877 | 268.590 | 0.091543 | XOJVVFBFDXDTEG-KDURUIRLSA-N | 25022101 |
| LF.94 | MOL005960 | 9-Octadecyne | 10.70089247 | 250.520 | 0.092219 | NKRBWIXXEQOWRY-UHFFFAOYSA-N | 141998 |
| LF.95 | MOL005961 | 10,13-Octadecadienoic acid, methyl ester | 41.93435814 | 294.530 | 0.16825 | TYEPJNKESLRTEJ-AVQMFFATSA-N | 5365678 |
| LF.96 | MOL006209 | cyanin | 47.42092269 | 411.660 | 0.75918 | ZNWVDIROEWRDJT-DARPEHSRSA-N | Not Available |
| LF.97 | MOL007449 | 24-methylidenelophenol | 44.19264545 | 412.770 | 0.7533 | RSMKYRDCCSNYFM-AAGDOFLISA-N | 5283640 |
| LF.98 | MOL007946 | 19435-97-3 | 33.04075784 | 222.410 | 0.093097 | LHYHMMRYTDARSZ-ZQDZILKHSA-N | 3084311 |
| LF.99 | MOL008159 | TRIACONTANE | 8.090493252 | 422.920 | 0.41499 | JXTPJDDICSTXJX-UHFFFAOYSA-N | 12535 |
| LF.100 | MOL008172 | daucosterol | 3.651514252 | 576.950 | 0.6265 | NPJICTMALKLTFW-FQZOOVCPSA-N | Not Available |
| LF.101 | MOL008173 | daucosterol_qt | 36.91390583 | 414.790 | 0.75316 | KZJWDPNRJALLNS-ZBNQVYKFSA-N | Not Available |
| LF.102 | MOL008400 | glycitein | 50.47891366 | 284.280 | 0.23826 | DXYUAIFZCFRPTH-UHFFFAOYSA-N | 5317750 |
| LF.103 | MOL008739 | Solavetivone | 36.73245879 | 218.370 | 0.086748 | FGCUSSRGQNHZRW-UMVBOHGHSA-N | 442399 |
| LF.104 | MOL009544 | Hypogaeic acid | 35.77585321 | 254.460 | 0.099636 | PJHOFUXBXJNUAC-KTKRTIGZSA-N | 5318393 |
| LF.105 | MOL009601 | 11-DECYLDOCOSANE | 9.27154264 | 450.980 | 0.4015 | ZTLKOYAJKRWSKL-UHFFFAOYSA-N | 41440 |
| LF.106 | MOL009602 | 12-decylheneicosane | 9.325776435 | 436.950 | 0.37206 | WMQSSFYTIFHSFT-HKBQPEDESA-N | Not Available |
| LF.107 | MOL009603 | 13,17,21-trimethyltritriacontane | 10.92370592 | 507.100 | 0.5253 | VPNSAYIYZOPVGL-YQMDEHACSA-N | Not Available |
| LF.108 | MOL009604 | 14b-pregnane | 34.77923299 | 288.570 | 0.33723 | JWMFYGXQPXQEEM-MTUNSFAGSA-N | Not Available |
| LF.109 | MOL009605 | 1,2,3,4,5-Pentamethylcyclopentadiene | 48.69002528 | 136.260 | 0.026351 | WQIQNKQYEUMPBM-UHFFFAOYSA-N | 77667 |
| LF.110 | MOL009606 | (4aS,7R)-7-isopropenyl-1,4a-dimethyl-5,6,7,8-tetrahydronaphthalen-2-one | 35.46418632 | 216.350 | 0.096592 | YFZICPBAKZACEG-DOMZBBRYSA-N | 10976879 |
| LF.111 | MOL009607 | (1R,4aR,5S,8aS)-1,5-dimethyldecalin | 59.44016414 | 166.340 | 0.048564 | FLBCYSDGVKDEEA-IWDIQUIJSA-N | 21717998 |
| LF.112 | MOL009608 | WLN: E6E | 7.571954576 | 243.980 | 0.0066514 | SGRHVVLXEBNBDV-UHFFFAOYSA-N | 12368 |
| LF.113 | MOL009609 | 1,6-dimethyl-1-isopropyl-1,2,3,4,4a,7-hexahydronaphthalene | 39.54485614 | 204.390 | 0.081253 | QZKLOBUKAPLBSE-HIFRSBDPSA-N | Not Available |
| LF.114 | MOL009610 | (1S,4aS,6S,8aR)-1,6-dimethyldecalin | 47.4646191 | 166.340 | 0.047679 | SXLVBBLVYUOSKX-FIQHERPVSA-N | 21718000 |
| LF.115 | MOL009611 | Stearyl iodide | 6.221126037 | 380.450 | 0.10718 | ZNJOCVLVYVOUGB-UHFFFAOYSA-N | 12402 |
| LF.116 | MOL009612 | (24R)-4alpha-Methyl-24-ethylcholesta-7,25-dien-3beta-ylacetate | 46.35749925 | 482.870 | 0.8398 | YXSNMSCMEXMDCO-ZDOZIUNCSA-N | Not Available |
| LF.117 | MOL009613 | 24-Methylcholest-7-en-3belta-ol | 8.790447814 | 400.760 | 0.71546 | PUGBZUWUTZUUCP-SRLXQNGGSA-N | Not Available |
| LF.118 | MOL009614 | 24-Methylcholesta-7,22-dien-3belta-ol | 12.21827793 | 398.740 | 0.72003 | QOXPZVASXWSKKU-QDZFOBFWSA-N | Not Available |
| LF.119 | MOL009615 | 24-Methylenecycloartan-3beta,21-diol | 37.31728162 | 456.830 | 0.79751 | FJXNINQGUTYPNE-DLJSKDEVSA-N | Not Available |
| LF.120 | MOL009616 | 24-Methylenecycloartanol ferulate | 22.03687458 | 617.010 | 0.39148 | JBSUVXVGZSMGDJ-YVMHCORFSA-N | Not Available |
| LF.121 | MOL009617 | 24-ethylcholest-22-enol | 37.09454086 | 414.790 | 0.7511 | CSVWWLUMXNHWSU-YASJBQHGSA-N | Not Available |
| LF.122 | MOL009618 | 24-ethylcholesta-5,22-dienol | 43.82985158 | 412.770 | 0.75636 | HCXVJBMSMIARIN-KRUSZOTOSA-N | Not Available |
| LF.123 | MOL009619 | Fucostanol | 25.32211635 | 416.810 | 0.74678 | LGJMUZUPVCAVPU-HRJGVYIJSA-N | 241572 |
| LF.124 | MOL009620 | 24-methyl-31-norlanost-9(11)-enol | 37.9996853 | 428.820 | 0.75092 | NWPXYRKVJLBSQU-NTJPQSOTSA-N | Not Available |
| LF.125 | MOL009621 | 24-methylenelanost-8-enol | 42.36819868 | 440.830 | 0.76769 | XJLZCPIILZRCPS-MCOFMKOSSA-N | Not Available |
| LF.126 | MOL009622 | Fucosterol | 43.77639556 | 412.770 | 0.75668 | OSELKOCHBMDKEJ-JUGJNGJRSA-N | 5281328 |
| LF.127 | MOL009623 | 2,21-Dimethyldocosane | 11.17737959 | 338.740 | 0.23787 | QQTULUIXVZMZAU-UHFFFAOYSA-N | 537323 |
| LF.128 | MOL009624 | (2S,3S,4aR,8aR)-2,3-dimethyldecalin | 46.48255587 | 166.340 | 0.047492 | UBGLIVPMNDOOTE-NNYUYHANSA-N | 21718043 |
| LF.129 | MOL009625 | 2,6,10,15-tetramethylheptadecane | 13.73377727 | 296.650 | 0.12827 | ZZEQNXPBKOFTBG-NJYVYQBISA-N | Not Available |
| LF.130 | MOL009626 | 2,6,10-trimethyl-hexadecane | 4.028440931 | 268.590 | 0.09265 | MHLZUZMLIFKALI-MOPGFXCFSA-N | Not Available |
| LF.131 | MOL009627 | 2,6,3-trimethyl-Dodecane | 3.84210297 | 212.470 | 0.044371 | TWZSKZFWKMZBHR-CABCVRRESA-N | Not Available |
| LF.132 | MOL009628 | (2-fluoro-2-methoxycyclopropyl)benzene | 62.78270162 | 166.210 | 0.052125 | GCXBCJBAQNROTA-VHSXEESVSA-N | Not Available |
| LF.133 | MOL009629 | 2-methyl-5-ethyloctane | 6.172669153 | 156.350 | 0.017989 | CQCKNPUKBOITAX-LLVKDONJSA-N | Not Available |
| LF.134 | MOL009630 | 2-o-(beta-d-glucopyranosyl)-ascorbic acid | 7.559324696 | 338.300 | 0.2445 | MLSJBGYKDYSOAE-DCWMUDTNSA-N | 54693473 |
| LF.135 | MOL009631 | 31-Norcyclolaudenol | 38.68209614 | 440.830 | 0.81391 | OSKBBWPZDJRQMS-TVCYAWOFSA-N | Not Available |
| LF.136 | MOL009632 | 31-norcycloartanol | 8.91774398 | 414.790 | 0.77995 | RXPPOAQPYLFCAS-RXEJFBRGSA-N | Not Available |
| LF.137 | MOL009633 | 31-norlanost-9(11)-enol | 38.35394137 | 414.790 | 0.7249 | SZCKXGWHINUNKB-SNKUZYNWSA-N | Not Available |
| LF.138 | MOL009634 | 31-norlanosterol | 42.20462055 | 412.770 | 0.73012 | KLZWTHGLLDRKHD-MGAQDEEHSA-N | Not Available |
| LF.139 | MOL009635 | 4,24-methyllophenol | 37.83467433 | 414.790 | 0.74999 | AOQRDALGACAKHI-AHSCMWHKSA-N | Not Available |
| LF.140 | MOL009636 | 4-((2Z,5E)-5-methylhepta-2,5-dien-2-yl)cyclohex-1-ene | 39.79788566 | 190.360 | 0.045171 | IXISKIFRJMSNOJ-XITYOTOGSA-N | Not Available |
| LF.141 | MOL009637 | 4-[(Z,1R)-3-(4-methoxyphenyl)-1-vinylprop-2-enyl]phenol | 43.47203793 | 266.360 | 0.15298 | MTYGOTBQCBXZQD-IJVDHGTGSA-N | 23626544 |
| LF.142 | MOL009638 | 1-(2-hydrazino-4-methyl-5-pyrimidinyl)ethanone | 38.52920074 | 166.210 | 0.03906 | QYDALMMTLZBJFX-UHFFFAOYSA-N | 341594 |
| LF.143 | MOL009639 | Lophenol | 38.12940252 | 400.760 | 0.714 | LMYZQUNLYGJIHI-SPONXPENSA-N | 160482 |
| LF.144 | MOL009640 | 4alpha,14alpha,24-trimethylcholesta-8,24-dienol | 38.90988973 | 426.800 | 0.75772 | GCZFZGBLBXBCJP-VXRRTDEQSA-N | Not Available |
| LF.145 | MOL009641 | 4alpha,24-dimethylcholesta-7,24-dienol | 42.65304098 | 412.770 | 0.75297 | KPIRFXVTLHBVFL-AAGDOFLISA-N | Not Available |
| LF.146 | MOL009642 | 4alpha-methyl-24-ethylcholesta-7,24-dienol | 42.29509453 | 426.800 | 0.78304 | HIIFUGOBGVCVLO-MTFRKTCUSA-N | Not Available |
| LF.147 | MOL009643 | 5-Butylnonane | 12.79646461 | 184.410 | 0.026726 | YXSQPZMAIVSYDV-UHFFFAOYSA-N | 300476 |
| LF.148 | MOL009644 | 6-Fluoroindole-7-Dehydrocholesterol | 43.72602513 | 402.700 | 0.72224 | HMEVHMIHKDZWLI-WXBCRLQESA-N | Not Available |
| LF.149 | MOL009645 | 7-O-Methylluteolin-6-C-beta-glucoside | 1.250686609 | 480.460 | 0.81572 | LDTLXTGZHCRGFD-HDUJVMFKSA-N | Not Available |
| LF.150 | MOL009646 | 7-O-Methylluteolin-6-C-beta-glucoside_qt | 40.77368843 | 318.300 | 0.30497 | PHGRMBKBKPAXKQ-LLVKDONJSA-N | Not Available |
| LF.151 | MOL009647 | 7-bromomethyl-7-hexadecene | 6.473259241 | 317.400 | 0.078832 | XWACLFGKPLOHDI-ICFOKQHNSA-N | Not Available |
| LF.152 | MOL009648 | 9-Octylheptadecane | 9.761082873 | 352.770 | 0.20234 | ZLIOPNROBPMBFI-UHFFFAOYSA-N | 292286 |
| LF.153 | MOL009649 | Anhydrorhodovibrin | 26.79476793 | 566.990 | 0.45997 | OCDSWQXGIQUZCF-AGVJHCIFSA-N | 5368308 |
| LF.154 | MOL009650 | Atropine | 42.15897078 | 289.410 | 0.19299 | RKUNBYITZUJHSG-JJXSEGSLSA-N | 637577 |
| LF.155 | MOL009651 | Cryptoxanthin monoepoxide | 46.95371937 | 568.960 | 0.56103 | CMOLUFWHADIFGS-WZIUPQIASA-N | Not Available |
| LF.156 | MOL009652 | Cycloartanolacetate | 6.384722115 | 470.860 | 0.79943 | RRIPWSLJXZPRIU-GUULDYRZSA-N | Not Available |
| LF.157 | MOL009653 | Cycloeucalenol | 39.72647216 | 426.800 | 0.79446 | HUNLTIZKNQDZEI-PGFZVWMDSA-N | Not Available |
| LF.158 | MOL009654 | Cyclofoetoside B | 8.240451355 | 933.270 | 0.075815 | JDYWIMCSAUNOHC-VVQWPGQESA-N | Not Available |
| LF.159 | MOL009655 | Cyclofoetoside B_qt | 10.9201851 | 492.820 | 0.78205 | VGUDSEYLIKUOJH-HUGZAVLDSA-N | Not Available |
| LF.160 | MOL009656 | (E,E)-1-ethyl octadeca-3,13-dienoate | 41.99620045 | 308.560 | 0.19364 | WDXJYOKJLIZASF-RSIFQWQVSA-N | Not Available |
| LF.161 | MOL009657 | Ethyl p-toluate | 39.64423118 | 164.220 | 0.03509 | NWPWRAWAUYIELB-UHFFFAOYSA-N | 66743 |
| LF.162 | MOL009658 | DGL | 60.77710368 | 147.150 | 0.021377 | WHUUTDBJXJRKMK-GSVOUGTGSA-N | 23327 |
| LF.163 | MOL009659 | Hypaconitine | 7.155121288 | 615.790 | 0.26085 | FIDOCHXHMJHKRW-ALHUPPKCSA-N | Not Available |
| LF.164 | MOL009660 | methyl (1R,4aS,7R,7aS)-4a,7-dihydroxy-7-methyl-1-[(2S,3R,4S,5S,6R)-3,4,5-trihydroxy-6-(hydroxymethyl)oxan-2-yl]oxy-1,5,6,7a-tetrahydrocyclopenta[d]pyran-4-carboxylate | 39.42847682 | 406.430 | 0.46558 | RWMXKBUPLSNIJL-VALIWVFLSA-N | 11968619 |
| LF.165 | MOL009661 | Ipolamiide_qt | 139.9587323 | 244.270 | 0.1198 | IAVUAKMWDUCOCS-CPOMMVLXSA-N | Not Available |
| LF.166 | MOL009662 | Lantadene A | 38.67942417 | 552.870 | 0.57405 | KCLIRHUTOPOHKJ-DMKBDCOSSA-N | Not Available |
| LF.167 | MOL009663 | Maaliol | 71.18852588 | 236.390 | 0.14612 | BEGPRDHBWMCZLC-SYZWTGEBSA-N | Not Available |
| LF.168 | MOL009664 | Physalin A | 91.70647491 | 526.580 | 0.27207 | VELDODQHYQSJOF-RLGRCWQRSA-N | Not Available |
| LF.169 | MOL009665 | Physcion-8-O-beta-D-gentiobioside | 43.90358656 | 608.600 | 0.62426 | LHWONDXFTUKXDH-BFTLVBKUSA-N | 5320543 |
| LF.170 | MOL009666 | Taurochenideixycholicacid | 15.76231491 | 499.790 | 0.87533 | BHTRKEVKTKCXOH-VNOLHDGTSA-N | Not Available |
| LF.171 | MOL009667 | 11Z-hexadecenoic acid | 35.77585321 | 254.460 | 0.10016 | JGMYDQCXGIMHLL-WAYWQWQTSA-N | 5312414 |
| LF.172 | MOL009668 | Zederone | 48.96773317 | 246.330 | 0.16855 | CVIVANCKIBYAOP-BKILABGBSA-N | Not Available |
| LF.173 | MOL009669 | aminoethyl thiosulfite | 72.15227015 | 141.240 | 0.0061665 | XTNGYWONEKLZFU-UHFFFAOYSA-N | Not Available |
| LF.174 | MOL009670 | 2-Pyridylamine | 79.04965885 | 94.130 | 0.010464 | ICSNLGPSRYBMBD-UHFFFAOYSA-N | 10439 |
| LF.175 | MOL009672 | beta-Cholestanol | 25.63362343 | 388.750 | 0.6735 | QYIXCDOBOSTCEI-QCYZZNICSA-N | 6665 |
| LF.176 | MOL009673 | cis,trans-1,6-dimethyl-spiro-[4,5]-decane | 46.65734264 | 166.340 | 0.04764 | NENGYSBMMAAPSK-GRYCIOLGSA-N | Not Available |
| LF.177 | MOL009674 | cryptoxanthin | 25.15706663 | 552.960 | 0.5714 | DMASLKHVQRHNES-GMKWGACXSA-N | 44554791 |
| LF.178 | MOL009675 | Ethyl anisate | 55.90362885 | 180.220 | 0.043943 | FHUODBDRWMIBQP-UHFFFAOYSA-N | 60979 |
| LF.179 | MOL009676 | DGN | 87.90022768 | 146.170 | 0.020527 | ZDXPYRJPNDTMRX-GSVOUGTGSA-N | 6992096 |
| LF.180 | MOL009677 | lanost-8-en-3beta-ol | 34.22630373 | 428.820 | 0.74036 | MBZYKEVPFYHDOH-BQNIITSRSA-N | 440560 |
| LF.181 | MOL009678 | lanost-8-enol | 34.22630373 | 428.820 | 0.74167 | MBZYKEVPFYHDOH-ZSLNGYDASA-N | Not Available |
| LF.182 | MOL009679 | lathosterol | 13.77621779 | 386.730 | 0.67698 | IZVFFXVYBHFIHY-SKCNUYALSA-N | 65728 |
| LF.183 | MOL009680 | mutatoxanthin | 5.710064316 | 570.930 | 0.58747 | BCGQYMDCTNZPSY-JKCCELRRSA-N | Not Available |
| LF.184 | MOL009681 | Obtusifoliol | 42.55200222 | 426.800 | 0.7565 | MMNYKQIDRZNIKT-VSADUBDNSA-N | 65252 |
| LF.185 | MOL009682 | octahydro-4,4,8,8-tetramethyl-4a,7-methano-4aH-naphth[1,8a-b]oxirene | 15.75796023 | 220.390 | 0.15862 | VQHLGZRKOZIABH-DRABBMOASA-N | Not Available |
| LF.186 | MOL009683 | p-Nitrobenzoic acid octadecyl ester | 4.68773443 | 419.670 | 0.52947 | VBSJEGSOHTUUGU-UHFFFAOYSA-N | 11200948 |
| LF.187 | MOL009685 | trans,trans-1,6-dimethyl-spiro-[4,5]-decane | 53.38017583 | 166.340 | 0.047646 | NENGYSBMMAAPSK-SDDRHHMPSA-N | Not Available |
| LF.188 | MOL010234 | delta-Carotene | 31.80094312 | 536.960 | 0.54639 | WGIYGODPCLMGQH-GOXCNPTKSA-N | 5281230 |
| SCF.1 | MOL000023 | Hemo-sol | 39.84097885 | 136.260 | 0.02231 | XMGQYMWWDOXHJM-JTQLQIEISA-N | 440917 |
| SCF.2 | MOL000024 | alpha-humulene | 22.97682413 | 204.390 | 0.061124 | FAMPSKZZVDUYOS-HRGUGZIWSA-N | 5281520 |
| SCF.3 | MOL000036 | beta-caryophyllene | 29.70229451 | 204.390 | 0.089893 | NPNUFJAVOOONJE-GFUGXAQUSA-N | 5281515 |
| SCF.4 | MOL000105 | protocatechuic acid | 25.36646796 | 154.130 | 0.035092 | YQUVCSBJEUQKSH-UHFFFAOYSA-N | 72 |
| SCF.5 | MOL000117 | Cymol | 27.20382673 | 134.240 | 0.022463 | HFPZCAJZSCWRBC-UHFFFAOYSA-N | 7463 |
| SCF.6 | MOL000118 | (L)-alpha-Terpineol | 48.79777273 | 154.280 | 0.030772 | WUOACPNHFRMFPN-SECBINFHSA-N | 443162 |
| SCF.7 | MOL000119 | ZINC02040970 | 40.43337559 | 222.410 | 0.06012 | FQTLCLSUCSAZDY-GOFCXVBSSA-N | 11241545 |
| SCF.8 | MOL000122 | 1,8-cineole | 39.72921646 | 154.280 | 0.049041 | WEEGYLXZBRQIMU-WAAGHKOSSA-N | 2758 |
| SCF.9 | MOL000125 | (-)-alpha-Pinene | 46.24981501 | 136.260 | 0.052565 | GRWFGVWFFZKLTI-IUCAKERBSA-N | 440968 |
| SCF.10 | MOL000126 | (-)-nopinene | 44.83529174 | 136.260 | 0.052595 | WTARULDDTDQWMU-IUCAKERBSA-N | 440967 |
| SCF.11 | MOL000128 | NERYLACETATE | 25.94000169 | 196.320 | 0.041771 | HIGQPQRQIQDZMP-DHZHZOJOSA-N | 1549026 |
| SCF.12 | MOL000162 | beta-Chamigrene | 31.99116089 | 204.390 | 0.080672 | WLNGPDPILFYWKF-OAHLLOKOSA-N | 442353 |
| SCF.13 | MOL000196 | L-Bornyl acetate | 65.52092198 | 196.320 | 0.075447 | KGEKLUUHTZCSIP-HOSYDEDBSA-N | 93009 |
| SCF.14 | MOL000197 | Myrcene | 24.96376008 | 136.260 | 0.015286 | UAHWPYUMFXYFJY-UHFFFAOYSA-N | 31253 |
| SCF.15 | MOL000198 | (R)-linalool | 39.80430084 | 154.280 | 0.022686 | CDOSHBSSFJOMGT-JTQLQIEISA-N | 443158 |
| SCF.16 | MOL000202 | Moslene | 33.01642148 | 136.260 | 0.02224 | YKFLAYDHMOASIY-UHFFFAOYSA-N | 7461 |
| SCF.17 | MOL000233 | delta-Terpineol | 55.10994784 | 154.280 | 0.030896 | SQIFACVGCPWBQZ-UHFFFAOYSA-N | 81722 |
| SCF.18 | MOL000264 | Tereben | 29.6244298 | 136.260 | 0.022251 | MOYAFQVGZZPNRA-UHFFFAOYSA-N | 11463 |
| SCF.19 | MOL000267 | beta-Citronellol | 38.88749784 | 156.300 | 0.019906 | QMVPMAAFGQKVCJ-SNVBAGLBSA-N | 101977 |
| SCF.20 | MOL000269 | Elemicin | 21.94067579 | 208.280 | 0.060865 | BPLQKQKXWHCZSS-UHFFFAOYSA-N | 10248 |
| SCF.21 | MOL000479 | Farnesene | 17.41806873 | 204.390 | 0.047569 | JSNRRGGBADWTMC-NTCAYCPXSA-N | 5281517 |
| SCF.22 | MOL000608 | ()-Terpinen-4-ol | 81.40667188 | 154.280 | 0.032323 | WRYLYDPHFGVWKC-SNVBAGLBSA-N | 2724161 |
| SCF.23 | MOL000611 | beta-Bourbonene | 16.97891757 | 204.390 | 0.11333 | YIRAHEODBQONHI-ZQNQSHIBSA-N | Not Available |
| SCF.24 | MOL000615 | delta-amorphene | 17.94609655 | 204.390 | 0.077173 | FUCYIEXQVQJBKY-ZFWWWQNUSA-N | 441005 |
| SCF.25 | MOL000676 | DBP | 64.5416405 | 278.380 | 0.13409 | DOIRQSBPFJWKBE-UHFFFAOYSA-N | 3026 |
| SCF.26 | MOL000696 | β-terpineol | 47.88836394 | 154.280 | 0.031028 | RUJPNZNXGCHGID-MGCOHNPYSA-N | 8748 |
| SCF.27 | MOL000748 | HMF | 45.06610249 | 126.120 | 0.019427 | NOEGNKMFWQHSLB-UHFFFAOYSA-N | 237332 |
| SCF.28 | MOL000765 | Citraurin beta | 20.5343046 | 432.700 | 0.55978 | AVPAEFHIEZLSLZ-KQGIJFHPSA-N | Not Available |
| SCF.29 | MOL000905 | ()-beta-Pinene | 44.76823783 | 136.260 | 0.052595 | WTARULDDTDQWMU-RKDXNWHRSA-N | 10290825 |
| SCF.30 | MOL000911 | Terpilene | 33.95079904 | 136.260 | 0.022228 | YHQGMYUVUMAZJR-UHFFFAOYSA-N | 7462 |
| SCF.31 | MOL000923 | ACETIC ACID,BORNYL ESTER | 67.15061585 | 196.320 | 0.075435 | KGEKLUUHTZCSIP-FOGDFJRCSA-N | 637531 |
| SCF.32 | MOL000968 | beta-Bisabolene | 29.58966171 | 204.390 | 0.055235 | XZRVRYFILCSYSP-HNNXBMFYSA-N | 68128 |
| SCF.33 | MOL001444 | LC 5504 | 18.52251691 | 318.500 | 0.3036 | MFAYXOLUJJXHCN-IZHFEEFNSA-N | Not Available |
| SCF.34 | MOL001566 | calarene | 52.16269245 | 204.390 | 0.11399 | MBIPADCEHSKJDQ-MUYACECFSA-N | 15560279 |
| SCF.35 | MOL001600 | copaene | 29.47338384 | 204.390 | 0.12401 | VLXDPFLIRFYIME-QRTUWBSPSA-N | Not Available |
| SCF.36 | MOL001604 | Linalool | 49.367371 | 170.280 | 0.042561 | SATQWIIUJKWZNO-SCZZXKLOSA-N | Not Available |
| SCF.37 | MOL001948 | Nootkatin | 31.82438355 | 232.350 | 0.079936 | MNMNTZYOZZLKSV-UHFFFAOYSA-N | 238797 |
| SCF.38 | MOL002028 | (+)-beta-Phellandrene | 40.30231564 | 136.260 | 0.022283 | LFJQCDVYDGGFCH-JTQLQIEISA-N | 442484 |
| SCF.39 | MOL002029 | ()-Cuparene | 38.26308654 | 202.370 | 0.07494 | SLKPBCXNFNIJSV-HNNXBMFYSA-N | 86895 |
| SCF.40 | MOL002153 | 1H-Cycloprop(e)azulen-7-ol, decahydro-1,1,7-trimethyl-4-methylene-, (1aR-(1aalpha,4aalpha,7beta,7abeta,7balpha))- | 82.32846156 | 220.390 | 0.12209 | FRMCCTDTYSRUBE-BGPZULBFSA-N | 92231 |
| SCF.41 | MOL002335 | beta-Gurjunene | 51.35822434 | 204.390 | 0.10414 | IRCZVRWQUNZGSH-DKTYCGPESA-N | 6450812 |
| SCF.42 | MOL002453 | (-)-Comphene | 34.97921026 | 136.260 | 0.039017 | CRPUJAZIXJMDBK-BDAKNGLRSA-N | 440966 |
| SCF.43 | MOL002455 | [(3S)-3,7-dimethyloct-6-enyl] acetate | 22.68036294 | 198.340 | 0.041156 | JOZKFWLRHCDGJA-NSHDSACASA-N | 6999975 |
| SCF.44 | MOL002516 | zingerone | 25.22551259 | 194.250 | 0.054204 | OJYLAHXKWMRDGS-UHFFFAOYSA-N | 31211 |
| SCF.45 | MOL003047 | [(1S)-endo]-(-)-Borneol | 83.54491086 | 154.280 | 0.05275 | DTGKSKDOIYIVQL-QXFUBDJGSA-N | 1201518 |
| SCF.46 | MOL003069 | quinic acid | 55.9242283 | 191.180 | 0.056729 | AAWZDTNXLSGCEK-WYWMIBKRSA-N | 6508 |
| SCF.47 | MOL003367 | Myricadiol | 13.58438558 | 442.800 | 0.76675 | RJAKLUPHSBOQNU-BCXDYLCKSA-N | Not Available |
| SCF.48 | MOL003519 | (E)-oct-2-en-4-one | 64.6159896 | 126.220 | 0.01152 | FMDLEUPBHMCPQV-GQCTYLIASA-N | 5365891 |
| SCF.49 | MOL003537 | T-Muurolol | 30.41445229 | 222.410 | 0.093121 | LHYHMMRYTDARSZ-AJNGGQMLSA-N | 3084331 |
| SCF.50 | MOL003541 | ()-alpha-Longipinene | 57.47116489 | 204.390 | 0.12474 | HICYDYJTCDBHMZ-UKTARXLSSA-N | Not Available |
| SCF.51 | MOL003546 | Aristolone | 45.30748966 | 218.370 | 0.13009 | UGVIZCBJCSXBCJ-JWFUOXDNSA-N | 165536 |
| SCF.52 | MOL003997 | 3-Furaldehyde | 50.95889475 | 96.090 | 0.010481 | AZVSIHIBYRHSLB-UHFFFAOYSA-N | 10351 |
| SCF.53 | MOL004174 | epsilon-Cadinene | 16.41043148 | 204.390 | 0.077488 | NOLWRMQDWRAODO-KKUMJFAQSA-N | 12302130 |
| SCF.54 | MOL004608 | (5S)-5-butyloxolan-2-one | 65.08186655 | 142.220 | 0.02095 | IPBFYZQJXZJBFQ-ZETCQYMHSA-N | 7057972 |
| SCF.55 | MOL004624 | Longikaurin A | 47.72214984 | 348.480 | 0.53015 | PSVHVXLCVSKJGM-MHRDNBEJSA-N | Not Available |
| SCF.56 | MOL004626 | Longispinogenin | 14.47504758 | 458.800 | 0.74887 | YHGVYECWZWIVJC-CXFYMCSDSA-N | Not Available |
| SCF.57 | MOL004678 | Limetin | 36.63110334 | 206.210 | 0.086466 | NXJCRELRQHZBQA-UHFFFAOYSA-N | 2775 |
| SCF.58 | MOL004723 | beta-Terpinene | 42.28925166 | 136.260 | 0.022295 | SCWPFSIZUZUCCE-UHFFFAOYSA-N | 66841 |
| SCF.59 | MOL005317 | Deoxyharringtonine | 39.27443988 | 515.660 | 0.8116 | WRCBXHDQHPUVHW-CDRKEARJSA-N | Not Available |
| SCF.60 | MOL005322 | Gamma-Selinene | 22.58083514 | 204.390 | 0.08087 | RMZHSBMIZBMVMN-HUUCEWRRSA-N | Not Available |
| SCF.61 | MOL005394 | (Z)-2-methyl-5-[(1S,2R,4R)-2-methyl-3-methylene-2-norbornanyl]pent-2-en-1-ol | 35.28336521 | 220.390 | 0.088125 | OJYKYCDSGQGTRJ-GQYWAMEOSA-N | 6857681 |
| SCF.62 | MOL005604 | Schisandrin | 7.693323595 | 432.560 | 0.65976 | YEFOAORQXAOVJQ-RKNYENMMSA-N | Not Available |
| SCF.63 | MOL006126 | Hexahydrocurcumin | 4.876340237 | 374.470 | 0.40571 | RSAHICAPUYTWHW-MRXNPFEDSA-N | 25763830 |
| SCF.64 | MOL006292 | δ-selinene | 19.01034506 | 204.390 | 0.080616 | VEGYMPQCXPVQJY-HNNXBMFYSA-N | 12308845 |
| SCF.65 | MOL007551 | α-santalene | 17.75660771 | 204.390 | 0.11164 | KWFJIXPIFLVMPM-AIEDFZFUSA-N | Not Available |
| SCF.66 | MOL007719 | Arnebin 7 | 73.8482146 | 272.320 | 0.17627 | VOMDIEGPEURZJO-UHFFFAOYSA-N | 98914 |
| SCF.67 | MOL008931 | Gomisin T | 8.008349162 | 402.530 | 0.58365 | RCPUCQCVTDMJGJ-QWHCGFSZSA-N | Not Available |
| SCF.68 | MOL008932 | (-)-Gomisin L1 | 9.379679522 | 386.480 | 0.70904 | OGJPBGDUYKEQLA-NWDGAFQWSA-N | Not Available |
| SCF.69 | MOL008933 | beta-Sesquiphellandrene | 30.57524882 | 218.420 | 0.063606 | FBMFZFQVVWUGRD-HRCADAONSA-N | Not Available |
| SCF.70 | MOL008934 | (-)-Gomisin L2 | 9.132759054 | 386.480 | 0.70706 | BVMLGLOHSDNEJG-NWDGAFQWSA-N | Not Available |
| SCF.71 | MOL008935 | thymoquinol 2-glucoside | 13.89664617 | 328.400 | 0.22741 | KSNLTHGWURXRJK-QMHWVQJVSA-N | Not Available |
| SCF.72 | MOL008936 | Thymoquinol | 44.43651588 | 166.240 | 0.038329 | OQIOHYHRGZNZCW-UHFFFAOYSA-N | 95779 |
| SCF.73 | MOL008937 | thymoquinol 5-glucoside | 15.30241585 | 328.400 | 0.22801 | XFPSRAYOBUHKRV-QMHWVQJVSA-N | Not Available |
| SCF.74 | MOL008938 | zingerone glucoside | 11.19876161 | 356.410 | 0.30219 | GXSGZLLXMDVQAS-KSWRQPAISA-N | Not Available |
| SCF.75 | MOL008939 | (9aS)-2,5,9,9-tetramethyl-3,4,6,7,8,9a-hexahydrobenzo[7]annulene | 45.35968187 | 204.390 | 0.079768 | LCOSCMLXPAQCLQ-CQSZACIVSA-N | 24798710 |
| SCF.76 | MOL008940 | γ-Cadinene | 21.33536121 | 204.390 | 0.077436 | YOCDGWMCBBMMGJ-RBSFLKMASA-N | 12358781 |
| SCF.77 | MOL008941 | (E)-9-Isopropyl-6-methyl-5,9-decadiene-2-one | 60.25457118 | 208.380 | 0.048444 | GVHYGHSXSIDTDI-KPKJPENVSA-N | 5318635 |
| SCF.78 | MOL008942 | 1,1alpha,4,5,6,7,7alpha,7beta-Octahydro-1,1,7,7alpha-tetramethyl-2H-cyclopropa (alpha)-naphthalen-2-one | 43.91268576 | 218.370 | 0.13008 | UGVIZCBJCSXBCJ-GCVYGYKWSA-N | 12305213 |
| SCF.79 | MOL008943 | 1,2,3,3alpha,8,8alpha-Hexahydro-2,2,8-trimethyl-5,6-azulenedimethanol | 12.1598999 | 236.390 | 0.10248 | YFVJPBYYCUEIKS-GYSYKLTISA-N | Not Available |
| SCF.80 | MOL008944 | 1-(1,5-Dimethyl-4-hexenyl)-4-methyl benzene | 30.94211529 | 208.430 | 0.053922 | PLGPPVNYZMVRCY-KKUMJFAQSA-N | Not Available |
| SCF.81 | MOL008945 | Benzoylacetone | 28.10670918 | 162.200 | 0.035608 | CVBUKMMMRLOKQR-UHFFFAOYSA-N | 7166 |
| SCF.82 | MOL008946 | Schisanhenol | 8.018626778 | 402.530 | 0.58537 | FYSHYFPJBONYCQ-QWHCGFSZSA-N | Not Available |
| SCF.83 | MOL008947 | 2,4-Dimethyl-6(3-methyl-isobuten-5-isopropyl)-phenyl-3,5 hexanedione | 6.057647531 | 328.540 | 0.19271 | NRKPQDAKPVCBIF-FXBPSFAMSA-N | Not Available |
| SCF.84 | MOL008948 | 3-carbomethoxy-3-hydroxy-glutaric acid | 28.81907253 | 206.170 | 0.053216 | HCVBQXINVUFVCE-UHFFFAOYSA-N | 12566215 |
| SCF.85 | MOL008949 | 3-Methyl betuletol | 22.1952479 | 346.360 | 0.3619 | QQLSQGGLMCHYTK-CQSZACIVSA-N | Not Available |
| SCF.86 | MOL008950 | 3-Phenyldecane | 5.185062552 | 218.420 | 0.063432 | PYVIFMPVFLOTLN-HNNXBMFYSA-N | Not Available |
| SCF.87 | MOL008951 | 3alpha-Tigloyloxytropane | 90.82386933 | 223.350 | 0.084871 | UVHGSMZRSVGWDJ-LKQNJMEQSA-N | Not Available |
| SCF.88 | MOL008952 | 4,7-dimethyl-7-(4-methylpent-3-enyl)bicyclo[2.2.1]heptan-3-ol | 24.95743795 | 222.410 | 0.093913 | SGAYOTORECIFCJ-ZQDZILKHSA-N | Not Available |
| SCF.89 | MOL008953 | 4-Ethenyl-2,2,4-trimethyl-3-(1-methylethenyl)-cyclo-hexane-methanol | 39.66614545 | 222.410 | 0.077172 | OUHIEGFKRWELJI-NFAWXSAZSA-N | Not Available |
| SCF.90 | MOL008954 | SMR000445689 | 9.516341093 | 386.480 | 0.70798 | PDDXWOMYBJCSQB-NEPJUHHUSA-N | Not Available |
| SCF.91 | MOL008955 | Angeloylgomisin H | 7.507978149 | 500.640 | 0.80819 | ZSAUXCVJDYCLRS-QEEHVONISA-N | Not Available |
| SCF.92 | MOL008956 | Angeloylgomisin O | 31.96538945 | 498.620 | 0.84763 | PLKFSXFJGNZAER-VDJLVHAZSA-N | Not Available |
| SCF.93 | MOL008957 | Schizandrer B | 30.70577053 | 514.620 | 0.82854 | BKGUPIVDQHHVMV-TWJXSMCESA-N | Not Available |
| SCF.94 | MOL008958 | Angeloylgomisin Q | 7.503208244 | 530.670 | 0.81312 | RHABJANPSGWEFC-JRJMQGIASA-N | Not Available |
| SCF.95 | MOL008959 | Angeloylisogomisin O | 7.522323388 | 498.620 | 0.85076 | PZUDCPSZWPLXKT-VDJLVHAZSA-N | Not Available |
| SCF.96 | MOL008960 | Benzoylgomisin H | 7.510450706 | 494.630 | 0.86196 | IMDRGXDKZPAJCS-ADKRDUOOSA-N | Not Available |
| SCF.97 | MOL008961 | Benzoylgomisin O | 7.589497111 | 492.610 | 0.85292 | GPXYRIDQNFPWMN-XOWTYJCDSA-N | Not Available |
| SCF.98 | MOL008962 | Benzoylgomisin P | 7.551339568 | 508.610 | 0.82292 | MSHJHBLTCDLVSX-YUTBMSQASA-N | Not Available |
| SCF.99 | MOL008963 | Benzoylgomisin Q | 7.504296759 | 524.660 | 0.84906 | UNXPYROOCYNIMG-JYBCAQAXSA-N | Not Available |
| SCF.100 | MOL008964 | Chamissonin diacetate | 10.27519328 | 348.430 | 0.32073 | DEBBYPCBXVYUCZ-WZLFHOKTSA-N | 5281433 |
| SCF.101 | MOL008965 | Clupanodonic acid | 44.01290151 | 276.460 | 0.14904 | GODPUQPTJYDDHR-HYGYRGJESA-N | 5315978 |
| SCF.102 | MOL008966 | Cyclokoreanine B | 17.48195508 | 414.750 | 0.73559 | YNGZPMBCHZYUHN-WBTNJATBSA-N | Not Available |
| SCF.103 | MOL008967 | Epiguaipyridine | 36.98196643 | 215.370 | 0.089577 | RVJOTNIWFWVNRS-DGCLKSJQSA-N | 13857098 |
| SCF.104 | MOL008968 | Gomisin-A | 30.69375343 | 416.510 | 0.77723 | ZWRRJEICIPUPHZ-MYODQAERSA-N | Not Available |
| SCF.105 | MOL008969 | Gomisin B | 12.65406322 | 498.570 | 0.76428 | NSSVWLXEWPFEIY-NNNXMFOHSA-N | Not Available |
| SCF.106 | MOL008970 | Gomisin C | 7.557497288 | 508.610 | 0.8229 | MSHJHBLTCDLVSX-CXNCDLMJSA-N | Not Available |
| SCF.107 | MOL008971 | Gomisin D | 7.875116732 | 530.620 | 0.58672 | VLLFEMVDMFTBHG-KMIFWYFFSA-N | Not Available |
| SCF.108 | MOL008972 | Gomisin E | 8.152747651 | 514.620 | 0.61997 | MLGBLGQFPHASJN-RKZUNUFISA-N | Not Available |
| SCF.109 | MOL008973 | Gomisin F | 7.520020076 | 514.620 | 0.8325 | LCQKYZXWCLBYHK-WIKKSFEZSA-N | Not Available |
| SCF.110 | MOL008974 | Gomisin G | 32.67834097 | 508.610 | 0.82713 | AZMQTBXLMHEDNG-JCNFZPKBSA-N | Not Available |
| SCF.111 | MOL008975 | Gomisin H | 7.884152208 | 418.530 | 0.62369 | NLJJSPKWNBUDNS-MYODQAERSA-N | Not Available |
| SCF.112 | MOL008976 | Gomisin J | 8.407250738 | 388.500 | 0.54311 | PICOUNAPKDEPCA-TXEJJXNPSA-N | Not Available |
| SCF.113 | MOL008977 | AIDS446185 | 8.770230345 | 416.510 | 0.77834 | GWDFJIBHVSYXQL-SYTFOFBDSA-N | Not Available |
| SCF.114 | MOL008978 | Gomisin R | 34.84255546 | 400.460 | 0.85805 | HOPDFAWBFXSPSA-FHGNATFXSA-N | Not Available |
| SCF.115 | MOL008979 | Gomisin S | 7.705183749 | 432.560 | 0.6603 | LRRQVPSJFUEMDR-QAJFTPDKSA-N | Not Available |
| SCF.116 | MOL008980 | Gomphrenin I | 5.926850873 | 552.540 | 0.80424 | YJNJBSPQGZKLJJ-QKLRNBKISA-N | Not Available |
| SCF.117 | MOL008981 | Gomphrenin I_qt | 23.04464382 | 390.380 | 0.51898 | BUNWMOMNBAQEIL-FCYDPWPLSA-N | Not Available |
| SCF.118 | MOL008982 | Hexahydroxytaxadiene | 8.281080378 | 368.520 | 0.40129 | RRAFCTVSIHVYHJ-AULKTIENSA-N | Not Available |
| SCF.119 | MOL008983 | 5,7-dihydroxy-2-(4-hydroxy-3-methoxyphenyl)-6-[(2S,3R,4S,5S,6R)-3,4,5-trihydroxy-6-(hydroxymethyl)oxan-2-yl]oxychromen-4-one | 20.90499173 | 478.440 | 0.82728 | HXITYQTUNBGSOA-GSVZXUNASA-N | 5318663 |
| SCF.120 | MOL008984 | (1R)-1-phenylpropan-1-ol | 70.32018425 | 136.210 | 0.022332 | DYUQAZSOFZSPHD-SECBINFHSA-N | 640199 |
| SCF.121 | MOL008985 | Psilostachyin A | 16.56074996 | 280.350 | 0.19237 | IRPFOXRBPHCCTG-SLGFJQRASA-N | Not Available |
| SCF.122 | MOL008986 | Rugosal | 20.9715244 | 266.370 | 0.17972 | NTVXLOKDTRVPSZ-FQKPHLNHSA-N | Not Available |
| SCF.123 | MOL008987 | Schisanhenol acetate | 19.67931602 | 444.570 | 0.70964 | OVPHXTVCLYBRDV-ZIAGYGMSSA-N | Not Available |
| SCF.124 | MOL008988 | Schizonepetoside A | 5.495611201 | 330.420 | 0.23402 | CMULNSPKSRFQAF-BDMIYPFCSA-N | Not Available |
| SCF.125 | MOL008989 | schizonepetoside A_qt | 60.29065471 | 168.260 | 0.036588 | HUWFDVNQSQKADM-PTEBFVFZSA-N | Not Available |
| SCF.126 | MOL008990 | 2-[(1R)-4-methyl-1-cyclohex-3-enyl]propan-2-yl acetate | 33.04760568 | 196.320 | 0.051384 | IGODOXYLBBXFDW-NSHDSACASA-N | 11469649 |
| SCF.127 | MOL008991 | Thujyalcohol | 73.07856319 | 154.280 | 0.051242 | DZVXRFMREAADPP-IMSYWVGJSA-N | 12304610 |
| SCF.128 | MOL008992 | Wuweizisu C | 46.26685721 | 384.460 | 0.8436 | HTBWBWWADZJXID-TXEJJXNPSA-N | Not Available |
| SCF.129 | MOL008993 | Wyerone | 79.23736047 | 258.290 | 0.12694 | NQZCQIDFBYCBAU-JHGYFVHDSA-N | 643733 |
| SCF.130 | MOL008994 | alpha-Cuparenol | 55.70061624 | 218.370 | 0.088611 | AFRLMSOCDMQZOR-HIFRSBDPSA-N | 5316200 |
| EH.1 | MOL000006 | luteolin | 36.16262934 | 286.250 | 0.24552 | IQPNAANSBPBGFQ-UHFFFAOYSA-N | 5280445 |
| EH.2 | MOL000008 | apigenin | 23.06216102 | 270.250 | 0.21306 | KZNIFHPLKGYRTM-UHFFFAOYSA-N | 5280443 |
| EH.3 | MOL000098 | quercetin | 46.43334812 | 302.250 | 0.27525 | REFJWTPEDVJJIY-UHFFFAOYSA-N | 5280343 |
| EH.4 | MOL000118 | (L)-alpha-Terpineol | 48.79777273 | 154.280 | 0.030772 | WUOACPNHFRMFPN-SECBINFHSA-N | 443162 |
| EH.5 | MOL000120 | dec-2-enal | 18.54769732 | 154.280 | 0.02049 | MMFCJPPRCYDLLZ-CMDGGOBGSA-N | 5283345 |
| EH.6 | MOL000130 | CAM | 67.17381285 | 152.260 | 0.053027 | DSSYKIVIOFKYAU-XCBNKYQSSA-N | 159055 |
| EH.7 | MOL000198 | (R)-linalool | 39.80430084 | 154.280 | 0.022686 | CDOSHBSSFJOMGT-JTQLQIEISA-N | 443158 |
| EH.8 | MOL000203 | Izosafrol | 56.91673775 | 162.200 | 0.046883 | VHVOLFRBFDOUSH-IHWYPQMZSA-N | 1549044 |
| EH.9 | MOL000205 | (6R)-6-isopropyl-3-methyl-1-cyclohex-2-enone | 53.87781814 | 152.260 | 0.029701 | YSTPAHQEHQSRJD-SECBINFHSA-N | 107561 |
| EH.10 | MOL000207 | Methyleugenol | 73.36011441 | 178.250 | 0.042845 | ZYEMGPIYFIJGTP-UHFFFAOYSA-N | 7127 |
| EH.11 | MOL000244 | ()-Borneol | 81.80314484 | 154.280 | 0.052753 | DTGKSKDOIYIVQL-WEDXCCLWSA-N | 6552009 |
| EH.12 | MOL000263 | oleanolic acid | 29.02084142 | 456.780 | 0.75599 | MIJYXULNPSFWEK-GTOFXWBISA-N | 10494 |
| EH.13 | MOL000305 | lauric acid | 23.58793922 | 200.360 | 0.043637 | POULHZVOKOAJMA-UHFFFAOYSA-N | 3893 |
| EH.14 | MOL000325 | (2R,3R)-2-(3,4-dimethoxyphenyl)-7-methoxy-3-methyl-5-[(E)-prop-1-enyl]-2,3-dihydrobenzofuran | 24.19190119 | 340.450 | 0.34542 | ITFKWUHXYCXXFF-XSOBDOKWSA-N | 6441048 |
| EH.15 | MOL000357 | Sitogluside | 20.63193686 | 576.950 | 0.6241 | NPJICTMALKLTFW-OFUAXYCQSA-N | 5742590 |
| EH.16 | MOL000359 | sitosterol | 36.91390583 | 414.790 | 0.7512 | KZJWDPNRJALLNS-ZFVHJZABSA-N | 12303645 |
| EH.17 | MOL000399 | Docosanoate | 15.68844863 | 340.660 | 0.26054 | UKMSUNONTOPOIO-UHFFFAOYSA-N | 8215 |
| EH.18 | MOL000422 | kaempferol | 41.88224954 | 286.250 | 0.24066 | IYRMWMYZSQPJKC-UHFFFAOYSA-N | 5280863 |
| EH.19 | MOL000437 | Hirsutrin | 1.858078251 | 464.410 | 0.76904 | OVSQVDMCBVZWGM-QSOFNFLRSA-N | 5280804 |
| EH.20 | MOL000472 | emodin | 24.39832432 | 270.250 | 0.23916 | RHMXXJGYXNZAPX-UHFFFAOYSA-N | 3220 |
| EH.21 | MOL000478 | Eucarvone | 53.13613969 | 150.240 | 0.030032 | QNGQIURXCUHNAT-UHFFFAOYSA-N | 136330 |
| EH.22 | MOL000561 | Astragalin | 14.02684507 | 448.410 | 0.73616 | JPUKWEQWGBDDQB-QSOFNFLRSA-N | 5282102 |
| EH.23 | MOL000622 | Magnograndiolide | 63.70888436 | 266.370 | 0.18833 | VHFXPBHLQOPQHJ-ABBQYLIMSA-N | 5319198 |
| EH.24 | MOL000667 | 1-hexanol | 22.04296444 | 102.200 | 0.0054272 | ZSIAUFGUXNUGDI-UHFFFAOYSA-N | 8103 |
| EH.25 | MOL000695 | patchouli alcohol | 101.9647362 | 222.410 | 0.13624 | GGHMUJBZYLPWFD-CUZKYEQNSA-N | Not Available |
| EH.26 | MOL000740 | (+)-Cycloolivil | 24.50473442 | 376.440 | 0.42259 | KCIQZCNOUZCRGH-VOBQZIQPSA-N | 5316262 |
| EH.27 | MOL001510 | 24-epicampesterol | 37.57681789 | 400.760 | 0.71413 | SGNBVLSWZMBQTH-ZRUUVFCLSA-N | 5283637 |
| EH.28 | MOL001579 | germacrene | 15.05844059 | 208.430 | 0.056031 | IQSPYJZCWMAJLU-CYSUXKILSA-N | Not Available |
| EH.29 | MOL001600 | copaene | 29.47338384 | 204.390 | 0.12401 | VLXDPFLIRFYIME-QRTUWBSPSA-N | Not Available |
| EH.30 | MOL001640 | NON | 26.73806656 | 172.300 | 0.027306 | GHVNFZFCNZKVNT-UHFFFAOYSA-N | 2969 |
| EH.31 | MOL001645 | Linoleyl acetate | 42.10076623 | 308.560 | 0.19845 | KFXARGMQYWECBV-ZDVGBALWSA-N | 5319042 |
| EH.32 | MOL001707 | 24190-29-2 | 21.67254932 | 192.330 | 0.054312 | UZFLPKAIBPNNCA-ABZNLYFFSA-N | 638013 |
| EH.33 | MOL001771 | poriferast-5-en-3beta-ol | 36.91390583 | 414.790 | 0.75034 | KZJWDPNRJALLNS-FBZNIEFRSA-N | 457801 |
| EH.34 | MOL001789 | isoliquiritigenin | 85.32179823 | 256.270 | 0.14805 | DXDRHHKMWQZJHT-FPYGCLRLSA-N | 638278 |
| EH.35 | MOL001792 | DFV | 32.76272375 | 256.270 | 0.18316 | FURUXTVZLHCCNA-AWEZNQCLSA-N | 114829 |
| EH.36 | MOL001972 | Pulegone | 51.59647117 | 152.260 | 0.029676 | NZGWDASTMWDZIW-MRVPVSSYSA-N | 442495 |
| EH.37 | MOL002040 | (1S,4R)-fenchone | 72.63902396 | 152.260 | 0.050709 | LHXDLQBQYFFVNW-XCBNKYQSSA-N | 1201521 |
| EH.38 | MOL002083 | tricin | 27.85992866 | 330.310 | 0.33891 | HRGUSFBJBOKSML-UHFFFAOYSA-N | 5281702 |
| EH.39 | MOL002085 | alpha-Cubebene | 16.73208261 | 204.390 | 0.10854 | XUEHVOLRMXNRKQ-KHMAMNHCSA-N | Not Available |
| EH.40 | MOL002307 | 20-Hexadecanoylingenol | 28.20395415 | 586.940 | 0.68297 | DSTCZBGJCUOFLM-SXNKARFESA-N | Not Available |
| EH.41 | MOL002361 | Terragon | 36.589797 | 148.220 | 0.027965 | ZFMSMUAANRJZFM-UHFFFAOYSA-N | 8815 |
| EH.42 | MOL002509 | Ginkgetin | 22.1896269 | 566.540 | 0.58939 | AIFCFBUSLAEIBR-UHFFFAOYSA-N | 5271805 |
| EH.43 | MOL002511 | Isoginkgetin | 21.55757968 | 566.540 | 0.5847 | HUOOMAOYXQFIDQ-UHFFFAOYSA-N | 5318569 |
| EH.44 | MOL002689 | 3,4,5-Trimethoxytoluene | 23.7290308 | 182.240 | 0.044897 | KCIZTNZGSBSSRM-UHFFFAOYSA-N | 80922 |
| EH.45 | MOL002697 | junipene | 44.07374961 | 204.390 | 0.10826 | PDSNLYSELAIEBU-ABHRYQDASA-N | Not Available |
| EH.46 | MOL002891 | magnoflorine | 0.47998537 | 342.450 | 0.54735 | YHJFRXGWUVSYOV-ZDUSSCGKSA-N | 73337 |
| EH.47 | MOL002929 | salidroside | 7.010257973 | 300.340 | 0.19818 | ILRCGYURZSFMEG-MQLXINIDSA-N | Not Available |
| EH.48 | MOL002930 | Tyrosol | 33.81193302 | 138.180 | 0.023971 | YCCILVSKPBXVIP-UHFFFAOYSA-N | 10393 |
| EH.49 | MOL003030 | Ginnol | 11.32791548 | 424.890 | 0.42613 | CPGCVOVWHCWVTP-GDLZYMKVSA-N | 342803 |
| EH.50 | MOL003044 | Chryseriol | 35.85089483 | 300.280 | 0.27415 | SCZVLDHREVKTSH-UHFFFAOYSA-N | 5280666 |
| EH.51 | MOL003097 | Flavone der. | 27.12376669 | 298.310 | 0.26743 | LZERJKGWTQYMBB-UHFFFAOYSA-N | 5281601 |
| EH.52 | MOL003518 | Vetol | 53.22920747 | 126.120 | 0.022632 | XPCTZQVDEJYUGT-UHFFFAOYSA-N | 8369 |
| EH.53 | MOL003520 | Damascenone | 36.42671434 | 190.310 | 0.054675 | POIARNZEYGURDG-FNORWQNLSA-N | 5366074 |
| EH.54 | MOL003542 | 8-Isopentenyl-kaempferol | 38.04433524 | 354.380 | 0.3948 | NADCVNHITZNGJU-UHFFFAOYSA-N | 5318624 |
| EH.55 | MOL003547 | Azaron | 38.38574731 | 208.280 | 0.060667 | RKFAZBXYICVSKP-AATRIKPKSA-N | 636822 |
| EH.56 | MOL004363 | (Z)-heptadec-3-ene | 20.24323888 | 238.510 | 0.075621 | YICJXYRVTBRLKS-ALCCZGGFSA-N | 13529206 |
| EH.57 | MOL004364 | 3,5-Dimethoxytoluene | 20.871324 | 152.210 | 0.029432 | RIZBLVRXRWHLFA-UHFFFAOYSA-N | 77844 |
| EH.58 | MOL004365 | Isomenthol | 55.30444011 | 156.300 | 0.029154 | NOOLISFMXDJSKH-OPRDCNLKSA-N | 19244 |
| EH.59 | MOL004366 | Octyl formate | 53.32366606 | 158.270 | 0.021108 | AVBRYQRTMPHARE-UHFFFAOYSA-N | 8176 |
| EH.60 | MOL004367 | olivil | 62.22859563 | 376.440 | 0.40642 | BVHIKUCXNBQDEM-XMCHAPAWSA-N | Not Available |
| EH.61 | MOL004368 | Hyperin | 6.93898582 | 464.410 | 0.76905 | OVSQVDMCBVZWGM-DTGCRPNFSA-N | 5281643 |
| EH.62 | MOL004369 | quercetin-3-rhamnooside | 2.608567025 | 448.410 | 0.7365 | OXGUCUVFOIWWQJ-AIRRAIBWSA-N | 40486293 |
| EH.63 | MOL004370 | Robinetin | 6.350398807 | 302.250 | 0.27538 | SOEDEYVDCDYMMH-UHFFFAOYSA-N | 5281692 |
| EH.64 | MOL004371 | rouhuoside | 3.618678499 | 824.860 | 0.31399 | JACMCCOFXWLINV-DASSDAFJSA-N | Not Available |
| EH.65 | MOL004372 | Sagittatoside A | 8.500725648 | 676.730 | 0.56959 | COHHGQPQHHUMDG-SXIVMDCFSA-N | Not Available |
| EH.66 | MOL004373 | Anhydroicaritin | 45.41193421 | 368.410 | 0.43786 | TUUXBSASAQJECY-UHFFFAOYSA-N | 5318980 |
| EH.67 | MOL004374 | sagittatoside B | 5.583750444 | 646.700 | 0.64316 | BVDGQVAUJNUPGW-LBEJLRJHSA-N | Not Available |
| EH.68 | MOL004375 | Trifolin | 3.097105504 | 448.410 | 0.73616 | JPUKWEQWGBDDQB-DTGCRPNFSA-N | 5282149 |
| EH.69 | MOL004376 | wanepimedoside A | 6.541509855 | 678.750 | 0.57706 | BVEUNAOFYLSYJU-LCIUXTAESA-N | Not Available |
| EH.70 | MOL004377 | wanepimedoside_qt | 6.025415496 | 386.430 | 0.48093 | VAYWXTLNNGACLF-UHFFFAOYSA-N | 12310757 |
| EH.71 | MOL004378 | Wushanicariin | 5.487720704 | 530.570 | 0.85506 | LLNBDBPIVSGXJA-ZANOAUCBSA-N | 3082728 |
| EH.72 | MOL004379 | wushanicariin_qt | 23.35930945 | 368.410 | 0.46277 | RTLJXHMORZDCAR-UHFFFAOYSA-N | Not Available |
| EH.73 | MOL004380 | C-Homoerythrinan, 1,6-didehydro-3,15,16-trimethoxy-, (3.beta.)- | 39.13992598 | 329.480 | 0.49461 | VFNBFPRWBICVGZ-JXFKEZNVSA-N | Not Available |
| EH.74 | MOL004381 | Besigomsin | 28.52265836 | 416.510 | 0.77726 | ZWRRJEICIPUPHZ-SFDCACGMSA-N | Not Available |
| EH.75 | MOL004382 | Yinyanghuo A | 56.9573795 | 420.490 | 0.76747 | ZAUWPDSVLSOCDG-SFHVURJKSA-N | Not Available |
| EH.76 | MOL004383 | Yinyanghuo B | 1.071103405 | 422.510 | 0.60365 | FIZBURLMLRCZTA-LJQANCHMSA-N | Not Available |
| EH.77 | MOL004384 | Yinyanghuo C | 45.67199685 | 336.360 | 0.50155 | GPXYBBZISZKRAH-UHFFFAOYSA-N | 5315395 |
| EH.78 | MOL004385 | Yinyanghuo D | 13.98524246 | 338.380 | 0.37995 | PFQMUQWFRINBBG-UHFFFAOYSA-N | 5315396 |
| EH.79 | MOL004386 | Yinyanghuo E | 51.63212506 | 352.360 | 0.5474 | FIKLOAGOJKGOFT-UHFFFAOYSA-N | 5315397 |
| EH.80 | MOL004387 | Yixinoside A | 6.132252091 | 1107.490 | 0.04011 | NELKWBXZWCWANB-UIFFJYSWSA-N | Not Available |
| EH.81 | MOL004388 | 6-hydroxy-11,12-dimethoxy-2,2-dimethyl-1,8-dioxo-2,3,4,8-tetrahydro-1H-isochromeno[3,4-h]isoquinolin-2-ium | 60.64150904 | 370.410 | 0.65693 | FUBYUUKASUJMSZ-UHFFFAOYSA-N | 12115137 |
| EH.82 | MOL004389 | 3-Hexenyl-beta-glucopyranoside | 12.12215967 | 262.340 | 0.10776 | OZIPFYKAIOOVEJ-WQPBHKFLSA-N | 5318046 |
| EH.83 | MOL004390 | 5,7,4'-trihydroxy8,3'-diprenylflavone | 1.01538062 | 406.510 | 0.58874 | NZIDLHYOWGCCCH-UHFFFAOYSA-N | Not Available |
| EH.84 | MOL004391 | 8-(3-methylbut-2-enyl)-2-phenyl-chromone | 48.54449639 | 290.380 | 0.25066 | FMPOBQILEGSRSJ-UHFFFAOYSA-N | 17861868 |
| EH.85 | MOL004392 | acuminatoside | 3.013824784 | 985.050 | 0.15494 | NXXVKOAEAAJROE-OCUWVLQZSA-N | Not Available |
| EH.86 | MOL004393 | anhydroicaritin | 28.27352777 | 368.410 | 0.59309 | PPCHTBBOSVKORE-UHFFFAOYSA-N | 14583584 |
| EH.87 | MOL004394 | Anhydroicaritin-3-O-alpha-L-rhamnoside | 41.5834004 | 676.730 | 0.60981 | TZJALUIVHRYQQB-YPRONELTSA-N | Not Available |
| EH.88 | MOL004395 | artonin U | 19.32902732 | 352.410 | 0.39114 | BSZYNZDGUZYMDL-UHFFFAOYSA-N | 44258358 |
| EH.89 | MOL004396 | 1,2-bis(4-hydroxy-3-methoxyphenyl)propan-1,3-diol | 52.31424958 | 320.370 | 0.22066 | DFUOJBWSSSODTR-SJCJKPOMSA-N | 12468616 |
| EH.90 | MOL004397 | baohuoside Ⅵ | 6.056085529 | 822.890 | 0.31821 | ULZLIYVOYYQJRO-FFHJHMEHSA-N | Not Available |
| EH.91 | MOL004398 | 3,5,7-Trihydroxy-4'-methoxyl-8-prenylflavone-3-O-rhamnopyranoside | 3.701024841 | 514.570 | 0.83885 | NGMYNFJANBHLKA-LVKFHIPRSA-N | 5488822 |
| EH.92 | MOL004399 | Baohuoside VI | 4.93561187 | 822.890 | 0.31438 | BTSHIWNGQGFPHJ-XODVFGJGSA-N | 11972471 |
| EH.93 | MOL004400 | Bilobanol | 14.22492548 | 234.370 | 0.0945 | SNMIMWYZCXBQQW-SWLSCSKDSA-N | Not Available |
| EH.94 | MOL004401 | bilobetin | 7.266010147 | 552.510 | 0.63317 | IWEIJEPIYMAGTH-UHFFFAOYSA-N | 5315459 |
| EH.95 | MOL004402 | brevicornin | 14.09321249 | 400.460 | 0.52143 | YUCRNUHJPUHOKK-UHFFFAOYSA-N | 15291301 |
| EH.96 | MOL004403 | caohuoside B | 3.016067105 | 965.010 | 0.18929 | FLAHBCCEDHEPGQ-UZORMKMBSA-N | Not Available |
| EH.97 | MOL004404 | caohuoside D | 24.88513494 | 562.620 | 0.82583 | IINPFAXRBBMPBJ-RAUBWGAYSA-N | Not Available |
| EH.98 | MOL004405 | 3-[(2S,3R,4R,5R,6S)-4,5-dihydroxy-6-methyl-3-[(2S,3R,4S,5S,6R)-3,4,5-trihydroxy-6-methyl-tetrahydropyran-2-yl]oxy-tetrahydropyran-2-yl]oxy-5-hydroxy-2-(4-methoxyphenyl)-8-(3-methylbut-2-enyl)-7-[(2S,3R,4S,5S,6R)-3,4,5-trihydroxy-6-methylol-tetrahydropyran | 6.056085529 | 822.890 | 0.31788 | ULZLIYVOYYQJRO-CEWIFYNKSA-N | 24721194 |
| EH.99 | MOL004406 | 2,15-Hexadecanedione | 8.153817748 | 254.460 | 0.10061 | ANOHLAYDIMKILU-UHFFFAOYSA-N | 458096 |
| EH.100 | MOL004407 | Epimedin B | 8.65312545 | 792.860 | 0.34125 | NVWDLULBKVOJFI-ZOCTVJJZSA-N | 25087708 |
| EH.101 | MOL004408 | Epimedin C_qt | 5.868957979 | 352.410 | 0.4003 | FGAWELLANIGUSJ-UHFFFAOYSA-N | Not Available |
| EH.102 | MOL004409 | Epimedin C | 16.29483623 | 790.890 | 0.34469 | KJRQFQYOQYXTRI-HEJAAUFXSA-N | 5317090 |
| EH.103 | MOL004410 | epimedokoreanone A | 59.84942744 | 142.120 | 0.029524 | PCWQIUUSFGFNSZ-UHFFFAOYSA-N | 54728446 |
| EH.104 | MOL004411 | (2S,3S)-3,5-dihydroxy-2-(4-hydroxyphenyl)-8-(3-methylbut-2-enyl)-7-[(2S,3R,4S,5S,6R)-3,4,5-trihydroxy-6-methylol-tetrahydropyran-2-yl]oxy-chroman-4-one | 3.580618078 | 518.560 | 0.83915 | GRDZTDZJQRPNCN-MMDZVIBYSA-N | 7163182 |
| EH.105 | MOL004413 | epimedoside C | 2.671474297 | 516.540 | 0.83699 | FJSOHLNMRNSYFR-UHXIJUMASA-N | Not Available |
| EH.106 | MOL004414 | epimedoside D | 5.489009722 | 794.830 | 0.34473 | FZHKFLMLBFFDJD-GQEKEQRWSA-N | Not Available |
| EH.107 | MOL004415 | Epimedoside E | 5.489009722 | 794.830 | 0.35201 | XDBZJWHRPCMWOO-NDANIPDNSA-N | Not Available |
| EH.108 | MOL004417 | epimedoside | 14.32023022 | 760.810 | 0.40802 | ZRGOVKQDBSFQIU-CQIFHBRTSA-N | Not Available |
| EH.109 | MOL004418 | DOB | 88.18404175 | 154.130 | 0.035244 | UIAFKZKHHVMJGS-UHFFFAOYSA-N | 1491 |
| EH.110 | MOL004419 | globulol | 19.94481443 | 222.410 | 0.12056 | AYXPYQRXGNDJFU-IMNVLQEYSA-N | 11996452 |
| EH.111 | MOL004420 | Hentriacontanol-6 | 11.05859619 | 452.950 | 0.53774 | BTIPNGPHUGUYAR-WJOKGBTCSA-N | Not Available |
| EH.112 | MOL004421 | hexandraside D | 5.435218403 | 822.890 | 0.31724 | LMZZWQKHDOVICB-QGQRWFJPSA-N | Not Available |
| EH.113 | MOL004422 | Hexandraside E | 13.55626344 | 678.700 | 0.596 | SLUGZPRLJCECEX-YVBMKHBZSA-N | Not Available |
| EH.114 | MOL004423 | hexandraside F | 3.668554708 | 838.890 | 0.29556 | NLVBYGTTYRFJKH-QSNGFQFUSA-N | Not Available |
| EH.115 | MOL004424 | Icaride A2 | 4.762280808 | 436.500 | 0.5399 | WKDDUPJDCWIWAP-HCIHMXRSSA-N | 15690604 |
| EH.116 | MOL004425 | Icariin | 41.5834004 | 676.730 | 0.61051 | TZJALUIVHRYQQB-XLRXWWTNSA-N | 5318997 |
| EH.117 | MOL004426 | Icariresinol | 7.133505493 | 433.470 | 0.52595 | MUYFUAAWKHTZDY-BHDDXSALSA-N | Not Available |
| EH.118 | MOL004427 | Icariside A7 | 31.90509191 | 462.490 | 0.85568 | HNMHZSRVQJZGPQ-PUIBNRJISA-N | 5318401 |
| EH.119 | MOL004428 | 3,4,6-trimethoxyphenanthrene-2,7-diol | 24.45738387 | 300.330 | 0.29767 | XGYYXBDYFNKQER-UHFFFAOYSA-N | 356766 |
| EH.120 | MOL004429 | icariside C1 | 5.123562048 | 418.590 | 0.37397 | PFJDJYAPRBPXLV-YKHOZLKDSA-N | Not Available |
| EH.121 | MOL004430 | icariside I | 21.87912754 | 530.570 | 0.84822 | IYCPMVXIUPYNHI-RBOMYRCTSA-N | Not Available |
| EH.122 | MOL004431 | icariside II | 3.701024841 | 514.570 | 0.839 | NGMYNFJANBHLKA-DYAPJGKMSA-N | Not Available |
| EH.123 | MOL004432 | 4H-1-Benzopyran-4-one, 3-((6-deoxy-alpha-L-mennopyranosyl)oxy)-5,7-dihydroxy-2-(4-hydroxyphenyl)-8-(3-methyl-2-butenyl)- | 4.750616797 | 500.540 | 0.82063 | RPLMLWBOUPDPQF-GULSFEPBSA-N | 5481982 |
| EH.124 | MOL004433 | 3-[(2S,3R,4S,5S,6R)-4,5-dihydroxy-6-methylol-3-[(2S,3R,4R,5R,6S)-3,4,5-trihydroxy-6-methyl-tetrahydropyran-2-yl]oxy-tetrahydropyran-2-yl]oxy-5,7-dihydroxy-2-(4-hydroxyphenyl)-8-(3-methylbut-2-enyl)chromone | 2.933783083 | 662.700 | 0.61213 | CJJCKYUGKAVWRW-LBNASTDVSA-N | 5318411 |
| EH.125 | MOL004434 | Ikarisoside C | 4.962231257 | 822.890 | 0.31395 | ZOIXTVDCTOAUDQ-FTRGSOTFSA-N | Not Available |
| EH.126 | MOL004435 | Ikarisoside F | 1.953835517 | 632.670 | 0.66552 | JFEVWNOGUFYCOV-NPFMWIEOSA-N | 5318413 |
| EH.127 | MOL004436 | Ikshusterol | 8.996922926 | 430.790 | 0.78524 | SXJVFYZNUGGHRG-BRVKPZBJSA-N | Not Available |
| EH.128 | MOL004437 | Lespedin | 7.969737184 | 578.570 | 0.78692 | PUPKKEQDLNREIM-QNSQPKOQSA-N | 5486199 |
| EH.129 | MOL004438 | korepimedoside A | 14.92897532 | 748.800 | 0.43535 | URYGGNASCSKKGR-WGMUAILBSA-N | Not Available |
| EH.130 | MOL004439 | korepimedoside B | 3.016810285 | 965.010 | 0.18982 | AHIXHBMUHIBLGS-LMMCPZMWSA-N | Not Available |
| FTB.1 | MOL000358 | beta-sitosterol | 36.91390583 | 414.790 | 0.75123 | KZJWDPNRJALLNS-VJSFXXLFSA-N | 222284 |
| FTB.2 | MOL000365 | syringaresinol | 3.291713283 | 418.480 | 0.72269 | KOWMJRJXZMEZLD-GKHNXXNSSA-N | 12309694 |
| FTB.3 | MOL001004 | pelargonidin | 37.98831233 | 271.260 | 0.21204 | SAURRTSFHXYOSN-UHFFFAOYSA-N | Not Available |
| FTB.4 | MOL003171 | 2,5-Dimethoxybenzoquinone | 8.721436037 | 168.160 | 0.041233 | RMMPZDDLWLALLJ-UHFFFAOYSA-N | 101405 |
| FTB.5 | MOL004440 | Peimisine | 57.4023933 | 427.690 | 0.8055 | KYELXPJVGNZIGC-GKFGJCLESA-N | 161294 |
| FTB.6 | MOL004442 | Pelargonidin-3,5-diglucoside | 10.9564535 | 595.580 | 0.75571 | YZIWUQDWVPEKFW-ZOTFFYTFSA-N | Not Available |
| FTB.7 | MOL004443 | Zhebeiresinol | 58.72053449 | 280.300 | 0.19384 | AUXYOVQIZNPKSO-KKFJDGPESA-N | Not Available |
| FTB.8 | MOL004444 | Ziebeimine | 64.24657792 | 413.710 | 0.70486 | OEJGVNMSFPGDPP-BDFZTFKFSA-N | Not Available |
| FTB.9 | MOL004445 | Verticine | 17.41967307 | 431.730 | 0.66834 | IUKLSMSEHKDIIP-BZMYINFQSA-N | Not Available |
| FTB.10 | MOL004446 | 6-Methoxyl-2-acetyl-3-methyl-1,4-naphthoquinone-8-O-beta-D-glucopyranoside | 33.30734381 | 422.420 | 0.57257 | GVMOOQOCLYWVKF-BNCZOOBYSA-N | 5319462 |
| FTB.11 | MOL004447 | 6-Methoxyl-2-acetyl-3-methyl-1,4-naphthoquinone-8-O-beta-D-glucopyranoside_qt | 19.87055512 | 260.260 | 0.15427 | GBEJSKTVOVIDMP-UHFFFAOYSA-N | 67877406 |
| FTB.12 | MOL004448 | Solatubin | 17.11866921 | 397.710 | 0.75605 | JVKYZPBMZPJNAJ-OQFNDJACSA-N | Not Available |
| FTB.13 | MOL004449 | OSI-2040 | 14.65142951 | 623.880 | 0.60624 | JWOGUUIOCYMBPV-GMFLJSBRSA-N | 6918328 |
| FTB.14 | MOL004450 | Chaksine | 65.63417036 | 450.660 | 0.66463 | CGGAHJGHSHWGLE-WJQMWINMSA-N | 120699 |
| FTB.15 | MOL004451 | Peiminine | 13.19805701 | 429.710 | 0.66975 | IQDIERHFZVCNRZ-MEYDDXOOSA-N | Not Available |
| FTB.16 | MOL004452 | Peiminoside | 2.251686894 | 593.890 | 0.21108 | HRUKKZDXKJUOSO-AXBHCRIWSA-N | Not Available |
| FTB.17 | MOL004453 | Peiminoside_qt | 11.75276358 | 431.730 | 0.66821 | IUKLSMSEHKDIIP-FWSIRKJPSA-N | Not Available |
| PF.1 | MOL000006 | luteolin | 36.16262934 | 286.250 | 0.24552 | IQPNAANSBPBGFQ-UHFFFAOYSA-N | 5280445 |
| PF.2 | MOL000008 | apigenin | 23.06216102 | 270.250 | 0.21306 | KZNIFHPLKGYRTM-UHFFFAOYSA-N | 5280443 |
| PF.3 | MOL000012 | Arachic acid | 16.65635621 | 312.600 | 0.19499 | VKOBVWXKNCXXDE-UHFFFAOYSA-N | 10467 |
| PF.4 | MOL000023 | Hemo-sol | 39.84097885 | 136.260 | 0.02231 | XMGQYMWWDOXHJM-JTQLQIEISA-N | 440917 |
| PF.5 | MOL000024 | alpha-humulene | 22.97682413 | 204.390 | 0.061124 | FAMPSKZZVDUYOS-HRGUGZIWSA-N | 5281520 |
| PF.6 | MOL000036 | beta-caryophyllene | 29.70229451 | 204.390 | 0.089893 | NPNUFJAVOOONJE-GFUGXAQUSA-N | 5281515 |
| PF.7 | MOL000057 | DIBP | 49.6340827 | 278.380 | 0.13067 | MGWAVDBGNNKXQV-UHFFFAOYSA-N | 6782 |
| PF.8 | MOL000069 | palmitic acid | 19.2965647 | 256.480 | 0.098573 | IPCSVZSSVZVIGE-UHFFFAOYSA-N | 985 |
| PF.9 | MOL000116 | Nonanal | 40.27634015 | 142.270 | 0.015196 | GYHFUZHODSMOHU-UHFFFAOYSA-N | 31289 |
| PF.10 | MOL000118 | (L)-alpha-Terpineol | 48.79777273 | 154.280 | 0.030772 | WUOACPNHFRMFPN-SECBINFHSA-N | 443162 |
| PF.11 | MOL000122 | 1,8-cineole | 39.72921646 | 154.280 | 0.049041 | WEEGYLXZBRQIMU-WAAGHKOSSA-N | 2758 |
| PF.12 | MOL000126 | (-)-nopinene | 44.83529174 | 136.260 | 0.052595 | WTARULDDTDQWMU-IUCAKERBSA-N | 440967 |
| PF.13 | MOL000131 | EIC | 41.90443602 | 280.500 | 0.14347 | OYHQOLUKZRVURQ-HZJYTTRNSA-N | 5280450 |
| PF.14 | MOL000198 | (R)-linalool | 39.80430084 | 154.280 | 0.022686 | CDOSHBSSFJOMGT-JTQLQIEISA-N | 443158 |
| PF.15 | MOL000208 | ()-Aromadendrene | 55.7416731 | 204.390 | 0.10418 | ITYNGVSTWVVPIC-XVIXHAIJSA-N | 11095734 |
| PF.16 | MOL000254 | eugenol | 56.24190209 | 164.220 | 0.036518 | RRAFCDWBNXTKKO-UHFFFAOYSA-N | 3314 |
| PF.17 | MOL000267 | beta-Citronellol | 38.88749784 | 156.300 | 0.019906 | QMVPMAAFGQKVCJ-SNVBAGLBSA-N | 101977 |
| PF.18 | MOL000358 | beta-sitosterol | 36.91390583 | 414.790 | 0.75123 | KZJWDPNRJALLNS-VJSFXXLFSA-N | 222284 |
| PF.19 | MOL000432 | linolenic acid | 45.00906591 | 278.480 | 0.14709 | DTOSIQBPPRVQHS-PDBXOOCHSA-N | 5280934 |
| PF.20 | MOL000449 | Stigmasterol | 43.82985158 | 412.770 | 0.75665 | HCXVJBMSMIARIN-PHZDYDNGSA-N | 5280794 |
| PF.21 | MOL000610 | TRD | 17.89122181 | 184.410 | 0.031021 | IIYFAKIEWZDVMP-UHFFFAOYSA-N | 12388 |
| PF.22 | MOL000611 | beta-Bourbonene | 16.97891757 | 204.390 | 0.11333 | YIRAHEODBQONHI-ZQNQSHIBSA-N | Not Available |
| PF.23 | MOL000615 | delta-amorphene | 17.94609655 | 204.390 | 0.077173 | FUCYIEXQVQJBKY-ZFWWWQNUSA-N | 441005 |
| PF.24 | MOL000666 | hexanal | 55.70702938 | 100.180 | 0.0055795 | JARKCYVAAOWBJS-UHFFFAOYSA-N | 6184 |
| PF.25 | MOL000675 | oleic acid | 33.12836481 | 282.520 | 0.14243 | ZQPPMHVWECSIRJ-KTKRTIGZSA-N | 445639 |
| PF.26 | MOL000708 | WLN: VHR | 32.62796592 | 106.130 | 0.014163 | HUMNYLRZRPPJDN-UHFFFAOYSA-N | 240 |
| PF.27 | MOL000860 | stearic acid | 17.82542938 | 284.540 | 0.14086 | QIQXTHQIDYTFRH-UHFFFAOYSA-N | 5281 |
| PF.28 | MOL000868 | LFA | 8.4605189 | 282.620 | 0.12999 | CBFCDTFDPHXCNY-UHFFFAOYSA-N | 8222 |
| PF.29 | MOL000909 | (3S,4R)-3-isopropenyl-1-isopropyl-4-methyl-4-vinylcyclohexene | 27.56639659 | 204.390 | 0.060189 | MXDMETWAEGIFOE-GJZGRUSLSA-N | 12004383 |
| PF.30 | MOL000932 | alpha-Farnesene | 21.70351121 | 204.390 | 0.047239 | CXENHBSYCFFKJS-VDQVFBMKSA-N | 5281516 |
| PF.31 | MOL000935 | Hepanal | 53.83317567 | 204.390 | 0.10397 | SPCXZDDGSGTVAW-XIDUGBJDSA-N | 15560276 |
| PF.32 | MOL000937 | 58870_FLUKA | 49.01059782 | 204.390 | 0.1039 | NUQDPKOFUKFKFD-BGOOENEXSA-N | 15431199 |
| PF.33 | MOL000953 | CLR | 37.87389754 | 386.730 | 0.67677 | HVYWMOMLDIMFJA-DPAQBDIFSA-N | 5997 |
| PF.34 | MOL001098 | m-xylene | 47.42747459 | 106.180 | 0.014128 | IVSZLXZYQVIEFR-UHFFFAOYSA-N | 7929 |
| PF.35 | MOL001201 | (1R,5R,7S)-4,7-dimethyl-7-(4-methylpent-3-enyl)bicyclo[3.1.1]hept-3-ene | 16.22557141 | 204.390 | 0.090114 | YMBFCQPIMVLNIU-KFWWJZLASA-N | 13889654 |
| PF.36 | MOL001283 | C09704 | 29.5610598 | 222.410 | 0.059922 | FQTLCLSUCSAZDY-ATGUSINASA-N | 5281525 |
| PF.37 | MOL001392 | Methyl myristate | 19.68203501 | 242.450 | 0.079634 | ZAZKJZBWRNNLDS-UHFFFAOYSA-N | 31284 |
| PF.38 | MOL001394 | Oktadekan | 9.806386361 | 254.560 | 0.090617 | RZJRJXONCZWCBN-UHFFFAOYSA-N | 11635 |
| PF.39 | MOL001439 | arachidonic acid | 45.57324991 | 304.520 | 0.20409 | YZXBAPSDXZZRGB-DOFZRALJSA-N | 444899 |
| PF.40 | MOL001501 | Daturic acid | 18.51057885 | 270.510 | 0.11827 | KEMQGTRYUADPNZ-UHFFFAOYSA-N | 10465 |
| PF.41 | MOL001600 | copaene | 29.47338384 | 204.390 | 0.12401 | VLXDPFLIRFYIME-QRTUWBSPSA-N | Not Available |
| PF.42 | MOL001739 | zoomaric acid | 35.77585321 | 254.460 | 0.099334 | SECPZKHBENQXJG-FPLPWBNLSA-N | 445638 |
| PF.43 | MOL001819 | METHYL PENTADECANOATE | 18.81525029 | 256.480 | 0.096511 | XIUXKAZJZFLLDQ-UHFFFAOYSA-N | 23518 |
| PF.44 | MOL001896 | bicyclo[3.1.1]hept-2-ene-2-methanol, 6,6-dimethyl- | 49.78860979 | 152.260 | 0.059011 | RXBQNMWIQKOSCS-RKDXNWHRSA-N | 636475 |
| PF.45 | MOL001901 | 24-Methylenecycloartanol | 10.39704683 | 440.830 | 0.78773 | BDHQMRXFDYJGII-UEBIAWITSA-N | Not Available |
| PF.46 | MOL001943 | lecithin | 0.266908251 | 758.200 | 0.29607 | NCEZGAAXVCBWBV-ZTIMHPMXSA-N | Not Available |
| PF.47 | MOL002031 | toluene | 42.5804258 | 92.150 | 0.00997 | YXFVVABEGXRONW-UHFFFAOYSA-N | 1140 |
| PF.48 | MOL002085 | alpha-Cubebene | 16.73208261 | 204.390 | 0.10854 | XUEHVOLRMXNRKQ-KHMAMNHCSA-N | Not Available |
| PF.49 | MOL002133 | 1,5,5-trimethyl-6-methylenecyclohexene | 46.07761588 | 136.260 | 0.028744 | FMXKKHBXBBAQBC-UHFFFAOYSA-N | 578237 |
| PF.50 | MOL002166 | ISOHEPTANE | 59.94045013 | 100.230 | 0.0055808 | GXDHCNNESPLIKD-UHFFFAOYSA-N | 11582 |
| PF.51 | MOL002203 | Exceparl M-OL | 31.89848081 | 296.550 | 0.16457 | QYDYPVFESGNLHU-KHPPLWFESA-N | 5364509 |
| PF.52 | MOL002378 | UND | 17.15299654 | 156.350 | 0.018387 | RSJKGSCJYJTIGS-UHFFFAOYSA-N | 14257 |
| PF.53 | MOL002678 | EB | 49.38328773 | 106.180 | 0.012997 | YNQLUTRBYVCPMQ-UHFFFAOYSA-N | 7500 |
| PF.54 | MOL002699 | Cetene | 5.338042166 | 224.480 | 0.062078 | GQEZCXVZFLOKMC-UHFFFAOYSA-N | 12395 |
| PF.55 | MOL002773 | beta-carotene | 37.18433337 | 536.960 | 0.58358 | OENHQHLEOONYIE-JLTXGRSLSA-N | 5280489 |
| PF.56 | MOL003053 | Methyl isomyristate | 20.2628253 | 242.450 | 0.078792 | FLESKWMKPOBWDE-UHFFFAOYSA-N | 21204 |
| PF.57 | MOL003127 | Germacrene D | 19.22250577 | 204.390 | 0.057074 | GAIBLDCXCZKKJE-RXJOXMPGSA-N | 5317570 |
| PF.58 | MOL003533 | Bicyclo[4.4.0]dec-1-ene,2-isopropy-5-methyl-9-methylene- | 19.77698117 | 204.390 | 0.077235 | FTSINDMZMFBWFS-JSGCOSHPSA-N | Not Available |
| PF.59 | MOL003622 | Methyl geranate | 26.35056915 | 182.290 | 0.033376 | ACOBBFVLNKYODD-CSKARUKUSA-N | 5365910 |
| PF.60 | MOL003717 | METHYL PALMITOLEATE | 34.6122634 | 268.490 | 0.1172 | IZFGRAGOVZCUFB-HJWRWDBZSA-N | 643801 |
| PF.61 | MOL003752 | cycloartanol | 27.20069495 | 428.820 | 0.7762 | YABASAWVVRQMEU-YBXTVTTCSA-N | Not Available |
| PF.62 | MOL003924 | Methyl azelate | 25.11886432 | 216.310 | 0.059455 | DRUKNYVQGHETPO-UHFFFAOYSA-N | 15612 |
| PF.63 | MOL004355 | Spinasterol | 42.97936552 | 412.770 | 0.75534 | JZVFJDZBLUFKCA-FXIAWGAOSA-N | 5281331 |
| PF.64 | MOL004590 | 2-METHYLHEXADECANE | 4.192982019 | 240.530 | 0.073648 | FNWWOHKUXFTKGN-UHFFFAOYSA-N | 15266 |
| PF.65 | MOL004627 | Methyl hexoate | 52.43726115 | 130.210 | 0.012058 | NUKZAGXMHTUAFE-UHFFFAOYSA-N | 7824 |
| PF.66 | MOL004682 | Methyl octylate | 18.71242187 | 158.270 | 0.020709 | JGHZJRVDZXSNKQ-UHFFFAOYSA-N | 8091 |
| PF.67 | MOL004727 | (1R,4aR,8aS)-1-isopropyl-7-methyl-4-methylene-2,3,4a,5,6,8a-hexahydro-1H-naphthalene | 21.34764637 | 204.390 | 0.077315 | WRHGORWNJGOVQY-QLFBSQMISA-N | 6432308 |
| PF.68 | MOL005030 | gondoic acid | 30.70294255 | 310.580 | 0.19743 | BITHHVVYSMSWAG-KTKRTIGZSA-N | 5282768 |
| PF.69 | MOL005043 | campest-5-en-3beta-ol | 37.57681789 | 400.760 | 0.71481 | SGNBVLSWZMBQTH-PODYLUTMSA-N | 173183 |
| PF.70 | MOL005151 | [(3S)-3,7-dimethylocta-1,6-dien-3-yl] acetate | 36.84342535 | 196.320 | 0.041586 | UWKAYLJWKGQEPM-GFCCVEGCSA-N | 6999980 |
| PF.71 | MOL005425 | Methyl caprate | 17.79795072 | 186.330 | 0.033893 | YRHYCMZPEVDGFQ-UHFFFAOYSA-N | 8050 |
| PF.72 | MOL005481 | 2,6,10,14,18-pentamethylicosa-2,6,10,14,18-pentaene | 33.4041173 | 342.670 | 0.24028 | IMXDCJPVYKXJPD-FMOJUEAUSA-N | 5366013 |
| PF.73 | MOL005712 | 3-methyl-6-(1-methylethylidene)-cyclohexene | 29.13667569 | 136.260 | 0.022217 | CIPXOBMYVWRNLL-SECBINFHSA-N | 71385764 |
| PF.74 | MOL005752 | [(4R)-4-isopropenyl-1-cyclohexenyl]methanol | 49.0128549 | 152.260 | 0.028346 | NDTYTMIUWGWIMO-JTQLQIEISA-N | 11788398 |
| PF.75 | MOL005971 | Nonadecylic acid | 17.20838049 | 298.570 | 0.16641 | ISYWECDDZWTKFF-UHFFFAOYSA-N | 12591 |
| PF.76 | MOL006289 | cis-Z-alpha-Bisabolene epoxide | 21.32504715 | 220.390 | 0.088069 | BOWJPMUUGHPAAF-VJUFAYPDSA-N | Not Available |
| PF.77 | MOL007233 | Methyl nonylate | 54.12027566 | 172.300 | 0.026672 | IJXHLVMUNBOGRR-UHFFFAOYSA-N | 15606 |
| PF.78 | MOL007262 | Methyl phenylacetate | 22.69969385 | 150.190 | 0.028334 | CRZQGDNQQAALAY-UHFFFAOYSA-N | 7559 |
| PF.79 | MOL007270 | DIMETHYL SUBERATE | 16.92816954 | 202.280 | 0.048324 | LNLCRJXCNQABMV-UHFFFAOYSA-N | 15611 |
| PF.80 | MOL007344 | tryptophane | 28.55485768 | 385.350 | 0.23724 | UNJJBGNPUUVVFQ-NXEZZACHSA-N | 44147587 |
| PF.81 | MOL007449 | 24-methylidenelophenol | 44.19264545 | 412.770 | 0.7533 | RSMKYRDCCSNYFM-AAGDOFLISA-N | 5283640 |
| PF.82 | MOL007577 | 2,4-Dimethylpentane | 48.96998827 | 100.230 | 0.0063031 | BZHMBWZPUJHVEE-UHFFFAOYSA-N | 7907 |
| PF.83 | MOL008401 | Henicosanoic acid | 16.14247045 | 326.630 | 0.22694 | CKDDRHZIAZRDBW-UHFFFAOYSA-N | 16898 |
| PF.84 | MOL008594 | methyl (Z)-hexadec-7-enoate | 34.6122634 | 268.490 | 0.11713 | FXCDESKKWMGGON-KHPPLWFESA-N | 14029831 |
| PF.85 | MOL008615 | Methyl 9-oxononanoate | 24.01872778 | 186.280 | 0.038082 | JMLYDLZRFNYHHO-UHFFFAOYSA-N | 74732 |
| PF.86 | MOL008653 | Acetylfuran | 49.63293985 | 110.120 | 0.014332 | IEMMBWWQXVXBEU-UHFFFAOYSA-N | 14505 |
| PF.87 | MOL009653 | Cycloeucalenol | 39.72647216 | 426.800 | 0.79446 | HUNLTIZKNQDZEI-PGFZVWMDSA-N | Not Available |
| PF.88 | MOL009681 | Obtusifoliol | 42.55200222 | 426.800 | 0.7565 | MMNYKQIDRZNIKT-VSADUBDNSA-N | 65252 |
| PF.89 | MOL010222 | Muscamone | 16.66019192 | 322.690 | 0.21239 | IGOWHGRNPLFNDJ-ZPHPHTNESA-N | 5365075 |
| PF.90 | MOL012563 | 3,6-dimethyldecane | 14.02167834 | 170.380 | 0.023057 | NQWFSCYWTXQNGG-NWDGAFQWSA-N | Not Available |
| PF.91 | MOL012862 | methyl (E)-dodec-9-enoate | 38.77305311 | 212.370 | 0.054258 | DUWQEMMRMJGHSA-SNAWJCMRSA-N | 5362755 |
| PF.92 | MOL012863 | [1R-(1.alpha.,3a.beta.,4.alpha.,8a.beta.,9S)]-decahydro-1,5,5,8a-tetramethyl-1,4-Methanoazulen-9-ol | 95.01890034 | 222.410 | 0.1284 | MNNFKQAYXGEKFA-AMVBZWJASA-N | Not Available |
| PF.93 | MOL012864 | Cellon | 7.573820359 | 167.840 | 0.0047401 | QPFMBZIOSGYJDE-UHFFFAOYSA-N | 6591 |
| PF.94 | MOL012865 | 1,4-diethyl-1,4-dimethyl-2,5-cyclohexadiene | 19.43390163 | 164.320 | 0.03901 | JTZDQRQJYHFOLP-HAQNSBGRSA-N | 572347 |
| PF.95 | MOL012866 | linalyl anthranilate | 64.91872773 | 273.410 | 0.12399 | WHIJSULEEDNKPD-QGZVFWFLSA-N | Not Available |
| PF.96 | MOL012867 | 1.3-dimethyl-4.8-dioxatricyclo[5.1.0.0(3.5)]octane-2,6-diol | 54.86179336 | 172.200 | 0.082444 | BVMYHHZHLJPRQI-QEEWUKHFSA-N | Not Available |
| PF.97 | MOL012868 | Methyl 12-tridecynoate | 14.22263288 | 224.380 | 0.069149 | LLYUQTVONAGBRD-UHFFFAOYSA-N | 554090 |
| PF.98 | MOL012869 | 1-chloro-4-mesyl-benzene | 26.3803164 | 190.660 | 0.032081 | LMCOQDVJBWVNNI-UHFFFAOYSA-N | 7395 |
| PF.99 | MOL012870 | 2-(1-Hydroxyethyl)norbornadiene | 81.65071648 | 136.210 | 0.032329 | FHTKYSNAPXOHOI-GJMOJQLCSA-N | Not Available |
| PF.100 | MOL012871 | 2-(4-Methylphenyl)indolizine | 26.53749956 | 207.290 | 0.10836 | DCLHWYSXBSMRRM-UHFFFAOYSA-N | 346948 |
| PF.101 | MOL012872 | WLN: Q1XGGG | 7.523691855 | 149.400 | 0.0065481 | KPWDGTGXUYRARH-UHFFFAOYSA-N | 8259 |
| PF.102 | MOL012873 | (3R)-2,2-dimethyl-3-propyloxirane | 51.11990052 | 114.210 | 0.021424 | ZWMFGIJPJXEKCV-ZCFIWIBFSA-N | 21720138 |
| PF.103 | MOL012874 | 2,5-dimethyl-3-methylenehexa-1,5-diene | 38.02331698 | 122.230 | 0.012889 | MMKJXJXYQZCQPL-UHFFFAOYSA-N | 535055 |
| PF.104 | MOL012875 | (1S,2S)-2-ethylcyclohexan-1-ol | 22.45897853 | 128.240 | 0.018412 | CFYUBZHJDXXXQE-YUMQZZPRSA-N | 6994303 |
| PF.105 | MOL012876 | 2-hexyl-cyclopropaneoctanoic acid methyl ester | 22.93770695 | 282.520 | 0.15013 | LQYIDOFTTFCXEO-IAGOWNOFSA-N | 14325746 |
| PF.106 | MOL012877 | 3,4-Dichlorobutane nitrile | 55.89075805 | 138 | 0.0061127 | PUFZOQWAWMKJAQ-BYPYZUCNSA-N | Not Available |
| PF.107 | MOL012878 | 3-Chloropropylsulfonyl chloride | 61.04358062 | 177.060 | 0.010784 | GPKDGVXBXQTHRY-UHFFFAOYSA-N | 15410 |
| PF.108 | MOL012879 | 3-oxiranyl-7-oxabicyclo[4.1.0]heptane | 60.55499882 | 140.200 | 0.049678 | MJNRFYZBHDTOEX-BIIVOSGPSA-N | Not Available |
| PF.109 | MOL012880 | ()-Isopulegol | 54.47534633 | 154.280 | 0.029538 | ZYTMANIQRDEHIO-AEJSXWLSSA-N | 1268090 |
| PF.110 | MOL012881 | 5-Methyl-2-phenylindole | 83.21469007 | 207.290 | 0.108 | JPFTUUXPCFNLIX-UHFFFAOYSA-N | 83247 |
| PF.111 | MOL012882 | Benzylidenemalonaldehyde | 18.71242187 | 160.180 | 0.035928 | XMXUYXIFVYQLKJ-UHFFFAOYSA-N | 583063 |
| PF.112 | MOL012883 | beta-patchoulene | 50.68856541 | 204.390 | 0.10733 | CSKINCSXMLCMAR-JRPNMDOOSA-N | Not Available |
| PF.113 | MOL012884 | butyrospermol | 8.696870434 | 426.800 | 0.75068 | DICCPNLDOZNSML-MUQOESGTSA-N | 12302190 |
| PF.114 | MOL012885 | Propyl chloroacetate | 30.97276661 | 136.590 | 0.0087601 | QJZNRCWAXUGABH-UHFFFAOYSA-N | 79378 |
| PF.115 | MOL012886 | Chloroethyl acetate | 40.23926135 | 122.560 | 0.0062443 | VIRWKAJWTKAIMA-UHFFFAOYSA-N | 10959 |
| PF.116 | MOL012887 | Chloromesyl chloride | 7.871907822 | 149 | 0.0068059 | KQDDQXNVESLJNO-UHFFFAOYSA-N | 77054 |
| PF.117 | MOL012888 | citrostadienol | 43.28127042 | 426.800 | 0.78568 | LPZCCMIISIBREI-ZXBKQEFASA-N | Not Available |
| PF.118 | MOL012889 | (-)-cis-.beta.-Elemene | 29.84626564 | 204.390 | 0.060512 | OPFTUNCRGUEPRZ-RRFJBIMHSA-N | 6431151 |
| PF.119 | MOL012890 | HAI | 86.34158165 | 99.200 | 0.0097644 | PAFZNILMFXTMIY-UHFFFAOYSA-N | 7965 |
| PF.120 | MOL012891 | (2E,4E,6E)-icosa-2,4,6-trienoic acid | 41.64184852 | 306.540 | 0.20213 | BBWMTEYXFFWPIF-CJBMEHDJSA-N | 6506063 |
| PF.121 | MOL012892 | methyl hept-2-enoate | 48.66984913 | 142.220 | 0.016205 | IQQDLHGWGKEQDS-VOTSOKGWSA-N | 5368087 |
| PF.122 | MOL012893 | (E)-(4-methylbenzylidene)-(4-phenyltriazol-1-yl)amine | 57.87220103 | 262.340 | 0.18812 | HGKZMFCXZIIEEU-GZTJUZNOSA-N | 9602469 |
| PF.123 | MOL012894 | 2-nonadecenoic acid | 29.83939407 | 296.550 | 0.16857 | INKQLYSQWHBEGS-ISLYRVAYSA-N | 5282766 |
| PF.124 | MOL012895 | ()-Perillaaldehyde | 42.93880958 | 150.240 | 0.028842 | RUMOYJJNUMEFDD-JTQLQIEISA-N | 1548901 |
| PF.125 | MOL012896 | Phthalic acid, butyl isohexyl ester | 45.5176676 | 306.440 | 0.17825 | MTYBJKMGPVGKGM-UHFFFAOYSA-N | 6423865 |
| PF.126 | MOL012897 | O-propyl aminosulfanylmethanethioate | 7.306543933 | 151.280 | 0.0081751 | JUKUHBMHFZORKU-UHFFFAOYSA-N | 536887 |
| PF.127 | MOL012899 | Tricyclo[5.2.1.0(2,6)]decan-10-one | 113.5376759 | 150.240 | 0.054695 | SORUNZRWXXOVLF-OJOKCITNSA-N | Not Available |
| PF.128 | MOL012900 | Trimethylphosphine oxide | 58.93591086 | 92.090 | 0.0044048 | LRMLWYXJORUTBG-UHFFFAOYSA-N | 69609 |
| AJH.1 | MOL000024 | alpha-humulene | 22.97682413 | 204.390 | 0.061124 | FAMPSKZZVDUYOS-HRGUGZIWSA-N | 5281520 |
| AJH.2 | MOL000032 | beta-Eudesmol | 26.09155723 | 222.410 | 0.095471 | BOPIMTNSYWYZOC-VNHYZAJKSA-N | 91457 |
| AJH.3 | MOL000066 | alloaromadedrene | 53.46135969 | 204.390 | 0.10414 | ITYNGVSTWVVPIC-PDWCTOEPSA-N | 44584667 |
| AJH.4 | MOL000098 | quercetin | 46.43334812 | 302.250 | 0.27525 | REFJWTPEDVJJIY-UHFFFAOYSA-N | 5280343 |
| AJH.5 | MOL000108 | quercetin 3-o-rhamnopyranosyl | 3.201533128 | 610.570 | 0.68243 | IKGXIBQEEMLURG-GALJDQSWSA-O | Not Available |
| AJH.6 | MOL000111 | quercetin,3-o-rutinoside | 3.201533128 | 610.570 | 0.683 | IKGXIBQEEMLURG-BKUODXTLSA-O | Not Available |
| AJH.7 | MOL000130 | CAM | 67.17381285 | 152.260 | 0.053027 | DSSYKIVIOFKYAU-XCBNKYQSSA-N | 159055 |
| AJH.8 | MOL000193 | (Z)-caryophyllene | 30.29074814 | 204.390 | 0.089886 | NPNUFJAVOOONJE-BLOBHPOZSA-N | 6429301 |
| AJH.9 | MOL000198 | (R)-linalool | 39.80430084 | 154.280 | 0.022686 | CDOSHBSSFJOMGT-JTQLQIEISA-N | 443158 |
| AJH.10 | MOL000206 | isoeugenol | 70.10031992 | 164.220 | 0.036329 | BJIOGJUNALELMI-ONEGZZNKSA-N | 853433 |
| AJH.11 | MOL000208 | ()-Aromadendrene | 55.7416731 | 204.390 | 0.10418 | ITYNGVSTWVVPIC-XVIXHAIJSA-N | 11095734 |
| AJH.12 | MOL000244 | ()-Borneol | 81.80314484 | 154.280 | 0.052753 | DTGKSKDOIYIVQL-WEDXCCLWSA-N | 6552009 |
| AJH.13 | MOL000252 | farnesol | 28.44199135 | 222.410 | 0.059083 | CRDAMVZIKSXKFV-YFVJMOTDSA-N | 445070 |
| AJH.14 | MOL000259 | o-Thymol | 43.28127042 | 150.240 | 0.029778 | RECUKUPTGUEGMW-UHFFFAOYSA-N | 10364 |
| AJH.15 | MOL000263 | oleanolic acid | 29.02084142 | 456.780 | 0.75599 | MIJYXULNPSFWEK-GTOFXWBISA-N | 10494 |
| AJH.16 | MOL000305 | lauric acid | 23.58793922 | 200.360 | 0.043637 | POULHZVOKOAJMA-UHFFFAOYSA-N | 3893 |
| AJH.17 | MOL000422 | kaempferol | 41.88224954 | 286.250 | 0.24066 | IYRMWMYZSQPJKC-UHFFFAOYSA-N | 5280863 |
| AJH.18 | MOL000463 | 16844-71-6 | 27.33568744 | 428.820 | 0.75524 | XCDQFROEGGNAER-PFOIMGGJSA-N | 119242 |
| AJH.19 | MOL000474 | (-)-Epoxycaryophyllene | 35.93684943 | 220.390 | 0.12925 | NVEQFIOZRFFVFW-RGCMKSIDSA-N | Not Available |
| AJH.20 | MOL000508 | Friedelin | 29.16218092 | 426.800 | 0.75897 | OFMXGFHWLZPCFL-SVRPQWSVSA-N | 91472 |
| AJH.21 | MOL000674 | Farnesol acetate | 21.96949125 | 264.450 | 0.10557 | ZGIGZINMAOQWLX-NCZFFCEISA-N | 638500 |
| AJH.22 | MOL000701 | quercitrin | 4.03765307 | 448.410 | 0.73649 | OXGUCUVFOIWWQJ-HQBVPOQASA-N | 5280459 |
| AJH.23 | MOL000708 | WLN: VHR | 32.62796592 | 106.130 | 0.014163 | HUMNYLRZRPPJDN-UHFFFAOYSA-N | 240 |
| AJH.24 | MOL000714 | Hyacinthin | 38.64648502 | 120.160 | 0.017812 | DTUQWGWMVIHBKE-UHFFFAOYSA-N | 998 |
| AJH.25 | MOL000719 | methyl salicylate | 42.55004268 | 152.160 | 0.032031 | OSWPMRLSEDHDFF-UHFFFAOYSA-N | 4133 |
| AJH.26 | MOL000775 | EEE | 45.0163211 | 88.120 | 0.0045272 | XEKOWRVHYACXOJ-UHFFFAOYSA-N | 8857 |
| AJH.27 | MOL000874 | paeonol | 28.78723811 | 166.190 | 0.039185 | UILPJVPSNHJFIK-UHFFFAOYSA-N | 11092 |
| AJH.28 | MOL000890 | (+)-alpha-Curcumene | 26.56378766 | 202.370 | 0.055919 | VMYXUZSZMNBRCN-AWEZNQCLSA-N | 3083834 |
| AJH.29 | MOL000922 | (R)-p-Menth-1-en-4-ol | 32.15585044 | 154.280 | 0.032284 | WRYLYDPHFGVWKC-JTQLQIEISA-N | 5325830 |
| AJH.30 | MOL000924 | Mnk | 17.6587517 | 170.330 | 0.025869 | KYWIYKKSMDLRDC-UHFFFAOYSA-N | 8163 |
| AJH.31 | MOL000930 | cis-.alpha.-Farnesene | 8.153141881 | 204.390 | 0.047425 | CXENHBSYCFFKJS-OXYODPPFSA-N | 5362889 |
| AJH.32 | MOL000971 | Ethylpalmitate | 18.98672237 | 284.540 | 0.13539 | XIRNKXNNONJFQO-UHFFFAOYSA-N | 12366 |
| AJH.33 | MOL000991 | cinnamaldehyde | 31.99042428 | 132.170 | 0.02266 | KJPRLNWUNMBNBZ-QPJJXVBHSA-N | 637511 |
| AJH.34 | MOL001132 | longipinene | 17.00748122 | 204.390 | 0.12477 | HICYDYJTCDBHMZ-SFDCQRBFSA-N | Not Available |
| AJH.35 | MOL001300 | PEL | 44.03418878 | 122.180 | 0.017357 | WRMNZCZEMHIOCP-UHFFFAOYSA-N | 6054 |
| AJH.36 | MOL001312 | 9-HEXADECENOIC ACID | 35.77585321 | 254.460 | 0.1 | SECPZKHBENQXJG-BQYQJAHWSA-N | 5282745 |
| AJH.37 | MOL001393 | myristic acid | 21.18117264 | 228.420 | 0.066784 | TUNFSRHWOTWDNC-UHFFFAOYSA-N | 11005 |
| AJH.38 | MOL001396 | PENTADECYLIC ACID | 20.18459315 | 242.450 | 0.081479 | WQEPLUUGTLDZJY-UHFFFAOYSA-N | 13849 |
| AJH.39 | MOL001434 | quercetin 3-o-rhamnopyranosyl_qt | 22.23668275 | 302.250 | 0.27568 | MICMWLZYOSRUAQ-UHFFFAOYSA-N | Not Available |
| AJH.40 | MOL001442 | phytol | 33.82439209 | 296.600 | 0.13342 | BOTWFXYSPFMFNR-PYDDKJGSSA-N | 5280435 |
| AJH.41 | MOL001556 | Isocaryophyllene | 27.30234813 | 204.390 | 0.089903 | NPNUFJAVOOONJE-FLFDDASRSA-N | 5281522 |
| AJH.42 | MOL001600 | copaene | 29.47338384 | 204.390 | 0.12401 | VLXDPFLIRFYIME-QRTUWBSPSA-N | Not Available |
| AJH.43 | MOL001663 | (4aS,6aR,6aS,6bR,8aR,10R,12aR,14bS)-10-hydroxy-2,2,6a,6b,9,9,12a-heptamethyl-1,3,4,5,6,6a,7,8,8a,10,11,12,13,14b-tetradecahydropicene-4a-carboxylic acid | 32.02801329 | 456.780 | 0.75713 | MIJYXULNPSFWEK-KDQGZELNSA-N | 11869658 |
| AJH.44 | MOL001862 | Cadalin | 12.96313369 | 198.330 | 0.078803 | VMOJIHDTVZTGDO-UHFFFAOYSA-N | 10225 |
| AJH.45 | MOL001891 | 9-methylenefluorene | 26.86705102 | 178.240 | 0.094202 | ZYASLTYCYTYKFC-UHFFFAOYSA-N | 78147 |
| AJH.46 | MOL002007 | myricitrin | 5.084579007 | 464.410 | 0.76702 | DCYOADKBABEMIQ-OWMUPTOHSA-N | 5281673 |
| AJH.47 | MOL002008 | myricetin | 13.74833165 | 318.250 | 0.31057 | IKMDFBPHZNJCSN-UHFFFAOYSA-N | 5281672 |
| AJH.48 | MOL002046 | hexanoic acid | 73.07519787 | 116.180 | 0.0090082 | FUZZWVXGSFPDMH-UHFFFAOYSA-N | 8892 |
| AJH.49 | MOL002063 | γ-selinene | 22.22798014 | 204.390 | 0.080871 | RMZHSBMIZBMVMN-GJZGRUSLSA-N | Not Available |
| AJH.50 | MOL002085 | alpha-Cubebene | 16.73208261 | 204.390 | 0.10854 | XUEHVOLRMXNRKQ-KHMAMNHCSA-N | Not Available |
| AJH.51 | MOL002365 | (s)-carvone | 47.42638254 | 150.240 | 0.029968 | ULDHMXUKGWMISQ-VIFPVBQESA-N | 16724 |
| AJH.52 | MOL002816 | Bergenin | 14.1130214 | 328.300 | 0.34195 | YWJXCIXBAKGUKZ-HJJNZUOJSA-N | 66065 |
| AJH.53 | MOL002835 | δ-cadinol | 17.12537144 | 204.390 | 0.077187 | FUCYIEXQVQJBKY-UKRRQHHQSA-N | 12306055 |
| AJH.54 | MOL002879 | Diop | 43.59332547 | 390.620 | 0.39247 | IJFPVINAQGWBRJ-UHFFFAOYSA-N | 33934 |
| AJH.55 | MOL002944 | (E)-Linalol pyranoxide | 44.25272307 | 170.280 | 0.043357 | BCTBAGTXFYWYMW-WPRPVWTQSA-N | 6428300 |
| AJH.56 | MOL002951 | 2,4-Dimethyl-2,4-heptadienal | 57.60895535 | 138.230 | 0.016162 | PHOBFHQBEYNKAL-XVYDYJIPSA-N | 5370090 |
| AJH.57 | MOL002972 | (4S)-1-methyl-4-(6-methylhepta-1,5-dien-2-yl)cyclohexene | 20.30158753 | 204.390 | 0.055521 | XZRVRYFILCSYSP-OAHLLOKOSA-N | 10104370 |
| AJH.58 | MOL002983 | Guasol | 51.59528313 | 124.150 | 0.019924 | LHGVFZTZFXWLCP-UHFFFAOYSA-N | 460 |
| AJH.59 | MOL003516 | ZINC00896812 | 33.13294198 | 128.240 | 0.011803 | OHEFFKYYKJVVOX-QMMMGPOBSA-N | 6971127 |
| AJH.60 | MOL003573 | calacorene | 16.19720206 | 200.350 | 0.078209 | CUUMXRBKJIDIAY-ZDUSSCGKSA-N | 12302243 |
| AJH.61 | MOL003582 | Prenol | 66.68681872 | 86.150 | 0.0042972 | ASUAYTHWZCLXAN-UHFFFAOYSA-N | 11173 |
| AJH.62 | MOL003745 | Belamcandol A | 18.32314422 | 362.610 | 0.33959 | GBCXPDPXQWPCTG-FPLPWBNLSA-N | 5321906 |
| AJH.63 | MOL003750 | 3-methoxy-5-[(Z)-pentadec-10-enyl]phenol | 18.67024427 | 332.580 | 0.27296 | NKOPRUNFJQCUCF-SREVYHEPSA-N | 10969651 |
| AJH.64 | MOL004175 | NSC733507 | 17.31848908 | 488.780 | 0.7121 | OXVUXGFZHDKYLS-QUFHAEKXSA-N | 471426 |
| AJH.65 | MOL004419 | globulol | 19.94481443 | 222.410 | 0.12056 | AYXPYQRXGNDJFU-IMNVLQEYSA-N | 11996452 |
| AJH.66 | MOL004647 | TDA | 22.31567357 | 214.390 | 0.054133 | SZHOJFHSIKHZHA-UHFFFAOYSA-N | 12530 |
| AJH.67 | MOL004673 | kaempferitrin | 8.161500283 | 578.570 | 0.78752 | PUPKKEQDLNREIM-NUEHWCDRSA-N | Not Available |
| AJH.68 | MOL004677 | ledol | 82.77515449 | 222.410 | 0.12057 | AYXPYQRXGNDJFU-BXCDGJFSSA-N | Not Available |
| AJH.69 | MOL004717 | (3R,4aR,8aR)-3-isopropenyl-5,8a-dimethyl-2,3,4,4a,7,8-hexahydro-1H-naphthalene | 23.862723 | 204.390 | 0.081045 | OZQAPQSEYFAMCY-QLFBSQMISA-N | 10856614 |
| AJH.70 | MOL004982 | 2,6,10-trimethyl-dodecane | 37.79984335 | 144.140 | 0.029582 | VOLMSPGWNYJHQQ-SCSAIBSYSA-N | Not Available |
| AJH.71 | MOL005235 | Embelin | 37.71811671 | 294.430 | 0.17501 | IRSFLDGTOHBADP-UHFFFAOYSA-N | 3218 |
| AJH.72 | MOL005303 | 7-Tetradecyne | 20.06596267 | 194.400 | 0.039931 | AFNWSIIBAYUTTL-UHFFFAOYSA-N | 141979 |
| AJH.73 | MOL005748 | 17020-04-1 | 9.527830007 | 468.840 | 0.73848 | DTHUXXMWYWKQKX-QXZXTIJDSA-N | 177801 |
| AJH.74 | MOL005749 | isopulegone | 64.3102207 | 152.260 | 0.029798 | RMIANEGNSBUGDJ-IUCAKERBSA-N | 44163790 |
| AJH.75 | MOL005855 | Tormentic acid | 11.4040733 | 488.780 | 0.71209 | OXVUXGFZHDKYLS-BLIWDXROSA-N | 73193 |
| AJH.76 | MOL005932 | Gaidic acid | 34.01809581 | 254.460 | 0.099837 | ZVRMGCSSSYZGSM-CCEZHUSRSA-N | 5282743 |
| AJH.77 | MOL006288 | α-cadinol | 31.0234434 | 222.410 | 0.093123 | LHYHMMRYTDARSZ-KBXIAJHMSA-N | 12302223 |
| AJH.78 | MOL006312 | Azulol | 15.14886167 | 198.330 | 0.073542 | FWKQNCXZGNBPFD-UHFFFAOYSA-N | 3515 |
| AJH.79 | MOL006735 | Furfuranol | 48.27030023 | 98.110 | 0.01009 | XPFVYQJUAUNWIW-UHFFFAOYSA-N | 7361 |
| AJH.80 | MOL007276 | VERATRIC ACID | 58.77464302 | 182.190 | 0.047524 | DAUAQNGYDSHRET-UHFFFAOYSA-N | 7121 |
| AJH.81 | MOL007909 | p-menth-4-en-3-one | 61.79167957 | 152.260 | 0.029683 | OAYBZGPDRAMDNF-QMMMGPOBSA-N | 12088058 |
| AJH.82 | MOL008318 | ardisiacrispin B | 5.565869251 | 1075.390 | 0.0248 | ZDIHSHLFPFGAGP-LLEYBADXSA-N | Not Available |
| AJH.83 | MOL008653 | Acetylfuran | 49.63293985 | 110.120 | 0.014332 | IEMMBWWQXVXBEU-UHFFFAOYSA-N | 14505 |
| AJH.84 | MOL009278 | Laricitrin | 35.38099156 | 332.280 | 0.34199 | CFYMYCCYMJIYAB-UHFFFAOYSA-N | 5282154 |
| AJH.85 | MOL009280 | 4H-1-Benzopyran-4-one, 2-(3-(beta-D-glucopyranosyloxy)-4-hydroxy-5-methoxyphenyl)-3,5,7-trihydroxy- | 20.96138622 | 494.440 | 0.82774 | RUJHFBFKZCYVLZ-ROSPJSJWSA-N | 5487255 |
| AJH.86 | MOL009667 | 11Z-hexadecenoic acid | 35.77585321 | 254.460 | 0.10016 | JGMYDQCXGIMHLL-WAYWQWQTSA-N | 5312414 |
| AJH.87 | MOL010127 | Hexyl acetate | 13.27883578 | 144.240 | 0.015933 | AOGQPLXWSUTHQB-UHFFFAOYSA-N | 8908 |
| AJH.88 | MOL010930 | 10,10-dimethyl-2,6-bis(methylene)-dicyclo[7.2.0]undecane | 31.96686154 | 204.390 | 0.090014 | PMMLIVYPEUJENN-KGLIPLIRSA-N | Not Available |
| AJH.89 | MOL010931 | (4R)-3,5,5-trimethyl-4-[(E,3R)-3-[(2R,3R,4S,5S,6R)-3,4,5-trihydroxy-6-(hydroxymethyl)oxan-2-yl]oxybut-1-enyl]cyclohex-2-en-1-one | 12.71305717 | 370.490 | 0.33513 | SZOPSAFLRCYJCX-ITEOXOHJSA-N | 9820702 |
| AJH.90 | MOL010932 | (4R)-4-[(E,3R)-3-hydroxybut-1-enyl]-3,5,5-trimethylcyclohex-2-en-1-one | 37.71551132 | 208.330 | 0.06536 | MDCGEAGEQVMWPE-SXGMEYSMSA-N | 13857510 |
| AJH.91 | MOL010933 | ardisianoside K | 1.84978818 | 1075.390 | 0.023985 | WLCKGGTZSALHSX-AQYMXCBLSA-N | Not Available |
| AJH.92 | MOL010934 | ardisianoside K_qt | 31.97790441 | 472.780 | 0.62575 | DLWHFHBRYSITBI-WGIDJJHTSA-N | Not Available |
| AJH.93 | MOL010935 | ardisicrenoside A | 5.608271113 | 1077.410 | 0.024787 | LTTSWSWZQNISIB-LLEYBADXSA-N | Not Available |
| AJH.94 | MOL010936 | ardisianoside F_qt | 16.77799266 | 474.800 | 0.62912 | AAXANNQJCBNQMB-GFRLSDCRSA-N | Not Available |
| AJH.95 | MOL010937 | ardisicrenoside G | 1.946435424 | 1091.390 | 0.035961 | LQOKWJKICXGHMS-JLXKNENYSA-N | Not Available |
| AJH.96 | MOL010938 | ardisicrenoside G_qt | 8.770735214 | 488.780 | 0.72302 | MLHMWJANFQYUES-ZFZJRSJFSA-N | Not Available |
| AJH.97 | MOL010939 | 2-methyl-5-[(Z)-tridec-8-enyl]resorcinol | 3.423394905 | 304.520 | 0.20329 | AAFWHAONVYRTMW-SREVYHEPSA-N | 6440454 |
| AJH.98 | MOL010940 | Ardisinol I | 3.423394905 | 304.520 | 0.20337 | AAFWHAONVYRTMW-VOTSOKGWSA-N | 5319846 |
| AJH.99 | MOL010941 | Ardisinol II | 3.43576143 | 290.490 | 0.17878 | ILUMNMFPGSFYMK-AATRIKPKSA-N | 5319856 |
| AJH.100 | MOL010942 | ardisinol | 3.506678899 | 304.520 | 0.15386 | WPISTRPEOKIHBW-KAMXEHQASA-N | Not Available |
| AJH.101 | MOL010943 | 1-(4-Hydroxy-3-methoxyphenyl)-3,5-diacetoxytane | 7.190084593 | 352.470 | 0.24056 | AUBPDZJRJKZQEX-DLBZAZTESA-N | Not Available |
| AJH.102 | MOL010944 | phenylmethanediol | 81.15961908 | 124.150 | 0.019261 | SNGARVZXPNQWEY-UHFFFAOYSA-N | 427890 |
| AJH.103 | MOL010945 | (2-hydroxyphenyl)methyl benzoate | 79.02599998 | 228.260 | 0.10458 | NLKAKOQQZSQWMO-UHFFFAOYSA-N | 12731045 |
| AJH.104 | MOL010946 | PHENYLACETONE | 39.65701303 | 134.190 | 0.022199 | QCCDLTOVEPVEJK-UHFFFAOYSA-N | 7678 |
| AJH.105 | MOL010947 | (Z)-calamenene | 17.75088457 | 202.370 | 0.077611 | PGTJIOWQJWHTJJ-STQMWFEESA-N | 6429077 |
| AJH.106 | MOL010948 | caryophyllenyl alcohol | 45.85640454 | 222.410 | 0.10324 | YWXBTHXTAMGLEW-OKMCCBHRSA-N | Not Available |
| AJH.107 | MOL010949 | Cyclamin | 6.698445065 | 1223.520 | 0.016572 | JPEQATLMKATGAQ-JOONIPAFSA-N | Not Available |
| AJH.108 | MOL010950 | cyclamin_qt | 15.92758751 | 472.780 | 0.63098 | UBWMMEPLQFWYCH-GFRLSDCRSA-N | Not Available |
| AJH.109 | MOL010951 | Methylsyringol | 36.35967562 | 168.210 | 0.037809 | CRUILBNAQILVHZ-UHFFFAOYSA-N | 12462 |
| AJH.110 | MOL010952 | triterpenoid glycoside 1 | 5.595836285 | 1075.390 | 0.024814 | WEEYYXZPPZELEU-QSSFKJCUSA-N | Not Available |
| AJH.111 | MOL010953 | triterpenoid glycoside 1_qt | 34.11143581 | 472.780 | 0.63118 | UBWMMEPLQFWYCH-GZAMYCOFSA-N | Not Available |
| AJH.112 | MOL010954 | Tropyliden | 45.5680034 | 92.150 | 0.0076811 | CHVJITGCYZJHLR-UHFFFAOYSA-N | 11000 |
| AJH.113 | MOL010955 | decahydro-2,2,4,8-tetramethyl-4,8-methano-azulen-9-ol | 129.75975 | 222.410 | 0.12109 | MJYUBUQHKCAJQR-LYOXDTQASA-N | Not Available |
| AJH.114 | MOL010956 | (3R,5S)-3,5-dimethyltetrahydrofuran-2-one | 83.94599865 | 114.160 | 0.015215 | IYJMJJJBGWGVKX-UHNVWZDZSA-N | 5324164 |
| AJH.115 | MOL010957 | (Z)-Ethyl cinnamate | 37.28292696 | 176.230 | 0.042149 | KBEBGUQPQBELIU-HJWRWDBZSA-N | 5284656 |
| AJH.116 | MOL010958 | (-)-Gallocatechol gallate | 3.013665178 | 458.400 | 0.77375 | WMBWREPUVVBILR-NQIIRXRSSA-N | 199472 |
| AJH.117 | MOL010959 | ilexol | 9.381947535 | 426.800 | 0.76236 | TZVDWGXUGGUMCE-MRUPOYLQSA-N | Not Available |
| AJH.118 | MOL010960 | 1H-benzocycloheptene,2,4a,5,6,7,8,9,9a-octahydro-3,3,5-trimethyl-9-methylene-,(4aS-cis)- | 36.59148206 | 205.400 | 0.079153 | MKABUZPLXHKDBL-MCIONIFRSA-N | Not Available |
| AJH.119 | MOL010961 | Kaempferol 3-O-rhamnoside | 5.309553485 | 432.410 | 0.69616 | SOSLMHZOJATCCP-LYHQQHOMSA-N | Not Available |
| AJH.120 | MOL010962 | kaempferol rhamnose | 17.73454293 | 432.410 | 0.69617 | SOSLMHZOJATCCP-QBWVUYDZSA-O | Not Available |
| AJH.121 | MOL010963 | 5-hydroxy-2-(4-hydroxyphenyl)-3,7-bis[[(2R,3S,4R,5R,6S)-3,4,5-trihydroxy-6-methyl-tetrahydropyran-2-yl]oxy]chromone | 8.161500283 | 578.570 | 0.78653 | PUPKKEQDLNREIM-FLMWOZIFSA-N | 11444588 |
| AJH.122 | MOL010964 | MAESANIN | 42.77499821 | 362.560 | 0.35103 | WVHQJXPRVZBEFP-SREVYHEPSA-N | 5384838 |
| AJH.123 | MOL010965 | Norbergenin | 13.39830932 | 314.270 | 0.31051 | GDYGAIKPBLFCKR-YWQRSDGBSA-N | 73192 |
| AJH.124 | MOL010966 | Phthalic acid, isobutyl octyl ester | 22.08258954 | 334.500 | 0.23999 | VUZMWXPSHBFURN-UHFFFAOYSA-N | 6423815 |
| AJH.125 | MOL010968 | primulanin | 8.130139548 | 899.200 | 0.034121 | JFAKXPCWZPQXLP-KASLOCEDSA-N | Not Available |
| AJH.126 | MOL010969 | Propyl isothiocyanate | 66.2323249 | 101.190 | 0.0033037 | KKASGUHLXWAKEZ-UHFFFAOYSA-N | 69403 |
| AJH.127 | MOL010970 | (2S)-2-methylthiirane | 15.03695846 | 74.160 | 0.0046261 | MBNVSWHUJDDZRH-VKHMYHEASA-N | 21766881 |
| AJH.128 | MOL010972 | Quercitrin-2'-gallate | 3.013123967 | 600.520 | 0.68318 | KTTNFIOZYNBKEY-VDSUVTDHSA-N | 5320864 |
| AJH.129 | MOL010973 | Rapanone | 34.1530898 | 322.490 | 0.23812 | AMKNOBHCKRZHIO-UHFFFAOYSA-N | 100659 |
| AJH.130 | MOL010974 | tri-O-methylnorbergenin | 33.17034587 | 356.360 | 0.40979 | RGHGUQJYNLPWPT-GVMTXOEMSA-N | 44584185 |
| AJH.131 | MOL010975 | triterpene glycoside 4 | 6.805562081 | 1207.520 | 0.017081 | ULVYRJVZULDUDV-UZZTZXPTSA-N | Not Available |
| AJH.132 | MOL010976 | triterpene glycoside 4_qt | 41.39996748 | 472.780 | 0.63122 | UBWMMEPLQFWYCH-RMXBMUOASA-N | Not Available |
| AJH.133 | MOL010977 | triterpenoid glycoside 2 | 5.732895164 | 1105.470 | 0.021528 | BSFAGLXMAHFGOM-PMTOMCKTSA-N | Not Available |
| AJH.134 | MOL010978 | triterpenoid glycoside 2_qt | 19.35307591 | 502.860 | 0.56439 | NEUGPAAYCHUDDH-JZGKDRKPSA-N | Not Available |
| AJH.135 | MOL010979 | 2,4-Dimethylpentan-2-ol | 64.00442495 | 116.230 | 0.01158 | FMLSQAUAAGVTJO-UHFFFAOYSA-N | 12235 |
| AJH.136 | MOL010980 | triterpenoid glycoside 3 | 5.553822556 | 1091.390 | 0.023143 | UVFTXZYUPYKWOF-YEOYDMOFSA-N | Not Available |
| AJH.137 | MOL010981 | triterpenoid glycoside 3_qt | 44.03723066 | 488.780 | 0.6009 | WTFUHAGPAIREPL-RYZHOHRSSA-N | Not Available |
| AJH.138 | MOL010982 | 2,5-dihydroxy-3-[(10Z)-pentadec-10-en-1-yl][1,4]benzoquinone | 34.73921496 | 460.770 | 0.60316 | OZOJZESTKHHHQP-WAYWQWQTSA-N | Not Available |
| AJH.139 | MOL010983 | 2,5-Dihydroxy-3-[(10Z)-pentadec-10-en-1-yl]cyclohexa-2,5-diene-1,4-dione | 37.3018181 | 348.530 | 0.32034 | YRIWERDENGDRIR-WAYWQWQTSA-N | Not Available |
| AJH.140 | MOL010984 | 2H-benz[e]indol-2-one,1,3,3a,4,5,9b-hexahydro- | 44.55844229 | 187.260 | 0.094409 | JVXUCAOHOJQAMI-MNOVXSKESA-N | Not Available |
| AJH.141 | MOL010985 | 2-hydroxy-5-methoxy-3-pentadecaenylbenzoquinone | 41.61117697 | 362.560 | 0.3151 | GSFLASJBASRJKZ-HYPNTESJSA-N | Not Available |
| AJH.142 | MOL010986 | (7E)-9-hydroxymegastigma-4, 7-dien-3-on-9-O-beta-D-glucopyranoside | 12.11853161 | 370.490 | 0.33514 | SZOPSAFLRCYJCX-UZOVYFIJSA-N | 38346679 |
| AJH.143 | MOL010987 | (7E)-9-hydroxymegastigma-4, 7-dien-3-on-9-O-beta-D-glucopyranoside_qt | 19.15490489 | 208.330 | 0.065349 | MDCGEAGEQVMWPE-PLWSPRCSSA-N | Not Available |
| AJH.144 | MOL010988 | 2-methyl-5-(1-methylethylidene)-cyclo-hexanone | 48.34482584 | 152.260 | 0.029504 | KCMPNSMMFORCER-QMMMGPOBSA-N | 14440745 |
| AJH.145 | MOL010989 | 2-Methylcardol monoene | 3.340373272 | 332.580 | 0.2727 | LDBPJTXLCRXBIJ-HJWRWDBZSA-N | 6452209 |
| AJH.146 | MOL010990 | Isomatsutakeol | 42.12306847 | 128.240 | 0.011157 | AYQPVPFZWIQERS-VOTSOKGWSA-N | 5318599 |
| AJH.147 | MOL010991 | (2S)-pentan-2-ol | 46.98183767 | 88.170 | 0.0041482 | JYVLIDXNZAXMDK-YFKPBYRVSA-N | 2724896 |
| AJH.148 | MOL010992 | 3,4,5-Trimethoxybenzaldehyde | 31.10928288 | 196.220 | 0.055221 | OPHQOIGEOHXOGX-UHFFFAOYSA-N | 6858 |
| AJH.149 | MOL010993 | (E)-3,4-dimethylhex-3-en-2-one | 41.66486702 | 126.220 | 0.014174 | WRHRFVOAEDXVPC-VOTSOKGWSA-N | 5363236 |
| AJH.150 | MOL010994 | 3beta-O-(alpha-L-rhamnopyranosyl-(1-2)-beta-D-glucopyranosyl-(1-4)-alpha-L-arabinopynanosyl)cyclamiretin A | 7.391949649 | 925.240 | 0.030202 | DMGYUBDDNMOPDH-PRTYXVATSA-N | Not Available |
| AJH.151 | MOL010995 | 3beta-O-(alpha-L-rhamnopyranosyl-(1-2)-beta-D-glucopyranosyl-(1-4)-alpha-L-arabinopynanosyl)cyclamiretin A_qt | 15.56252277 | 484.790 | 0.60072 | DDDQOLWDMXGELK-RGBYWQDLSA-N | Not Available |
| AJH.152 | MOL010996 | 3-hexen-2-one(E) | 49.72330643 | 98.160 | 0.0062016 | LPCWMYHBLXLJJQ-SNAWJCMRSA-N | 5367744 |
| AJH.153 | MOL010997 | 3-Hexynol | 55.21410032 | 98.160 | 0.0057496 | ODEHKVYXWLXRRR-UHFFFAOYSA-N | 66083 |
| AJH.154 | MOL010998 | 3-methoxy-1-ethenyl-cyclopentene | 64.75599785 | 124.200 | 0.016963 | DYJCJVOTPBIIHK-MRVPVSSYSA-N | Not Available |
| AJH.155 | MOL010999 | 2-isopropyl-3-methyl-1-cyclohex-2-enone | 63.8748657 | 152.260 | 0.030712 | DODMAZCOMJAOQC-UHFFFAOYSA-N | 10103344 |
| AJH.156 | MOL011000 | (E,Z)-alpha-farnesene | 7.664961892 | 204.390 | 0.047288 | CXENHBSYCFFKJS-DZKMRSEMSA-N | 5353086 |
| AJH.157 | MOL011001 | Mesityloxid | 50.93191434 | 98.160 | 0.0069009 | SHOJXDKTYKFBRD-UHFFFAOYSA-N | 8858 |
| AJH.158 | MOL011002 | 5-ethoxy-2-hydroxy-3-[(10Z)-pentadec-10-en-1-yl][1,4]benzoquinone | 42.77105866 | 376.590 | 0.38323 | YQDOWDWMMZKLQR-FPLPWBNLSA-N | Not Available |
| AJH.159 | MOL011003 | 5-ethoxy-2-hydroxy-3-[(8Z)-tridec-8-en-1-yl][1,4]benzoquinone | 43.22748811 | 348.530 | 0.2961 | XELCDMYKAGKICS-FPLPWBNLSA-N | Not Available |
| AJH.160 | MOL011004 | (E)-5-methyloct-5-en-1-ol | 33.23915694 | 142.270 | 0.015266 | SAGBALOKGFKCER-RMKNXTFCSA-N | 6536854 |
| AJH.161 | MOL011005 | 6-O-Methyl catalpol | 8.883047819 | 376.400 | 0.4812 | CQHVYUDLQLYNAI-FJAHIDBWSA-N | Not Available |
| AJH.162 | MOL011006 | 6-O-Methyl catalpol_qt | 12.61362746 | 214.240 | 0.11448 | NZBCOJZIKRIKRS-MXTLHFIKSA-N | Not Available |
| AJH.163 | MOL011007 | 1-(1-ethoxyethoxy)hexane | 46.42586455 | 174.320 | 0.025238 | YJYLJPSYBMNHTG-SNVBAGLBSA-N | Not Available |
| AJH.164 | MOL011008 | 8-oxabicyclo[5.1.0]octane | 45.77201167 | 112.190 | 0.02068 | MLOZFLXCWGERSM-KNVOCYPGSA-N | 642973 |
| AJH.165 | MOL011009 | alloaromadendrene | 50.62208165 | 204.390 | 0.10417 | ITYNGVSTWVVPIC-RYMFRWLXSA-N | Not Available |
| AJH.166 | MOL011010 | ardimerin digallate | 3.013720692 | 960.810 | 0.097565 | DXKGWDISVOGUDQ-HQWPKFJNSA-N | Not Available |
| AJH.167 | MOL011011 | ardimerin | 6.138566995 | 656.600 | 0.55283 | ORAUCQAGCNPIAZ-BQGIUCMVSA-N | 44423020 |
| AJH.168 | MOL011012 | 1-(1-ethoxyethoxy)pentane | 25.5487702 | 160.290 | 0.019621 | QMLYOIJQQWWNKE-SECBINFHSA-N | Not Available |
| AJH.169 | MOL011013 | ardisiamamilloside C | 1.91211056 | 1077.410 | 0.038215 | FUEJSDQPLRTIHO-DXBHXUPOSA-N | Not Available |
| AJH.170 | MOL011014 | ardisiamamilloside C_qt | 14.25279373 | 474.800 | 0.7381 | MCZHPIHVRHTCIM-DIEVBXJSSA-N | Not Available |
| AJH.171 | MOL011015 | ardisiamamilloside F | 5.516619116 | 1091.390 | 0.023131 | GBIVVEOMDGPTFX-KORAFSQNSA-N | Not Available |
| AJH.172 | MOL011016 | ardisiamamilloside F_qt | 17.72433705 | 488.780 | 0.60055 | WTFUHAGPAIREPL-RDFYQBLMSA-N | Not Available |
| AJH.173 | MOL011017 | ardisiamamilloside H | 13.78097675 | 911.210 | 0.031792 | LDHINNKHMNWJCX-LOFXXVTMSA-N | Not Available |
| AJH.174 | MOL011018 | ardisiamamilloside H_qt | 17.60921516 | 470.760 | 0.6323 | RNJXNQHGXAEMIC-LQVIMQQQSA-N | Not Available |
| AJH.175 | MOL011019 | ardisianone A | 44.22012847 | 346.560 | 0.24889 | WNZXOLJKBYUFDZ-ZFYPLVIYSA-N | Not Available |
| AJH.176 | MOL011020 | ardisianone B | 60.90178195 | 334.500 | 0.19783 | TYEYBRGUMMKRGI-ZHANPKHBSA-N | Not Available |
| AJH.177 | MOL011021 | ardisianoside A | 7.565680552 | 1545.860 | 0.0059917 | OPLKRQJWFNZARS-MZTARDFQSA-N | Not Available |
| AJH.178 | MOL011022 | ardisianoside C_qt | 20.35119895 | 458.800 | 0.656 | LFBHIAAGGTTWAT-DNSXXVLGSA-N | Not Available |
| AJH.179 | MOL011023 | ardisianoside B | 7.436986618 | 1371.700 | 0.0093404 | OCSVOVNGBJMZTD-JKDSUZQNSA-N | Not Available |
| AJH.180 | MOL011024 | ardisianoside C | 17.73740163 | 899.250 | 0.03426 | HERCSTJVHFDRGB-JTWYRERBSA-N | Not Available |
| AJH.181 | MOL011025 | ardisianoside D | 7.414177857 | 897.230 | 0.034832 | PLIZSMTWYQHOFY-ZAEULYPRSA-N | Not Available |
| AJH.182 | MOL011026 | ardisianoside D_qt | 16.57104894 | 470.810 | 0.62697 | KMZIUMFELNYSTH-GZMIIRINSA-N | Not Available |
| AJH.183 | MOL011027 | ardisianoside E | 6.541931615 | 931.250 | 0.048663 | QMDCUVOUEVYVPH-CDLHYRBUSA-N | Not Available |
| AJH.184 | MOL011028 | 1(2H)-naphthalenone,3,4,4a,5,8,8a-hexahydro-8a-methyl-,trans- | 29.21258574 | 164.270 | 0.055218 | BOFYYUXJGLWGLT-ONGXEEELSA-N | 12512776 |
| AJH.185 | MOL011029 | ardisianoside F | 6.770581964 | 1225.540 | 0.016566 | OCLRKHSJKXGXRY-JOONIPAFSA-N | Not Available |
| AJH.186 | MOL011030 | ardisianoside G | 5.618404458 | 1063.380 | 0.026737 | LZUMOKWHTUFQPN-NLJCTUQTSA-N | Not Available |
| AJH.187 | MOL011031 | ardisianoside G_qt | 16.96563034 | 460.770 | 0.65962 | RVXZVZJOPGSXKL-IKVNGRJPSA-N | Not Available |
| AJH.188 | MOL011032 | ardisianoside H | 7.563607782 | 1547.830 | 0.0059939 | WCJAQHZDGNSJBM-BNJUBNBQSA-N | Not Available |
| AJH.189 | MOL011033 | ardisianoside H_qt | 17.2695094 | 472.780 | 0.62966 | YNVKKFBRPWLSCJ-KSGIOOATSA-N | Not Available |
| AJH.190 | MOL011034 | ardisianoside I | 5.65296114 | 1091.390 | 0.023591 | QUPKOBZUSQZXJV-KORAFSQNSA-N | Not Available |
| AJH.191 | MOL011035 | ardisianoside I_qt | 17.73005162 | 488.780 | 0.60137 | HHYLXGZNUCELFI-RDFYQBLMSA-N | Not Available |
| AJH.192 | MOL011036 | ardisianoside J | 5.778578269 | 1089.370 | 0.023601 | SYINIHYNGYHPRE-KORAFSQNSA-N | Not Available |
| AJH.193 | MOL011037 | ardisianoside J_qt | 21.2427139 | 486.760 | 0.60293 | WBPFBIUINMGVJM-RDFYQBLMSA-N | Not Available |
| CRP.1 | MOL000001 | anthocyanidin | 45.59739165 | 251.220 | 0.18968 | YLJQHJNWIHUOKW-UHFFFAOYSA-N | Not Available |
| CRP.2 | MOL000057 | DIBP | 49.6340827 | 278.380 | 0.13067 | MGWAVDBGNNKXQV-UHFFFAOYSA-N | 6782 |
| CRP.3 | MOL000118 | (L)-alpha-Terpineol | 48.79777273 | 154.280 | 0.030772 | WUOACPNHFRMFPN-SECBINFHSA-N | 443162 |
| CRP.4 | MOL000121 | Decanal | 29.80505997 | 156.300 | 0.020124 | KSMVZQYAVGTKIV-UHFFFAOYSA-N | 8175 |
| CRP.5 | MOL000125 | (-)-alpha-Pinene | 46.24981501 | 136.260 | 0.052565 | GRWFGVWFFZKLTI-IUCAKERBSA-N | 440968 |
| CRP.6 | MOL000127 | Neral | 19.48363567 | 152.260 | 0.020741 | WTEVQBCEXWBHNA-YFHOEESVSA-N | 643779 |
| CRP.7 | MOL000168 | ()-2-Carene | 46.69280884 | 136.260 | 0.042621 | IBVJWOMJGCHRRW-BDAKNGLRSA-N | 78249 |
| CRP.8 | MOL000198 | (R)-linalool | 39.80430084 | 154.280 | 0.022686 | CDOSHBSSFJOMGT-JTQLQIEISA-N | 443158 |
| CRP.9 | MOL000201 | p-Ocimene | 15.06052114 | 136.260 | 0.015199 | IHPKGUQCSIINRJ-CSKARUKUSA-N | 5281553 |
| CRP.10 | MOL000259 | o-Thymol | 43.28127042 | 150.240 | 0.029778 | RECUKUPTGUEGMW-UHFFFAOYSA-N | 10364 |
| CRP.11 | MOL000268 | (1S,5S)-1-isopropyl-4-methylenebicyclo[3.1.0]hexane | 46.2051091 | 136.260 | 0.043544 | NDVASEGYNIMXJL-UWVGGRQHSA-N | 11051711 |
| CRP.12 | MOL000305 | lauric acid | 23.58793922 | 200.360 | 0.043637 | POULHZVOKOAJMA-UHFFFAOYSA-N | 3893 |
| CRP.13 | MOL000359 | sitosterol | 36.91390583 | 414.790 | 0.7512 | KZJWDPNRJALLNS-ZFVHJZABSA-N | 12303645 |
| CRP.14 | MOL000597 | Neryl acetate | 57.46587185 | 196.320 | 0.041454 | HIGQPQRQIQDZMP-FLIBITNWSA-N | 1549025 |
| CRP.15 | MOL000608 | ()-Terpinen-4-ol | 81.40667188 | 154.280 | 0.032323 | WRYLYDPHFGVWKC-SNVBAGLBSA-N | 2724161 |
| CRP.16 | MOL000615 | delta-amorphene | 17.94609655 | 204.390 | 0.077173 | FUCYIEXQVQJBKY-ZFWWWQNUSA-N | 441005 |
| CRP.17 | MOL000635 | vanillin | 51.99600777 | 152.160 | 0.032596 | MWOOGOJBHIARFG-UHFFFAOYSA-N | 1183 |
| CRP.18 | MOL000696 | β-terpineol | 47.88836394 | 154.280 | 0.031028 | RUJPNZNXGCHGID-MGCOHNPYSA-N | 8748 |
| CRP.19 | MOL000710 | OYA | 19.06821324 | 128.240 | 0.011222 | NUJGJRNETVAIRJ-UHFFFAOYSA-N | 454 |
| CRP.20 | MOL000748 | HMF | 45.06610249 | 126.120 | 0.019427 | NOEGNKMFWQHSLB-UHFFFAOYSA-N | 237332 |
| CRP.21 | MOL000771 | p-coumaric acid | 43.29024064 | 164.170 | 0.039118 | NGSWKAQJJWESNS-ZZXKWVIFSA-N | 637542 |
| CRP.22 | MOL000860 | stearic acid | 17.82542938 | 284.540 | 0.14086 | QIQXTHQIDYTFRH-UHFFFAOYSA-N | 5281 |
| CRP.23 | MOL000869 | Henicosane | 8.412905294 | 296.650 | 0.15364 | FNAZRRHPUDJQCJ-UHFFFAOYSA-N | 12403 |
| CRP.24 | MOL000922 | (R)-p-Menth-1-en-4-ol | 32.15585044 | 154.280 | 0.032284 | WRYLYDPHFGVWKC-JTQLQIEISA-N | 5325830 |
| CRP.25 | MOL001055 | 5-isopropyl-2-methylbicyclo[3.1.0]hex-2-ene | 47.19217569 | 136.260 | 0.043509 | KQAZVFVOEIRWHN-NXEZZACHSA-N | 637518 |
| CRP.26 | MOL001101 | alpha-Ocimene | 21.43137325 | 136.260 | 0.015292 | XJPBRODHZKDRCB-CSKARUKUSA-N | 5320249 |
| CRP.27 | MOL001110 | cis-beta-Ocimene | 25.38399655 | 136.260 | 0.015124 | IHPKGUQCSIINRJ-NTMALXAHSA-N | 5320250 |
| CRP.28 | MOL001797 | (2S)-7-[(2S,3R,4S,5S,6R)-4,5-dihydroxy-6-methylol-3-[(2S,3R,4R,5R,6S)-3,4,5-trihydroxy-6-methyl-tetrahydropyran-2-yl]oxy-tetrahydropyran-2-yl]oxy-5-hydroxy-2-(3-hydroxy-5-methoxy-phenyl)chroman-4-one | 11.1740343 | 610.620 | 0.69663 | TWAZWVPPDIUVOD-UZRWAPQLSA-N | 24721685 |
| CRP.29 | MOL002029 | ()-Cuparene | 38.26308654 | 202.370 | 0.07494 | SLKPBCXNFNIJSV-HNNXBMFYSA-N | 86895 |
| CRP.30 | MOL002050 | Isovanillic acid | 39.42393771 | 168.160 | 0.041046 | LBKFGYZQBSGRHY-UHFFFAOYSA-N | 12575 |
| CRP.31 | MOL002092 | Antioxidant No. 33 | 26.74299234 | 206.360 | 0.062536 | ICKWICRCANNIBI-UHFFFAOYSA-N | 7311 |
| CRP.32 | MOL002095 | DEP | 52.18551816 | 222.260 | 0.074284 | FLKPEMZONWLCSK-UHFFFAOYSA-N | 6781 |
| CRP.33 | MOL002138 | p-Cymen-8-ol | 32.26487597 | 150.240 | 0.031428 | XLPDVYGDNRIQFV-UHFFFAOYSA-N | 14529 |
| CRP.34 | MOL002336 | farnesane | 3.13243451 | 212.470 | 0.044511 | YFHFHLSMISYUAQ-CABCVRRESA-N | Not Available |
| CRP.35 | MOL002456 | [(3R)-3,7-dimethyloct-6-enyl] butanoate | 21.03051946 | 226.400 | 0.059422 | XQPZQXTWYZAXAK-CYBMUJFWSA-N | 23616651 |
| CRP.36 | MOL002868 | 1-Undecyne | 33.99069151 | 152.310 | 0.019331 | YVSFLVNWJIEJRV-UHFFFAOYSA-N | 75249 |
| CRP.37 | MOL002944 | (E)-Linalol pyranoxide | 44.25272307 | 170.280 | 0.043357 | BCTBAGTXFYWYMW-WPRPVWTQSA-N | 6428300 |
| CRP.38 | MOL003450 | dodec-2-enal | 31.95214365 | 182.340 | 0.034022 | SSNZFFBDIMUILS-ZHACJKMWSA-N | 5283361 |
| CRP.39 | MOL003508 | Antak | 16.85194372 | 158.320 | 0.019682 | MWKFXSUHUHTGQN-UHFFFAOYSA-N | 8174 |
| CRP.40 | MOL003538 | ()-Ledene | 51.84417883 | 204.390 | 0.10403 | WGTRJVCFDUCKCM-FMKGYKFTSA-N | 10910653 |
| CRP.41 | MOL003949 | Dimethyl anthranilate | 65.86883769 | 165.210 | 0.037148 | GVOWHGSUZUUUDR-UHFFFAOYSA-N | 6826 |
| CRP.42 | MOL004328 | naringenin | 59.29389773 | 272.270 | 0.21128 | FTVWIRXFELQLPI-ZDUSSCGKSA-N | 439246 |
| CRP.43 | MOL005100 | 5,7-dihydroxy-2-(3-hydroxy-4-methoxyphenyl)chroman-4-one | 47.73643694 | 302.300 | 0.27226 | AIONOLUJZLIMTK-CQSZACIVSA-N | 676152 |
| CRP.44 | MOL005577 | undecanal | 22.90445698 | 170.330 | 0.02616 | KMPQYAYAQWNLME-UHFFFAOYSA-N | 8186 |
| CRP.45 | MOL005811 | Hepta-3 | 23.90506891 | 432.460 | 0.58111 | SSXJHQZOHUYEGD-UHFFFAOYSA-N | 150893 |
| CRP.46 | MOL005812 | naringin | 6.917815897 | 580.590 | 0.77847 | DFPMSGMNTNDNHN-ZPHOTFPESA-N | 442428 |
| CRP.47 | MOL005813 | Germacrene A | 19.21188596 | 204.390 | 0.05718 | XMRKUJJDDKYUHV-ZCGSDFCLSA-N | 6440527 |
| CRP.48 | MOL005814 | tangeretin | 21.3751908 | 372.400 | 0.42958 | ULSUXBXHSYSGDT-UHFFFAOYSA-N | 68077 |
| CRP.49 | MOL005815 | Citromitin | 86.90404672 | 404.450 | 0.51439 | LTRBUBSPQISFFL-CQSZACIVSA-N | Not Available |
| CRP.50 | MOL005816 | alpha-Sinensal | 57.79230283 | 218.370 | 0.06176 | PFSTYGCNVAVZBK-JQGMZEBDSA-N | 5281534 |
| CRP.51 | MOL005817 | 2-(2-butynyl)-cyclohexanone | 47.77602354 | 150.240 | 0.028165 | JNULPQQVLJEQMV-VIFPVBQESA-N | Not Available |
| CRP.52 | MOL005818 | 2,5,5-trimethylhepta-1,6-diene | 44.33635645 | 138.280 | 0.017873 | IFNFARVJUNNPDT-UHFFFAOYSA-N | 534952 |
| CRP.53 | MOL005819 | 2,6,11-trimethyldodecane | 14.19188228 | 212.470 | 0.045164 | FONXOARHSFUBAN-OAHLLOKOSA-N | Not Available |
| CRP.54 | MOL005820 | 22410-74-8 | 39.90616556 | 154.280 | 0.020413 | JSMKSZJPQZMEHN-JXMROGBWSA-N | 5365825 |
| CRP.55 | MOL005821 | (2S)-2-ethoxypentane | 39.60499839 | 116.230 | 0.0083132 | XFKPOLRDQWCGPV-ZETCQYMHSA-N | 11804727 |
| CRP.56 | MOL005822 | 3-decyn-2-ol | 39.32692591 | 154.280 | 0.020227 | PGIQIBRWODQISW-JTQLQIEISA-N | Not Available |
| CRP.57 | MOL005823 | Isoprenol | 65.72037003 | 86.150 | 0.0043431 | CPJRRXSHAYUTGL-UHFFFAOYSA-N | 12988 |
| CRP.58 | MOL005824 | 4-ACETYLBENZOIC ACID | 28.65761211 | 164.170 | 0.039882 | QBHDSQZASIBAAI-UHFFFAOYSA-N | 11470 |
| CRP.59 | MOL005825 | 6-Hepten-1-ol | 21.3432229 | 114.210 | 0.008194 | UFULDTPDHIRNGS-UHFFFAOYSA-N | 543123 |
| CRP.60 | MOL005826 | cis-2,6-Dimethyl-2,6-octadiene | 21.09988071 | 138.280 | 0.014856 | MZPDTOMKQCMETI-YHYXMXQVSA-N | 5352478 |
| CRP.61 | MOL005827 | Eufin | 0.257346935 | 118.150 | 0.0095066 | OIFBSDVPJOWBCH-UHFFFAOYSA-N | 7766 |
| CRP.62 | MOL005828 | nobiletin | 61.66943932 | 402.430 | 0.51652 | MRIAQLRQZPPODS-UHFFFAOYSA-N | 72344 |
| CRP.63 | MOL005829 | Undecyl acetate | 19.92186533 | 214.390 | 0.053706 | CKQGCFFDQIFZFA-UHFFFAOYSA-N | 15605 |
| CRP.64 | MOL007930 | hesperidin | 13.33460023 | 610.620 | 0.66749 | QUQPHWDTPGMPEX-QJBIFVCTSA-N | 10621 |
| CF.1 | MOL000027 | alpha-Curcumene | 4.677265254 | 202.370 | 0.055937 | VMYXUZSZMNBRCN-CQSZACIVSA-N | 442360 |
| CF.2 | MOL000034 | 2-[(1R,3S,4S)-3-isopropenyl-4-methyl-4-vinylcyclohexyl]propan-2-ol | 19.03312062 | 222.410 | 0.072534 | GFJIQNADMLPFOW-VNHYZAJKSA-N | 92138 |
| CF.3 | MOL000041 | PHA | 41.61788444 | 165.210 | 0.036077 | COLNVLDHVKWLRT-QMMMGPOBSA-N | 6925665 |
| CF.4 | MOL000042 | LPG | 87.69200501 | 89.110 | 0.0059892 | QNAYBMKLOCPYGJ-REOHCLBHSA-N | 7311724 |
| CF.5 | MOL000050 | GLY | 48.73713548 | 75.080 | 0.0033178 | DHMQDGOQFOQNFH-UHFFFAOYSA-N | 5257127 |
| CF.6 | MOL000052 | Gulutamine | 6.655918569 | 147.150 | 0.021387 | WHUUTDBJXJRKMK-VKHMYHEASA-N | 44272391 |
| CF.7 | MOL000057 | DIBP | 49.6340827 | 278.380 | 0.13067 | MGWAVDBGNNKXQV-UHFFFAOYSA-N | 6782 |
| CF.8 | MOL000061 | Prolinum | 77.57468129 | 115.150 | 0.014161 | ONIBWKKTOPOVIA-BYPYZUCNSA-N | 6971047 |
| CF.9 | MOL000065 | ASI | 79.73701985 | 133.120 | 0.017132 | CKLJMWTZIZZHCS-REOHCLBHSA-N | 44367445 |
| CF.10 | MOL000068 | L-Ile | 59.05137302 | 131.200 | 0.015008 | AGPKZVBTJJNPAG-WHFBIAKZSA-N | 7043901 |
| CF.11 | MOL000069 | palmitic acid | 19.2965647 | 256.480 | 0.098573 | IPCSVZSSVZVIGE-UHFFFAOYSA-N | 985 |
| CF.12 | MOL000071 | Istidina | 53.17530628 | 155.180 | 0.029475 | HNDVDQJCIGZPNO-YFKPBYRVSA-N | 6971009 |
| CF.13 | MOL000105 | protocatechuic acid | 25.36646796 | 154.130 | 0.035092 | YQUVCSBJEUQKSH-UHFFFAOYSA-N | 72 |
| CF.14 | MOL000118 | (L)-alpha-Terpineol | 48.79777273 | 154.280 | 0.030772 | WUOACPNHFRMFPN-SECBINFHSA-N | 443162 |
| CF.15 | MOL000126 | (-)-nopinene | 44.83529174 | 136.260 | 0.052595 | WTARULDDTDQWMU-IUCAKERBSA-N | 440967 |
| CF.16 | MOL000131 | EIC | 41.90443602 | 280.500 | 0.14347 | OYHQOLUKZRVURQ-HZJYTTRNSA-N | 5280450 |
| CF.17 | MOL000172 | Furol | 34.35104869 | 96.090 | 0.010395 | HYBBIBNJHNGZAN-UHFFFAOYSA-N | 7362 |
| CF.18 | MOL000199 | Safrol | 45.3367099 | 162.200 | 0.047125 | ZMQAAUBTXCXRIC-UHFFFAOYSA-N | 5144 |
| CF.19 | MOL000206 | isoeugenol | 70.10031992 | 164.220 | 0.036329 | BJIOGJUNALELMI-ONEGZZNKSA-N | 853433 |
| CF.20 | MOL000207 | Methyleugenol | 73.36011441 | 178.250 | 0.042845 | ZYEMGPIYFIJGTP-UHFFFAOYSA-N | 7127 |
| CF.21 | MOL000223 | caffeic acid | 25.76439804 | 180.170 | 0.050089 | QAIPRVGONGVQAS-DUXPYHPUSA-N | 689043 |
| CF.22 | MOL000261 | Myristicin | 17.98539581 | 192.230 | 0.067649 | BNWJOHGLIBDBOB-UHFFFAOYSA-N | 4276 |
| CF.23 | MOL000263 | oleanolic acid | 29.02084142 | 456.780 | 0.75599 | MIJYXULNPSFWEK-GTOFXWBISA-N | 10494 |
| CF.24 | MOL000269 | Elemicin | 21.94067579 | 208.280 | 0.060865 | BPLQKQKXWHCZSS-UHFFFAOYSA-N | 10248 |
| CF.25 | MOL000305 | lauric acid | 23.58793922 | 200.360 | 0.043637 | POULHZVOKOAJMA-UHFFFAOYSA-N | 3893 |
| CF.26 | MOL000357 | Sitogluside | 20.63193686 | 576.950 | 0.6241 | NPJICTMALKLTFW-OFUAXYCQSA-N | 5742590 |
| CF.27 | MOL000358 | beta-sitosterol | 36.91390583 | 414.790 | 0.75123 | KZJWDPNRJALLNS-VJSFXXLFSA-N | 222284 |
| CF.28 | MOL000359 | sitosterol | 36.91390583 | 414.790 | 0.7512 | KZJWDPNRJALLNS-ZFVHJZABSA-N | 12303645 |
| CF.29 | MOL000431 | coumarin | 29.16755329 | 146.150 | 0.043014 | ZYGHJZDHTFUPRJ-UHFFFAOYSA-N | 323 |
| CF.30 | MOL000449 | Stigmasterol | 43.82985158 | 412.770 | 0.75665 | HCXVJBMSMIARIN-PHZDYDNGSA-N | 5280794 |
| CF.31 | MOL000475 | anethole | 32.492266 | 148.220 | 0.02782 | RUVINXPYWBROJD-ONEGZZNKSA-N | 637563 |
| CF.32 | MOL000511 | ursolic acid | 16.77490232 | 456.780 | 0.75457 | WCGUUGGRBIKTOS-GPOJBZKASA-N | 64945 |
| CF.33 | MOL000513 | 3,4,5-trihydroxybenzoic acid | 31.69129601 | 170.130 | 0.044562 | LNTHITQWFMADLM-UHFFFAOYSA-N | 370 |
| CF.34 | MOL000520 | alpha-amyrin | 10.2789795 | 426.800 | 0.76228 | FSLPMRQHCOLESF-SFMCKYFRSA-N | 73170 |
| CF.35 | MOL000554 | gallic acid-3-O-(6'-O-galloyl)-glucoside | 30.25032187 | 484.400 | 0.6746 | NRQUZRZEYPSZEY-IDXPAVDQSA-N | Not Available |
| CF.36 | MOL000570 | Nonox D | 39.82171868 | 219.300 | 0.13135 | KEQFTVQCIQJIQW-UHFFFAOYSA-N | 8679 |
| CF.37 | MOL000610 | TRD | 17.89122181 | 184.410 | 0.031021 | IIYFAKIEWZDVMP-UHFFFAOYSA-N | 12388 |
| CF.38 | MOL000635 | vanillin | 51.99600777 | 152.160 | 0.032596 | MWOOGOJBHIARFG-UHFFFAOYSA-N | 1183 |
| CF.39 | MOL000644 | swertiamarin_qt | 2.577561285 | 212.220 | 0.094881 | MTGJYYGNMXYVIV-SKWCMTHISA-N | Not Available |
| CF.40 | MOL000650 | 1H,3H-Pyrano(3,4-c)pyran-1-one, 5-ethenyl-6-(beta-D-glucopyranosyloxy)-4,4a,5,6-tetrahydro-, (4aS-(4aalpha,5beta,6alpha))- | 4.955220674 | 358.380 | 0.38457 | VSJGJMKGNMDJCI-ZASXJUAOSA-N | 161036 |
| CF.41 | MOL000651 | Sweroside aglycone | 68.6831176 | 196.220 | 0.075793 | HBAKFDGYROBYSH-BKPPORCPSA-N | 203797 |
| CF.42 | MOL000666 | hexanal | 55.70702938 | 100.180 | 0.0055795 | JARKCYVAAOWBJS-UHFFFAOYSA-N | 6184 |
| CF.43 | MOL000676 | DBP | 64.5416405 | 278.380 | 0.13409 | DOIRQSBPFJWKBE-UHFFFAOYSA-N | 3026 |
| CF.44 | MOL000704 | styrene | 29.54540857 | 104.160 | 0.013312 | PPBRXRYQALVLMV-UHFFFAOYSA-N | 7501 |
| CF.45 | MOL000749 | Linoleic | 41.90443602 | 280.500 | 0.14468 | OYHQOLUKZRVURQ-AVQMFFATSA-N | 5282457 |
| CF.46 | MOL000775 | EEE | 45.0163211 | 88.120 | 0.0045272 | XEKOWRVHYACXOJ-UHFFFAOYSA-N | 8857 |
| CF.47 | MOL000860 | stearic acid | 17.82542938 | 284.540 | 0.14086 | QIQXTHQIDYTFRH-UHFFFAOYSA-N | 5281 |
| CF.48 | MOL000864 | MYS | 13.9810568 | 212.470 | 0.04922 | YCOZIPAWZNQLMR-UHFFFAOYSA-N | 12391 |
| CF.49 | MOL000865 | hexadecane | 12.31600907 | 226.500 | 0.060928 | DCAYPVUWAIABOU-UHFFFAOYSA-N | 11006 |
| CF.50 | MOL000867 | Heptadekan | 8.642472374 | 240.530 | 0.074658 | NDJKXXJCMXVBJW-UHFFFAOYSA-N | 12398 |
| CF.51 | MOL000873 | CYH | 74.98596762 | 98.160 | 0.010105 | JHIVVAPYMSGYDF-UHFFFAOYSA-N | 7967 |
| CF.52 | MOL000879 | methyl palmitate | 18.08756063 | 270.510 | 0.11594 | FLIACVVOZYBSBS-UHFFFAOYSA-N | 8181 |
| CF.53 | MOL000885 | Dodekan | 17.73740163 | 170.380 | 0.02413 | SNRUBQQJIBEYMU-UHFFFAOYSA-N | 8182 |
| CF.54 | MOL000886 | tetradecane | 15.94226412 | 198.440 | 0.039319 | BGHCVCJVXZWKCC-UHFFFAOYSA-N | 12389 |
| CF.55 | MOL000890 | (+)-alpha-Curcumene | 26.56378766 | 202.370 | 0.055919 | VMYXUZSZMNBRCN-AWEZNQCLSA-N | 3083834 |
| CF.56 | MOL000971 | Ethylpalmitate | 18.98672237 | 284.540 | 0.13539 | XIRNKXNNONJFQO-UHFFFAOYSA-N | 12366 |
| CF.57 | MOL001300 | PEL | 44.03418878 | 122.180 | 0.017357 | WRMNZCZEMHIOCP-UHFFFAOYSA-N | 6054 |
| CF.58 | MOL001393 | myristic acid | 21.18117264 | 228.420 | 0.066784 | TUNFSRHWOTWDNC-UHFFFAOYSA-N | 11005 |
| CF.59 | MOL001394 | Oktadekan | 9.806386361 | 254.560 | 0.090617 | RZJRJXONCZWCBN-UHFFFAOYSA-N | 11635 |
| CF.60 | MOL001398 | Methyllinolenate | 46.15300681 | 292.510 | 0.17063 | DVWSXZIHSUZZKJ-YSTUJMKBSA-N | 5319706 |
| CF.61 | MOL001403 | ERUCAMIDE | 27.8451781 | 337.660 | 0.26008 | UAUDZVJPLUQNMU-KTKRTIGZSA-N | 5365371 |
| CF.62 | MOL001468 | MLT | 59.6169838 | 134.100 | 0.017766 | BJEPYKJPYRNKOW-REOHCLBHSA-N | 222656 |
| CF.63 | MOL001494 | Mandenol | 41.99620045 | 308.560 | 0.19321 | FMMOOAYVCKXGMF-MURFETPASA-N | 5282184 |
| CF.64 | MOL001495 | Ethyl linolenate | 46.10096327 | 306.540 | 0.19716 | JYYFMIOPGOFNPK-XSHSMGBESA-N | 6371716 |
| CF.65 | MOL001562 | Nonadecene | 5.233919033 | 266.570 | 0.111 | NHLUYCJZUXOUBX-UHFFFAOYSA-N | 29075 |
| CF.66 | MOL001599 | α-cubebol | 64.8052216 | 208.380 | 0.085702 | BOBTWVZHSPRDFC-OUCADQQQSA-N | Not Available |
| CF.67 | MOL001600 | copaene | 29.47338384 | 204.390 | 0.12401 | VLXDPFLIRFYIME-QRTUWBSPSA-N | Not Available |
| CF.68 | MOL001604 | Linalool | 49.367371 | 170.280 | 0.042561 | SATQWIIUJKWZNO-SCZZXKLOSA-N | Not Available |
| CF.69 | MOL001606 | BB_NC-0668 | 35.57214058 | 204.390 | 0.082297 | QEBNYNLSCGVZOH-NFAWXSAZSA-N | 9855795 |
| CF.70 | MOL001618 | Pellitorin | 23.80509944 | 223.400 | 0.061274 | MAGQQZHFHJDIRE-BNFZFUHLSA-N | 5318516 |
| CF.71 | MOL001619 | UPL | 8.520498415 | 268.590 | 0.10901 | LQERIDTXQFOHKA-UHFFFAOYSA-N | 12401 |
| CF.72 | MOL001680 | Loganin | 5.901127525 | 390.430 | 0.43563 | AMBQHHVBBHTQBF-UOUCRYGSSA-N | 87691 |
| CF.73 | MOL001681 | methyl (1R,4aS,6S,7R,7aS)-1,6-dihydroxy-7-methyl-1,4a,5,6,7,7a-hexahydrocyclopenta[d]pyran-4-carboxylate | 29.98748198 | 228.270 | 0.096848 | XWOHZIIPBYAMJX-KHBMLBSESA-N | 10466307 |
| CF.74 | MOL001682 | (1S,4aS,6R,8S,8aS)-6-hydroxy-8-methyl-1-[(2S,3R,4S,5S,6R)-3,4,5-trihydroxy-6-methylol-tetrahydropyran-2-yl]oxy-1,4a,5,6,8,8a-hexahydropyrano[4,3-d]pyran-4-carboxylic acid methyl ester | 13.86372707 | 406.430 | 0.49675 | YTZSBJLNMIQROD-SFBCHFHNSA-N | 11228693 |
| CF.75 | MOL001683 | morroniside_qt | 1.677258494 | 244.270 | 0.11735 | ZIMQGAMJDWXOIL-DANLAGSESA-N | Not Available |
| CF.76 | MOL001696 | Morusin | 11.5180776 | 420.490 | 0.75644 | XFFOMNJIDRDDLQ-UHFFFAOYSA-N | 5281671 |
| CF.77 | MOL001739 | zoomaric acid | 35.77585321 | 254.460 | 0.099334 | SECPZKHBENQXJG-FPLPWBNLSA-N | 445638 |
| CF.78 | MOL001746 | ELD | 31.19607818 | 281.540 | 0.13978 | FATBGEAMYMYZAF-KTKRTIGZSA-N | 5283387 |
| CF.79 | MOL001771 | poriferast-5-en-3beta-ol | 36.91390583 | 414.790 | 0.75034 | KZJWDPNRJALLNS-FBZNIEFRSA-N | 457801 |
| CF.80 | MOL001816 | Amide HPL | 19.7865465 | 255.500 | 0.096685 | HSEMFIZWXHQJAE-UHFFFAOYSA-N | 69421 |
| CF.81 | MOL001886 | Tar | 66.37583538 | 150.100 | 0.024845 | FEWJPZIEWOKRBE-LWMBPPNESA-N | 439655 |
| CF.82 | MOL001889 | Methyl linolelaidate | 41.93435814 | 294.530 | 0.16791 | WTTJVINHCBCLGX-ZDVGBALWSA-N | 5362793 |
| CF.83 | MOL001893 | BU3 | 34.87385841 | 90.140 | 0.0056516 | OWBTYPJTUOEWEK-QWWZWVQMSA-N | 225936 |
| CF.84 | MOL001906 | Methylgallate | 30.90793569 | 184.160 | 0.051049 | FBSFWRHWHYMIOG-UHFFFAOYSA-N | 7428 |
| CF.85 | MOL001996 | Betulonic acid | 16.83371616 | 454.760 | 0.77936 | SLJTWDNVZKIDAU-SVAFSPIFSA-N | 122844 |
| CF.86 | MOL002167 | WLN: T5OJ BVO1 | 49.40603481 | 126.120 | 0.01872 | HDJLSECJEQSPKW-UHFFFAOYSA-N | 11902 |
| CF.87 | MOL002250 | [(2R,3R,4S,5R,6R)-3,5-dihydroxy-2-(3,4,5-trihydroxybenzoyl)oxy-6-[(3,4,5-trihydroxybenzoyl)oxymethyl]oxan-4-yl] 3,4,5-trihydroxybenzoate | 3.013720692 | 636.510 | 0.54177 | RNKMOGIPOMVCHO-SVHODSNWSA-N | 11969003 |
| CF.88 | MOL002307 | 20-Hexadecanoylingenol | 28.20395415 | 586.940 | 0.68297 | DSTCZBGJCUOFLM-SXNKARFESA-N | Not Available |
| CF.89 | MOL002343 | tetrandrine | 26.63974111 | 622.820 | 0.10166 | WVTKBKWTSCPRNU-KYJUHHDHSA-N | Not Available |
| CF.90 | MOL002373 | gamma-tocopherol | 15.621042 | 416.760 | 0.51641 | QUEDXNHFTDJVIY-DQCZWYHMSA-N | 92729 |
| CF.91 | MOL002534 | 1,6-dimethyl-4-isopropyl-1,2,3,4,4a,7-hexahydronaphthalene | 17.14233587 | 204.390 | 0.077167 | JUQGWBAOQUBVFP-IPYPFGDCSA-N | Not Available |
| CF.92 | MOL002703 | OCTADECENE | 19.20878962 | 252.540 | 0.092277 | CCCMONHAUSKTEQ-UHFFFAOYSA-N | 8217 |
| CF.93 | MOL002778 | THZ | 70.3331389 | 135.200 | 0.025246 | IOJUPLGTWVMSFF-UHFFFAOYSA-N | 7222 |
| CF.94 | MOL002850 | butylated hydroxytoluene | 40.02026868 | 220.390 | 0.071985 | NLZUEZXRPGMBCV-UHFFFAOYSA-N | 31404 |
| CF.95 | MOL002879 | Diop | 43.59332547 | 390.620 | 0.39247 | IJFPVINAQGWBRJ-UHFFFAOYSA-N | 33934 |
| CF.96 | MOL002883 | Ethyl oleate (NF) | 32.39738821 | 310.580 | 0.19061 | LVGKNOAMLMIIKO-QXMHVHEDSA-N | 5363269 |
| CF.97 | MOL003010 | quercetin-3-o-beta-D-glu | 1.814813495 | 478.390 | 0.78674 | DUBCCGAQYVUYEU-GGTBVAQXSA-N | Not Available |
| CF.98 | MOL003080 | 2-METHYLPENTADECANE | 4.351720561 | 226.500 | 0.06027 | BANXPJUEBPWEOT-UHFFFAOYSA-N | 15267 |
| CF.99 | MOL003137 | Leucanthoside | 32.11589283 | 462.440 | 0.78146 | DLVLXOYLQKCAME-DGHBBABESA-N | 442659 |
| CF.100 | MOL003166 | Swertiamarin | 21.90382697 | 374.380 | 0.42213 | HEYZWPRKKUGDCR-QBXMEVCASA-N | 442435 |
| CF.101 | MOL003374 | Urushiol III | 3.57344416 | 316.530 | 0.24673 | RMTXUPIIESNLPW-AOSYACOCSA-N | 5281862 |
| CF.102 | MOL003484 | PEY | 25.69685654 | 178.240 | 0.097919 | YNPNZTXNASCQKK-UHFFFAOYSA-N | 995 |
| CF.103 | MOL003527 | Tyranton | 58.33645039 | 116.180 | 0.012407 | SWXVUIWOUIDPGS-UHFFFAOYSA-N | 31256 |
| CF.104 | MOL003546 | Aristolone | 45.30748966 | 218.370 | 0.13009 | UGVIZCBJCSXBCJ-JWFUOXDNSA-N | 165536 |
| CF.105 | MOL003547 | Azaron | 38.38574731 | 208.280 | 0.060667 | RKFAZBXYICVSKP-AATRIKPKSA-N | 636822 |
| CF.106 | MOL003573 | calacorene | 16.19720206 | 200.350 | 0.078209 | CUUMXRBKJIDIAY-ZDUSSCGKSA-N | 12302243 |
| CF.107 | MOL003788 | Cinnamein | 78.79524315 | 238.300 | 0.12088 | NGHOLYJTSCBCGC-VAWYXSNFSA-N | 5273469 |
| CF.108 | MOL003789 | Coumaran | 50.89557204 | 120.160 | 0.025593 | HBEDSQVIWPRPAY-UHFFFAOYSA-N | 10329 |
| CF.109 | MOL003877 | Fuseloel | 65.66591497 | 88.170 | 0.0041988 | PHTQWCKDNZKARW-UHFFFAOYSA-N | 31260 |
| CF.110 | MOL003940 | Stearamide | 18.44123491 | 283.560 | 0.13834 | LYRFLYHAGKPMFH-UHFFFAOYSA-N | 31292 |
| CF.111 | MOL003955 | D-Cystine | 39.58220648 | 240.340 | 0.048873 | LEVWYRKDKASIDU-IMJSIDKUSA-N | 6992103 |
| CF.112 | MOL003969 | L-Serin | 98.46910714 | 105.110 | 0.0091307 | MTCFGRXMJLQNBG-REOHCLBHSA-N | 6857581 |
| CF.113 | MOL003971 | Threonin | 73.51568361 | 119.140 | 0.012819 | AYFVYJQAPQTCCC-GBXIJSLDSA-N | 6971019 |
| CF.114 | MOL004048 | keto-L-fructose | 49.67181164 | 180.180 | 0.034443 | BJHIKXHVCXFQLS-FUTKDDECSA-N | 5460024 |
| CF.115 | MOL004284 | 2-Nonadecanone | 14.37970578 | 282.570 | 0.13569 | IEDKVDCIEARIIU-UHFFFAOYSA-N | 69423 |
| CF.116 | MOL004582 | Methyl naphthalene | 39.01126202 | 142.210 | 0.0413 | QPUYECUOLPXSFR-UHFFFAOYSA-N | 7002 |
| CF.117 | MOL004590 | 2-METHYLHEXADECANE | 4.192982019 | 240.530 | 0.073648 | FNWWOHKUXFTKGN-UHFFFAOYSA-N | 15266 |
| CF.118 | MOL004741 | (7aR)-4,4,7a-trimethyl-6,7-dihydro-5H-benzofuran-2-one | 40.47808891 | 180.270 | 0.065047 | IMKHDCBNRDRUEB-LLVKDONJSA-N | 6432173 |
| CF.119 | MOL004784 | Stenol | 12.65726871 | 270.560 | 0.11373 | GLDOVTGHNKAZLK-UHFFFAOYSA-N | 8221 |
| CF.120 | MOL005022 | 1,8-DIMETHYLNAPHTHALENE | 42.79667217 | 156.240 | 0.050481 | XAABPYINPXYOLM-UHFFFAOYSA-N | 11287 |
| CF.121 | MOL005272 | 13-Tetradecenyl acetate | 36.76038067 | 254.460 | 0.10041 | DZXBZPMJYIXTTI-UHFFFAOYSA-N | 521718 |
| CF.122 | MOL005306 | Acetal | 26.39611428 | 118.200 | 0.0087235 | DHKHKXVYLBGOIT-UHFFFAOYSA-N | 7765 |
| CF.123 | MOL005360 | malkangunin | 57.71384384 | 432.560 | 0.62642 | DTMIMKTZETWDJV-DUUKBJRLSA-N | Not Available |
| CF.124 | MOL005386 | Vulgarin | 29.21124048 | 264.350 | 0.1974 | NGPDZEACIWDCKX-WUDKWMPASA-N | 94253 |
| CF.125 | MOL005449 | h-Met-h | 70.86633942 | 149.240 | 0.013018 | FFEARJCKVFRZRR-BYPYZUCNSA-N | 6992087 |
| CF.126 | MOL005467 | Epicatechin gallate | 17.88586712 | 442.400 | 0.74627 | LSHVYAFMTMFKBA-FPOVZHCZSA-N | 65056 |
| CF.127 | MOL005468 | 1,2,3,6-tetra-O-galloyl-β-D-glucose | 3.013720692 | 788.620 | 0.33023 | RATQVALKDAUZBW-QYEBMHKLSA-N | Not Available |
| CF.128 | MOL005469 | 1,2,3-tri-O-galloyl-β-D-glucose | 3.013720692 | 636.510 | 0.57591 | MACFXELYCBWKGT-SASJIJFGSA-N | Not Available |
| CF.129 | MOL005470 | Durol | 17.73740163 | 134.240 | 0.025222 | SQNZJJAZBFDUTD-UHFFFAOYSA-N | 7269 |
| CF.130 | MOL005471 | [(2R,3R,4S,5S,6R)-4,5-dihydroxy-2-(3,4,5-trihydroxybenzoyl)oxy-6-[(3,4,5-trihydroxybenzoyl)oxymethyl]oxan-3-yl] 3,4,5-trihydroxybenzoate | 3.013720692 | 636.510 | 0.54091 | LLENXGNWVNSBQG-WPNCQGPHSA-N | 11969002 |
| CF.131 | MOL005472 | 1,2-Benzenedicarboxylicacid, mono(2-ethyl) hexylester | 55.16962094 | 278.380 | 0.13194 | DJDSLBVSSOQSLW-GFCCVEGCSA-N | Not Available |
| CF.132 | MOL005473 | 1,5-DIACETYLNAPHTHALENE | 11.59818415 | 212.260 | 0.098774 | XWZKPVAYNUXKCZ-UHFFFAOYSA-N | 219219 |
| CF.133 | MOL005474 | 1-Acetyl-4,6,8-trimethylazulene | 27.02153399 | 212.310 | 0.090065 | JUOAKPCWAPSQLK-UHFFFAOYSA-N | 607590 |
| CF.134 | MOL005475 | Chloroicosane | 10.43301904 | 317.060 | 0.15292 | AFGNVSCTEXUEJE-UHFFFAOYSA-N | 39150 |
| CF.135 | MOL005476 | Docosene | 16.73786266 | 308.660 | 0.18352 | SPURMHFLEKVAAS-UHFFFAOYSA-N | 74138 |
| CF.136 | MOL005477 | Eicosene | 17.82912378 | 280.600 | 0.13239 | VAMFXQBUQXONLZ-UHFFFAOYSA-N | 18936 |
| CF.137 | MOL005478 | 11,14-Octadecadienoic acid, methyl ester | 41.93435814 | 294.530 | 0.16862 | KXQNNBUXFKDSAX-HHWLVVFRSA-N | 5365677 |
| CF.138 | MOL005479 | pentatriacont-17-ene | 13.18621037 | 491.050 | 0.43259 | BLCUZCCTSBVFSV-LAPDZXRHSA-N | 5365022 |
| CF.139 | MOL005480 | Ucar AC | 60.16861296 | 104.170 | 0.0063478 | HCGFUIQPSOCUHI-UHFFFAOYSA-N | 7996 |
| CF.140 | MOL005481 | 2,6,10,14,18-pentamethylicosa-2,6,10,14,18-pentaene | 33.4041173 | 342.670 | 0.24028 | IMXDCJPVYKXJPD-FMOJUEAUSA-N | 5366013 |
| CF.141 | MOL005482 | FOA | 35.66235345 | 112.090 | 0.015249 | SMNDYUVBFMFKNZ-UHFFFAOYSA-N | 6919 |
| CF.142 | MOL005483 | 2-Methylnaphthalene | 33.68526746 | 142.210 | 0.040434 | QIMMUPPBPVKWKM-UHFFFAOYSA-N | 7055 |
| CF.143 | MOL005484 | GRO | 52.16269245 | 150.190 | 0.029914 | YPGCWEMNNLXISK-SSDOTTSWSA-N | 446626 |
| CF.144 | MOL005485 | 3-dibenzofuransulfonic acid | 74.42348454 | 248.270 | 0.15506 | QQYILFJVCFLPNB-UHFFFAOYSA-N | 3616629 |
| CF.145 | MOL005486 | 3,4-Dehydrolycopen-16-al | 46.64445252 | 548.920 | 0.4906 | FPLASDSFNINBIY-QETLVAEPSA-N | 5316458 |
| CF.146 | MOL005487 | 5-Carboxyresorcinol | 69.33779075 | 154.130 | 0.034392 | UYEMGAFJOZZIFP-UHFFFAOYSA-N | 7424 |
| CF.147 | MOL005488 | 3,5-Di-t-butyl-4-hydroxybenzaldehyde | 27.19067569 | 234.370 | 0.083939 | DOZRDZLFLOODMB-UHFFFAOYSA-N | 73219 |
| CF.148 | MOL005489 | 3,6-Digalloylglucose | 31.41521237 | 484.400 | 0.66343 | LRSHPKZSGRNHIX-MPWSSXMOSA-N | Not Available |
| CF.149 | MOL005490 | 4-(4-ethylphenyl)benzoic acid | 42.0591024 | 226.290 | 0.10276 | SCEBDBNGUCNRCE-UHFFFAOYSA-N | 521801 |
| CF.150 | MOL005491 | 4-Ethyl-o-xylene | 39.26539765 | 134.240 | 0.023389 | SBUYFICWQNHBCM-UHFFFAOYSA-N | 13629 |
| CF.151 | MOL005492 | 4-Methoxy-1,2-benzodioxole | 57.0768504 | 152.160 | 0.041332 | SMUNJBNGTHEKIO-UHFFFAOYSA-N | 5319407 |
| CF.152 | MOL005493 | 6-Isopropyl-1,4-dimethylnaphthalene | 33.79169682 | 198.330 | 0.079159 | AIZMBERBKBKQKL-UHFFFAOYSA-N | 603340 |
| CF.153 | MOL005494 | 6-Tetradecanesulfonic acid,butyl ester | 21.3623979 | 334.630 | 0.1335 | IUOLOMPNSNUSAF-SFHVURJKSA-N | Not Available |
| CF.154 | MOL005495 | 6-ethyl-2,5-dihydroxy-1,4-naphthoquinone | 20.58401075 | 218.220 | 0.10429 | IAOGCOUDTZVSLT-UHFFFAOYSA-N | 612788 |
| CF.155 | MOL005496 | 7,8-Dehydropenstemoside | 3.408230825 | 404.410 | 0.47063 | WUZGENDUAYSYJW-MEANJNGLSA-N | Not Available |
| CF.156 | MOL005497 | 7,8-Dehydropenstemoside_qt | 108.3253248 | 242.250 | 0.11913 | VLRPMHXSYJTZAU-YJFSRANCSA-N | Not Available |
| CF.157 | MOL005498 | 7-Hydroxycadalene | 29.30353404 | 214.330 | 0.093612 | RIWNMJBJRPCUBX-UHFFFAOYSA-N | 608115 |
| CF.158 | MOL005499 | 7-O-methylmorroniside | 3.982327749 | 406.430 | 0.50154 | DGNXCLIPLOECFN-GLWRDHDSSA-N | Not Available |
| CF.159 | MOL005500 | linolenate | 45.00906591 | 278.480 | 0.14777 | DTOSIQBPPRVQHS-IUQGRGSQSA-N | 5282822 |
| CF.160 | MOL005501 | Green Oil | 17.73740163 | 178.240 | 0.097161 | MWPLVEDNUUSJAV-UHFFFAOYSA-N | 8418 |
| CF.161 | MOL005502 | Butoxysuccinic Acid | 37.55432842 | 190.220 | 0.036664 | UDLOAJGWSDUKME-ZCFIWIBFSA-N | Not Available |
| CF.162 | MOL005503 | Cornudentanone | 39.6634055 | 378.560 | 0.327 | JIUGZSYPFREDLG-HXUWFJFHSA-N | 46191017 |
| CF.163 | MOL005504 | Cornusiin A | 7.951480532 | 1557.190 | 0.013682 | QVBGRJFHOYPNGA-NHCBRPIISA-N | Not Available |
| CF.164 | MOL005505 | Cornusiin B | 17.8352828 | 1086.770 | 0.014265 | NELJHVPUFBRAMZ-HSUKUTDESA-N | Not Available |
| CF.165 | MOL005506 | Cornusiin C | 3.013720692 | 784.570 | 0.14071 | UCTUGQCJSNXNNR-FEGNPHFYSA-N | Not Available |
| CF.166 | MOL005507 | Cornusiin G | 6.952788202 | 1725.300 | 0.0098068 | OXYNTHAZHTYEDD-VAZJVBODSA-N | Not Available |
| CF.167 | MOL005508 | Glucosol | 15.86390133 | 472.780 | 0.73993 | HFGSQOYIOKBQOW-ZSDYHTTISA-N | 6918774 |
| CF.168 | MOL005509 | 11-Cyclohexylheneicosane | 9.936215453 | 378.810 | 0.28252 | ZSEPDKTYQJRZIS-UHFFFAOYSA-N | 138812 |
| CF.169 | MOL005510 | D-1-O-Methyl mucoinositol | 25.10614311 | 194.210 | 0.053507 | DSCFFEYYQKSRSV-MAFUWASYSA-N | 5319730 |
| CF.170 | MOL005511 | D-delta-tocopherol | 16.35686239 | 402.730 | 0.48246 | GZIFEOYASATJEH-UMTXDNHDSA-N | 71257536 |
| CF.171 | MOL005512 | Dehydromorroniaglycone | 58.77599637 | 226.250 | 0.12967 | QGBCGMGBGAHJIT-LODYRLCVSA-N | Not Available |
| CF.172 | MOL005513 | DBF | 37.27949323 | 168.200 | 0.080246 | TXCDCPKCNAJMEE-UHFFFAOYSA-N | 568 |
| CF.173 | MOL005514 | Ethyl,alpha-hydroxymyristate | 18.71328363 | 272.480 | 0.11059 | ILJLABRQPCOYHY-OAHLLOKOSA-N | Not Available |
| CF.174 | MOL005515 | Eugenone | 18.696917 | 252.290 | 0.09996 | ZYRBXTNFHYZHSK-UHFFFAOYSA-N | 5317271 |
| CF.175 | MOL005516 | Fluoren | 18.94218178 | 166.230 | 0.07896 | NIHNNTQXNPWCJQ-UHFFFAOYSA-N | 6853 |
| CF.176 | MOL005517 | 2-METHYLHEPTADECANE | 10.56744509 | 254.560 | 0.089658 | RJWUMFHQJJBBOD-UHFFFAOYSA-N | 15265 |
| CF.177 | MOL005518 | Heptadecane,3-methyl | 10.56744509 | 254.560 | 0.088431 | HPDKJRSKBCPMIY-GOSISDBHSA-N | Not Available |
| CF.178 | MOL005519 | (7R)-7-methylheptadecane | 10.56744509 | 254.560 | 0.084034 | AZGIFKCGYRMPKP-GOSISDBHSA-N | 10848577 |
| CF.179 | MOL005520 | Heptadecane,8-methyl | 10.56744509 | 254.560 | 0.082515 | AFKUSTCGONJZHE-GOSISDBHSA-N | Not Available |
| CF.180 | MOL005521 | phytane | 13.86308864 | 282.620 | 0.10838 | GGYKPYDKXLHNTI-VAMGGRTRSA-N | Not Available |
| CF.181 | MOL005522 | LEN | 62.94047266 | 87.190 | 0.0040485 | BMFVGAAISNGQNM-UHFFFAOYSA-N | 7894 |
| CF.182 | MOL005523 | Nonadecane,2,3-di methyl | 11.59658191 | 296.650 | 0.14798 | JXKFQCQTFUPIFF-NRFANRHFSA-N | Not Available |
| CF.183 | MOL005524 | Nonadecane,2,6,10,14,18-pentamethyl, | 13.99297312 | 338.740 | 0.19794 | OJXBLWMIJXWKQV-BKFWDETESA-N | Not Available |
| CF.184 | MOL005525 | Octadecane,2,6,10,14-tetramethyl- | 13.58407279 | 310.680 | 0.14798 | PUTSHCSOTBWQAI-YPAWHYETSA-N | Not Available |
| CF.185 | MOL005526 | Octadecane,3-methyl | 10.42485445 | 268.590 | 0.10524 | PGZRTPKNSFMAOP-LJQANCHMSA-N | Not Available |
| CF.186 | MOL005527 | Octadecane,6-methyl | 10.42485445 | 268.590 | 0.10258 | MMQAZWNADBJFCD-LJQANCHMSA-N | Not Available |
| CF.187 | MOL005528 | 2,6,10,14-tetramethylpentadecane | 3.60206877 | 268.590 | 0.091543 | XOJVVFBFDXDTEG-KDURUIRLSA-N | 25022101 |
| CF.188 | MOL005529 | Pentadecane,2,6,10-trimethyl | 13.33306511 | 254.560 | 0.077831 | LBWPYRZGHYVSEL-ROUUACIJSA-N | Not Available |
| CF.189 | MOL005530 | Hydroxygenkwanin | 36.46699689 | 300.280 | 0.27206 | RRRSSAVLTCVNIQ-UHFFFAOYSA-N | 5318214 |
| CF.190 | MOL005531 | Telocinobufagin | 69.99386894 | 402.580 | 0.79297 | PBSOJKPTQWWJJD-XECVOWJVSA-N | Not Available |
| CF.191 | MOL005532 | Tetradecane,2,6,10-trimethyl | 3.931959175 | 240.530 | 0.064203 | IMTCMWSWXFQQDL-IAGOWNOFSA-N | Not Available |
| CF.192 | MOL005534 | trans-Verbenol | 52.00798167 | 152.260 | 0.061515 | WONIGEXYPVIKFS-DJLDLDEBSA-N | 88298 |
| CF.193 | MOL005535 | Vitamin B1.mol2 | 49.33100917 | 266.410 | 0.11237 | CUHJDHQZPBVYHJ-UHFFFAOYSA-N | Not Available |
| CF.194 | MOL005536 | Z,Z-10,12-Hexadecadien-1-ol acetate | 44.09202043 | 280.500 | 0.14453 | CMCBHGAXGCXMIP-ISTTXYCBSA-N | 5363372 |
| CF.195 | MOL005537 | Z-5-Nonadecene | 18.80658755 | 266.570 | 0.10981 | LHPVEDYVSCTZAY-LUAWRHEFSA-N | 5364560 |
| CF.196 | MOL005538 | Linolenyl alcohol | 42.79174531 | 264.500 | 0.11781 | IKYKEVDKGZYRMQ-PDBXOOCHSA-N | 6436081 |
| CF.197 | MOL005539 | Acenaphthylene | 28.55222781 | 152.200 | 0.06674 | HXGDTGSAIMULJN-UHFFFAOYSA-N | 9161 |
| CF.198 | MOL005540 | alpha-Corocalene | 13.97461976 | 200.350 | 0.078132 | VTUZIFHLLUSULC-UHFFFAOYSA-N | 5316074 |
| CF.199 | MOL005541 | TBU | 60.44633866 | 74.140 | 0.0044962 | DKGAVHZHDRPRBM-UHFFFAOYSA-N | 6386 |
| CF.200 | MOL005542 | camptothin A | 6.267740395 | 1571.170 | 0.018301 | CGTQPVKZNROYFH-SKEITSDKSA-N | Not Available |
| CF.201 | MOL005543 | camptothin B | 3.013720692 | 1086.770 | 0.04115 | IBUZLJOVINHPCM-IMELRQICSA-N | Not Available |
| CF.202 | MOL005544 | Cornin | 12.68527831 | 388.410 | 0.43901 | HLXRWTJXGMHOFN-XJSNKYLASA-N | 73467 |
| CF.203 | MOL005545 | cornin_qt | 25.1044089 | 226.250 | 0.097726 | ICLHTGIHDLYEDX-PPZZJSARSA-N | 12444745 |
| CF.204 | MOL005546 | cornuside | 2.610111139 | 542.540 | 0.70539 | SMTKSCGLXONVGL-MUCSSEFLSA-N | Not Available |
| CF.205 | MOL005547 | cornuside_qt | 2.372602589 | 380.380 | 0.39489 | KDGCSTFYBWGBNX-PJYBLOJUSA-N | Not Available |
| CF.206 | MOL005548 | cornusiin E | 7.366428876 | 1857.370 | 0.0020114 | LXPDKGXZXITTJG-LAARJLNFSA-N | Not Available |
| CF.207 | MOL005549 | Dehydromevalonic lactone | 53.46135969 | 112.140 | 0.015077 | RPEASMBMVIKUTH-UHFFFAOYSA-N | 557445 |
| CF.208 | MOL005550 | Vanirom | 28.06661259 | 166.190 | 0.038176 | CBOQJANXLMLOSS-UHFFFAOYSA-N | 8467 |
| CF.209 | MOL005552 | gemin D | 68.8303535 | 634.490 | 0.56075 | XKVYZLLWKHGKMT-UPMOLNEASA-N | Not Available |
| CF.210 | MOL005553 | 2-Monopalmitin | 26.73806656 | 330.570 | 0.21662 | BBNYCLAREVXOSG-UHFFFAOYSA-N | 123409 |
| CF.211 | MOL005554 | i-Butanol | 28.2618118 | 74.140 | 0.0027413 | ZXEKIIBDNHEJCQ-UHFFFAOYSA-N | 6560 |
| CF.212 | MOL005555 | isoterchebin | 3.013720692 | 954.700 | 0.085217 | QFJVFTCFAZSXCY-NFSXZKMWSA-N | Not Available |
| CF.213 | MOL005556 | 1-tert-Butyl-7-methoxynaphthalene | 27.4637603 | 214.330 | 0.093603 | KGIHXPXSMAHFPD-UHFFFAOYSA-N | 608110 |
| CF.214 | MOL005557 | lanosta-8,24-dien-3-ol,3-acetate | 44.29553995 | 468.840 | 0.82425 | BQPPJGMMIYJVBR-MJGQBHONSA-N | Not Available |
| CF.215 | MOL005558 | DLE | 55.10487226 | 131.200 | 0.014068 | ROHFNLRQFUQHCH-RXMQYKEDSA-N | 6950207 |
| CF.216 | MOL005559 | Maslinic acid | 15.54246865 | 472.780 | 0.74138 | MDZKJHQSJHYOHJ-LLICELPBSA-N | 73659 |
| CF.217 | MOL005560 | olean-13(18)-ene | 6.595109339 | 410.800 | 0.76195 | GNSWPULAOZONLL-ICNADSGASA-N | Not Available |
| CF.218 | MOL005561 | Anisylacetone | 19.69110101 | 178.250 | 0.043219 | PCBSXBYCASFXTM-UHFFFAOYSA-N | 61007 |
| CF.219 | MOL005562 | phosphonofluoridic acid,(l-methylethyl)-,cyclohexyl ester | 76.0046214 | 208.240 | 0.042722 | BSSKRMAEPPGXAO-ZDUSSCGKSA-N | Not Available |
| CF.220 | MOL005563 | (-)-Pulegone | 51.22948618 | 152.260 | 0.029704 | NZGWDASTMWDZIW-QMMMGPOBSA-N | 638012 |
| CF.221 | MOL005564 | tellimagrandin I | 3.013720692 | 786.590 | 0.31692 | XUZYVFYOPRXTRB-YWZBFRKASA-N | Not Available |
| CF.222 | MOL005565 | Uretan | 3.97184233 | 89.110 | 0.0047502 | JOYRKODLDBILNP-UHFFFAOYSA-N | 5641 |
| CF.223 | MOL005566 | urs-12-en-28-al | 8.659742932 | 424.780 | 0.76838 | YJVXDRHQRLHFGY-PTXBVUTMSA-N | Not Available |
| CF.224 | MOL005567 | vitamin a | 19.52765079 | 286.500 | 0.15689 | FPIPGXGPPPQFEQ-OVSJKPMPSA-N | 445354 |
| CF.225 | MOL007019 | Eugeniin | 10.06143672 | 938.700 | 0.12895 | JCGHAEBIBSEQAD-UUUCSUBKSA-N | Not Available |
| CF.226 | MOL008457 | Tetrahydroalstonine | 32.41977527 | 352.470 | 0.81311 | GRTOGORTSDXSFK-DLLGKBFGSA-N | Not Available |
| PRR.1 | MOL000069 | palmitic acid | 19.2965647 | 256.480 | 0.098573 | IPCSVZSSVZVIGE-UHFFFAOYSA-N | 985 |
| PRR.2 | MOL000106 | PYG | 22.97788227 | 126.120 | 0.022766 | WQGWDDDVZFFDIG-UHFFFAOYSA-N | 1057 |
| PRR.3 | MOL000114 | vanillic acid | 35.47235319 | 168.160 | 0.040917 | WKOLLVMJNQIZCI-UHFFFAOYSA-N | 8468 |
| PRR.4 | MOL000131 | EIC | 41.90443602 | 280.500 | 0.14347 | OYHQOLUKZRVURQ-HZJYTTRNSA-N | 5280450 |
| PRR.5 | MOL000219 | BOX | 31.54641411 | 121.120 | 0.020166 | WPYMKLBDIGXBTP-UHFFFAOYSA-N | 20144841 |
| PRR.6 | MOL000357 | Sitogluside | 20.63193686 | 576.950 | 0.6241 | NPJICTMALKLTFW-OFUAXYCQSA-N | 5742590 |
| PRR.7 | MOL000358 | beta-sitosterol | 36.91390583 | 414.790 | 0.75123 | KZJWDPNRJALLNS-VJSFXXLFSA-N | 222284 |
| PRR.8 | MOL000359 | sitosterol | 36.91390583 | 414.790 | 0.7512 | KZJWDPNRJALLNS-ZFVHJZABSA-N | 12303645 |
| PRR.9 | MOL000361 | Amyrin | 17.60191826 | 426.800 | 0.7633 | JFSHUTJDVKUMTJ-QHPUVITPSA-N | 73145 |
| PRR.10 | MOL000449 | Stigmasterol | 43.82985158 | 412.770 | 0.75665 | HCXVJBMSMIARIN-PHZDYDNGSA-N | 5280794 |
| PRR.11 | MOL000463 | 16844-71-6 | 27.33568744 | 428.820 | 0.75524 | XCDQFROEGGNAER-PFOIMGGJSA-N | 119242 |
| PRR.12 | MOL000492 | (+)-catechin | 54.82643405 | 290.290 | 0.24164 | PFTAWBLQPZVEMU-DZGCQCFKSA-N | 9064 |
| PRR.13 | MOL000508 | Friedelin | 29.16218092 | 426.800 | 0.75897 | OFMXGFHWLZPCFL-SVRPQWSVSA-N | 91472 |
| PRR.14 | MOL000513 | 3,4,5-trihydroxybenzoic acid | 31.69129601 | 170.130 | 0.044562 | LNTHITQWFMADLM-UHFFFAOYSA-N | 370 |
| PRR.15 | MOL000578 | arbutin | 6.815629908 | 272.280 | 0.14592 | BJRNKVDFDLYUGJ-MTVMDMGHSA-N | 40467854 |
| PRR.16 | MOL000579 | hydroquinone | 29.2590351 | 110.120 | 0.015607 | QIGBRXMKCJKVMJ-UHFFFAOYSA-N | 785 |
| PRR.17 | MOL000874 | paeonol | 28.78723811 | 166.190 | 0.039185 | UILPJVPSNHJFIK-UHFFFAOYSA-N | 11092 |
| PRR.18 | MOL001002 | ellagic acid | 43.06455858 | 302.200 | 0.43417 | AFSDNFLWKVMVRB-UHFFFAOYSA-N | Not Available |
| PRR.19 | MOL001801 | salicylic acid | 32.12698718 | 138.130 | 0.027213 | YGSDEFSMJLZEOE-UHFFFAOYSA-N | 338 |
| PRR.20 | MOL001906 | Methylgallate | 30.90793569 | 184.160 | 0.051049 | FBSFWRHWHYMIOG-UHFFFAOYSA-N | 7428 |
| PRR.21 | MOL001907 | Progallin A | 25.61474283 | 198.190 | 0.058527 | VFPFQHQNJCMNBZ-UHFFFAOYSA-N | 13250 |
| PRR.22 | MOL001918 | paeoniflorgenone | 87.59312084 | 318.350 | 0.36678 | BANPEMKDTXIFRE-GHVWTTSJSA-N | Not Available |
| PRR.23 | MOL001921 | Lactiflorin | 49.12131675 | 462.490 | 0.79711 | KEMSOUIGHYEWRY-UAJYNZJHSA-N | Not Available |
| PRR.24 | MOL001924 | paeoniflorin | 53.87037516 | 480.510 | 0.78709 | YKRGDOXKVOZESV-WRJNSLSBSA-N | Not Available |
| PRR.25 | MOL001925 | paeoniflorin_qt | 68.17576188 | 318.350 | 0.39507 | GWQHMWOOQLVRLG-JGAAPJFWSA-N | Not Available |
| PRR.26 | MOL001932 | galloylpaeoniflorin | 3.028419263 | 632.620 | 0.42335 | KLFIUQCKSSAFFU-JRWJHFJZSA-N | Not Available |
| PRR.27 | MOL002714 | baicalein | 33.51891869 | 270.250 | 0.20888 | FXNFHKRTJBSTCS-UHFFFAOYSA-N | 5281605 |
| PRR.28 | MOL002776 | Baicalin | 40.12360996 | 446.390 | 0.75264 | IKIIZLYTISPENI-ZFORQUDYSA-N | 64982 |
| PRR.29 | MOL003867 | Paeonolide | 6.298773621 | 460.480 | 0.6449 | IDZZECHGWAZTIB-NYBIBFQCSA-N | 442923 |
| PRR.30 | MOL004355 | Spinasterol | 42.97936552 | 412.770 | 0.75534 | JZVFJDZBLUFKCA-FXIAWGAOSA-N | 5281331 |
| PRR.31 | MOL005090 | oxypaeoniflorin_qt | 19.39679608 | 334.350 | 0.44099 | FICRKDWVMQNZMX-JGAAPJFWSA-N | Not Available |
| PRR.32 | MOL006179 | 2-(6-carboxy-2,3,4-trihydroxyphenyl)-3,4,5-trihydroxybenzoic acid | 15.20582542 | 338.240 | 0.26955 | MFTSECOLKFLUSD-UHFFFAOYSA-N | 10315050 |
| PRR.33 | MOL006765 | peonidin | 26.91534804 | 301.290 | 0.27286 | LVYJSZMNOULQRF-UHFFFAOYSA-N | Not Available |
| PRR.34 | MOL006798 | pedunculagin | 37.81377189 | 784.570 | 0.074325 | IYMHVUYNBVWXKH-GVVCPQIVSA-N | Not Available |
| PRR.35 | MOL006990 | (1S,2S,4R)-trans-2-hydroxy-1,8-cineole-B-D-glucopyranoside | 30.25241156 | 332.440 | 0.27464 | NWZYTZHMCGWGOF-KOQJZNESSA-N | Not Available |
| PRR.36 | MOL006991 | 60761-00-4 | 45.4401755 | 170.280 | 0.059727 | YVCUGZBVCHODNB-WEDXCCLWSA-N | 109010 |
| PRR.37 | MOL006992 | (2R,3R)-4-methoxyl-distylin | 59.98325098 | 318.300 | 0.29949 | ITWRRUUKFUXICF-CVEARBPZSA-N | Not Available |
| PRR.38 | MOL006993 | 1-o-beta-d-glucopyranosyl-8-o-benzoylpaeonisuffrone | 12.6232156 | 464.510 | 0.76809 | XEXJEQNKBLWQDB-MENCKEIZSA-N | Not Available |
| PRR.39 | MOL006994 | 1-o-beta-d-glucopyranosyl-8-o-benzoylpaeonisuffrone_qt | 36.01305796 | 302.350 | 0.29897 | RZPMQFMUQXXSKB-VDNDLQMASA-N | Not Available |
| PRR.40 | MOL006995 | 1-o-beta-d-glucopyranosylpaeonisuffrone | 26.62011946 | 494.540 | 0.7824 | CUOSPKQOVDKAPD-ASLXHAQJSA-N | Not Available |
| PRR.41 | MOL006996 | 1-o-beta-d-glucopyranosylpaeonisuffrone_qt | 65.08186655 | 332.380 | 0.35391 | GHLQXVJMYVGPCU-WYGQYTNYSA-N | Not Available |
| PRR.42 | MOL006997 | 2-[(2R,5R,6R)-6,10-dimethylspiro[4.5]dec-9-en-2-yl]propan-2-ol | 18.44250883 | 222.410 | 0.08708 | ICWHTQRTTHCUHW-NFAWXSAZSA-N | 21675005 |
| PRR.43 | MOL006999 | stigmast-7-en-3-ol | 37.42312067 | 414.790 | 0.75088 | YSKVBPGQYRAUQO-NPAMSQCVSA-N | Not Available |
| PRR.44 | MOL007000 | 2-methoxy-5-(e)-propenyl-phenol-beta-vicianoside | 4.381743993 | 458.510 | 0.64613 | KELQHXBBNXFZEP-LZPNQAPJSA-N | Not Available |
| PRR.45 | MOL007001 | 2-methoxy-5-[(Z)-prop-1-enyl]phenol | 58.73270456 | 164.220 | 0.036349 | LHJZSWVADJCBNI-ARJAWSKDSA-N | 1781945 |
| PRR.46 | MOL007002 | paeonioflorin | 10.22068492 | 480.510 | 0.7872 | YKRGDOXKVOZESV-QVMPQSNJSA-N | Not Available |
| PRR.47 | MOL007003 | benzoyl paeoniflorin | 31.13866577 | 584.620 | 0.54227 | LATYEZNGPQKAIK-RZRIYOMWSA-N | Not Available |
| PRR.48 | MOL007004 | Albiflorin | 30.24614292 | 480.510 | 0.77038 | QQUHMASGPODSIW-BNAVNNOTSA-N | Not Available |
| PRR.49 | MOL007005 | Albiflorin_qt | 48.70011649 | 318.350 | 0.32628 | WSVOZDIRZKFUCH-GVDRCSPZSA-N | Not Available |
| PRR.50 | MOL007006 | oxypaeoniflorin | 12.9810553 | 496.510 | 0.782 | FCHVXNVDFYXLIL-QVMPQSNJSA-N | Not Available |
| PRR.51 | MOL007007 | 4-ethyl-paeoniflorin | 24.81132743 | 494.540 | 0.81551 | FCCQSHXCIDUVRV-OPWFMGFHSA-N | Not Available |
| PRR.52 | MOL007008 | 4-ethyl-paeoniflorin_qt | 56.86957727 | 332.380 | 0.44483 | UPDCGAQEQJAPLA-NLHADHJGSA-N | Not Available |
| PRR.53 | MOL007009 | 4-o-galloylalbiflorin | 3.09221726 | 632.620 | 0.60866 | UXHIYEMICNYJGK-FCOMPSCZSA-N | Not Available |
| PRR.54 | MOL007010 | 4-o-galloylalbiflorin_qt | 2.135851234 | 470.460 | 0.79547 | XDVBNXQBFUDMQN-VBHKTOHQSA-N | Not Available |
| PRR.55 | MOL007011 | 4-o-methyl-paeoniflorin | 25.70632536 | 494.540 | 0.78445 | HBUSZOJOEGAJCJ-NHAMKOHOSA-N | Not Available |
| PRR.56 | MOL007012 | 4-o-methyl-paeoniflorin_qt | 56.70351745 | 332.380 | 0.42562 | LKWXNVNXHRSLRT-HZYJHYJVSA-N | Not Available |
| PRR.57 | MOL007014 | 8-debenzoylpaeonidanin | 31.74314844 | 390.430 | 0.45389 | FICAFDZLGACQCA-GTDRRMEFSA-N | Not Available |
| PRR.58 | MOL007015 | 8-debenzoylpaeonidanin_qt | 129.3034165 | 228.270 | 0.14565 | GFWJYUIWMJQGLU-IIALBSNWSA-N | Not Available |
| PRR.59 | MOL007016 | Paeoniflorigenone | 65.3341131 | 318.350 | 0.36711 | BANPEMKDTXIFRE-LQPBRMSDSA-N | Not Available |
| PRR.60 | MOL007017 | 9-ethyl-neo-paeoniaflorin A | 23.63578332 | 496.560 | 0.67544 | VVZAYARTDZDVMY-JEQNFFGSSA-N | Not Available |
| PRR.61 | MOL007018 | 9-ethyl-neo-paeoniaflorin A_qt | 64.41989313 | 334.400 | 0.29598 | LNQMUAGNQVZCET-BODMPHMZSA-N | Not Available |
| PRR.62 | MOL007019 | Eugeniin | 10.06143672 | 938.700 | 0.12895 | JCGHAEBIBSEQAD-UUUCSUBKSA-N | Not Available |
| PRR.63 | MOL007020 | Lactiflorin_qt | 20.44419431 | 318.350 | 0.32506 | AASZOTTYVMGDQA-MCPLSKDVSA-N | Not Available |
| PRR.64 | MOL007021 | trans-.beta.-Terpinyl benzoate | 46.88781556 | 258.390 | 0.12283 | CTROGWVSSFFUJS-UHFFFAOYSA-N | 570530 |
| PRR.65 | MOL007022 | evofolinB | 64.73661695 | 318.350 | 0.22232 | QMYGRGKKZBRZKH-LBPRGKRZSA-N | Not Available |
| PRR.66 | MOL007023 | galloylpaeoniflorine | 3.028419263 | 632.620 | 0.4233 | KLFIUQCKSSAFFU-CUVIGFRKSA-N | Not Available |
| PRR.67 | MOL007024 | galloylpaeoniflorine_qt 2 | 28.17539414 | 318.350 | 0.395 | GWQHMWOOQLVRLG-IAPUWKBBSA-N | Not Available |
| PRR.68 | MOL007025 | isobenzoylpaeoniflorin | 31.13866577 | 584.620 | 0.54234 | LATYEZNGPQKAIK-SYYPVXOBSA-N | Not Available |
| PRR.69 | MOL007026 | paeonin,a | 20.34604497 | 372.410 | 0.36898 | RNAOSOMTAGOVSH-LBOLTGTOSA-N | Not Available |
| PRR.70 | MOL007027 | paeonin,a_qt | 73.79042301 | 210.250 | 0.087206 | ZSBJUXRXUSRBEG-MWLCHTKSSA-N | Not Available |
| PRR.71 | MOL007028 | paeonin,b | 17.28582054 | 358.380 | 0.33542 | JJUACHJWALUCMP-JLMIEGKISA-N | Not Available |
| PRR.72 | MOL007029 | paeonin,b_qt | 105.4459733 | 196.220 | 0.077163 | PAVFQMYHZSGPSN-PSASIEDQSA-N | Not Available |
| PRR.73 | MOL007030 | paeonin,c | 10.25770016 | 372.410 | 0.36127 | OMNBOKMNIVQDNN-IWEBEACQSA-N | Not Available |
| PRR.74 | MOL007031 | paeonin,c_qt | 72.69758769 | 210.250 | 0.087628 | KWIXKLIBOCGXPF-FBIMIBRVSA-N | Not Available |
| PRR.75 | MOL009092 | Pentagalloylglucose | 3.013720692 | 940.720 | 0.20921 | QJYNZEYHSMRWBK-NIKIMHBISA-N | 65238 |
